# Stereoselective Synthesis of Fluoroalkanes via FLP Mediated Monoselective C─F Activation of Geminal Difluoroalkanes

**DOI:** 10.1002/advs.202305768

**Published:** 2023-10-31

**Authors:** Dániel Csókás, Bivas Mondal, Miloš Đokić, Richa Gupta, Beatrice J. Y. Lee, Rowan D. Young

**Affiliations:** ^1^ Department of Chemistry National University of Singapore Singapore 117543 Singapore; ^2^ Research Centre for Natural Sciences Institute of Organic Chemistry Budapest 1117 Hungary; ^3^ School of Chemistry and Molecular Biosciences The University of Queensland St Lucia 4067 Australia

**Keywords:** asymmetric synthesis, carbon–fluorine activation, difluoromethyl desymmetrization, frustrated Lewis pair, stereoselective

## Abstract

A method of desymmetrization of geminal difluoroalkanes using frustrated Lewis pair (FLP) mediated monoselective C–F activation where a chiral sulfide is the Lewis base component is reported. The stereoselective reaction provides generally high yields of diastereomeric sulfonium salts with *dr* of up to 95:5. The distribution of diastereomers is found to be thermodynamically controlled via facile sulfide exchange. The use of enantiopure chiral sulfides allows for high stereospecificity in nucleophilic substitution reactions and the formation of stereoenriched products.

## Graphical abstract

Methods for the installation of stereoenriched fluorocarbon centers are underdeveloped. A method is reported that allows for the stereoselective activation of enantiotopic fluoride positions in *geminal* difluoromethyl groups utilizing a frustrated Lewis pair approach. Access to a range of stereoenriched fluorocarbons, including drug analogs, is demonstrated using the approach.

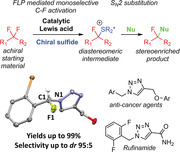

## 1 Introduction

Fluorocarbons are exceedingly rare in biology.^[^ ^1^ ^]^ Nonetheless, fluorine has had a significant impact on modern biological systems through synthetic compounds.^[^ ^2^ ^]^ Importantly, fluorine and fluorine containing groups act as bioisosteres, allowing simple access to chemical antagonists and agonists. Fluorine (or a fluorine containing group) is known to emulate hydrogen, alkyl, hydroxyl, and amide groups (inter alia).^[^ ^2^ ^]^ In a biological context, apart from chemical isostericity, stereochemistry is intrinsically important to the design of effective antagonists/agonists. Thus, it is surprising that methods for stereoselective installation of fluorine into organic molecules are belatedly undeveloped.^[^ ^3^ ^]^

**Figure 1:**
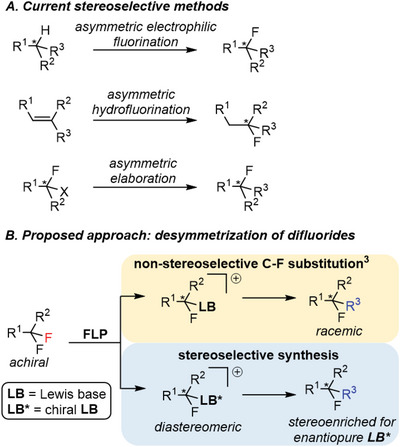
A) Examples of current methods to access chiral C─F centers. B) We have shown that FLP mediated monoselective C─F activation can generate racemic fluorocarbon products. We propose the use of chiral Lewis bases to generate diastereomeric products in a stereoselective manner.

Methods that generate chiral centers at C─F positions rely upon the stereoselective introduction of fluorine via proton substitution with electrophilic fluorine, stereoselective addition of nucleophilic fluoride (commonly across unsaturated bonds), or stereoselective elaborations of monofluorides (**Figure** **1**A). Stereoselective fluorination often suffers from the need to use toxic/hazardous fluorine sources, to employ starting materials with either acidic hydrogen positions (normally subtended by electron withdrawing groups) or starting materials with unsaturated bonds (across that HF can add), and a high incompatibility of electrophilic fluorine with many functional groups, while stereoselective elaborations are generally restricted to hydro‐ or bromo‐functionalization at α‐fluorocarbonyl positions.^[^ ^3a^ ^]^ Notably, despite the synthesis of benzyl fluorides being routine,^[^ ^4^ ^]^ the stereoselective generation of enantioenriched benzyl fluorides remains challenging.^[^ ^5^ ^]^

As part of our contribution to the field of Lewis acid mediated C─F bond activatrion,^[^ ^6^ ^]^ we previously reported frustrated Lewis pair (FLP) mediated monoselective activation of *gem*‐difluoromethyl groups generating chiral carbon centers in racemic mixtures.^[^ ^7^ ^]^ The stereoselective functionalization of an enantiotopic fluorine allows access to a wide variety of enantio and/or diastereo enriched products from a common precursor (Figure 1B).^[^ ^8^ ^]^

Previously, stereoselective FLP catalysis has been largely restricted to reductions of imines, ketones, and enones, and it has focused on employing a chiral Lewis acid to impart stereoselectivity to products.^[^ ^9^ ^]^ Generally, because the FLP base partners used in the activation of the reducing agent are (or compete with) the achiral substrates.^[^ ^10^ ^]^ In contrast, FLP mediated C─F bond activation relies upon capture of the activated fragment by a suitable Lewis base, and the substrates do not commonly act as FLP components. As such, we saw an opportunity to utilize affordable, stable, and easily accessible (or commercially available) chiral sulfide Lewis bases to induce stereoselectivity. Such an approach is a departure from pre‐existing approaches to FLP enantioselective catalysis and represents the first example of FLP stereoselective activation of C─F bonds.

## 2 Results and Discussion

**Figure 2:**
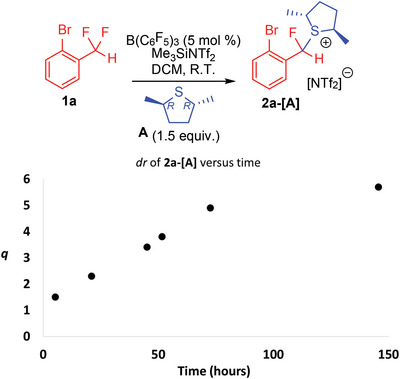
FLP mediated monoselective C─F activation of **1a** utilizing the enantiopure thiolane **A** as the Lewis base partner. The *dr* of the reaction improves from *q* = 1.5 (2 h) to *q* = 5.7 (144 h).

To provide proof‐of‐principle for this concept, we utilized the enantiopure base (*R,R*)−2,5‐dimethylthiolane (**A**)^[^ ^11^ ^]^ with previously reported catalytic conditions.^[^ ^7b^ ^]^ Application of such conditions to 1‐Br‐2‐(CF_2_H)‐C_6_H_4_ (**1a**) resulted in diastereomeric products **2a‐[A]** with distinct ^19^F NMR signals at *δ*_F_ −173.5 (d, ^2^ *J*_HF_ = 45.6 Hz) and −152.2 (q, ^3^ *J*_HF_ = 46.5 Hz) in 83% yield and a diastereomeric ratio (*dr*) of 40:60 after 2 h. The reaction was monitored over 6 days to determine the change in yield and *dr*. After 24 h the reaction yield had improved to 98% and the *dr* had improved to 30:70. The *dr* continued to increase to 15:85 after 6 days, with the product still present in 98% (**Figure** **2**).

With the aid of theoretical analysis, we sought to understand how diastereoselectivity arose so we might improve upon our initial results. DFT calculations for the C─F activation event in substrate **1a** suggested an S_N_1 pathway proceeding via a common benzylium intermediate, similar to the calculated activation profile using tetrahydrothiophene (THT) as a base partner (see Figure S143, Supporting Information).^[^ ^12^ ^]^ It was calculated that the addition of sulfide **A** to this intermediate was barrierless, precluding the likelihood that differential kinetic barriers existed capable of giving rise to the observed *dr* in our products.

**Figure 3:**
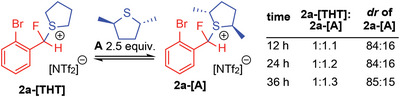
Generation of **2a‐[A]** from **2a‐[THT]** demonstrating facile exchange of sulfide nucleofuges and establishing that stereoselectivity is induced via a thermodynamic equilibrium between diastereomers.

Indeed, a thermodynamic equilibrium is suggested by the improvement in *dr* over time in Figure 2, and evidence for facile exchange of **A** leading to a thermodynamic product distribution was also obtained via reaction of **A** with preformed **2a‐[THT]** (**Figure** **3**). Addition of 2.5 equivalents of **A** to a solution of **2a‐[THT]** resulted in the slow formation of **2a‐[A]** with the ratio of **2a‐[THT]** to **2a‐[A]** being 1:1.3 after 36 h with **2a‐[A]** having a *dr* of 15:85 (see Figure S50, Supporting Information).

**Figure 4:**
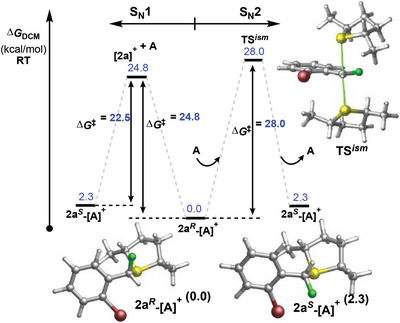
Calculated SN1 and SN2 pathways for isomerization of the C─F chiral center in **2a** from *R* to *S* configuration.

In silico, carbocation **[2a]^+^** was found to remain kinetically accessible at only 22.5–24.8 kcal mol^−1^ above the most stable diastereomer conformers of **2a‐[A]** (**Figure** **4**). An S_N_2 isomerization pathway was located with a higher barrier (as compared to the S_N_1 pathway) of 25.7–28.0 kcal mol^−1^ above **2a‐[A]**, suggesting that *dr* arises from a thermodynamic equilibrium that operates via an S_N_1 isomerization pathway. Indeed, the addition of excessive **A** failed to improve the rate of isomerization between diastereomers but instead promoted the isomerization of **A** from its (*R,R*) isomer into a *meso* form, giving rise to a new enantiomeric product **2a‐[A*_meso_*]**.

With this knowledge in‐hand, we proceeded to optimize the reaction. We found that gentle heating at 40 ˚C and increasing the reaction concentration from 0.12 to 0.60 mm (**Table** **1**, entry 2) generated **2a‐[A]** in 98% with a *dr* of 85:15 after only 24 h (*cf*. 6 days, Figure 2), demonstrating practical access to stereoenriched monofluorides via FLP mediated C─F bond activation. Continued heating for 48 h only improved the *dr* to 86:14 indicating that the equilibrium point of the reaction was likely approached. Under this assumption, a lower experimental estimate of Δ*G* ^0^ = 1.1 kcal mol^−1^ can be derived between the two epimers of **2a‐[A]** (*cf*. Δ*G* ^0^ = 2.3 kcal mol^−1^, computationally derived value).

**Table 1:** Exploration of yield and diastereoselectivity for the stereoselective C─F activation reaction using sulfides **A‐D** with substrates **1a‐b**. Yields based on ^19^F NMR analysis.

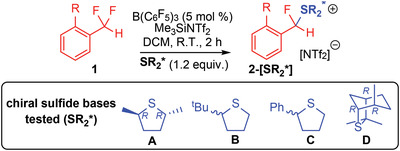						
entry	R group	chiral sulfide	temp	conc. [mm]	time	
					2 h	24 h
**1**	Br (**1a**)	**A**	25 °C	0.12	83% *dr* 60:40	98% *dr 70:30*
**2**	Br (**1a**)	**A**	40 °C	0.60	‐	99% *dr* 85:15
**3**	Br (**1a**)	**B**	25 °C	0.12	70% *dr* 52:48	‐
**4**	Br (**1a**)	**B**	40 °C	0.60	‐	54% *dr* 57:43
**5**	Br (**1a**)	**C**	25 °C	0.12	0%	‐
**6**	Br (**1a**)	**D**	25 °C	0.12	0%	‐
**7**	H (**1b**)	**A**	25 °C	0.12	98% *dr* 52:48	98% *dr* 55:45
**8**	H (**1b**)	**A**	40 °C	0.60	‐	99% *dr* 62:38
**9**	H (**1b**)	**B**	25 °C	0.12	72% *dr* 52:48	‐
**10**	H (**1b**)	**C**	25 °C	0.12	27% *dr* 62:38	0%
**11**	H (**1b**)	**D**	25 °C	0.12	21% *dr* 73:27	0%

Testing the related thiolane **B** ^[^ ^13^ ^]^ with **1a** failed to improve upon the yield or selectivity observed using sulfide **A**, while employing thiolane **C** ^[^ ^14^ ^]^ and sulfide **D** ^[^ ^15^ ^]^ led to no desired products. However, it was found that selective activation of PhCF_2_H (**1b**) with **C** and **D** led to products **2b‐[C]** and **2b‐[D]** in low yield but improved *dr* as compared to that observed in **2b‐[A]**. These products proved much less stable than **2b‐[A]**, precluding the generic use of **C** and **D** for practical syntheses.

**Figure 5:**
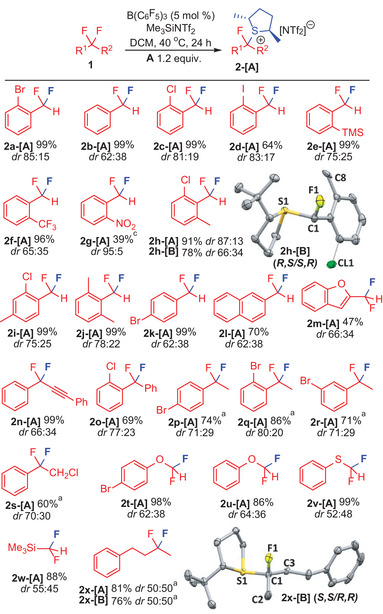
Scope of stereoselective C─F activation reaction. a) [Al(C_6_F_5_)_3_•C_7_H_8_] and PhCl used in place of B(C_6_F_5_)_3_ and DCM. b) Run at r.t. for 2 h. c) 10 mol.% B(C_6_F_5_)_3_ used. See Supporting Information for crystallographic details for structures of **2h‐[B]** and **2x‐[B]**. Yields based on ^19^F NMR analysis.

Overall, it was deemed that Lewis base **A** gave the best combination of high yield and diastereoselectivity. As such, **A** was utilized to explore substrate scope (**Figure** **5**). Compounds **2‐[A]** could be generated in moderate to high yield from difluorides **1**. 1,1‐diflouoromethylaryl substrates that feature substituents in the 2‐position gave very good stereoselectivity, with all examples (except **2f‐[A]**) demonstrating *dr* of greater than 75:25, with **2g‐[A]** having a *dr* of 95:5 (albeit with a reduced yield of 39%).

Internal difluorides (i.e., difluoromethylene) in benzylic positions {**2o‐[A]** – **2s‐[A]**} were also found to give moderate to good stereoselectivity, with **2q‐[A]** generated in 86% yield with a *dr* of 80:20. Difluoromethoxy groups demonstrated moderate stereoselectivity, despite the absence of any *ortho* substituents in products **2t‐[A]** and **2u‐[A]**, while other non‐benzylic supports tested (silyl, sulfide, alkyl) gave poor selectivity. This can be attributed to less stabilized carbocation intermediates raising the kinetic barrier for isomerization. This was exemplified by **2x‐[A]** and **2x‐[B]** that could only be generated in a *dr* of 50:50.

Pleasingly, we were able to isolate compounds **2a‐[A]**, **2c‐[B]**, **2h‐[A]**, **2h‐[B]**, **2i‐[A]**, **2i‐[B]**, **2x‐[A]**, and **2x‐[B]** in 59–88% yield (see Supporting Information). Molecular structures for single diastereomers of **2h‐[B]** and **2x‐[B]** showed that the substitution of enantiotopic fluorides with the chiral sulfide **B** had indeed led to the formation of chiral centers at the C─F position (Figure 5). Further, crystallisation of **2i‐[B]** led to a single diastereomer (see Figure S16, Supporting Information). It was found that the isolated diastereomer slowly isomerized in solution (over hours) to give the original mixture of two diastereomers, suggesting an S_N_1 isomerization process. The isolation of **2i‐[B]** provides proof‐of‐principle for chiral resolution of sulfonium salts 2‐[SR_2_*].

**Figure 6:**
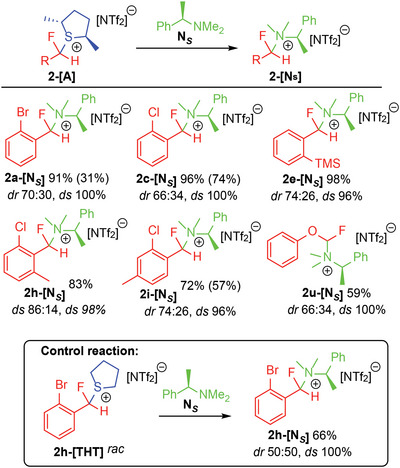
Transfer of fluorocarbon fragment to enantiopure chiral amine **N*_S_*** with high stereospecificity. Yields based on ^19^F NMR analysis. Isolated yields in brackets. See Supporting Information for reaction conditions.

Given that **A** is an enantiopure base, products **2‐[A]** represent epimers and optically enriched products can be generated with the stereospecific transfer of the chiral fluorocarbon fragment. This was demonstrated by the reaction of enantiopure amine (*S*)‐*N,N‐*dimethyl‐1‐phenylethylamine (**N*_S_***) with **2‐[A]** to generate epimeric products **2‐[N*_S_*]** in high diastereospecificity (Figure 6). For example, addition of **N*_S_*** to a solution of **2a‐[A]** resulted in the formation of **2a‐[N*_S_*]** in 91% yield with a diastereospecificity (*ds*) of 100%. Similarly, *ds* for products **2c‐[N*_S_*]**, **2e‐[N*_S_*]**, **2h‐[N*_S_*]**, **2i‐[N*_S_*]**, and **2u‐[N*_S_*]** were all over 96%. A control reaction of **N*_S_*** with *racemic* **2a‐[THT]** resulted in **2a‐[N*_S_*]** with a *dr* of 50:50, precluding diastereoselective induction through exchange of **N*_S_*** (additionally, the barrier for exchange of **N*_S_*** was found to be kinetically inaccessible, see Pages S37–S39, Supporting Information). (**Figure** **6**)

**Figure 7:**
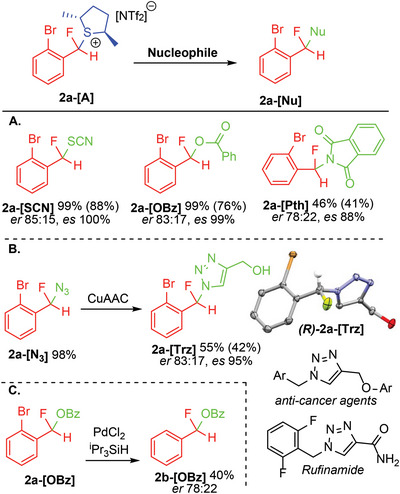
A) Generation of enantioenriched products via stereospecific reaction of **2a‐[A]** with achiral nucleophiles. B) Access to an enantioenriched fluoro‐analog of benzyltriazole motifs present in current and potential pharmaceuticals.^[^ ^12^ ^]^ Absolute configuration determination revealed the product **2a‐[Trz]** to be the *R*‐enantiomer. C) Hydrodebromination of **2a‐[OBz]** to give **2b‐[OBz]**. See SI for reaction conditions.

High enantiospecificity (*es*) of the S_N_2 transfer also allowed for the generation of enantioenriched products (**Figure** **7**). For example, the reaction of **2a‐[A]** with [N^n^Bu_4_][SCN] resulted in **2a‐[SCN]** in 88% isolated yield with an enantiomeric ratio (*er*) of 85:15 (*ee* 70%) and *es* of 100%. Similarly, **2a‐[OBz]** was generated in 76% isolated yield with *er* of 83:17 and **2a‐[Pth]** was generated in 41% isolated yield with *er* of 78:22. Importantly, enantioenriched benzylfluorides in the absence of α‐carbonyls are difficult to generate using existing synthetic technologies.^[^ ^3^, ^5^ ^]^

Further functionalization possibilities were also demonstrated by installing an azide group to generate **2a‐[N_3_]**, which could then be employed in a copper catalyzed azide‐alkyne cycloaddition (CuAAC) to generate **2a‐[Trz]** in 55% yield with *er* of 83:17 (Figure 7B). Absolute configuration of **2a‐[Trz]** revealed the product to be the *R*‐enantiomer, in agreement with an inversion of the (calculated) thermodynamically preferred epimer **2a*^R^*‐[A]** (Figure 4). **2a‐[Trz]** represents an enantioenriched fluoro‐analog of benzyltriazole motifs that are present in current and potential pharmaceuticals.^[^ ^16^ ^]^

Lastly, the superior stereoselectivity in 1,1‐difluoromethylarene substrates with *ortho* substituents presents an opportunity for further functionalization. For example, the removal of silyl and halo groups is prevalent in the literature (i.e. hydrodesilylation and hydrodehalogenation) and allows for traceless enhanced stereoselectivity.^[^ ^17^ ^]^ The veracity of such an approach was demonstrated through the hydrodebromination of **2a‐[OBz]** in 40% generating **2b‐[OBz]** with an *er* or 78:22 (Figure 7C).

## 3 Conclusion

In summary, we have shown that chiral Lewis bases can be utilized to induce stereoselectivity in the FLP selective C─F activation of *geminal* difluorides (**1**). The sulfonium products, 2‐[SR_2_*], exhibit moderate to good diastereoselectivity (up to *dr* 95:5 for **2g‐[A]**) that arises from facile isomerization of 2‐[SR_2_*] via a calculated S_N_1 pathway. As such, substrates that prevent the facile exchange of sulfide and substrates that do not sterically impede the bound sulfide (e.g., benzyl groups without *ortho* substituents) give limited stereoselectivity. The use of enantiopure chiral Lewis bases allows for the transfer of the chiral fluorocarbon fragment with high stereospecificity leading to diastereo and enantioenriched products that are difficult to generate using current stereoselective methodologies.

## Conflict of Interest

The authors declare no conflict of interest.

## Supporting information

Supporting Information

## Data Availability

The data that support the findings of this study are available from the corresponding author upon reasonable request.
