# Supplementary material for: Stereoselective Synthesis of Fluoroalkanes via FLP Mediated Monoselective C─F Activation of Geminal Difluoroalkanes
Source: Adv Sci (Weinh). 2023 Oct 31;10(36):2305768. doi: 10.1002/advs.202305768 (PMC10754124; doi:10.1002/advs.202305768)
Supplement: Supplementary file 1 — Supporting Information [file ADVS-10-2305768-s001.pdf]

ADVANCED SCIENCE
Open Access

Supporting Information

for *Adv. Sci.*, DOI 10.1002/advs.202305768

Stereoselective Synthesis of Fluoroalkanes via FLP Mediated Monoselective C—F Activation of Geminal Difluoroalkanes

*Dániel Csókás, Bivas Mondal, Miloš Đokić, Richa Gupta, Beatrice J. Y. Lee and Rowan D. Young**

<u>**Electronic Supporting Information**</u>

Stereoselective synthesis of fluoroalkanes via FLP mediated monoselective C–F activation of *geminal* difluoroalkanes

Dániel Csókás[a,b], Bivas Mondal[a], Miloš Đokić[a], Richa Gupta[a], Beatrice J. Y. Lee[a], and Rowan D. Young[a,c]*

[a] Department of Chemistry, National University of Singapore, Singapore 117543
[b] Research Centre for Natural Sciences, Institute of Organic Chemistry, Budapest, Hungary 1117
[c] School of Chemistry and Molecular Biosciences, The University of Queensland, Australia 4072

*Corresponding Author: rowan.young@uq.edu.au

Table of Contents

Experimental **2**
General information 2
General procedure for synthesis of salts 2-[SR_2] (reaction scope) 3
^{19}F NMR spectra and selected *in situ* data used for Figure 3 4
Isolation and characterisation of compounds 2-[SR_2*] 21
Synthesis and characterisation of stereoenriched salts 2-[N_S] (chiral resolution) 25
NMR spectra for Figure 5 28
Synthesis and characterization of enantioenriched neutral compounds 2a-[Nu] (*er* determined by chiral HPLC) 34
Evidence that *dr* in products 2-[N_S] does not arise from amine exchange 37
Monitoring *dr* of 2a-[A] after completion of C–F activation reaction 40
Reaction of 2a-[THT] with A 41
Attempts to isomerize free A to A_{meso} 41
Crystallographic details 42
Characterisation data **43**
NMR spectra for isolated compounds 43
Chiral HPLC traces for compounds 2a-[Nu] 76
Mass Spectra 81
DFT Studies **87**
Computational methodology 87
Computational results 88
Computed energy components of the reported structures. 95
Cartesian coordinates of the reported structures 96
References **116**

Experimental

General information

Experiments were carried under inert conditions using standard Schlenk techniques or a glove box (Vacuum Atmospheres Company) as appropriate, although subsequent manipulations of air and moisture stable ammonium salts were performed under ambient conditions. Dichloromethane (DCM), *n*-hexane, and toluene were dispensed from an LC Technology Solution Inc. SP-1 Solvent Purification System, deoxygenated (N_2 bubbled for 20 min), and stored over 4 Å molecular sieves prior to use. 1,2-Dichlorobenzene (1,2-DCB), chloroform-d and dichloromethane-d_2 solvents were stirred over CaH_2 at room temperature under N_2 overnight prior to distillation under reduced pressure and stored over 4 Å molecular sieves before use. Vials and stirrer bars used for reactions were oven-dried overnight before experiments. ^{1}H, ^{19}F, $^{19}F\{^{1}H\}$ and $^{13}C\{^{1}H\}$ NMR spectra were recorded at 298 K on Bruker AV-400 or AV-500 spectrometers. The chemical shifts (δ, ppm) for ^{1}H and $^{13}C\{^{1}H\}$ NMR spectra are given relative to solvent signals whereas an external reference standard was used for ^{19}F ($CFCl_3$) NMR spectra referencing. NMR data are reported as: chemical shift, multiplicity (s = singlet, d = doublet, t = triplet, q = quartet, m = multiplet, br = broad), coupling constants (Hz) and integration. HRMS spectra were recorded on an Agilent Technologies 6230 TOF MS (ESI-TOF). The reagents $B(C_6F_5)_3$[1] (BCF), $[Al(C_6F_5]_3{\bullet}C_7H_8]$[2] (ACF), (2*R,5R*)-2,5-dimethylthiolane (**A**)[3], 2-*tert*-butylthiolane (**B**)[4] and 2-phenylthiolane (**C**)[5] were prepared by following literature method, and purified via column chromatography (hexane eluent). Sulfide **A** was also subject to trap-to-trap distillation under reduced pressure to ensure removal of residual alkali metals. All other reagents, including (*1R,4R,5R*)-4,7,7-Trimethyl-6-thiabicyclo[3.2.1]octane/Isothiocineole (**D**), were purchased commercially and used as received.

General procedure for synthesis of salts 2-[SR$_2$] (reaction scope)

To a mixture of BCF (1.6 mg, 0.003 mmol, 0.05 equiv.), sulfide **A** (0.07 mmol, 1.2 equiv.), TMSNTf$_2$ (25.6 mg, 0.07 mmol, 1.2 equiv.) and PhOCF$_3$ (9.7 mg, 0.06 mmol, 1 equiv., IS) in DCM (0.1 mL) in a J. Young's NMR tube was added the difluoride substrate (0.06 mmol, 1 equiv.). The reaction was heated at 40 ˚C for 24 hours before ^{19}F NMR analysis was performed to determine NMR yield and selectivity.

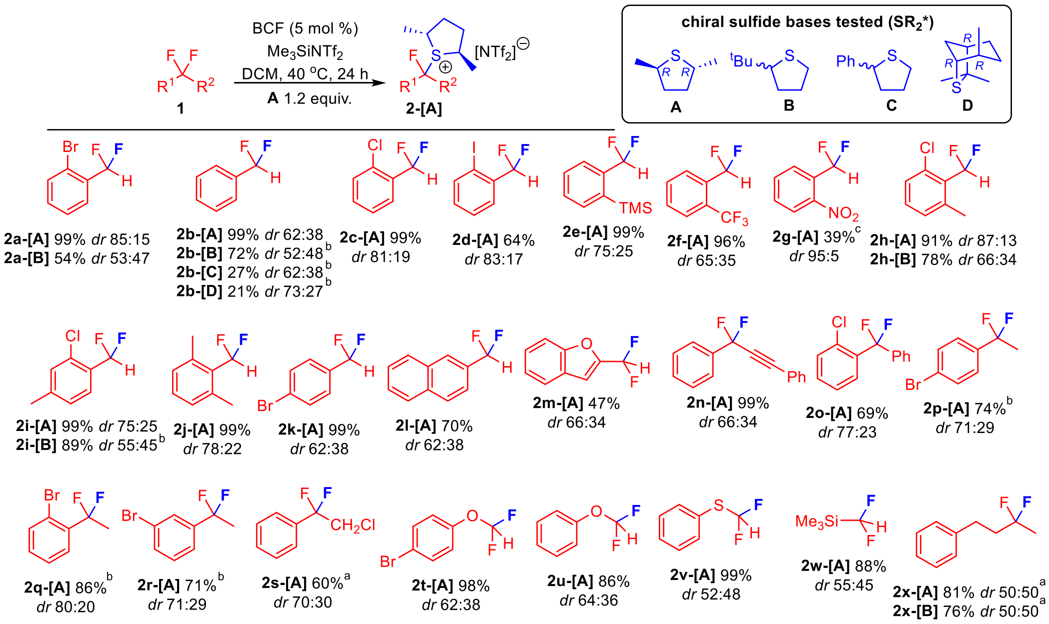

[a] ACF catalyst and 1,2-DCB solvent used. [b] Reaction carried out at room temperature for 2 hours. [c] 10 mol % BCF used.

^{19}F NMR spectra and selected *in situ* data used for Figure 3

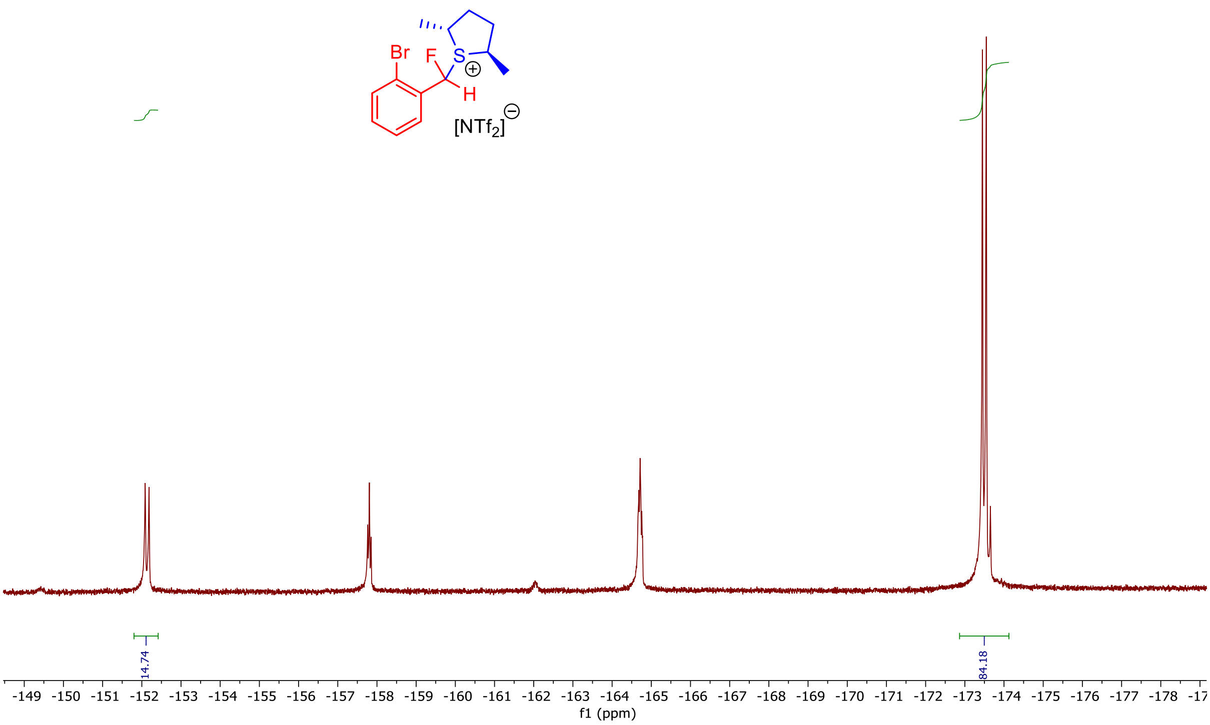

Figure S1. ^{19}F NMR spectrum of crude reaction for **2a-[A]** in DCM-d$_2$. NMR Yield: = 99%; *dr* = 85:15. Major isomer: ^{19}F NMR (376 MHz, CD_2Cl_2): δ_F -173.5 (d, $^2J_{FH}$ = 45.6 Hz, 1 F). Minor isomer: ^{19}F NMR (376 MHz, CD_2Cl_2): δ_F -152.2 (d, $^2J_{FH}$ = 46.5 Hz, 1 F).

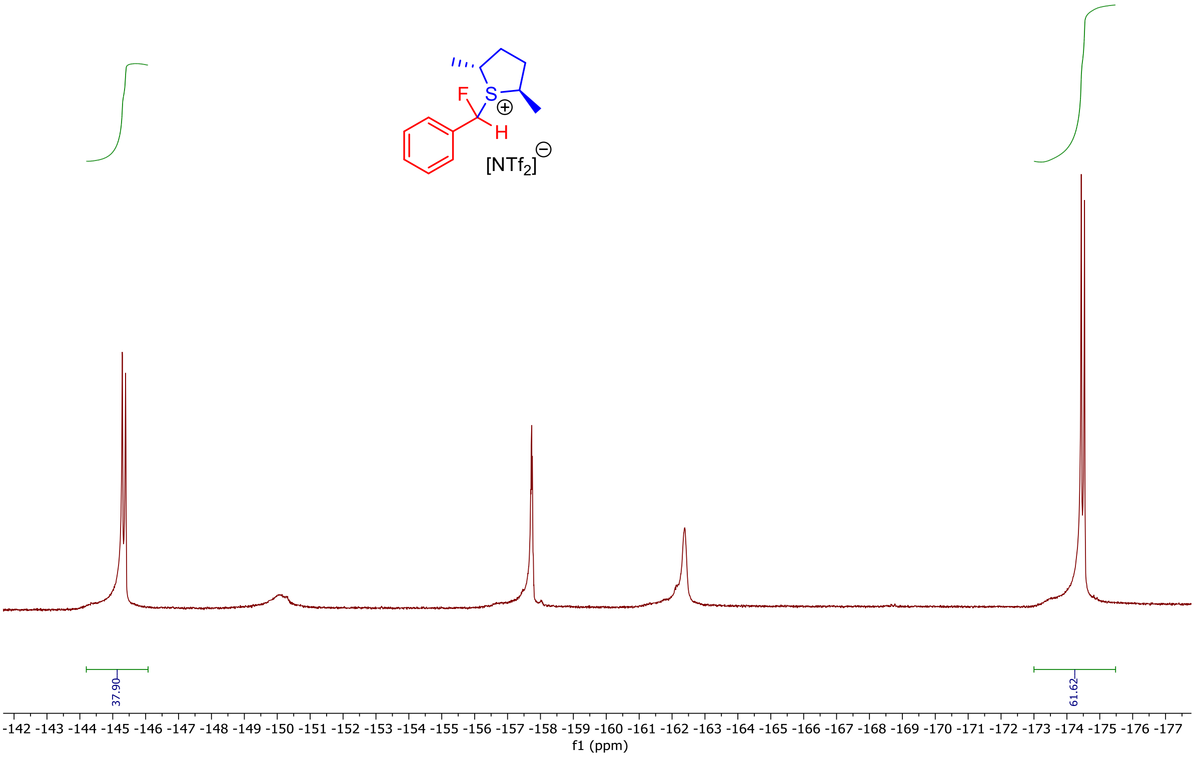

Figure S2. ^{19}F NMR spectrum of crude reaction for **2b-[A]** in DCM-d$_2$. NMR Yield: = 99%; *dr* = 62:38. Major isomer: ^{19}F NMR (376 MHz, CD_2Cl_2): δ_F -174.7 (d, $^2J_{FH}$ = 47.1 Hz, 1 F). Minor isomer: ^{19}F NMR (376 MHz, CD_2Cl_2): δ_F -145.4 (d, $^2J_{FH}$ = 47.4 Hz, 1 F).

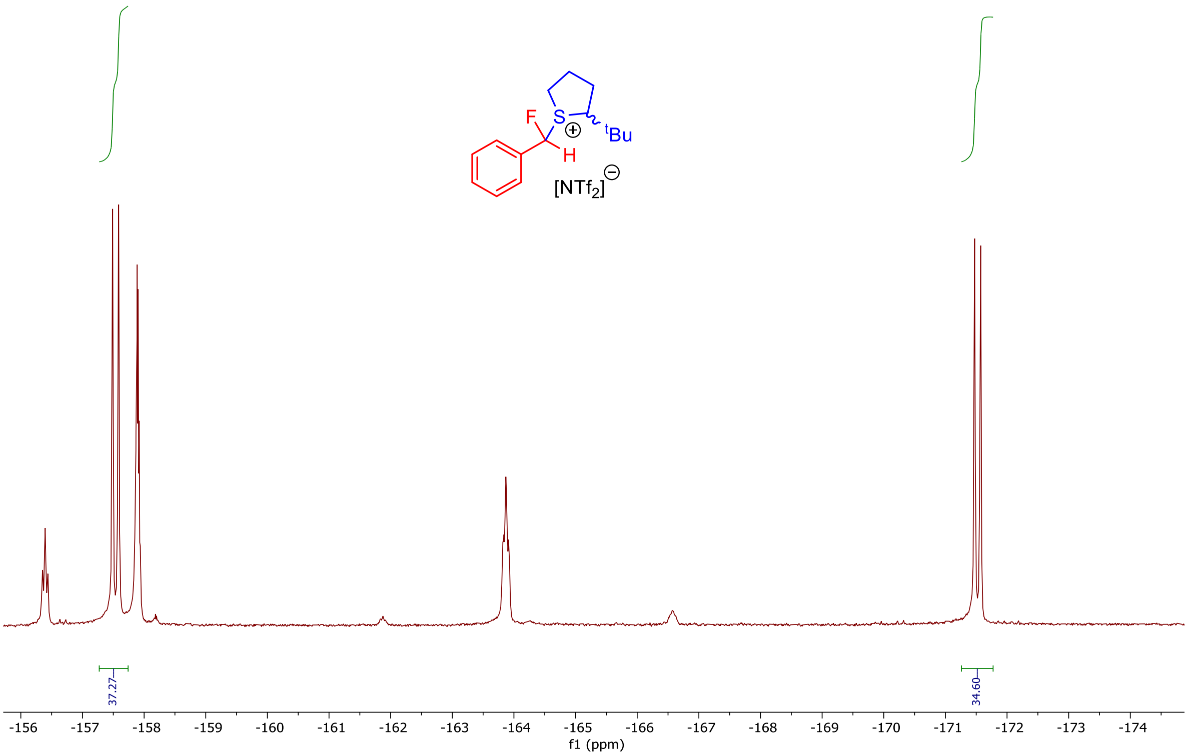

Figure S3. ^{19}F NMR spectrum of crude reaction for **2b-[B]** in DCM-d$_2$. NMR Yield: = 72%; *dr* = 52:48. Major isomer: ^{19}F NMR (376 MHz, CD_2Cl_2): δ_F -157.5 (d, $^2J_{FH}$ = 46.2 Hz, 1 F). Minor isomer: ^{19}F NMR (376 MHz, CD_2Cl_2): δ_F -171.5 (d, $^2J_{FH}$ = 45.8 Hz, 1 F).

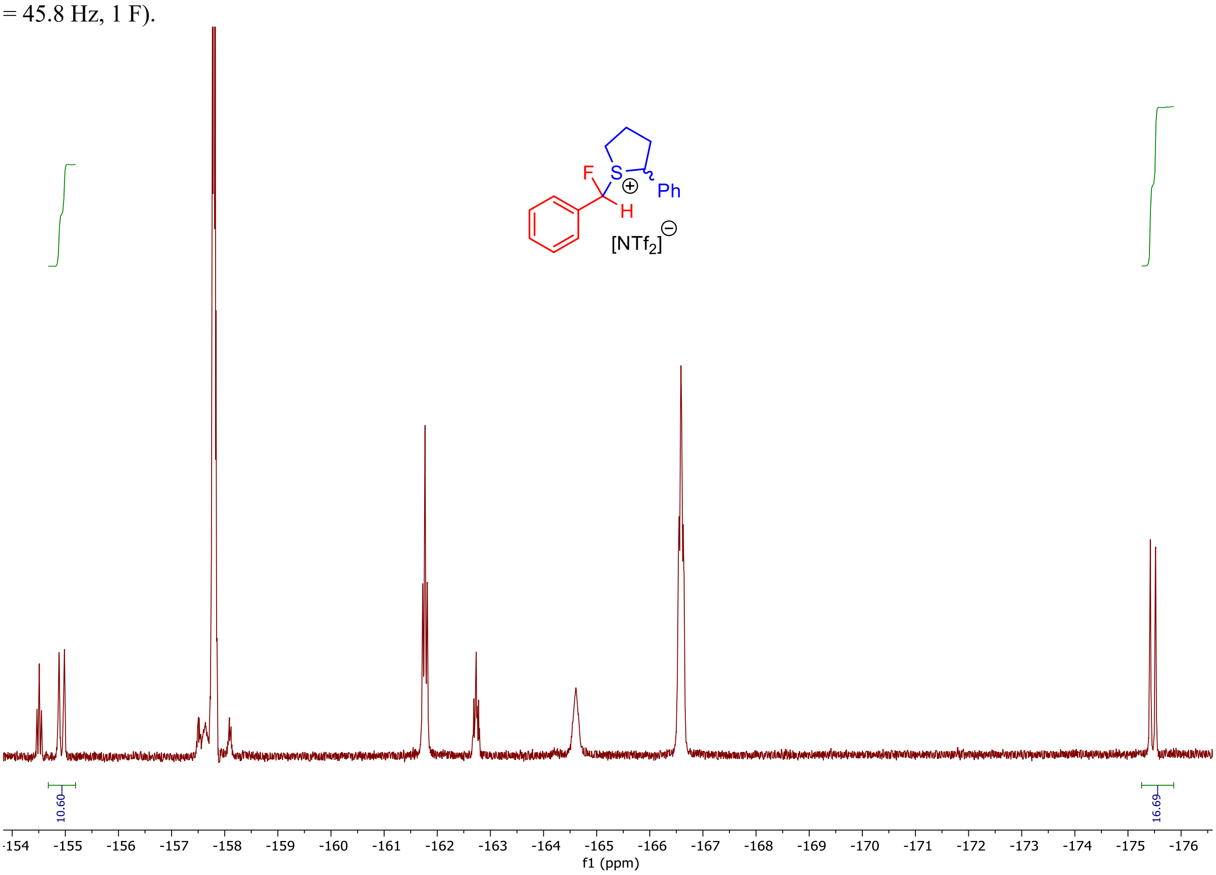

Figure S4. ^{19}F NMR spectrum of crude reaction for **2b-[C]** in DCM-d$_2$. NMR Yield: = 27%; *dr* = 62:38. Major isomer: ^{19}F NMR (376 MHz, CD_2Cl_2): δ_F -175.5 (d, $^2J_{FH}$ = 47.0 Hz, 1 F). Minor isomer: ^{19}F NMR (376 MHz, CD_2Cl_2): δ_F -154.9 (d, $^2J_{FH}$ = 46.8 Hz, 1 F).

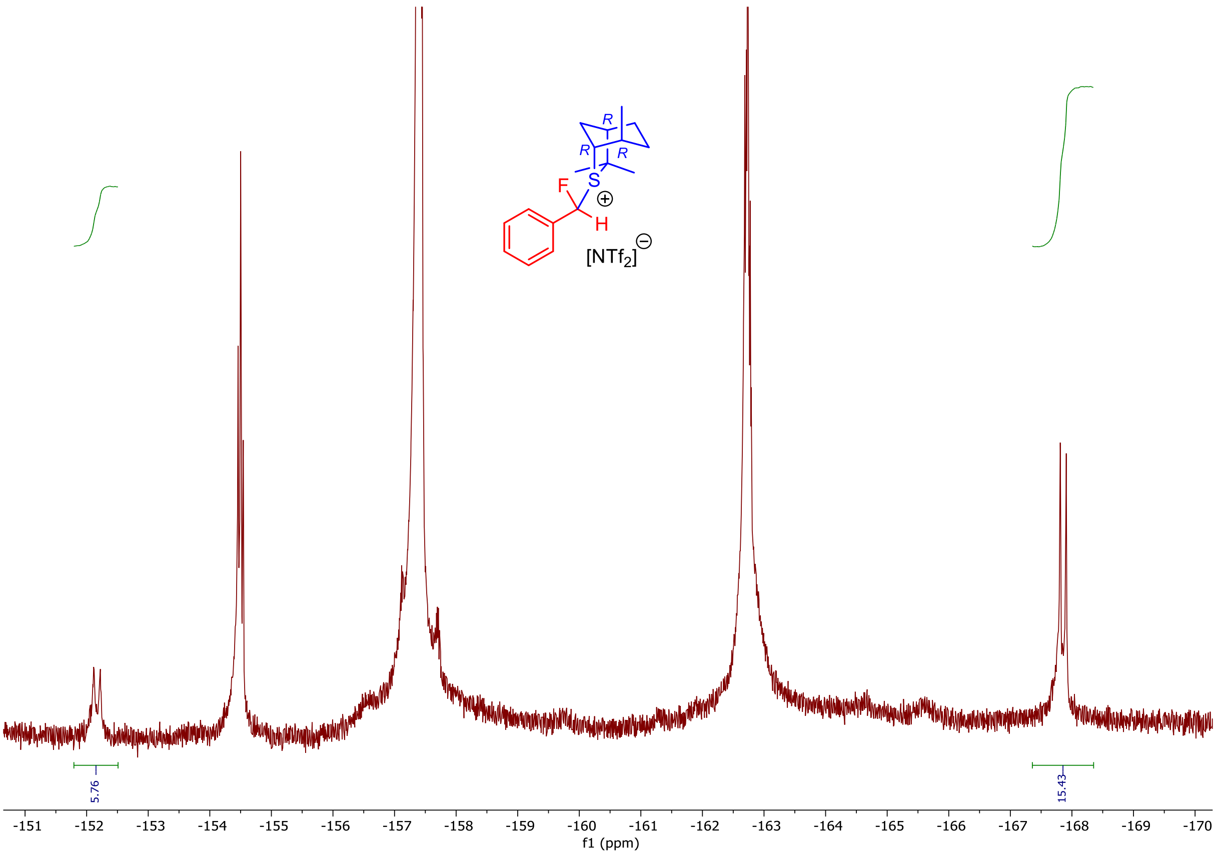

Figure S5. ^{19}F NMR spectrum of crude reaction for **2b-[D]** in DCM-d$_2$ after 2 hours. NMR Yield: = 21%; *dr* = 73:27. Major isomer: ^{19}F NMR (376 MHz, CD_2Cl_2): δ_F -167.8 (d, $^2J_{FH}$ = 46.1 Hz, 1 F). Minor isomer: ^{19}F NMR (376 MHz, CD_2Cl_2): δ_F -152.2 (d, $^2J_{FH}$ = 47.4 Hz, 1 F).

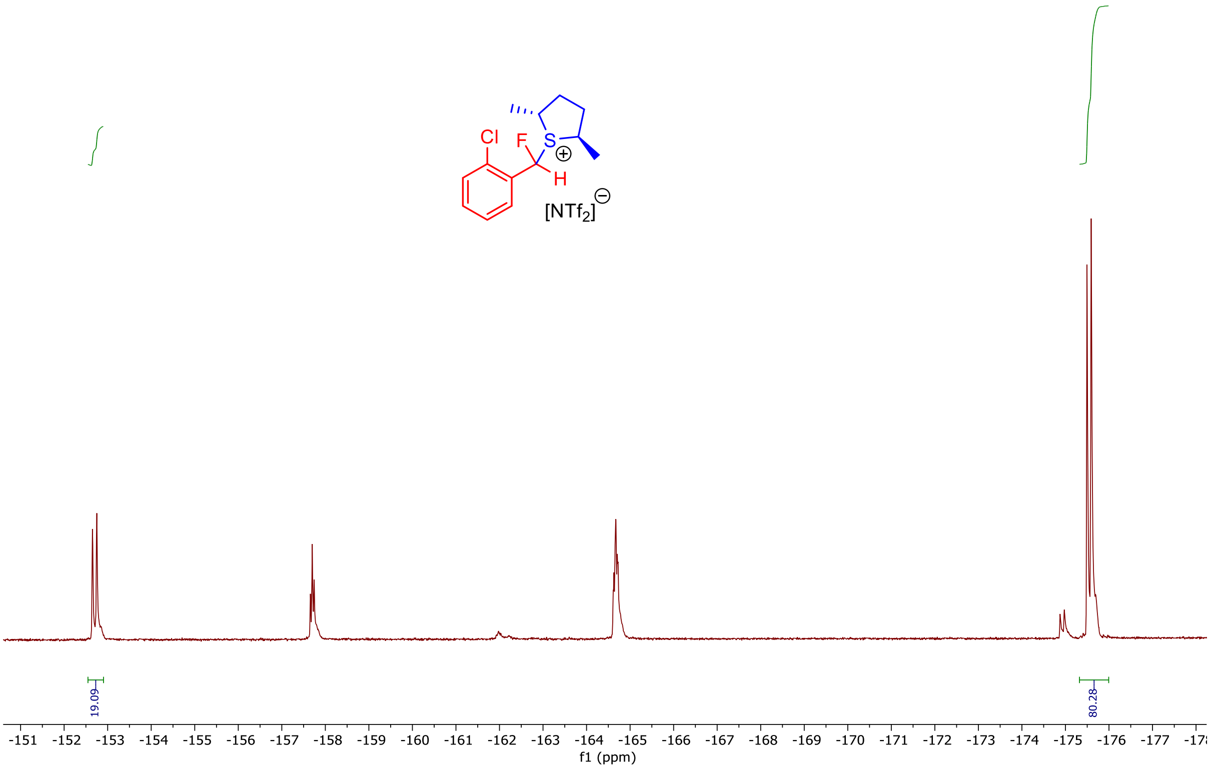

Figure S6. ^{19}F NMR spectrum of crude reaction for **2c-[A]** in DCM-d$_2$. NMR Yield: = 99%; *dr* = 81:19. Major isomer: ^{19}F NMR (376 MHz, CD_2Cl_2): δ_F -175.6 (d, $^2J_{FH}$ = 45.8 Hz, 1 F). Minor isomer: ^{19}F NMR (376 MHz, CD_2Cl_2): δ_F -152.8 (d, $^2J_{FH}$ = 46.7 Hz, 1 F).

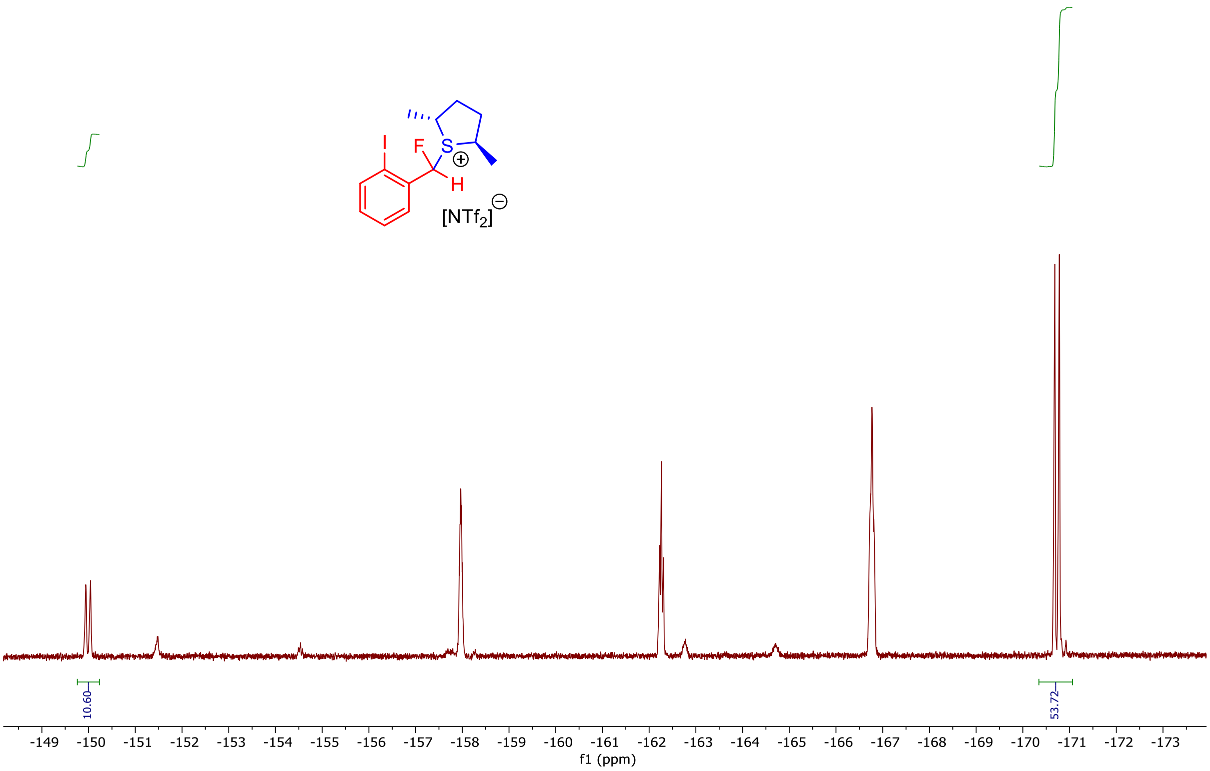

Figure S7. ^{19}F NMR spectrum of crude reaction for **2d-[A]** in DCM-d$_2$. NMR Yield: = 64%; *dr* = 83:17. Major isomer: ^{19}F NMR (376 MHz, CD_2Cl_2): δ_F -170.7 (d, $^2J_{FH}$ = 46.0 Hz, 1 F). Minor isomer: ^{19}F NMR (376 MHz, CD_2Cl_2): δ_F -150.0 (d, $^2J_{FH}$ = 47.3 Hz, 1 F).

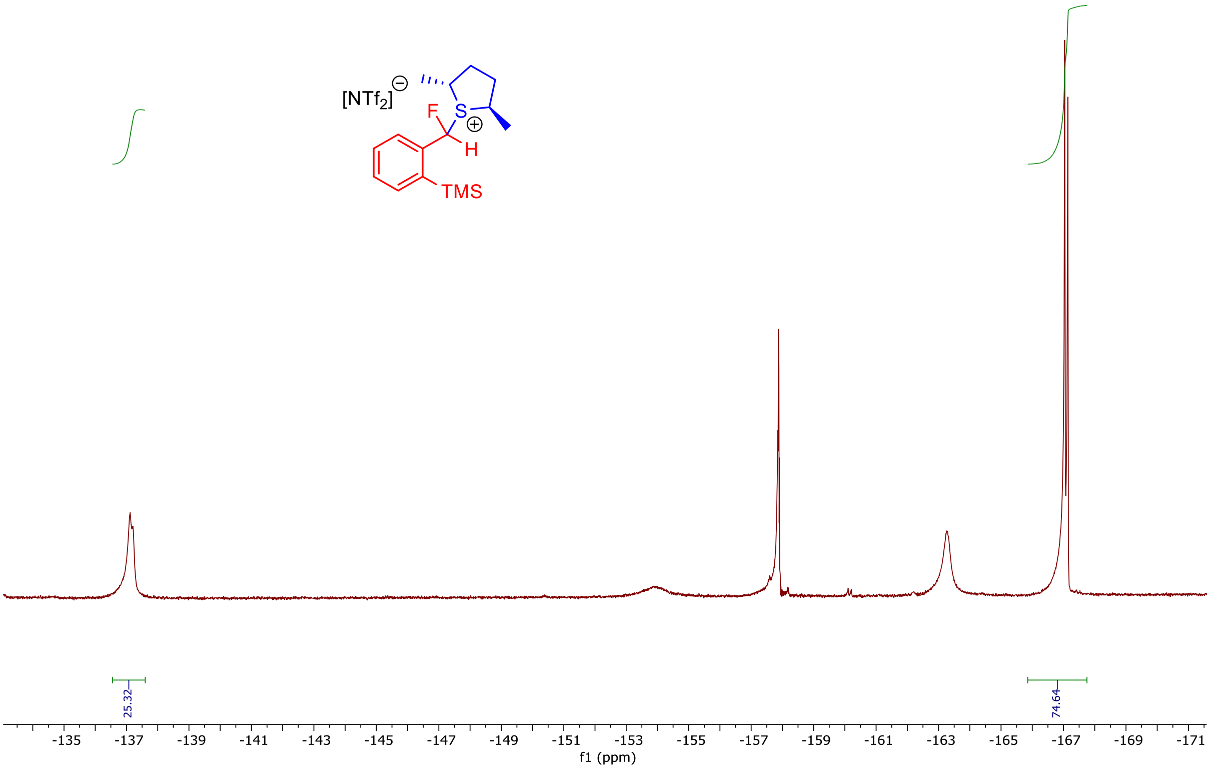

Figure S8. ^{19}F NMR spectrum of crude reaction for **2e-[A]** in DCM-d$_2$. NMR Yield: = 99%; *dr* = 75:25. Major isomer: ^{19}F NMR (376 MHz, CD_2Cl_2): δ_F -167.1 (d, $^2J_{FH}$ = 47.2 Hz, 1 F). Minor isomer: ^{19}F NMR (376 MHz, CD_2Cl_2): δ_F -137.1 (d, $^2J_{FH}$ = 46.3 Hz, 1 F). **HRMS** (ESI-TOF) m/z: 297.1507 for $[C_{16}H_{26}FSSi]^+$ (calcd.: 297.1503).

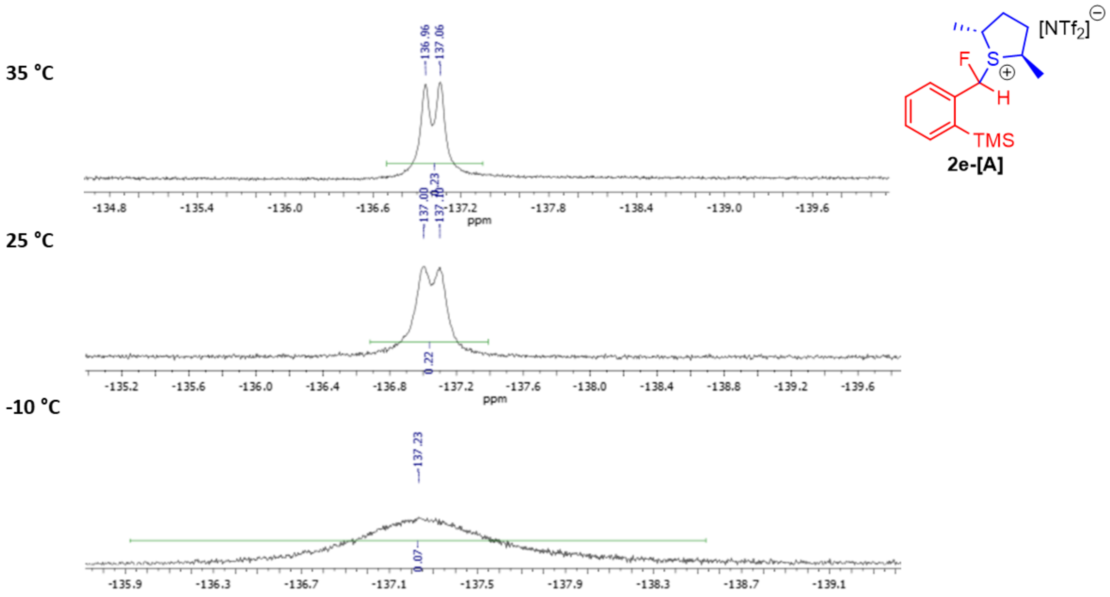

Figure S9. VT-^{19}F NMR spectrum for **2e-[A]** in DCM-d_2 showing resolution of coupling for signal at δ_F -137.1 at elevated temperatures.

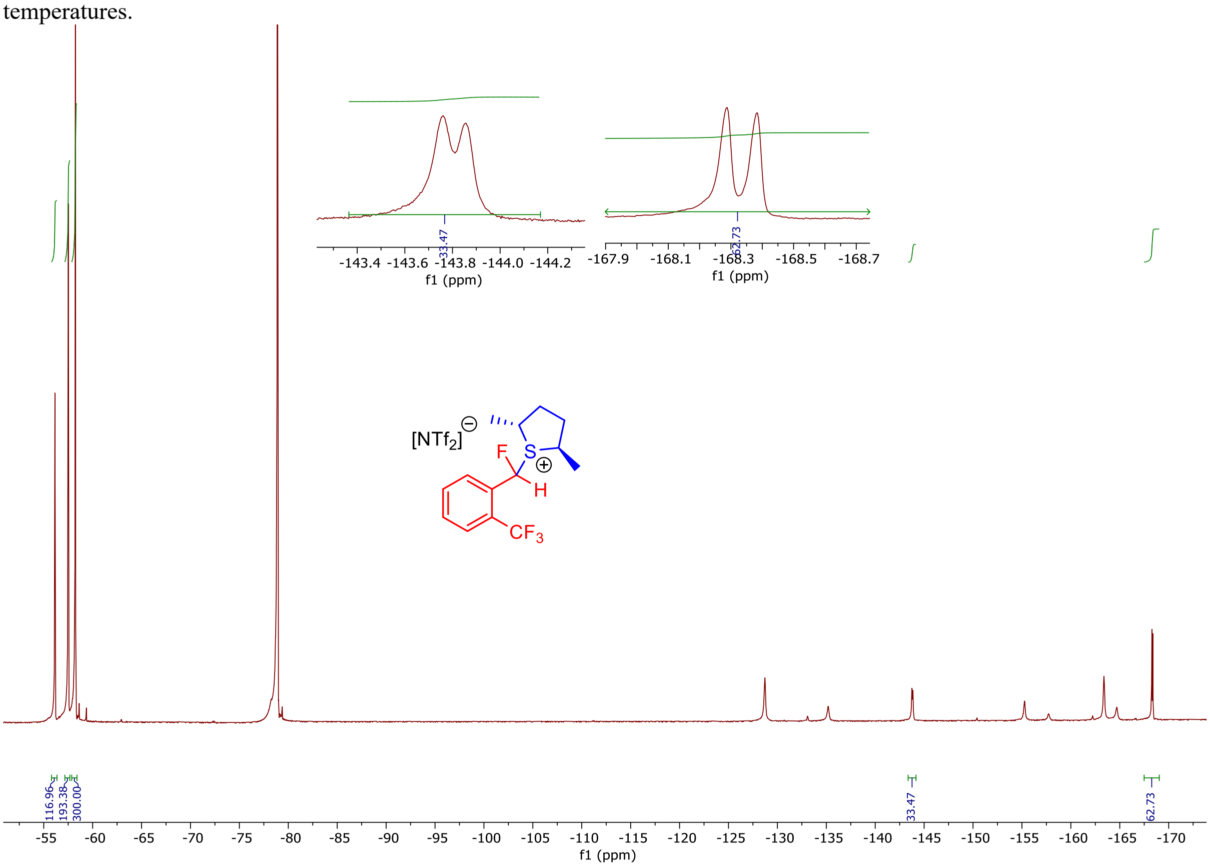

Figure S10. ^{19}F NMR spectrum of crude reaction for **2f-[A]** in DCM-d_2. NMR Yield: = 96%; *dr* = 65:35. Major isomer: ^{19}F NMR (376 MHz, CD_2Cl_2): δ_F -168.3 (d, $^2J_{FH}$ = 46.5 Hz, 1 F), -57.5 (s, 3 F). Minor isomer: ^{19}F NMR (376 MHz, CD_2Cl_2): δ_F -143.8 (d, $^2J_{FH}$ = 45.1 Hz, 1 F), -56.2 (s, 3 F).

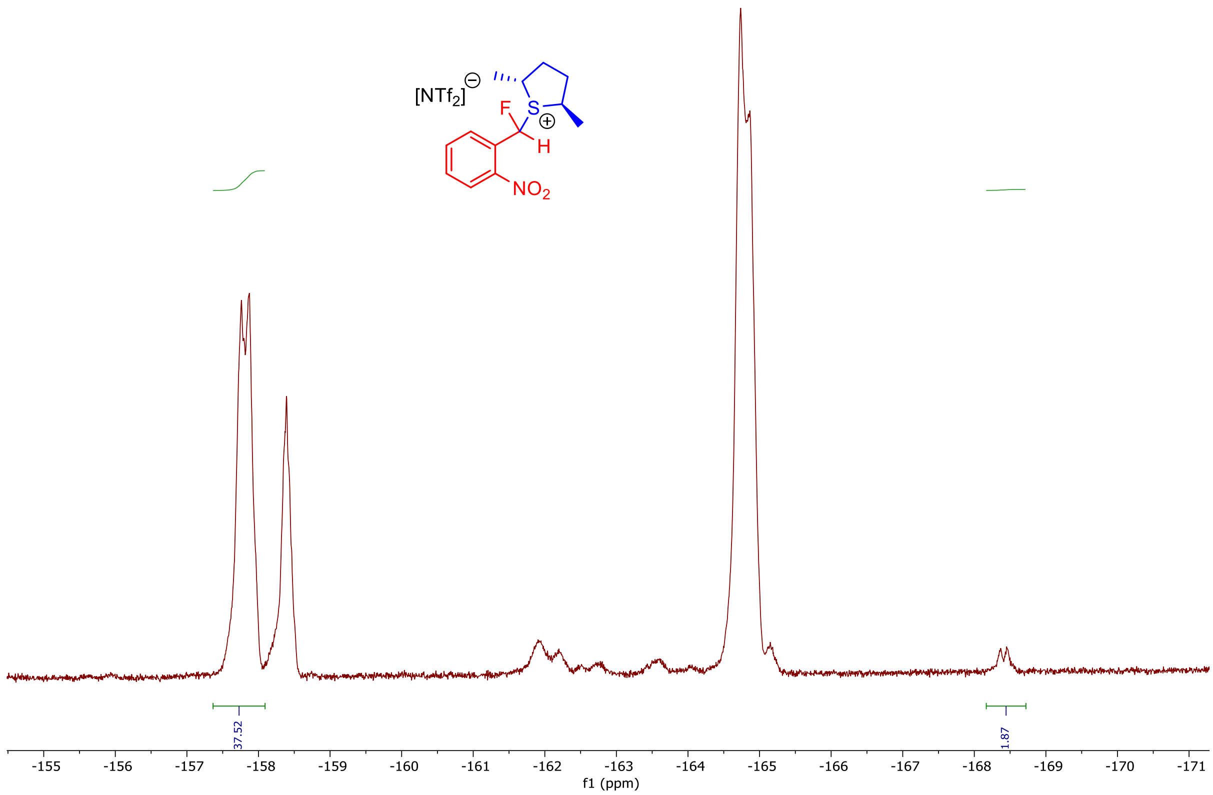

Figure S11. ^{19}F NMR spectrum of crude reaction for **2g-[A]** in DCM-d2. NMR Yield: = 39%; *dr* = 95:5. Major isomer: ^{19}F NMR (376 MHz, CD_2Cl_2): δ_F -157.8 (d, $^2J_{FH}$ = 47.9 Hz, 1 F). Minor isomer: ^{19}F NMR (376 MHz, CD_2Cl_2): δ_F -168.4 (d, $^2J_{FH}$ = 44.1 Hz, 1 F). **HRMS** (ESI-TOF) m/z: 270.0961 for $[C_{13}H_{17}FNO_2S]^+$ (calcd.: 270.0959).

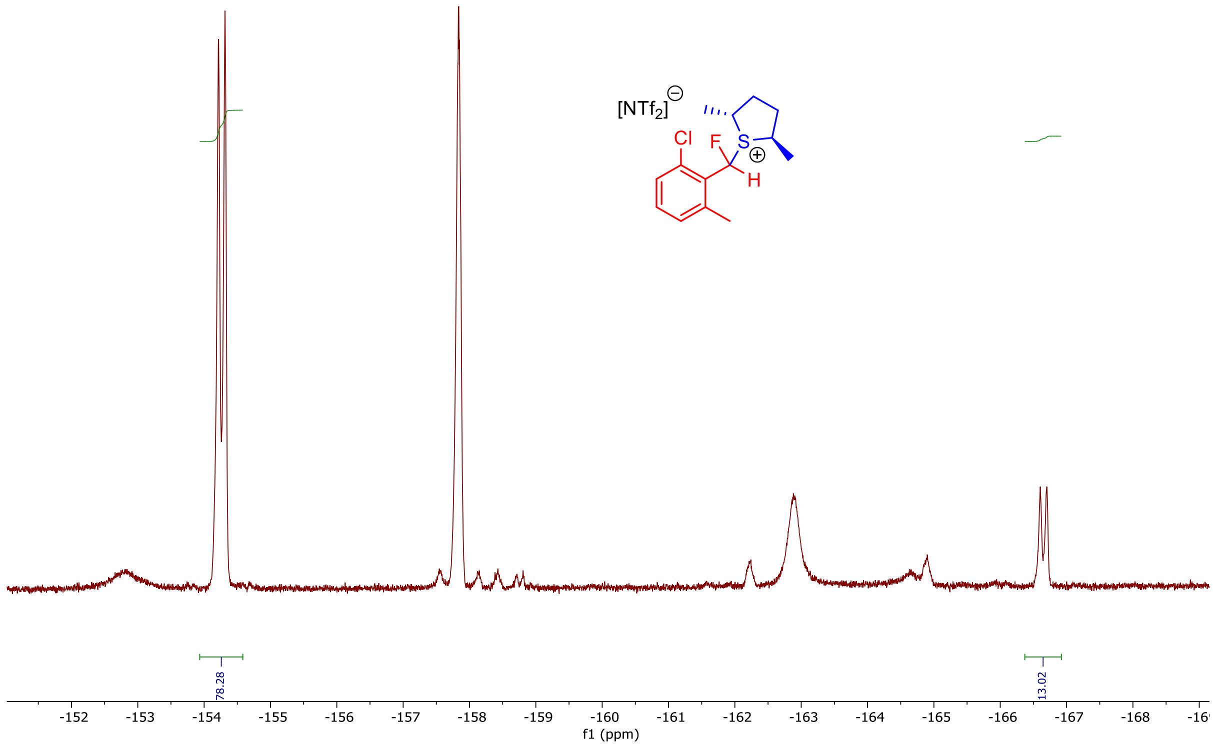

Figure S12. ^{19}F NMR spectrum of crude reaction for **2h-[A]** in DCM-d2 after 2 hours. NMR Yield: = 91%; *dr* = 87:13. Major isomer: ^{19}F NMR (376 MHz, CD_2Cl_2): δ_F -154.3 (d, $^2J_{FH}$ = 47.2 Hz, 1 F). Minor isomer: ^{19}F NMR (376 MHz, CD_2Cl_2): δ_F -166.6 (d, $^2J_{FH}$ = 46.0 Hz, 1 F).

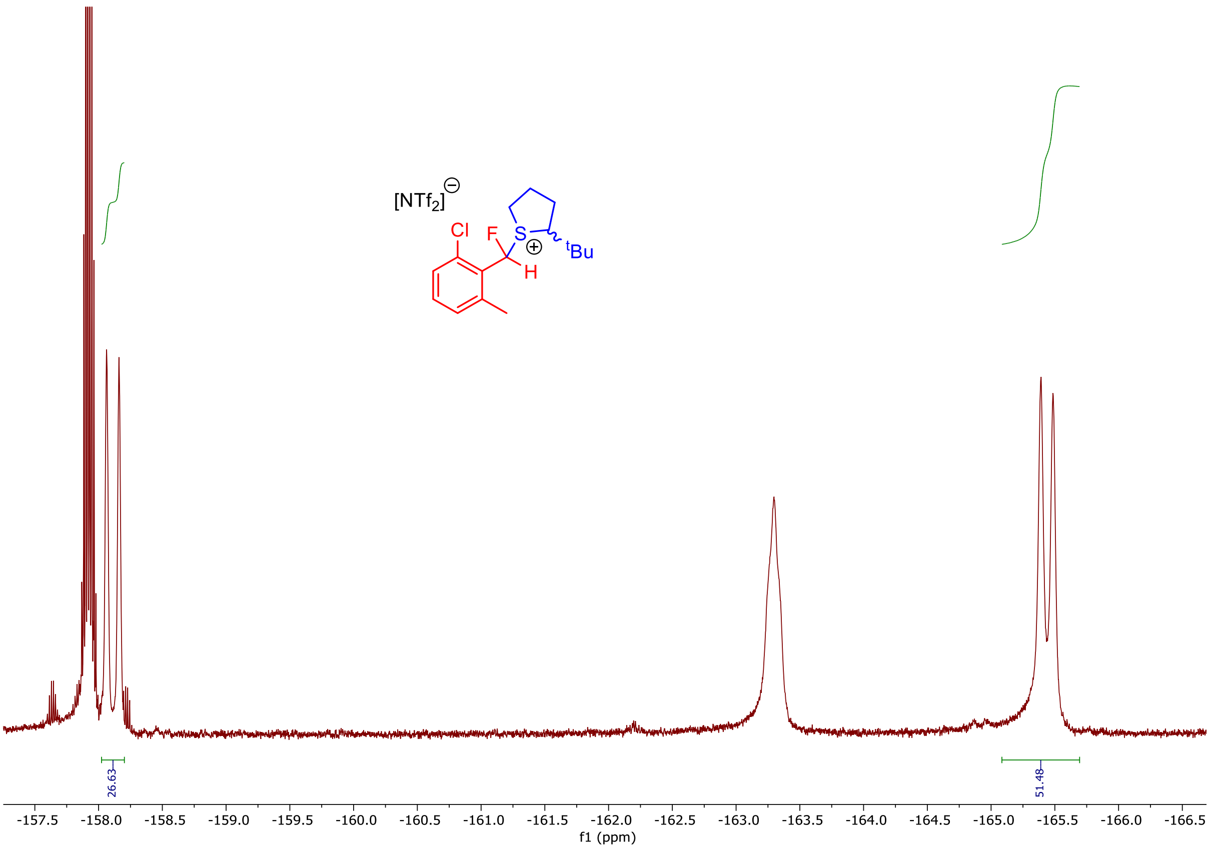

Figure S13. ^{19}F NMR spectrum of crude reaction for **2h-[B]** in DCM-d$_2$. NMR Yield: = 78%; *dr* = 66:34. Major isomer: ^{19}F NMR (376 MHz, CD_2Cl_2): δ_F -165.4 (d, $^2J_{FH}$ = 44.6 Hz, 1 F). Minor isomer: ^{19}F NMR (376 MHz, CD_2Cl_2): δ_F -158.1 (d, $^2J_{FH}$ = 45.7 Hz, 1 F).

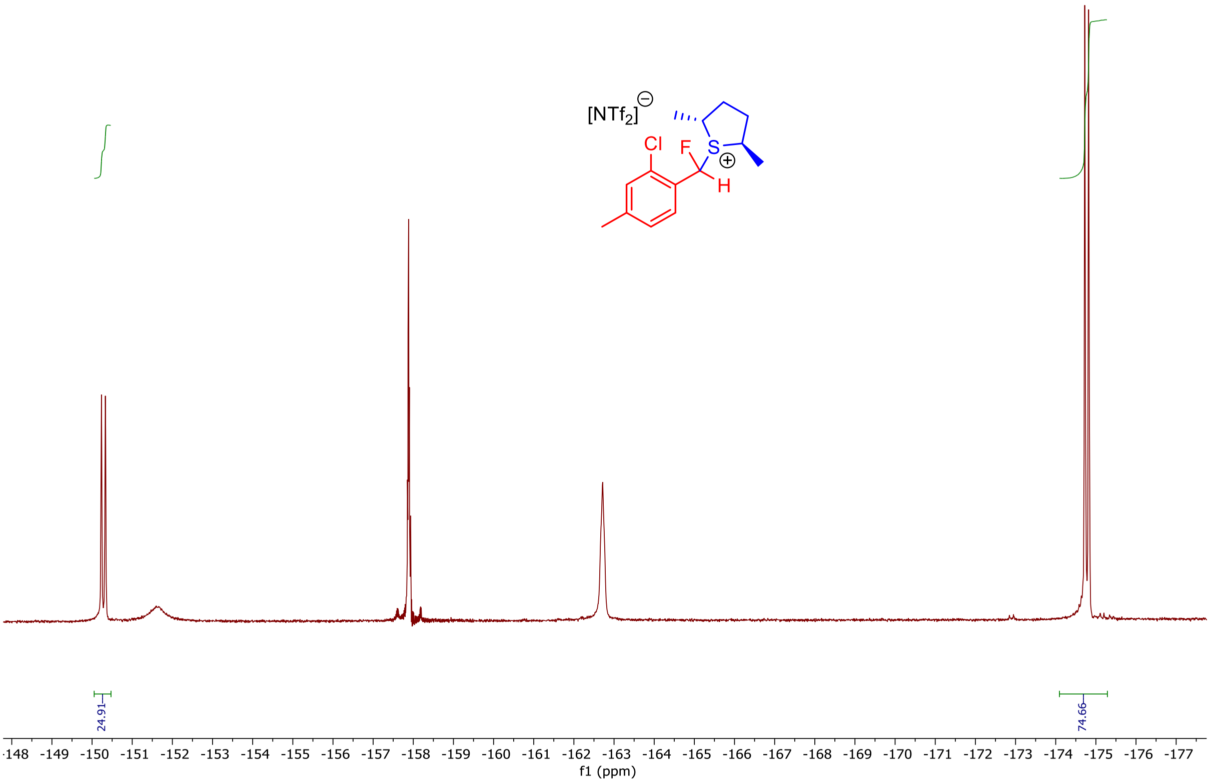

Figure S14. ^{19}F NMR spectrum of crude reaction for **2i-[A]** in DCM-d$_2$. NMR Yield: = 99%; *dr* = 75:25. Major isomer: ^{19}F NMR (376 MHz, CD_2Cl_2): δ_F -174.8 (d, $^2J_{FH}$ = 45.6 Hz, 1 F). Minor isomer: ^{19}F NMR (376 MHz, CD_2Cl_2): δ_F -150.2 (d, $^2J_{FH}$ = 45.6 Hz, 1 F).

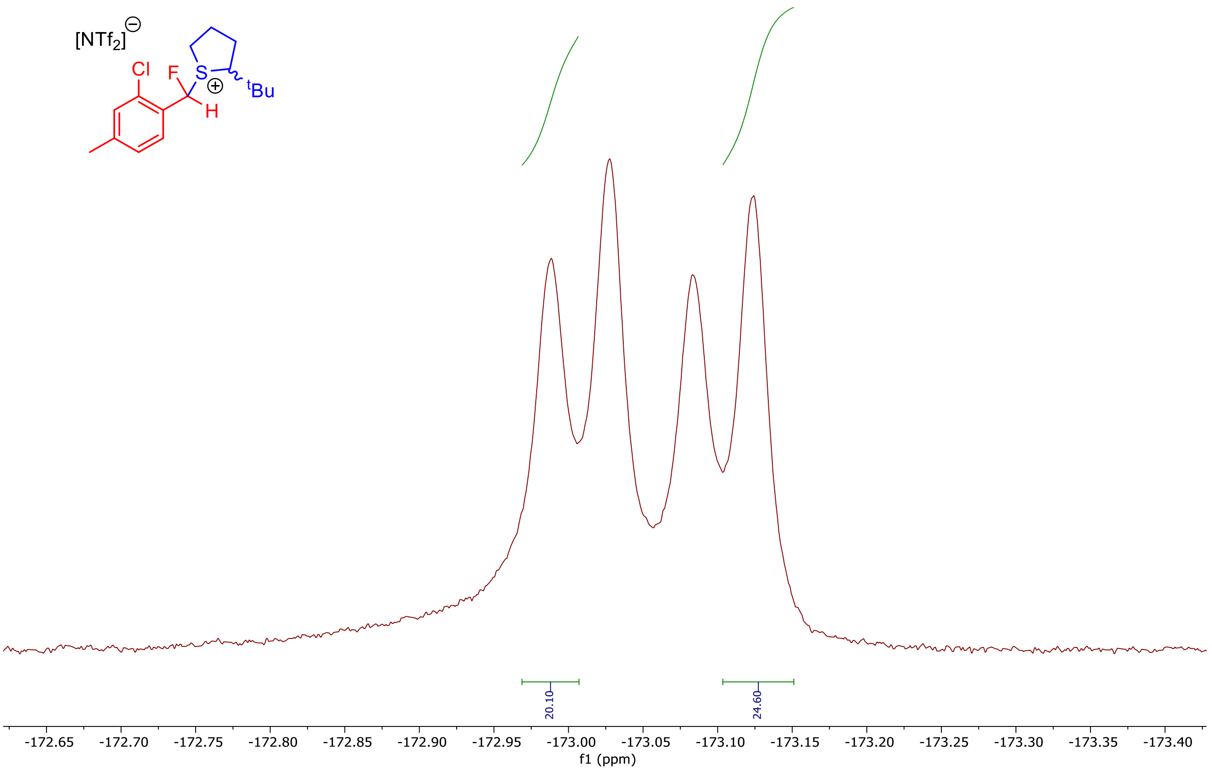

Figure S15. ^{19}F NMR spectrum of crude reaction for **2i-[B]** in DCM-d$_2$. NMR Yield: = 89%; *dr* = 55:45. Major isomer: ^{19}F NMR (376 MHz, CD_2Cl_2): δ_F -173.1 (d, $^2J_{FH}$ = 45.6 Hz, 1 F). Minor isomer: ^{19}F NMR (376 MHz, CD_2Cl_2): δ_F -173.0 (d, $^2J_{FH}$ = 44.3 Hz, 1 F).

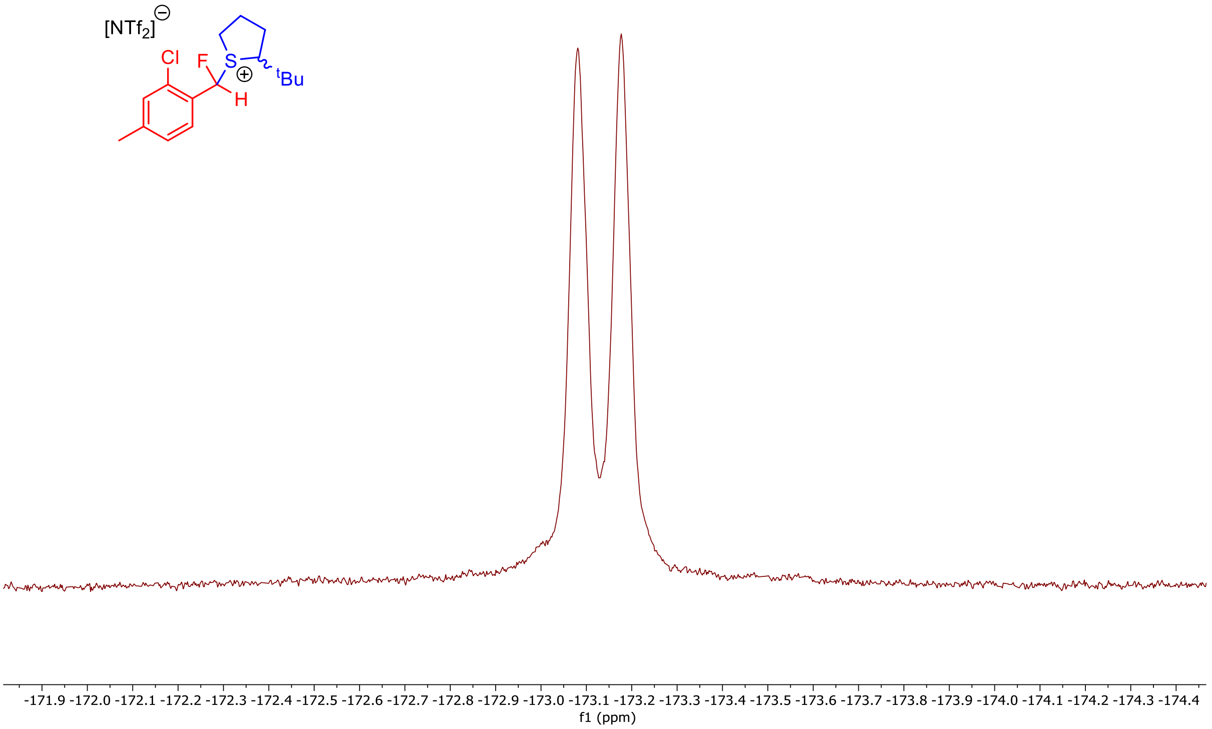

Figure S16. ^{19}F NMR spectrum of recrystallised sample of **2i-[B]** in DCM-d$_2$. Only major isomer observed 5 min after dissolution.

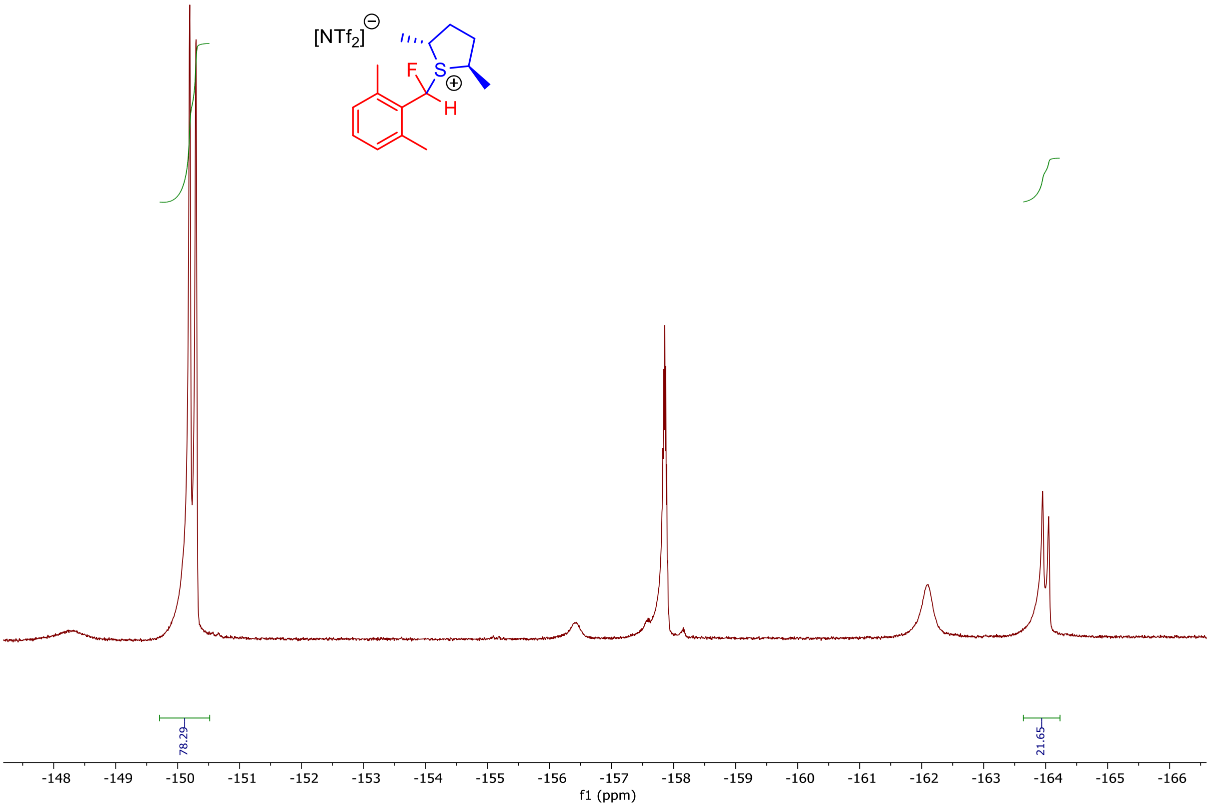

Figure S17. ^{19}F NMR spectrum of crude reaction for **2j-[A]** in DCM-d2. NMR Yield: = 99%; *dr* = 78:22. Major isomer: ^{19}F NMR (376 MHz, CD_2Cl_2): δ_F -150.3 (d, $^2J_{FH}$ = 46.8 Hz, 1 F). Minor isomer: ^{19}F NMR (376 MHz, CD_2Cl_2): δ_F -164.0 (d, $^2J_{FH}$ = 46.4 Hz, 1 F).

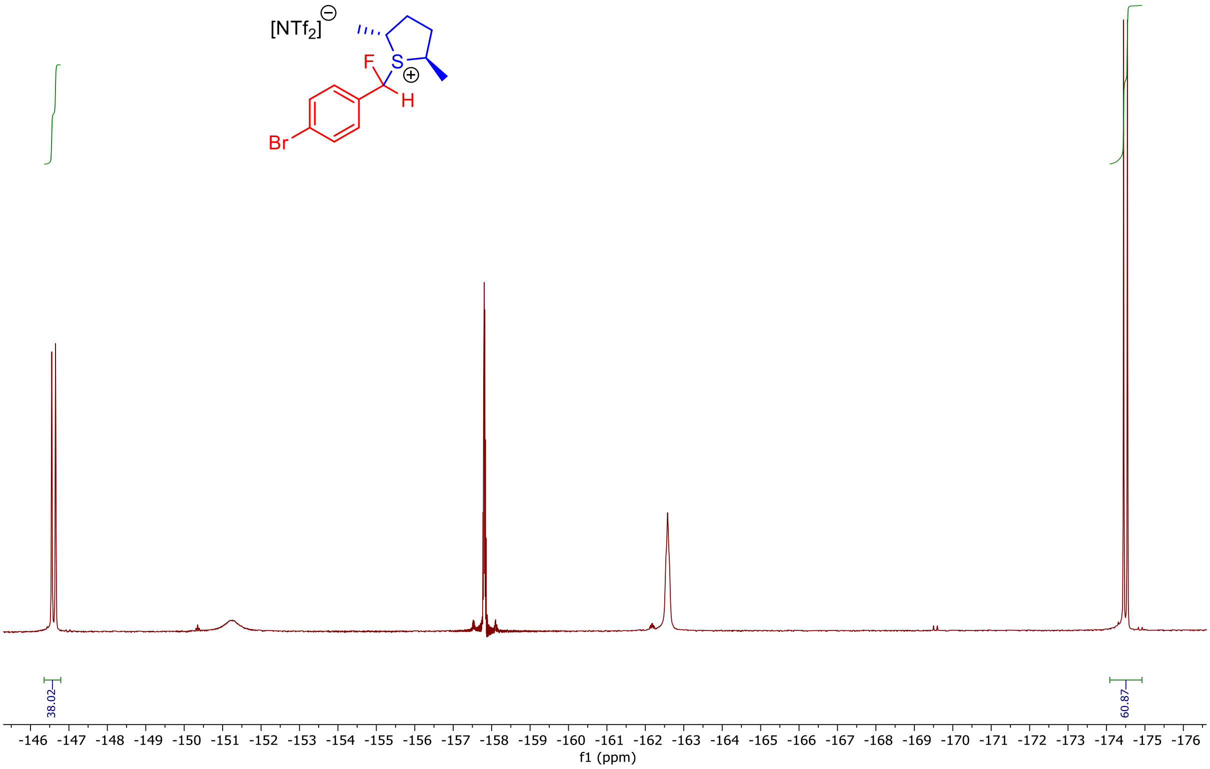

Figure S18. ^{19}F NMR spectrum of crude reaction for **2k-[A]** in DCM-d2. NMR Yield: = 99%; *dr* = 62:38. Major isomer: ^{19}F NMR (376 MHz, CD_2Cl_2): δ_F -146.6 (d, $^2J_{FH}$ = 46.2 Hz, 1 F). Minor isomer: ^{19}F NMR (376 MHz, CD_2Cl_2): δ_F -174.7 (d, $^2J_{FH}$ = 46.7 Hz, 1 F).

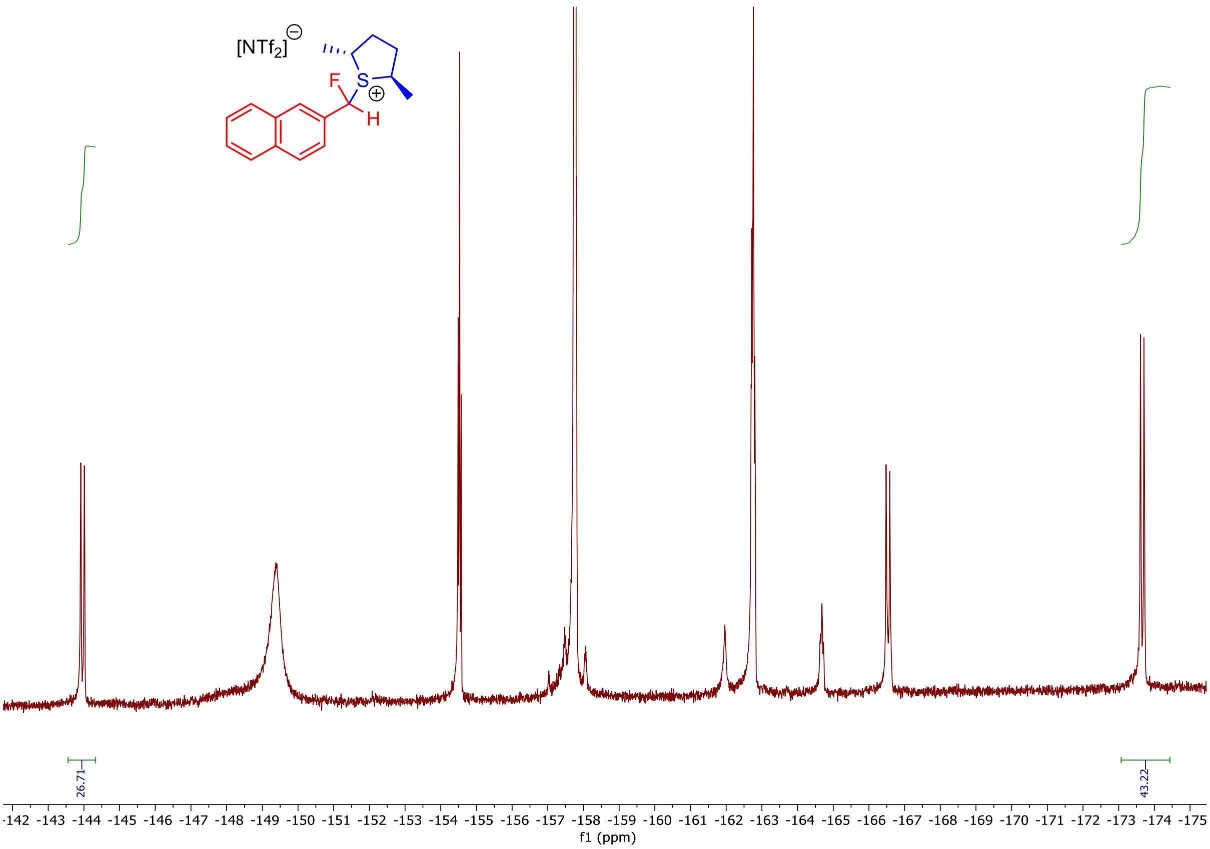

Figure S19. ^{19}F NMR spectrum of crude reaction for **2l-[A]** in DCM-d$_2$. NMR Yield: = 70%; *dr* = 62:38. Major isomer: ^{19}F NMR (376 MHz, CD_2Cl_2): δ_F -173.6 (d, $^2J_{FH}$ = 46.5 Hz, 1 F). Minor isomer: ^{19}F NMR (376 MHz, CD_2Cl_2): δ_F -144.1 (d, $^2J_{FH}$ = 47.1 Hz, 1 F).

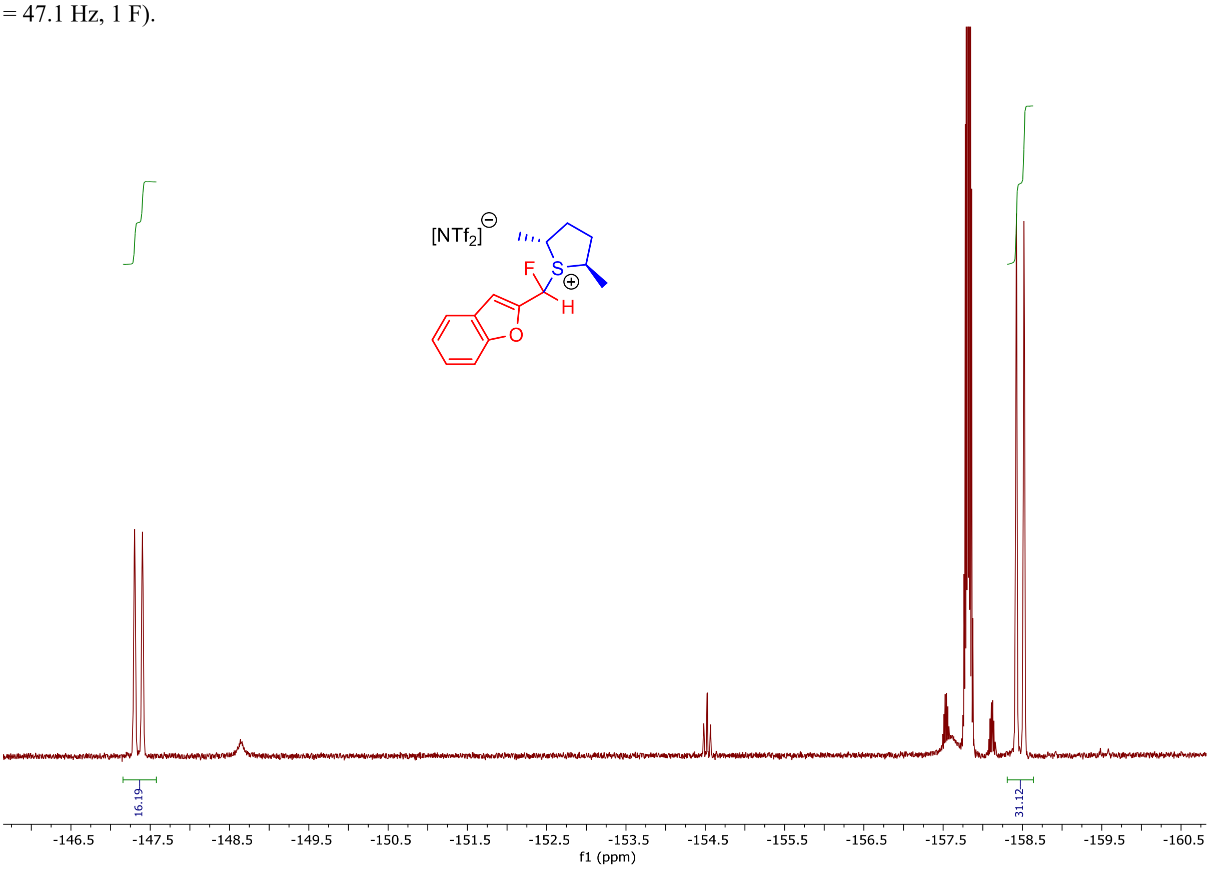

Figure S20. ^{19}F NMR spectrum of crude reaction for **2m-[A]** in DCM-d$_2$. NMR Yield: = 47%; *dr* = 66:34. Major isomer: ^{19}F NMR (376 MHz, CD_2Cl_2): δ_F -158.5 (d, $^2J_{FH}$ = 45.6 Hz, 1 F). Minor isomer: ^{19}F NMR (376 MHz, CD_2Cl_2): δ_F -147.4 (d, $^2J_{FH}$ = 46.5 Hz, 1 F).

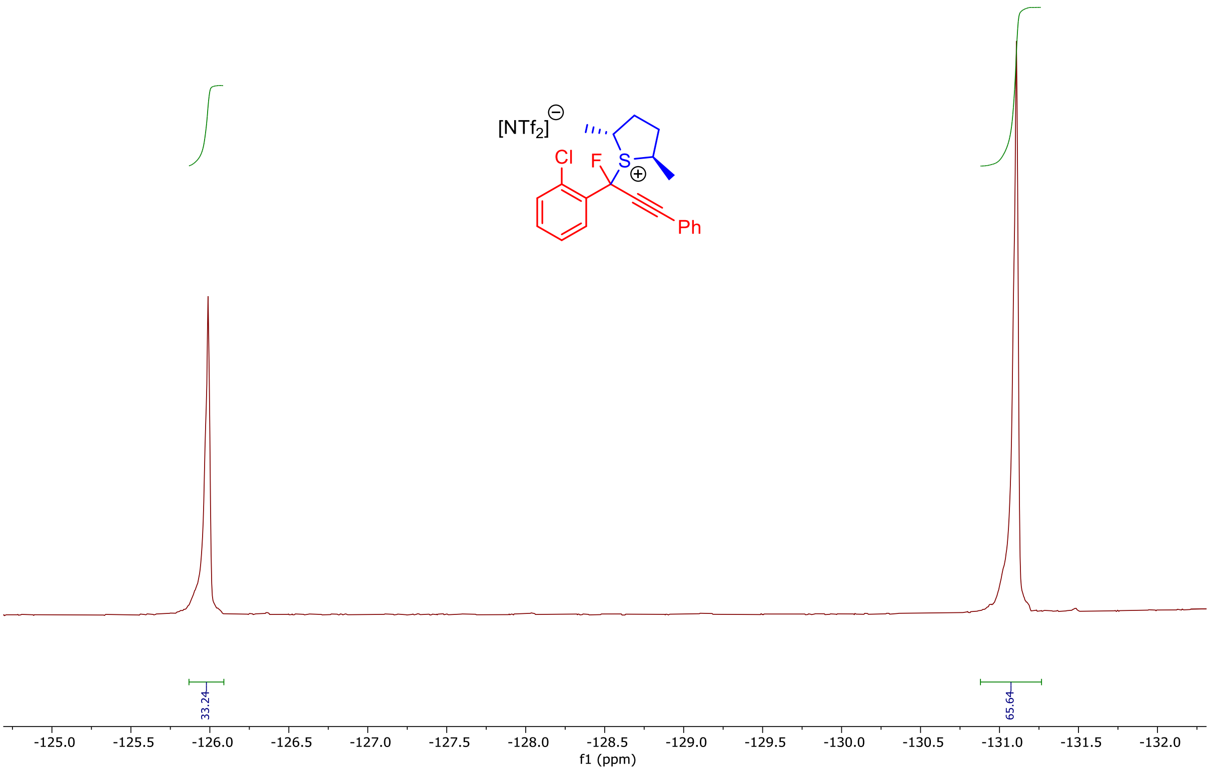

Figure S21. ^{19}F NMR spectrum of crude reaction for **2n-[A]** in DCM-d2. NMR Yield: = 99%; *dr* = 66:34. Major isomer: ^{19}F NMR (376 MHz, CD_2Cl_2): δ_F -131.1 (s, 1 F). Minor isomer: ^{19}F NMR (376 MHz, CD_2Cl_2): δ_F -126.0 (s, 1 F).

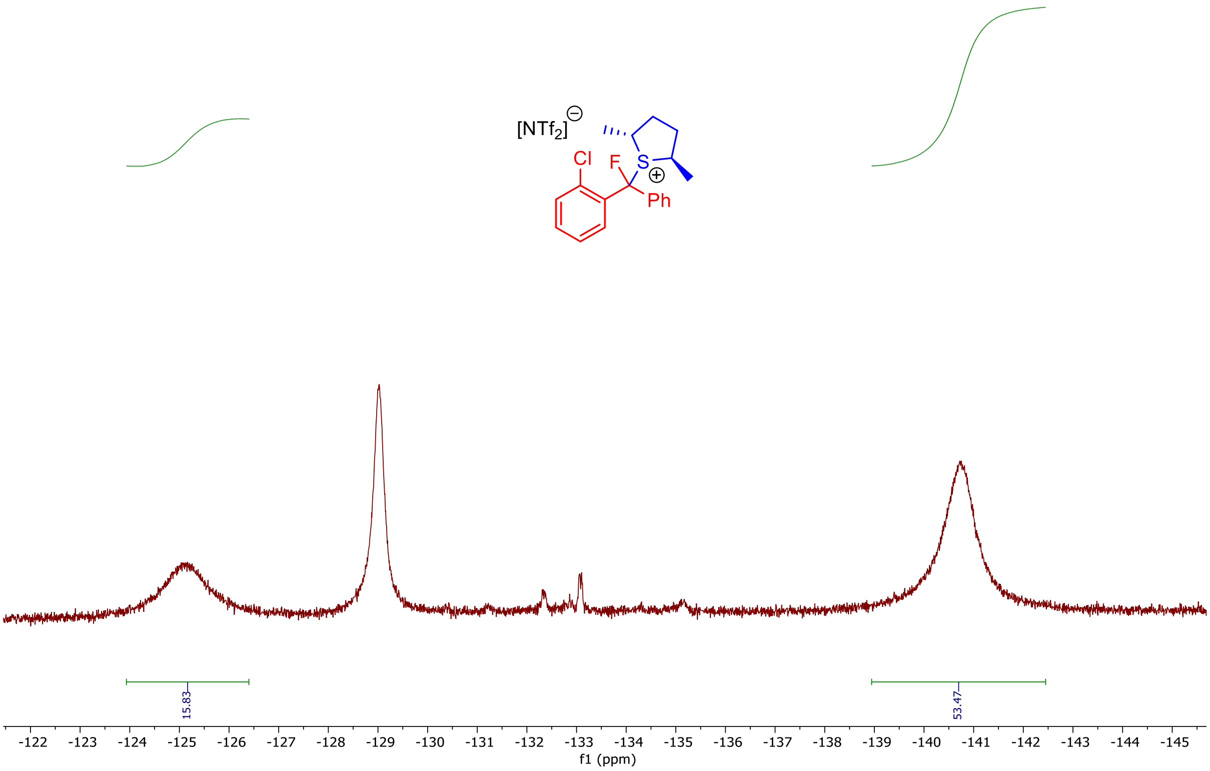

Figure S22. ^{19}F NMR spectrum of crude reaction for **2o-[A]** in DCM-d2. NMR Yield: = 69%; *dr* = 77:23. Major isomer: ^{19}F NMR (376 MHz, CD_2Cl_2): δ_F -140.7 (br, 1 F). Major isomer: ^{19}F NMR (376 MHz, CD_2Cl_2): δ_F -125.1 (br, 1 F).

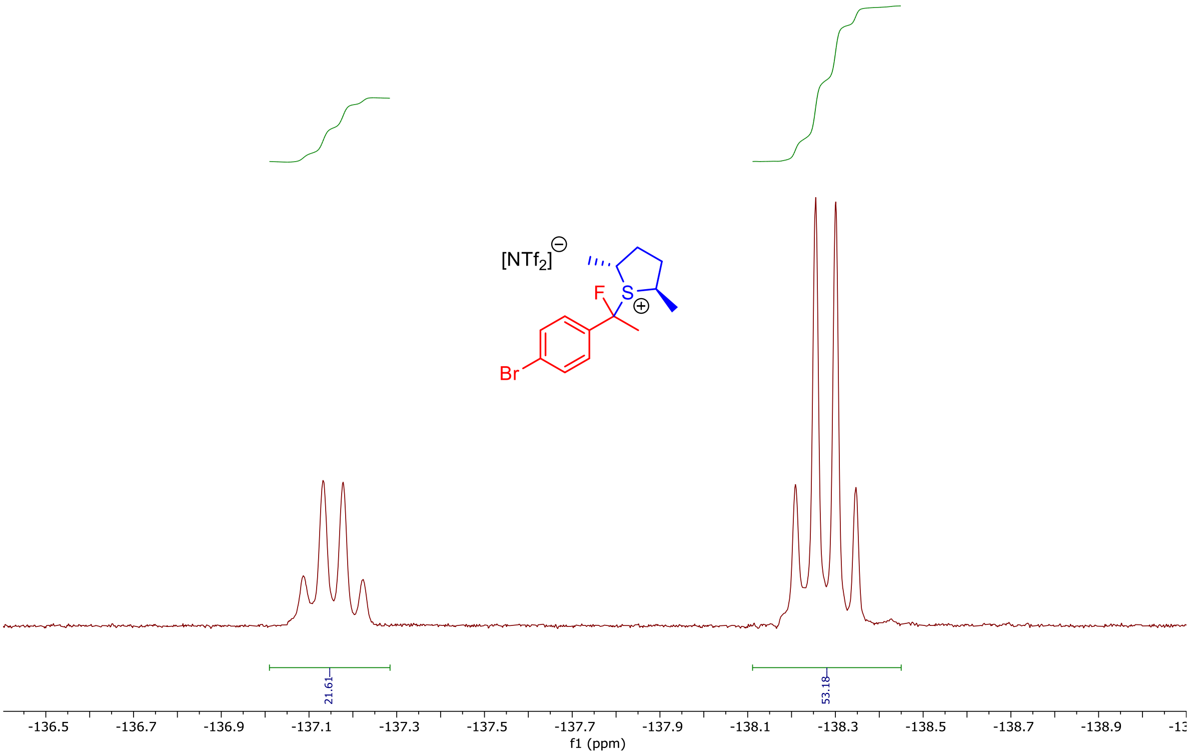

Figure S23. ^{19}F NMR spectrum of crude reaction for **2p-[A]** in DCM-d2. NMR Yield: = 74%; *dr* = 71:29. Major isomer: ^{19}F NMR (376 MHz, CD_2Cl_2): δ_F -138.3 (q, $^2J_{FH}$ = 21.7 Hz, 1 F). Minor isomer: ^{19}F NMR (376 MHz, CD_2Cl_2): δ_F -137.3 (q, $^2J_{FH}$ = 21.7 Hz, 1 F).

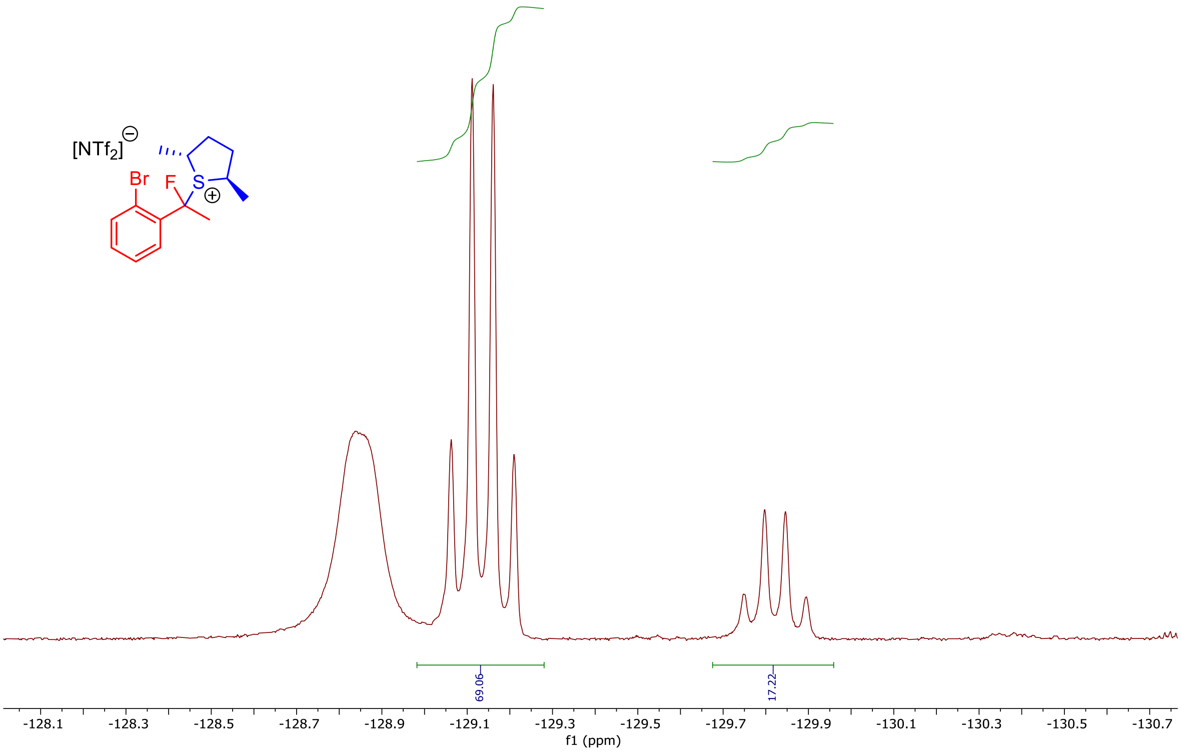

Figure S24. ^{19}F NMR spectrum of crude reaction for **2q-[A]** in DCM-d2. NMR Yield: = 86%; *dr* = 80:20. Major isomer: ^{19}F NMR (376 MHz, CD_2Cl_2): δ_F -129.2 (q, $^2J_{FH}$ = 22.9 Hz, 1 F). Minor isomer: ^{19}F NMR (376 MHz, CD_2Cl_2): δ_F -129.9 (q, $^2J_{FH}$ = 22.5 Hz, 1 F).

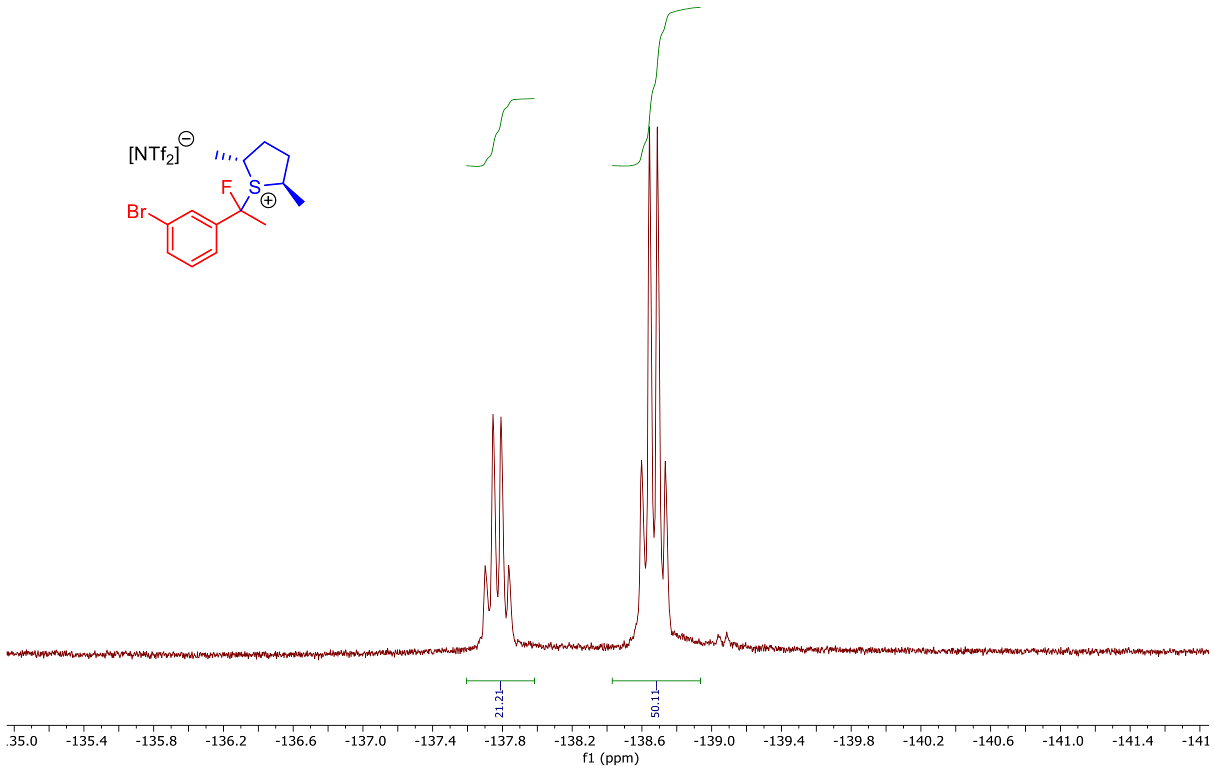

Figure S25. ^{19}F NMR spectrum of crude reaction for **2r-[A]** in DCM-d$_2$. NMR Yield: = 71%; *dr* = 71:29. Major isomer: ^{19}F NMR (376 MHz, CD_2Cl_2): δ_F -138.7 (q, $^2J_{FH}$ = 22.6 Hz, 1 F). Minor isomer: ^{19}F NMR (376 MHz, CD_2Cl_2): δ_F -137.8 (q, $^2J_{FH}$ = 22.6 Hz, 1 F).

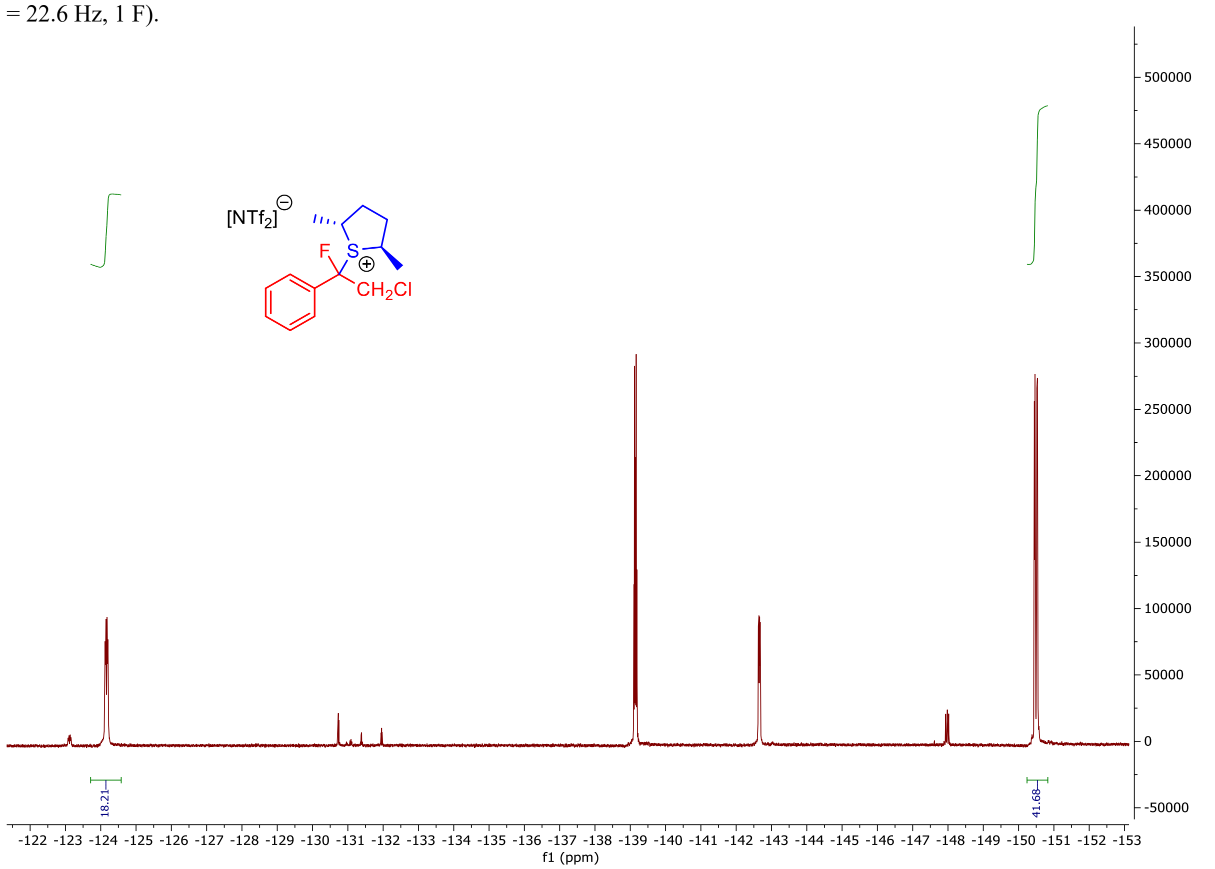

Figure S26. ^{19}F NMR spectrum of crude reaction for **2s-[A]** in DCM-d$_2$ after 2 hours. NMR Yield: = 60%; *dr* = 70:30. Major isomer: ^{19}F NMR (376 MHz, CD_2Cl_2): δ_F -150.5 (dd, $^2J_{FH}$ = 31.9, 8.0 Hz, 1 F). Minor isomer: ^{19}F NMR (376 MHz, CD_2Cl_2): δ_F -124.2 (dd, $^2J_{FH}$ = 26.5, 11.6 Hz, 1 F).

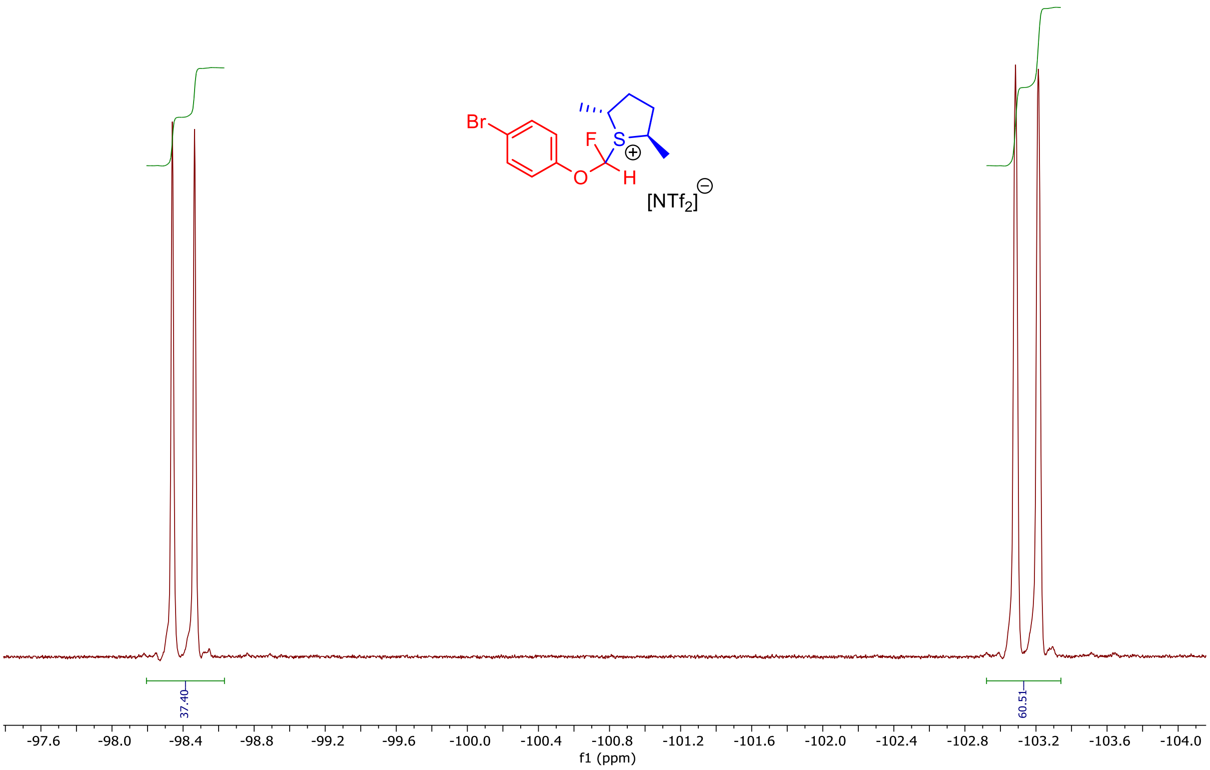

Figure S27. ${}^{19}F$ NMR spectrum of crude reaction for **2t-[A]** in DCM-d$_2$. NMR Yield: = 98%; *dr* = 62:38. Major isomer: ${}^{19}F$ NMR (376 MHz, CD_2Cl_2): δ_F -103.2 (d, ${}^{2}J_{FH}$ = 60.5 Hz, 1 F). Minor isomer: ${}^{19}F$ NMR (376 MHz, CD_2Cl_2): δ_F -98.4 (d, ${}^{2}J_{FH}$ = 58.8 Hz, 1 F).

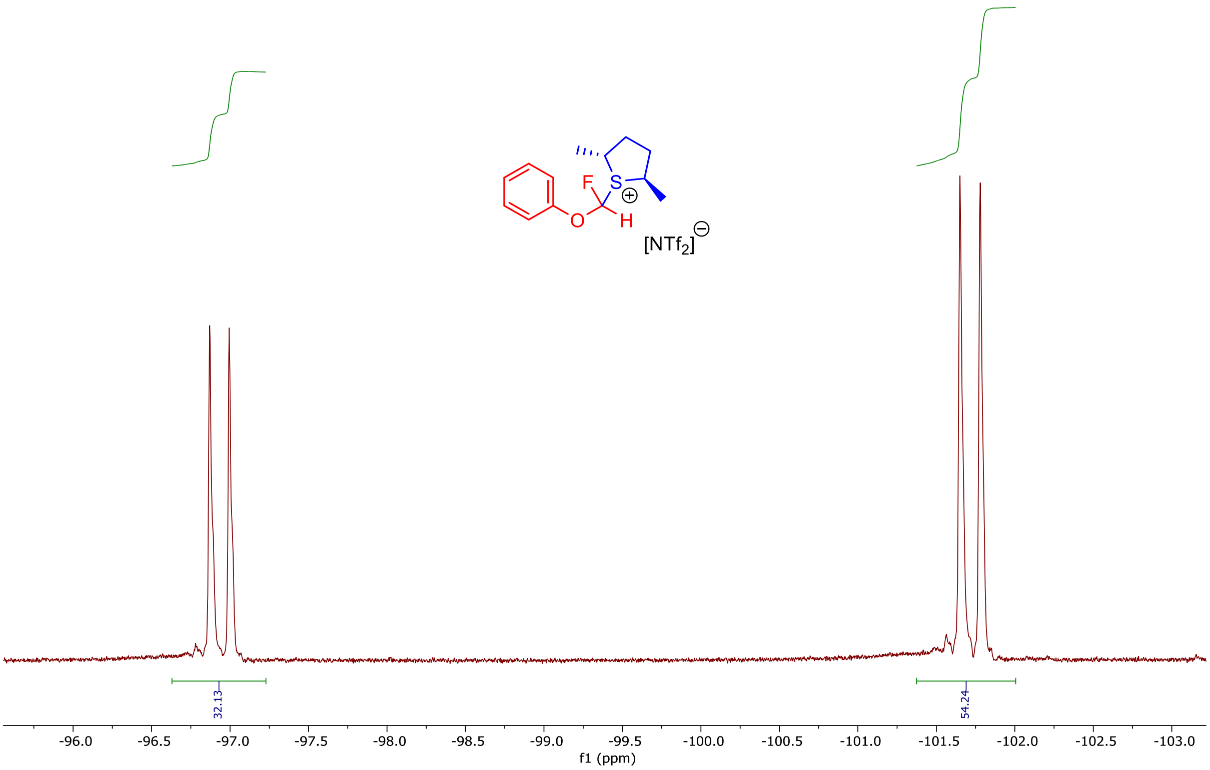

Figure S28. ${}^{19}F$ NMR spectrum of crude reaction for **2u-[A]** in DCM-d$_2$. NMR Yield: = 86%; *dr* = 64:36. Major isomer: ${}^{19}F$ NMR (376 MHz, CD_2Cl_2): δ_F -101.7 (d, ${}^{2}J_{FH}$ = 60.8 Hz, 1 F). Minor isomer: ${}^{19}F$ NMR (376 MHz, CD_2Cl_2): δ_F -96.9 (d, ${}^{2}J_{FH}$ = 58.4 Hz, 1 F).

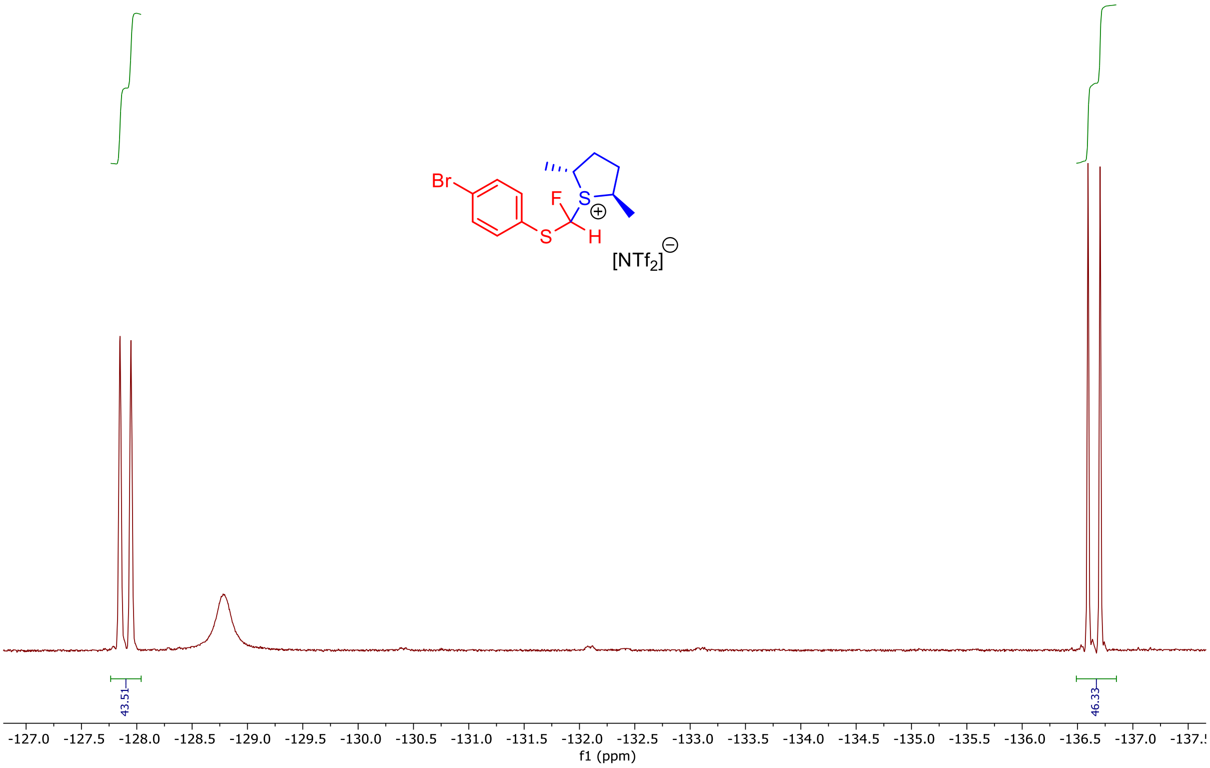

Figure S29. ^{19}F NMR spectrum of crude reaction for **2v-[A]** in DCM-d2. NMR Yield: = 99%; *dr* = 52:48. Major isomer: ^{19}F NMR (376 MHz, CD_2Cl_2): δ_F -136.7 (d, $^2J_{FH}$ = 51.5 Hz, 1 F). Minor isomer: ^{19}F NMR (376 MHz, CD_2Cl_2): δ_F -127.19(d, $^2J_{FH}$ = 46.5 Hz, 1 F).

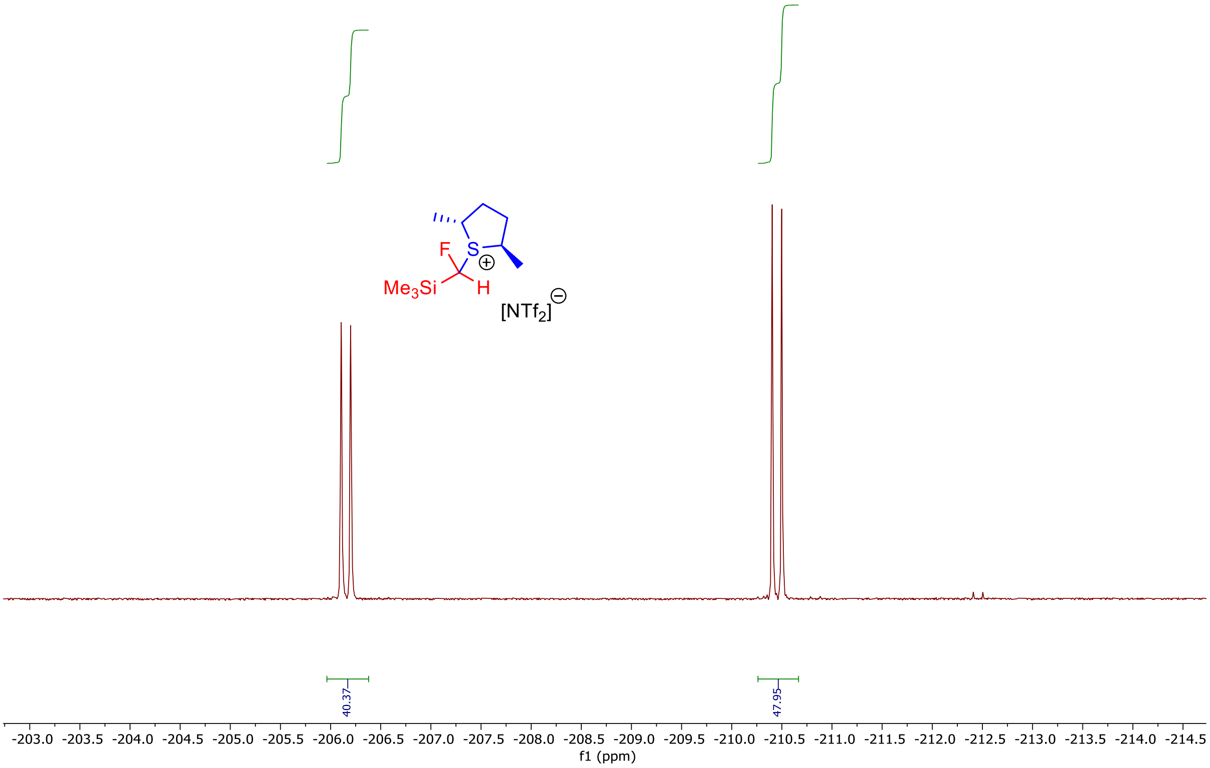

Figure S30. ^{19}F NMR spectrum of crude reaction for **2w-[A]** in DCM-d2. NMR Yield: = 88%; *dr* = 55:45. Major isomer: ^{19}F NMR (376 MHz, CD_2Cl_2): δ_F -210.5 (d, $^2J_{FH}$ = 44.7 Hz, 1 F). Minor isomer: ^{19}F NMR (376 MHz, CD_2Cl_2): δ_F -206.2 (d, $^2J_{FH}$ = 44.5 Hz, 1 F). **HRMS** (ESI-TOF) m/z: 221.1188 for $[C_{10}H_{22}FSSi]^+$ (calcd.: 221.1190).

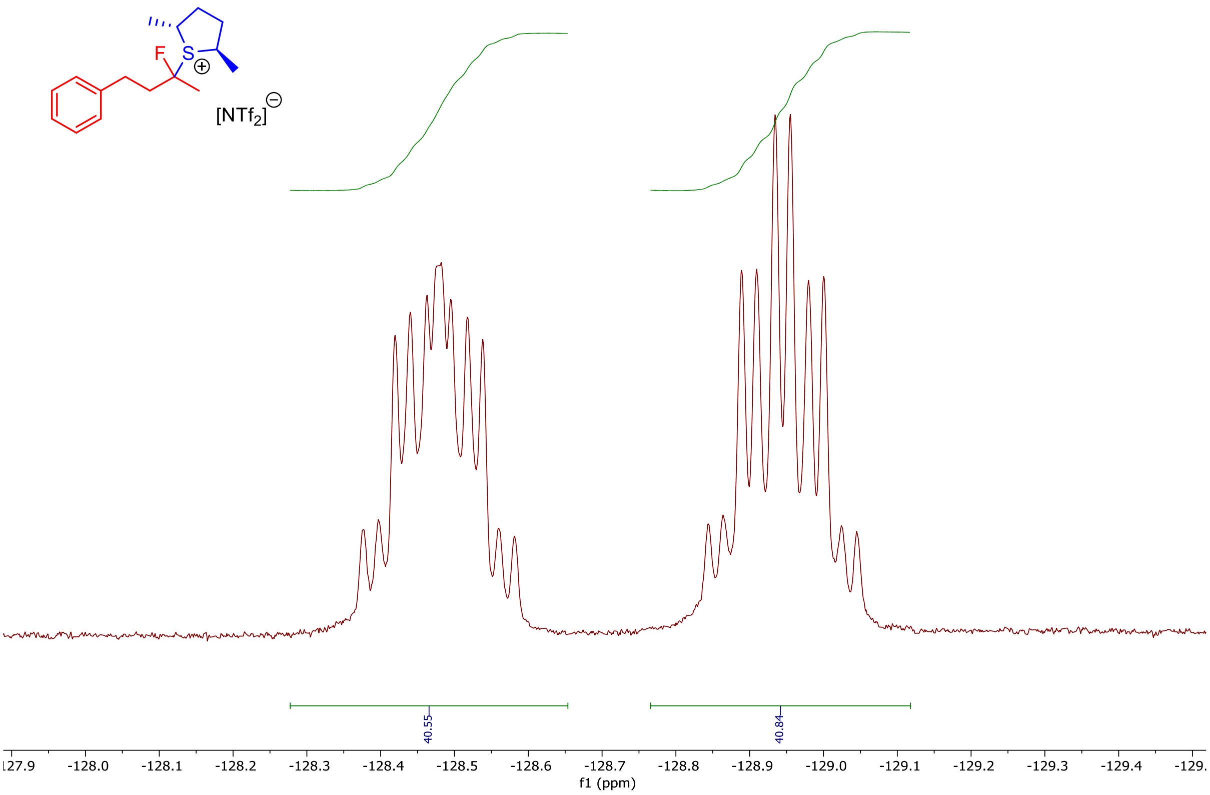

Figure S31. ^{19}F NMR spectrum of crude reaction for **2x-[A]** in DCM-d$_2$. NMR Yield: = 81%; *dr* = 50:50. Major isomer: ^{19}F NMR (376 MHz, CD_2Cl_2): δ_F -128.9 (m, 1 F). Minor isomer: ^{19}F NMR (376 MHz, CD_2Cl_2): δ_F -128.4 (m, 1 F). **HRMS** (ESI-TOF) m/z: 267.1578 for $[C_{16}H_{24}FS]^+$ (calcd.: 267.1577).

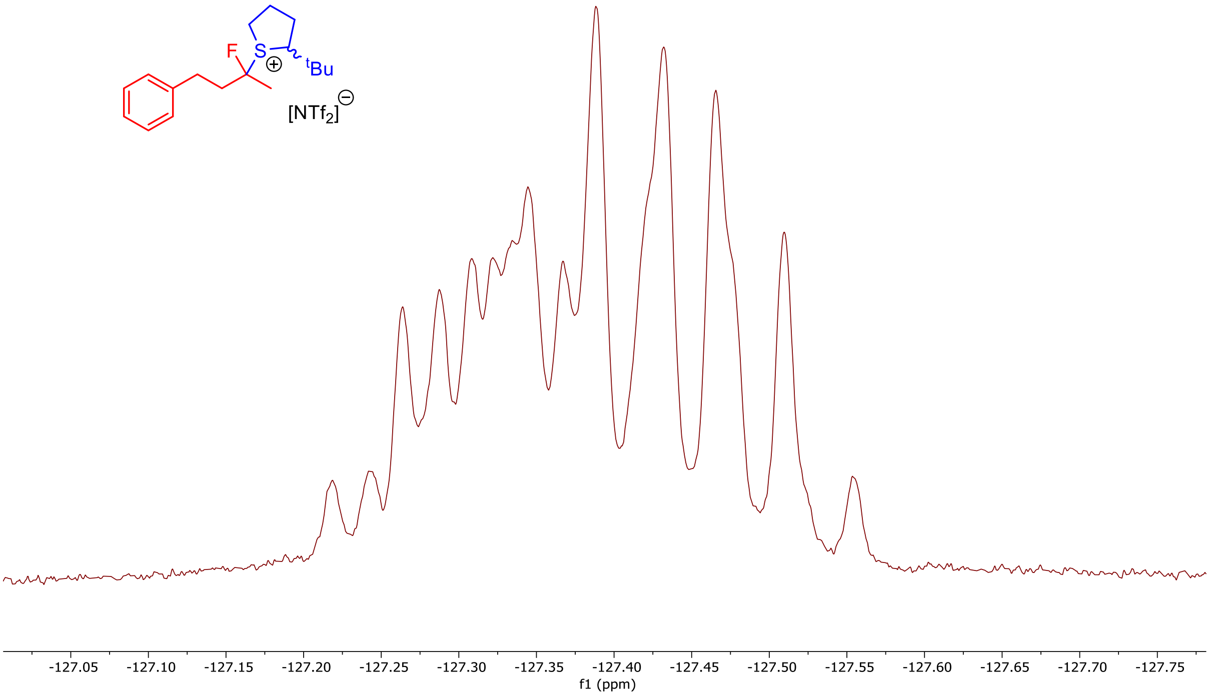

Figure S32. ^{19}F NMR spectrum of crude reaction for **2x-[B]** in DCM-d$_2$. NMR Yield: = 76%; *dr* = 50:50. Major isomer: ^{19}F NMR (376 MHz, CD_2Cl_2): δ_F -127.4 (m, 1 F). Minor isomer: ^{19}F NMR (376 MHz, CD_2Cl_2): δ_F -127.3 (m, 1 F).

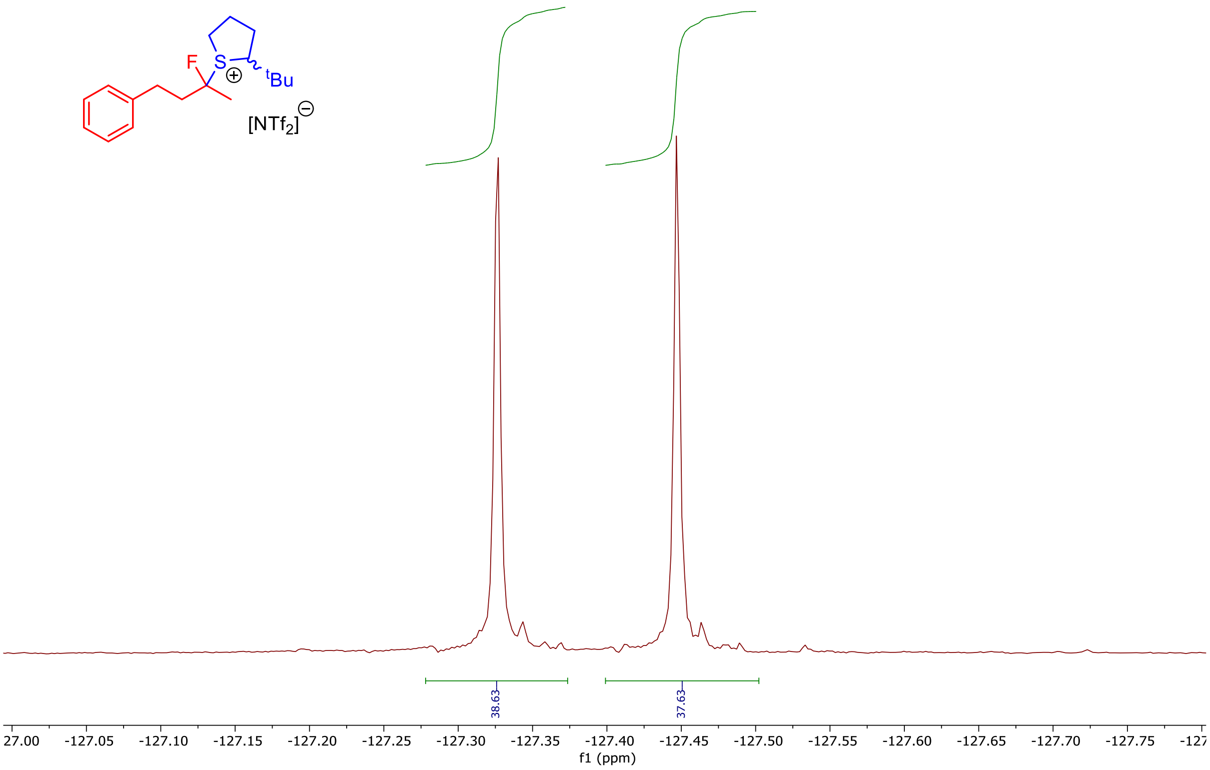

Figure S33. $^{19}F\{^{1}H\}$ NMR spectrum of crude reaction for **2x-[B]** in DCM-d_2.

Isolation and characterisation of compounds 2-[SR$_2$*]

General procedure for synthesis of salts 2-[SR$_2$]

To a mixture of BCF (1.6 mg, 0.003 mmol, 0.05 equiv.), sulfide **A** or **B** (0.09 mmol, 1.5 equiv.), TMSNTf$_2$ (32.0 mg, 0.09 mmol, 1.5 equiv.) in DCM (0.3 mL) in a 2 mL vial was added the difluoride substrate (0.06 mmol, 1 equiv.). The reaction was left for 12 hours at room temperature before evaporation to dryness. The residue was washed with hexanes (3x2 mL) and redissolved in DCM (0.5 mL). The solution was layered with hexanes and the product allowed to slowly separate as either a white solid or an oil. Excess solvent was decanted and the product dried under vacuum.

Compound of 2a-[A]

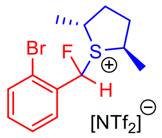

NMR Yield: 98%. Isolated yield: 29.5 mg, 84%, *dr* 71:29.

Minor isomer: ^{1}H NMR 7.87-7.56 (m, 4 H), 7.43 (d, *J* = 45.6 Hz, 1 H), 4.64-4.54 (m, 1 H), 4.55-4.45 (m, 1 H), 2.78-2.71 (m, 1 H), 2.70-2.62 (m, 1 H), 2.25-2.11 (m, 1 H), 2.10-2.00 (m, 1 H), 1.97 (d, *J* = 6.9 Hz, 3 H), 1.32-1.27 (m, 3 H); ^{13}C NMR 135.0 (d, *J* = 1.4 Hz, 1 C), 134.6 (s, 1 C), 134.3 (s, 1 C), 134.0 (s, 1 C), 129.3 (s, 1 C), 127.7 (d, *J* = 10.8 Hz, 1 C), 119.7 (q, *J* = 321.2 Hz, 2 C), 95.8 (d, *J* = 248.0 Hz, 1 C), 60.9 (s, 1 C), 56.1 (d, *J* = 4.6 Hz, 1 C), 38.3 (s, 1 C), 36.8 (s, 1 C), 18.0 (s, 1 C), 14.6 (d, *J* = 1.7 Hz, 1 C); ^{19}F NMR (376 MHz, CD_2Cl_2): δ_F -173.5 (d, $^{2}J_{FH}$ = 45.6 Hz, 1 F); HRMS (ESI-TOF) m/z: 303.0212 for $[C_{13}H_{17}BrFS]^+$ (calcd.: 303.0213).

Minor isomer: ^{1}H NMR 7.87-7.56 (m, 4 H), 7.26 (d, *J* = 46.5 Hz, 1 H), 4.64-4.54 (m, 1 H), 3.92-3.82 (m, 1 H), 2.78-2.71 (m, 1 H), 2.70-2.62 (m, 1 H), 2.33 (qd, *J* = 12.6, 5.6 Hz, 1 H), 2.10-2.00 (m, 1 H), 1.88 (dd, *J* = 6.9, 2.8 Hz, 1 H), 1.32-1.27 (m, 3 H); ^{13}C NMR 134.1 (s, 1 C), 131.2 (d, *J* = 5.0 Hz, 1 C), 129.5 (s, 1 C), 126.8 (d, *J* = 21.0 Hz, 1 C), 122.8 (d, *J* = 3.9 Hz, 1 C), 120.1 (d, *J* = 4.6 Hz, 1 C), 119.7 (q, *J* = 321.2 Hz, 2 C), 97.3 (d, *J* = 234.3 Hz, 1 C), 61.3 (s, 1 C), 61.0 (s, 1 C), 37.9 (s, 1 C), 36.8 (s, 1 C), 17.3 (s, 1 C), 14.0 (d, *J* = 3.8 Hz, 1 C); ^{19}F NMR (376 MHz, CD_2Cl_2): δ_F -152.2 (d, $^{2}J_{FH}$ = 46.5 Hz, 1 F); HRMS (ESI-TOF) m/z: 303.0212 for $[C_{13}H_{17}BrFS]^+$ (calcd.: 303.0213).

Compound 2c-[B]

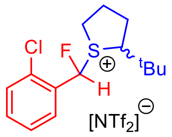

NMR yield: 67%. Isolated yield: 20.2 mg, 59%, *dr* 57:43.

Major isomer: ^{1}H NMR 7.82-7.59 (m, 4 H), 7.54 (d, *J* = 45.7 Hz, 1 H), 4.14 (dd, *J* = 10.4, 7.2 Hz, 1 H), 4.03 (ddt, *J* = 14.0, 6.3, 1.5 Hz, 1 H), 3.67 (ddd, *J* = 13.8, 11.8, 6.0 Hz, 1 H), 2.77-2.65 (m, 2 H), 2.57-2.40 (m, 1 H), 2.21-2.09 (m, 1 H), 0.86 (s, 9 H); ^{13}C NMR 134.2 (s, 1 C), 132.0 (d, *J* = 5.4 Hz, 1 C), 131.2 (s, 1 C), 128.8 (s, 1 C), 128.5 (d, *J* = 9.1 Hz, 1 C), 126.4 (d, *J* = 20.7 Hz, 1 C), 119.6 (q, *J* = 322.7 Hz, 2 C), 98.8 (d, *J* = 246.0 Hz, 1 C), 73.8 (s, 1 C), 44.3 (d, *J* = 1.9 Hz, 1 C), 33.7 (s, 1 C), 32.0 (s, 1 C), 31.1 (d, *J* = 2.7 Hz, 1 C), 27.3 (s, 3 C); ^{19}F NMR (376 MHz, CD_2Cl_2): δ_F -174.7 (d, $^{2}J_{FH}$ = 45.7 Hz, 1 F); HRMS (ESI-TOF) m/z: 287.1032 for $[C_{15}H_{21}ClFS]^+$ (calcd.: 287.1031).

Minor isomer: ^{1}H NMR 7.82-7.59 (m, 4 H), 7.25 (d, *J* = 45.3 Hz, 1 H), 4.39 (dd, *J* = 11.6, 6.3 Hz, 1 H), 3.76 (ddt, *J* = 14.3, 6.8, 1.7 Hz, 1 H), 3.45 (ddd, *J* = 14.0, 12.0, 6.0 Hz, 1 H), 2.77-2.65 (m, 2 H), 2.57-2.40 (m, 1 H), 2.21-2.09 (m, 1 H), 1.23 (s, 9 H); ^{13}C NMR 134.3 (s, 1 C), 131.8 (d, *J* = 5.4 Hz, 1 C), 131.2 (s, 1 C), 128.4 (s, 1 C), 128.1 (d, *J* = 9.1 Hz, 1 C), 125.8 (d, *J*

= 20.3 Hz, 1 C), 119.6 (q, J = 322.7 Hz, 2 C), 98.1 (d, J = 247.0 Hz, 1 C), 77.7 (s, 1 C), 40.0 (d, J = 1.8 Hz, 1 C), 34.2 (s, 1 C), 32.0 (s, 1 C), 30.7 (d, J = 1.8 Hz, 1 C), 27.7 (s, 3 C); ^{19}F NMR (376 MHz, CD_2Cl_2): δ_F -174.6 (d, $^2J_{FH}$ = 45.3 Hz, 1 F); HRMS (ESI-TOF) m/z: 287.1032 for $[C_{15}H_{21}ClFS]^+$ (calcd.: 287.1031).

Compound 2i-[A]

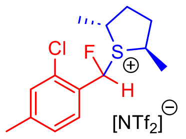

NMR Yield: 99%. Isolated yield: 31.6 mg, 88%, *dr* 77:23.

Major isomer: ^{1}H NMR 7.75-7.25 (m, 3 H), 7.44 (d, J = 45.6 Hz, 1 H), 4.61-4.50 (m, 1 H), 4.50-4.40 (m, 1 H), 2.79-2.59 (m, 2 H), 2.47 (s, 3 H), 2.23-2.11 (m, 1 H), 2.10-1.98 (m, 1 H), 1.94 (d, J = 6.5 Hz, 3 H), 1.30 (d, J = 6.7 Hz, 3 H); ^{13}C NMR 145.4 (s, 1 C), 131.4 (d, J = 1.3 Hz, 1 C), 130.9 (d, J = 4.8 Hz, 1 C), 129.5 (d, J = 1.0 Hz, 1 C), 127.0 (d, J = 9.7 Hz, 1 C), 122.1 (d, J = 20.1 Hz, 1 C), 119.6 (q, J = 322.7 Hz, 2 C), 94.2 (d, J = 242.6 Hz, 1 C), 60.8 (s, 1 C), 56.0 (d, J = 4.6 Hz, 1 C), 38.3 (d, J = 1.4 Hz, 1 C), 36.8 (s, 1 C), 21.0 (s, 1 C), 18.1 (s, 1 C), 14.5 (d, J = 2.4 Hz, 1 C); ^{19}F NMR (376 MHz, CD_2Cl_2): δ_F -174.8 (d, $^2J_{FH}$ = 45.6 Hz, 1 F); HRMS (ESI-TOF) m/z: 273.0876 for $[C_{14}H_{19}ClFS]^+$ (calcd.: 273.0875).

Minor isomer: ^{1}H NMR 7.75-7.25 (m, 3 H), 7.19 (d, J = 45.6 Hz, 1 H), 4.61-4.50 (m, 1 H), 3.80-3.72 (m, 1 H), 2.79-2.59 (m, 2 H), 2.31 (qd, J = 13.8, 5.2 Hz, 2 H), 1.88 (dd, J = 6.7, 2.7 Hz, 3 H), 1.25 (d, J = 7.1 Hz, 3 H); ^{19}F NMR (376 MHz, CD_2Cl_2): δ_F -150.2 (d, $^2J_{FH}$ = 45.6 Hz, 1 F); HRMS (ESI-TOF) m/z: 273.0876 for $[C_{14}H_{19}ClFS]^+$ (calcd.: 273.0875).

Compound 2i-[B]

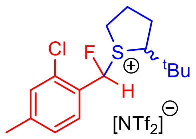

NMR Yield: 89%. Isolated yield: 25.1 mg, 72%, *dr* 60:40. Recrystallisation of **2i-[B]** from dcm/n-hexane generated a diastereomerically pure sample (see Figure S16) that regenerated a diastereomeric mixture over time when redissolved.

Major isomer: ^{1}H NMR 7.69-7.38 (m, 3 H), 7.46 (d, J = 45.6 Hz, 1 H), 4.16 (dd, J = 10.5, 7.0 Hz, 1 H), 3.93 (dd, J = 14.0, 6.5 Hz, 1 H), 3.62 (td, J = 13.0, 6.0 Hz, 1 H), 2.75-2.63 (m, 2 H), 2.55-2.41 (m, 1 H), 2.46 (s, 3 H), 2.20-2.08 (m, 1 H), 0.89 (d, J = 1.1 Hz, 9 H); ^{13}C NMR 145.8 (s, 1 C), 131.5 (s, 1 C), 129.2 (s, 1 C), 128.4 (d, J = 9.2 Hz, 1 C), 119.7 (q, J = 325.0 Hz, 2 C), 99.1 (d, J = 244.5 Hz, 1 C), 74.0 (s, 1 C), 44.0 (s, 1 C), 33.7 (s, 1 C), 32.0 (s, 1 C), 31.0 (s, 1 C), 27.4 (s, 3 C), 21.0 (s, 1 C); ^{19}F NMR (376 MHz, CD_2Cl_2): δ_F -173.1 (d, $^2J_{FH}$ = 45.6 Hz, 1 F); HRMS (ESI-TOF) m/z: 301.1187 for $[C_{16}H_{23}ClFS]^+$ (calcd.: 301.1188).

Minor isomer: ^{1}H NMR 7.69-7.38 (m, 3 H), 7.20 (d, J = 44.3 Hz, 1 H), 4.34 (dd, J = 11.2, 6.1 Hz, 1 H), 3.75 (dd, J = 14.4 Hz, 7.0 Hz, 1 H), 3.44 (td, J = 13.0, 6.0 Hz, 1 H), 2.75-2.63 (m, 2 H), 2.55-2.41 (m, 1 H), 2.47 (s, 3 H), 2.20-2.08 (m, 1 H), 1.20 (d, J = 1.1 Hz, 9 H); ^{13}C NMR 145.9 (s, 1 C), 129.5 (s, 1 C), 128.0 (d, J = 9.2 Hz, 1 C), 119.7 (q, J = 325.0 Hz, 2 C), 98.4 (d, J = 245.0 Hz, 1 C), 77.3 (s, 1 C), 40 .0 (s, 1 C), 34.1 (s, 1 C), 32.0 (s, 1 C), 30.6 (s, 1 C), 27.7 (s, 3 C), 21.0 (s, 1 C); ^{19}F NMR (376 MHz, CD_2Cl_2): δ_F -173.0 (d, $^2J_{FH}$ = 44.3 Hz, 1 F); HRMS (ESI-TOF) m/z: 301.1187 for $[C_{16}H_{23}ClFS]^+$ (calcd.: 301.1188).

Compound 2h-[A]

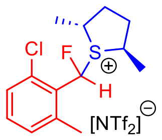

NMR Yield: 91%. Isolated yield: 26.9 mg, 75%, *dr* 83:17.

Major isomer: ^{1}H NMR 7.60-7.32 (m, 3 H), 7.51 (d, J = 47.2 Hz, 1 H), 4.75-4.64 (m, 1 H), 3.64-3.55 (m, 1 H), 2.85-2.77 (m, 1 H), 2.69 (d, J = 3.2 Hz, 3 H), 2.64-2.57 (m, 1 H), 2.22 (qd, J = 13.1, 4.8 Hz, 1 H), 2.06 (qd, J = 13.1, 4.8 Hz, 1 H), 1.91 (dd, J = 6.8, 2.5 Hz, 3 H), 1.25 (d, J = 6.9 Hz, 3 H); ^{13}C NMR 142.4 (s, 1 C), 134.6 (d, J = 2.2 Hz, 1 C), 132.4 (d, J = 2.3 Hz, 1 C), 129.4 (s, 1 C), 128.8 (d, J = 1.2 Hz, 1 C), 122.8 (d, J = 17.9 Hz, 1 C), 119.6 (q, J = 322.7 Hz, 2 C), 94.1 (d, J = 232.4 Hz, 1 C), 61.2 (d, J = 2.0 Hz, 1 C), 60.2 (s, 1 C), 37.7 (s, 1 C), 36.8 (s, 1 C), 20.3 (d, J = 4.4 Hz, 1 C), 17.4 (s, 1 C), 13.7 (d, J = 3.6 Hz, 1 C); ^{19}F NMR (376 MHz, CD_2Cl_2): δ_F -154.3 (d, $^{2}J_{FH}$ = 47.2 Hz, 1 F); HRMS (ESI-TOF) m/z: 273.0874 for $[C_{14}H_{19}ClFS]^+$ (calcd.: 273.0875).

Minor isomer: ^{1}H NMR 7.60-7.32 (m, 3 H), 7.50 (d, J = 46.0 Hz, 1 H), 4.75-4.64 (m, 1 H), 4.57-4.48 (m, 1 H), 2.85-2.77 (m, 1 H), 2.66 (d, J = 4.5 Hz, 3 H), 2.64-2.57 (m, 1 H), 1.81 (ddd, J = 11.8, 7.0, 0.6 Hz, 2 H), 1.75 (d, J = 6.8 Hz, 3 H), 1.65 (d, J = 6.8 Hz, 3 H); ^{19}F NMR (376 MHz, CD_2Cl_2): δ_F -166.6 (d, $^{2}J_{FH}$ = 46.0 Hz, 1 F); HRMS (ESI-TOF) m/z: 273.0874 for $[C_{14}H_{19}ClFS]^+$ (calcd.: 273.0875).

Compound 2h-[B]

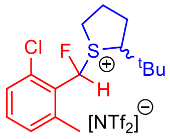

NMR Yield: 78%. Isolated yield: 21.7 mg, 62%, *dr* 66:34.

Major isomer: ^{1}H NMR 7.58-7.36 (m, 3 H), 7.46 (d, J = 44.6 Hz, 1 H), 4.54 (dd, J = 11.5, 6.8 Hz, 1 H), 3.46-3.37 (m, 1 H), 3.34-3.28 (m, 1 H), 2.94-2.69 (m, 3 H), 2.65 (d, J = 3.6 Hz, 3 H), 2.62-2.52 (m, 1 H), 2.24-2.12 (m, 1 H), 1.23 (s, 9 H); ^{13}C NMR 141.8 (s, 1 C), 134.6 (s, 1 C), 134.4 (s, 1 C), 132.3 (s, 1 C), 132.1 (s, 1 C), 129.1 (s, 1 C), 119.6 (q, J = 322.7 Hz, 2 C), 98.5 (d, J = 236.6 Hz, 1 C), 79.8 (d, J = 3.0 Hz, 1 C), 42.8 (s, 1 C), 34.2 (s, 1 C), 32.6 (s, 1 C), 31.1 (s, 1 C), 27.8 (s, 3 C), 20.4 (d, J = 1.5 Hz, 1 C); ^{19}F NMR (376 MHz, CD_2Cl_2): δ_F -165.4 (d, $^{2}J_{FH}$ = 44.6 Hz, 1 F); HRMS (ESI-TOF) m/z: 301.1188 for $[C_{16}H_{23}ClFS]^+$ (calcd.: 301.1188).

Minor isomer: ^{1}H NMR 7.58-7.36 (m, 3 H), 7.37 (d, J = 45.7 Hz, 1 H), 4.05 (dd, J = 14.4, 6.1 Hz, 1 H), 3.92 (dd, J = 11.0, 7.0 Hz, 1 H), 3.71-3.63 (m, 1 H), 2.94-2.69 (m, 3 H), 2.67 (d, J = 3.9 Hz, 3 H), 2.62-2.52 (m, 1 H), 2.24-2.12 (m, 1 H), 0.87 (s, 9 H); ^{13}C NMR 142.1 (s, 1 C), 134.6 (s, 1 C), 134.4 (s, 1 C), 132,3 (s, 1 C), 132.1 (s, 1 C), 128.9 (s, 1 C), 119.6 (q, J = 322.7 Hz, 2 C), 97.8 (d, J = 238.9 Hz, 1 C), 77.3 (d, J = 2.0 Hz, 1 C), 43.0 (s, 1 C), 34.1 (s, 1 C), 31.9 (s, 1 C), 30.3 (s, 1 C), 27.2 (s, 3 C), 20.4 (d, J = 2.0 Hz, 1 C); ^{19}F NMR (376 MHz, CD_2Cl_2): δ_F -158.1 (d, $^{2}J_{FH}$ = 45.7 Hz, 1 F); HRMS (ESI-TOF) m/z: 301.1188 for $[C_{16}H_{23}ClFS]^+$ (calcd.: 301.1188).

Compound 2x-[A]

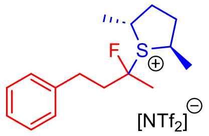

NMR Yield: 81%. Diastereomeric mixture separated via silica column chromatography, eluent DCM/Et_2O (1:4). Individual diastereomers could not be adequetely resolved. Isolated yield: 57%, 31 mg, *dr* 50:50.

Major isomer: ^{1}H NMR 7.36-7.15 (m, 5 H), 4.5-4.3 (m, 2 H), 2.9-2.3 (m, 6 H), 2.04-1.84 (m, 2 H), 1.81 (d, J = 6.9 Hz, 3 H), 1.69 (d, J = 6.9 Hz, 3 H); ^{13}C NMR 138.7 (s, 1 C), 129.4 (s, 2 C), 128.6 (s, 2 C), 127.5 (s, 1 C), 120.1 (q, J = 320.4 Hz, 2 C), 113.6 (d, J = 249.5 Hz, 1 C), 64.3 (s, 1 C), 59.4 (s, 1 C), 41.2 (d, J = 29.3 Hz, 1 C), 39.0 (d, J = 0.8 Hz, 1 C), 36.5 (d, J = 0.8 Hz, 1 C), 29.3 (d, J = 4.5 Hz, 1 C), 24.5 (d, J = 21.3 Hz, 1 C), 19.4 (s, 1 C), 16.3 (d, J = 2.9 Hz); ^{19}F NMR (376 MHz, CD_2Cl_2): δ_F -128.6 (m, 1 F).

Minor isomer: ^{1}H NMR 7.36-7.15 (m, 5 H), 4.5-4.3 (m, 2 H), 2.9-2.3 (m, 6 H), 1.96 (d, J = 20.2 Hz, 3 H), 1.76 (dd, J = 7.0, 0.7 Hz, 3 H), 1.64 (d, J = 6.8, 3 H); ^{13}C NMR 138.4 (s, 1 C), 129.3 (s, 2 C), 128.6 (s, 2 C), 127.4 (s, 1 C), 120.1 (q, J = 320.4 Hz, 2 C), 113.2 (d, J = 249.5 Hz, 1 C), 64.2 (s, 1 C), 58.7 (s, 1 C), 41.0 (d, J = 29.9 Hz, 1 C), 38.9 (d, J = 0.8 Hz, 1 C), 30.0 (s, 1 C), 29.4 (d, J = 4.5 Hz, 1 C), 24.2 (d, J = 21.3 Hz, 1 C), 19.4 (s, 1 C), 16.1 (d, J = 2.9 Hz); ^{19}F NMR (376 MHz, CD_2Cl_2): δ_F -129.0 (m, 1 F).

Compound 2x-[B]

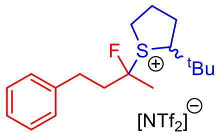

NMR Yield: 76%, *dr* 50:50. Isolated yield for 2nd recrystallization (used for SCXRD): 4.2 mg, 12%.

Major isomer: ^{1}H NMR 7.42-7.24 (m, 5 H), 4.22 (t, J = 7.7 Hz, 1 H), 3.89-3.83 (m, 1 H), 3.70-3.63 (m, 1 H), 3.01-2.90 (m, 2 H), 2.65-2.46 (m, 4 H), 2.44-2.33 (m, 1 H), 2.28-2.19 (m, 1 H), 2.14 (d, J = 21.3 Hz, 3 H), 1.19 (d, J = 7.3 Hz, 9 H); ^{13}C NMR 138.1 (s, 1 C), 128.9 (s, 2 C), 128.2 (s, 2 C), 127.0 (s, 1 C), 120.1 (q, J = 320.4 Hz, 2 C), 110.7 (br, 1 C), 73.9 (s, 1 C), 42.0 (s, 1 C), 39.7 (d, J = 20.1 Hz, 1 C), 30.8 (s, 1 C), 29.2 (s, 1 C), 28.0 (s, 3 C), 22.5 (d, J = 20.8 Hz, 1 C); ^{19}F NMR (376 MHz, CD_2Cl_2): δ_F -127.4 (m, 1 F).

Minor isomer: ^{1}H NMR 7.42-7.24 (m, 5 H), 4.18 (t, J = 7.7 Hz, 1 H), 3.82-3.77 (m, 1 H), 3.59-3.52 (m, 1 H), 3.01-2.90 (m, 2 H), 2.65-2.46 (m, 4 H), 2.44-2.33 (m, 1 H), 2.28-2.19 (m, 1 H), 2.09 (d, J = 21.3 Hz, 3 H), 1.19 (d, J = 7.3 Hz, 9 H); ^{13}C NMR 138.2 (s, 1 C), 129.0 (s, 2 C), 128.4 (s, 2 C), 127.2 (s, 1 C), 120.1 (q, J = 320.4 Hz, 2 C), 112.7 (br, 1 C), 74.1 (s, 1 C), 42.3 (s, 1 C), 40.2 (d, J = 20.1 Hz, 1 C), 31.7 (s, 1 C), 29.5 (s, 1 C), 28.0 (s, 3 C), 23.4 (d, J = 20.8 Hz, 1 C); ^{19}F NMR (376 MHz, CD_2Cl_2): δ_F -127.3 (m, 1 F).

Synthesis and characterisation of stereoenriched salts 2-[N*S*] (chiral resolution)

General procedure for synthesis of salts 2-[N*S*]

To a mixture of BCF (1.5 mg, 0.003 mmol, 0.05 equiv.), sulfide **A** (10.4 mg, 0.09 mmol, 1.5 equiv.), $TMSNTf_2$ (32.0 mg, 0.09 mmol, 1.5 equiv.) and $PhOCF_3$ (9.7 mg, 0.06 mmol, 1 equiv., IS) in DCM (0.3 mL) in a J. Young's NMR tube was added the difluoride substrate (0.06 mmol, 1 equiv.). The reaction was left for 12-24 hours at room temperature before ^{19}F NMR analysis was performed to determine NMR yield and selectivity of the intermediuate **2-[A]**. The enantiopure chiral amine **N*S*** {(*S*)-*N,N*-dimethyl-1-phenylethylamine} (89.4 mg, 0.06 mmol, 1.0 equiv.) was added and the reaction left for another 5 hours before NMR analysis to determine yield and selectivity. Compounds **2a-[N*S*], 2c-[N*S*]** and **2i-[N*S*]** were isolated via silica column chromatography using hexane/dcm eluent.

Compound 2a-[N*S*]

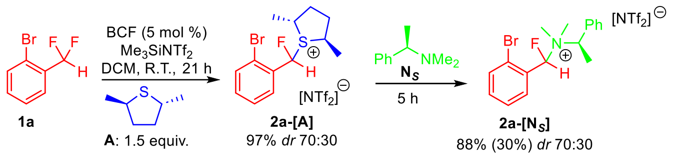

NMR Yield (based on **1a**): 88%, *dr* 70:30. Isolated yield (based on **1a**): 11 mg, 30%, *dr* 72:28 (Note: partial decomposition observed on silica).

Major isomer: ^1H NMR (500 MHz, $CDCl_3$) δ_H 7.76 - 7.44 (m, 9H), 6.92 (d, J = 43.2 Hz, 1H), 5.24 (q, J = 7.1 Hz, 1H), 3.32 (s, 3H), 2.95 (s, 3H), 2.09 (dd, J = 7.1, 3.2 Hz, 3H); ^{19}F NMR (471 MHz, $CDCl_3$) δ_F -78.7 (6F), -152.8 (br, 1F); HRMS (ESI-TOF) m/z: 336.0756 for $[C_{17}H_{20}BrFN]^+$ (calcd.: 336.0758).

Minor isomer: ^1H NMR (500 MHz, $CDCl_3$) δ_H 7.76 - 7.44 (m, 9H), 6.52 (d, J = 42.4 Hz, 1H), 5.15 (q, J = 7.0 Hz, 1H), 3.36 (s, 3H), 2.97 (s, 3H), 2.06 (d, J = 7.1 Hz, 3H); ^{19}F NMR (471 MHz, $CDCl_3$) δ_F -78.7 (6F), -157.9 (d, J = 42.3 Hz, 1F); HRMS (ESI-TOF) m/z: 336.0756 for $[C_{17}H_{20}BrFN]^+$ (calcd.: 336.0758).

Compound 2c-[N*S*]

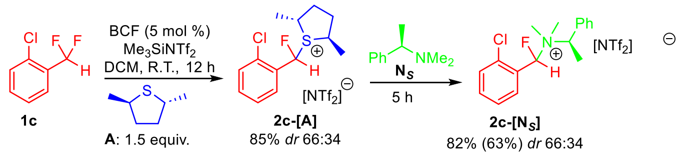

NMR Yield (based on **1c**): 82%, *dr* 66:34. Isolated yield (based on **1c**): 21.6 mg, 63%, *dr* 95:5.

Major isomer: ^1H NMR (500MHz, $CDCl_3$): δ_H 7.76 (dd, J = 7.3, 2.3Hz, 1H), 7.65 - 7.54 (m, 7H), 7.46 (dd, J = 6.4, 1.7 Hz, 1H), 6.44 (d, J = 42.3 Hz, 1H), 5.16 (q, J = 7.0 Hz, 1H), 3.29 (d, J = 1.9 Hz, 3H), 2.87 (d, J = 1.7 Hz, 3H), 1.99 (d, J = 7.0 Hz, 3H); ^{19}F NMR (376 MHz, CD_2Cl_2): δ_F -78.9 (s, 6 F), -157.0 (br, 1 F); HRMS (ESI-TOF) m/z: 292.1263 for $[C_{17}H_{20}ClFN]^+$ (calcd.: 292.1263).

Minor isomer: ^{19}F NMR (471 MHz, $CDCl_3$) δ_F -78.9 (s, 6F), -159.3 (d, J = 42.2 Hz, 1F); HRMS (ESI-TOF) m/z: 292.1263 for $[C_{17}H_{20}ClFN]^+$ (calcd.: 292.1263).

Compound 2e-[N*S*]

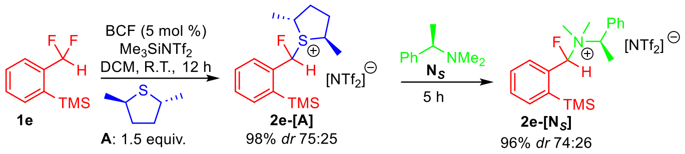

NMR Yield (based on **1e**): 96%, *dr* 74:26.

Major isomer: ^{19}F NMR (376 MHz, CD_2Cl_2): δ_F -78.8 (s, 6 F), -147.0 (d, J = 42.6 Hz, 1 F); HRMS (ESI-TOF) m/z: 330.2053 for $[C_{20}H_{29}FNSi]^+$ (calcd.: 330.2048).

Minor isomer: ^{19}F NMR (377 MHz, $CDCl_3$) δ_F -78.8 (s, 6F), -151.1 (d, J = 42.5 Hz, 1 F); HRMS (ESI-TOF) m/z: 330.2053 for $[C_{20}H_{29}FNSi]^+$ (calcd.: 330.2048).

Compound 2i-[N*S*]

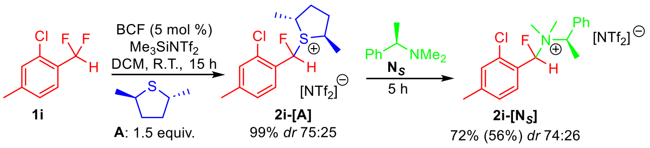

NMR Yield (based on **1i**): 72%, *dr* 74:26. Isolated yield (based on **1i**): 19.7 mg, 56%, *dr* 95:5.

Major isomer: ^{1}H NMR (400 MHz, $CDCl_3$): δ_H 7.59 (m, J = 8.5 Hz, 5H), 7.35 (d, J = 8.1 Hz, 2H), 7.28 (s, 1H), 6.40 (d, J = 42.3 Hz, 1H), 5.10 (q, J = 7.0 Hz, 1H), 3.29 (s, 3H), 2.87 (s, 3H), 2.41 (s, 3H), 2.01 (d, J = 7.0 Hz, 3H); ^{19}F NMR (376 MHz, CD_2Cl_2): δ_F -78.8 (s, 6 F), -157.0 (br, 1 F); HRMS (ESI-TOF) m/z: 306.1420 for $[C_{18}H_{22}ClFN]^+$ (calcd.: 306.1419).

Minor isomer: ^{19}F NMR (377 MHz, $CDCl_3$) δ_F -78.8 (s, 6F), -159.4 (d, J = 41.7 Hz, 1 F); HRMS (ESI-TOF) m/z: 306.1420 for $[C_{18}H_{22}ClFN]^+$ (calcd.: 306.1419).

Compound 2h-[N*S*]

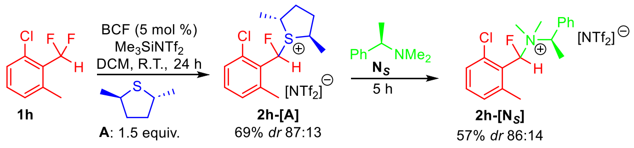

NMR Yield (based on **1h**): 57%, *dr* 86:14.

Major isomer: ^{19}F NMR (376 MHz, CD_2Cl_2): δ_F -78.8 (s, 6 F), -164.4 (d, J = 42.5 Hz, 1 F); HRMS (ESI-TOF) m/z: 306.1421 for $[C_{18}H_{22}ClFN]^+$ (calcd.: 306.1419).

Minor isomer: ^{19}F NMR (377 MHz, $CDCl_3$) δ_F -78.8 (s, 6F), -161.6 (d, J = 43.6 Hz, 1 F); HRMS (ESI-TOF) m/z: 306.1421 for $[C_{18}H_{22}ClFN]^+$ (calcd.: 306.1419).

Compound 2u-[N*S*]

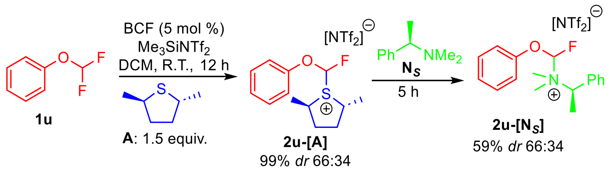

NMR Yield (based on **1u**): 59%, *dr* 66:34.

Major isomer: ^{19}F NMR (376 MHz, CD_2Cl_2): δ_F -114.7 (d, J = 65.8 Hz, 1 F); HRMS (ESI-TOF) m/z: 274.1607 for $[C_{17}H_{21}FNO]^+$ (calcd.: 274.1602).

Minor isomer: ^{19}F NMR (376 MHz, CD_2Cl_2): δ_F -112.7 (d, J = 65.3 Hz, 1 F); HRMS (ESI-TOF) m/z: 274.1607 for $[C_{17}H_{21}FNO]^+$ (calcd.: 274.1602).

NMR spectra for Figure 5

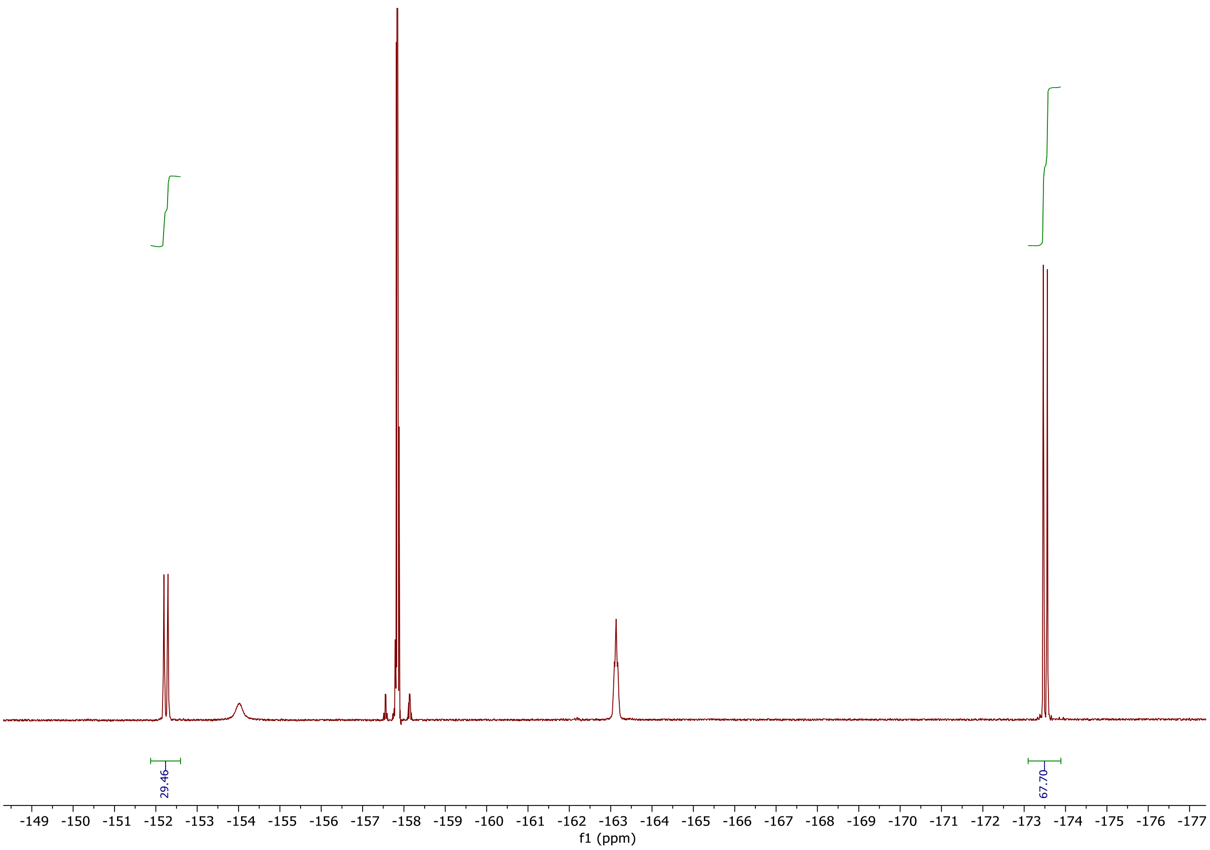

Figure S34. ^{19}F NMR spectrum of crude reaction for **2a-[A]** in DCM-d2 (used for reaction with **N*S*** in Figure S35).

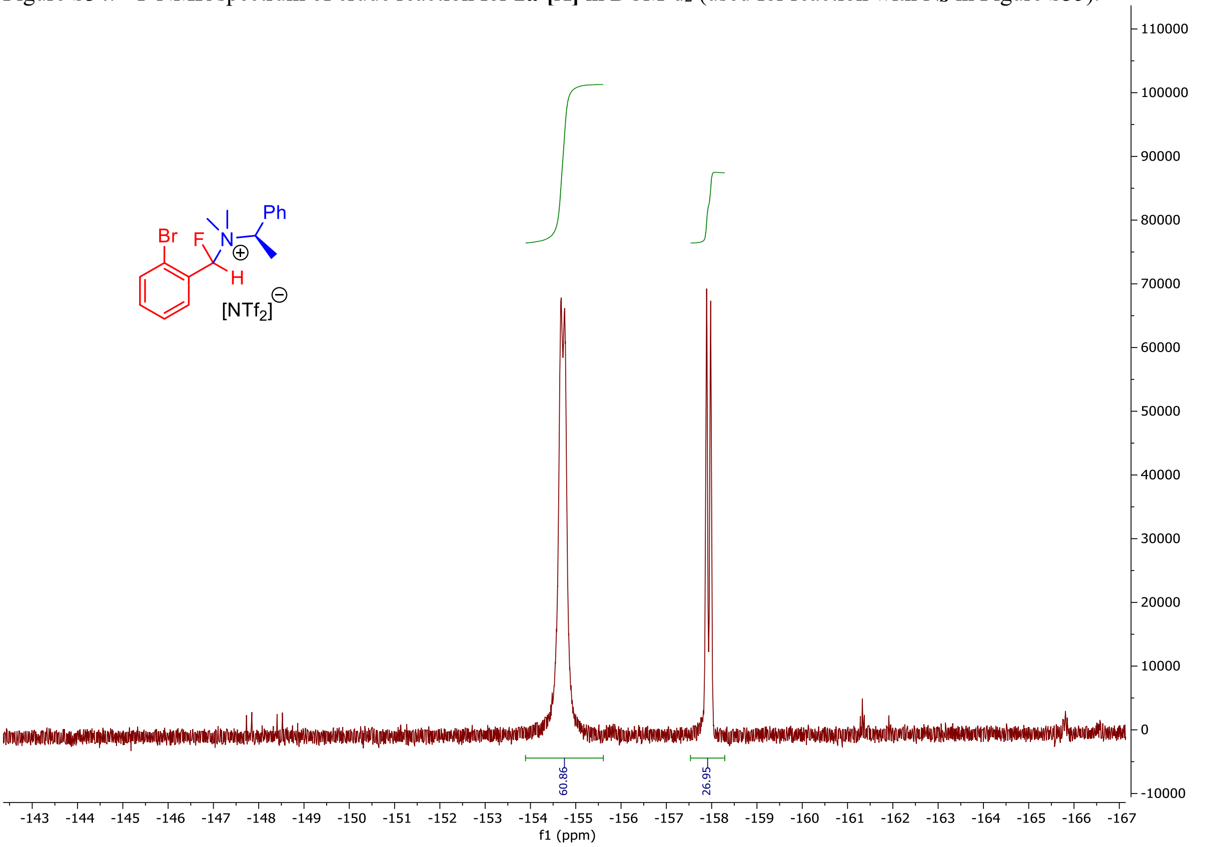

Figure S35. ^{19}F NMR spectrum of crude reaction for **2a-[N*S*]** in DCM-d2 5 hours after addition of **N*S*** and after silica filtration and evaporation (product signals coincided with Me_3SiF).

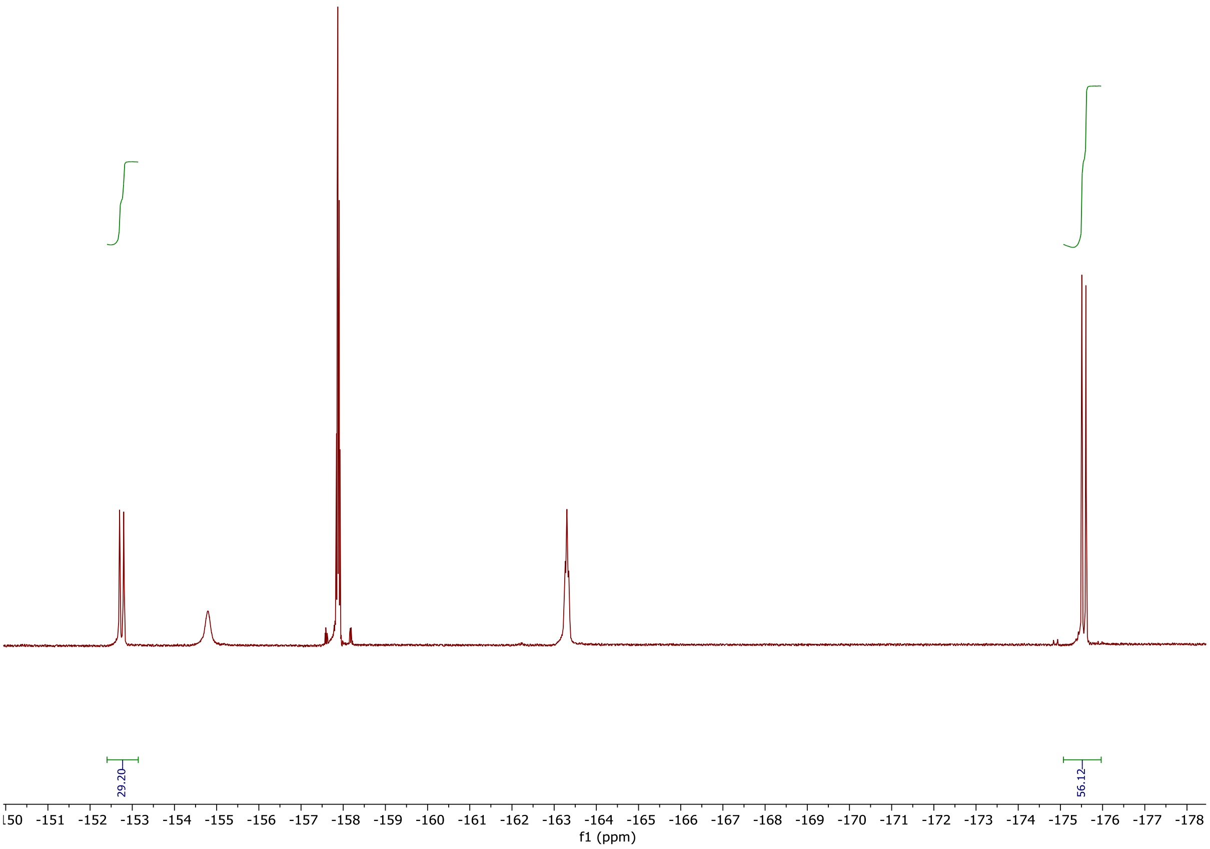

Figure S36. ^{19}F NMR spectrum of crude reaction for **2c-[A]** in DCM-d_2 (used for reaction with **N***S* in Figure S37).

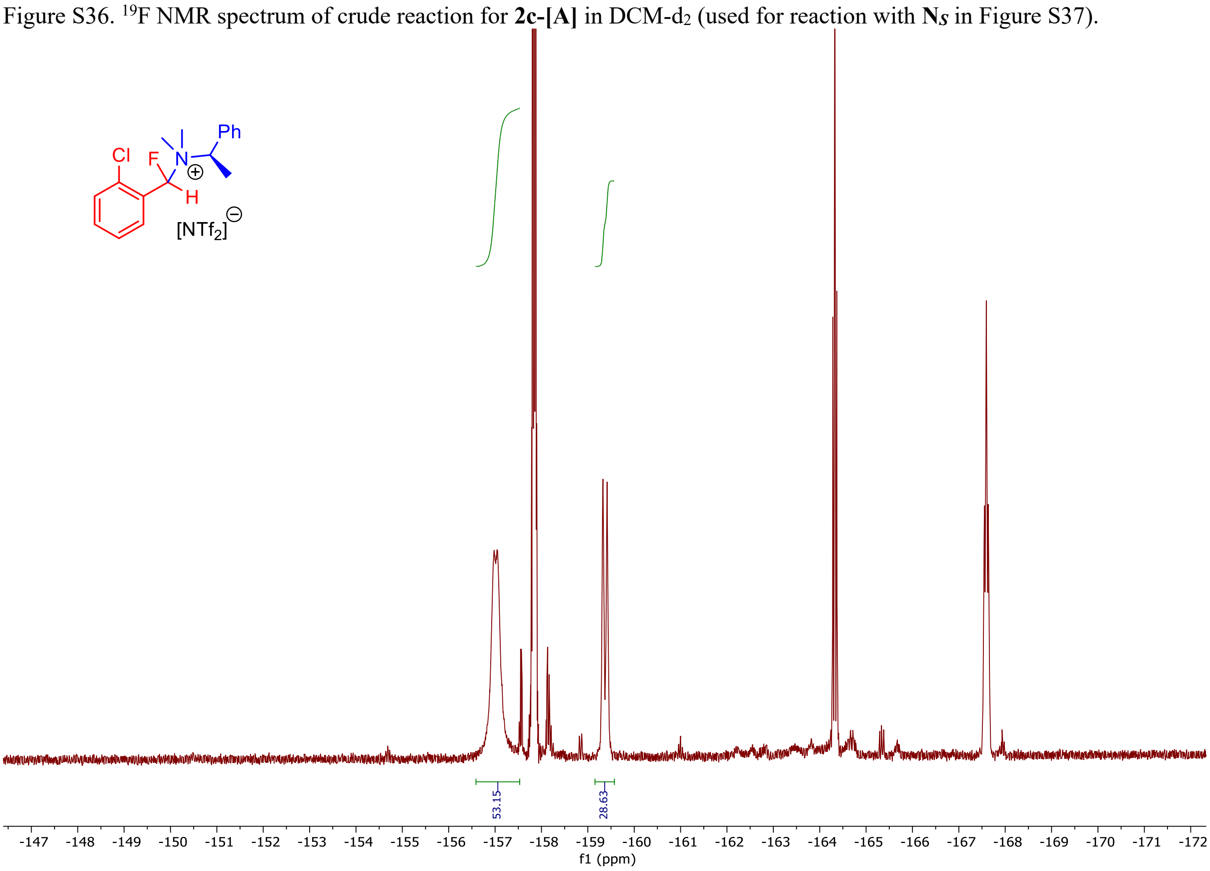

Figure S37. ^{19}F NMR spectrum of crude reaction for **2c-[N*S*]** in DCM-d_2 5 hours after addition of **N***S*.

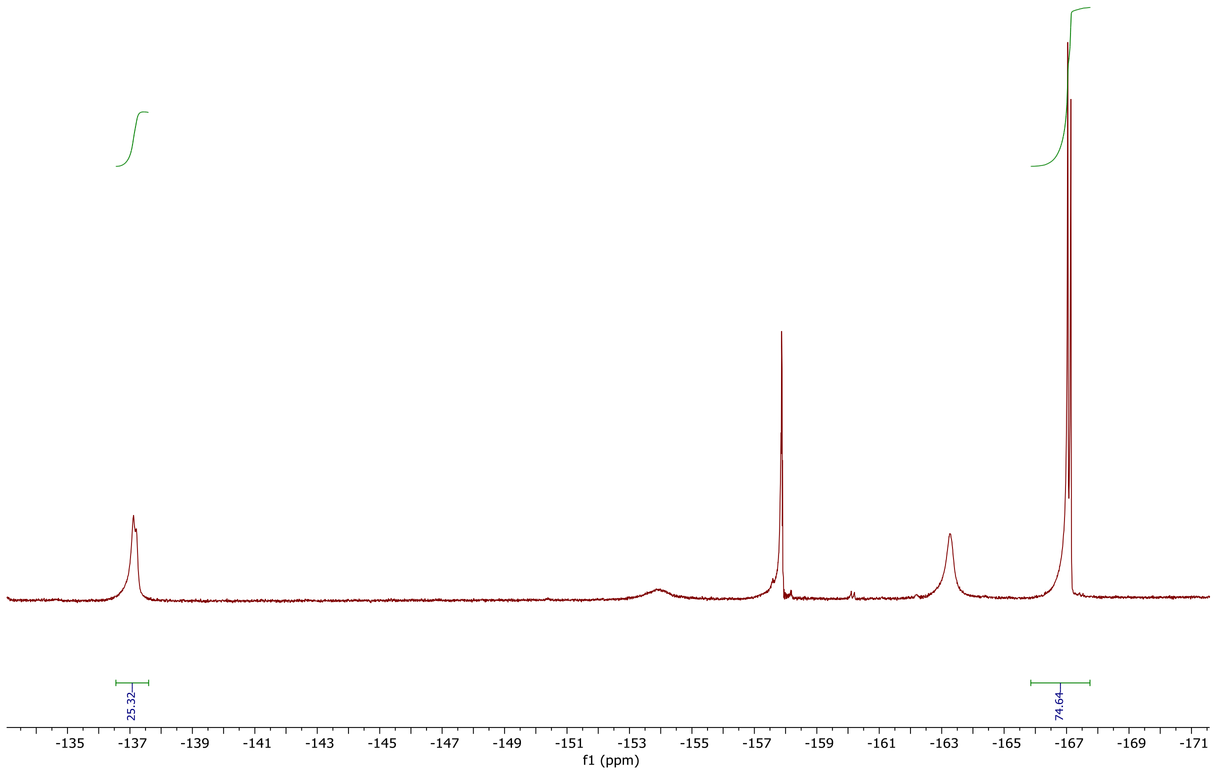

Figure S38. ^{19}F NMR spectrum of crude reaction for **2e-[A]** in DCM-d$_2$ (used for reaction with **N*S*** in Figure S39).

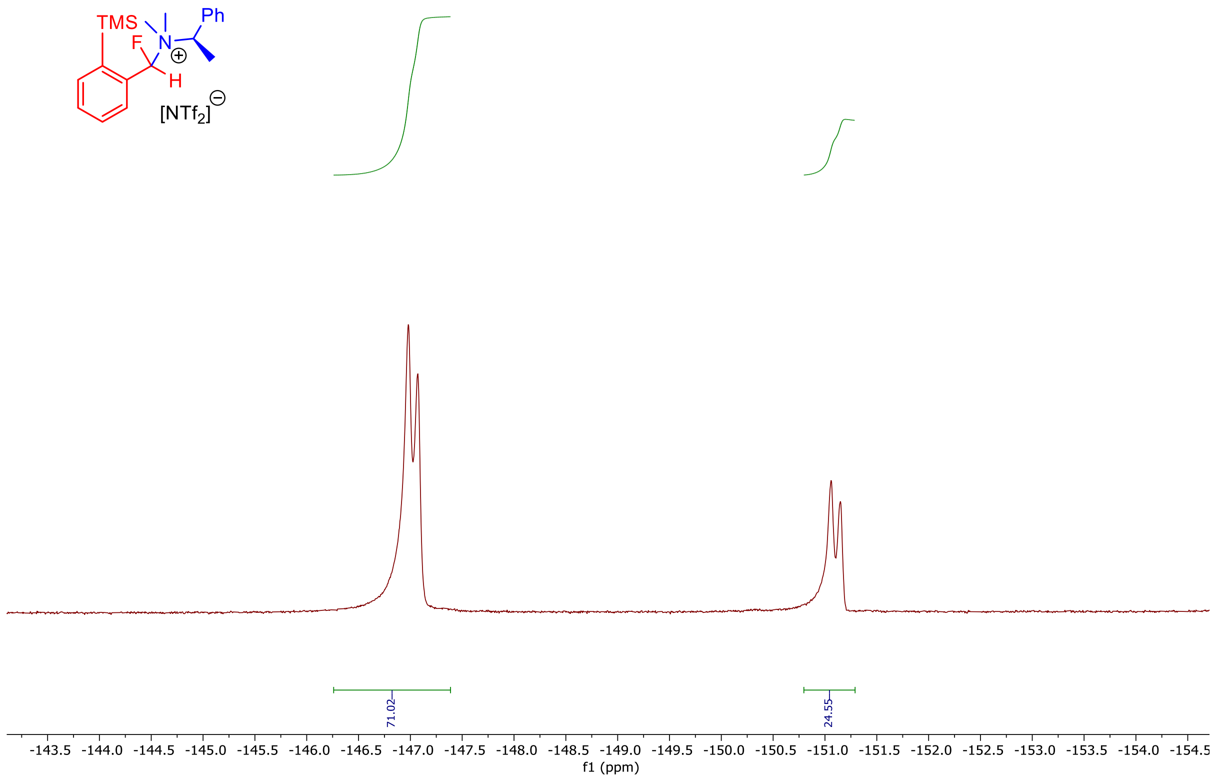

Figure S39. ^{19}F NMR spectrum of crude reaction for **2e-[N*S*]** in DCM-d$_2$ 5 hours after addition of **N*S***.

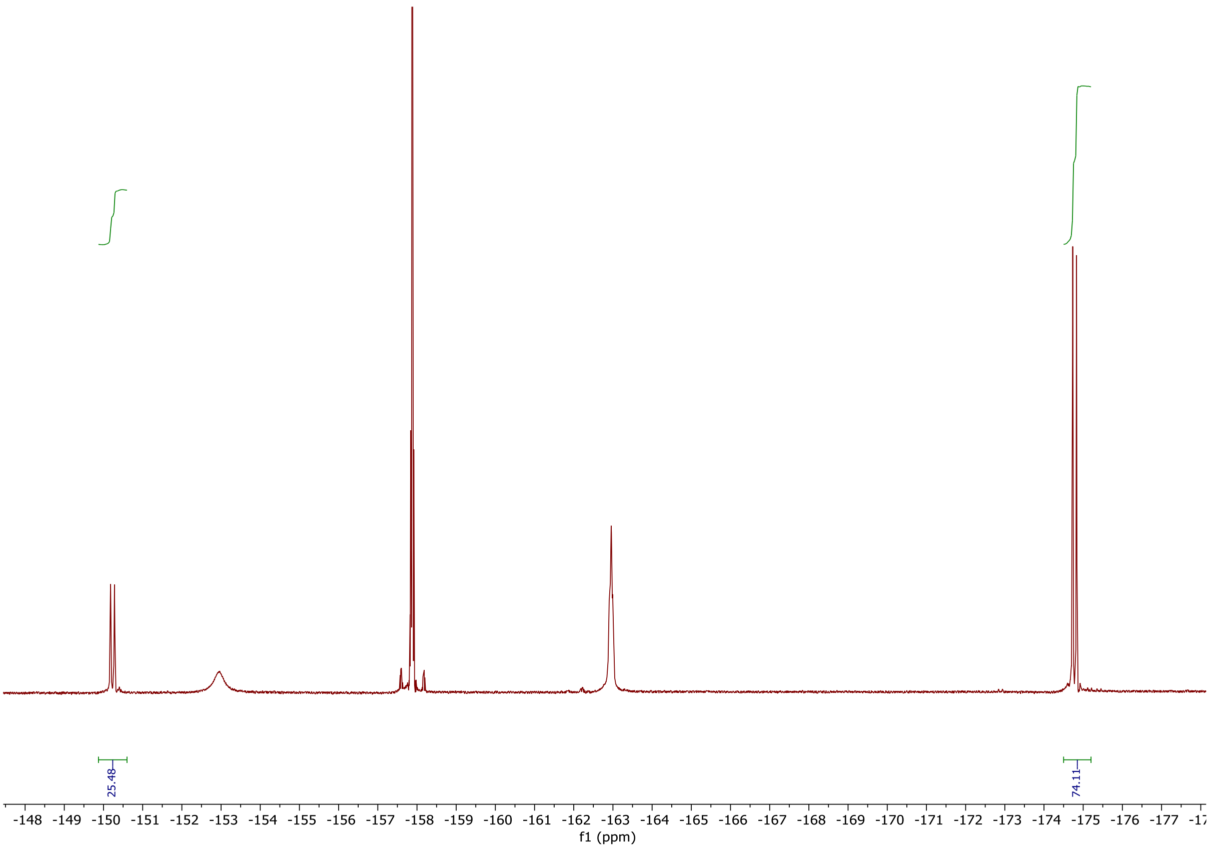

Figure S40. ^{19}F NMR spectrum of crude reaction for **2i-[A]** in DCM-d$_2$ (used for reaction with **N*s*** in Figure S41).

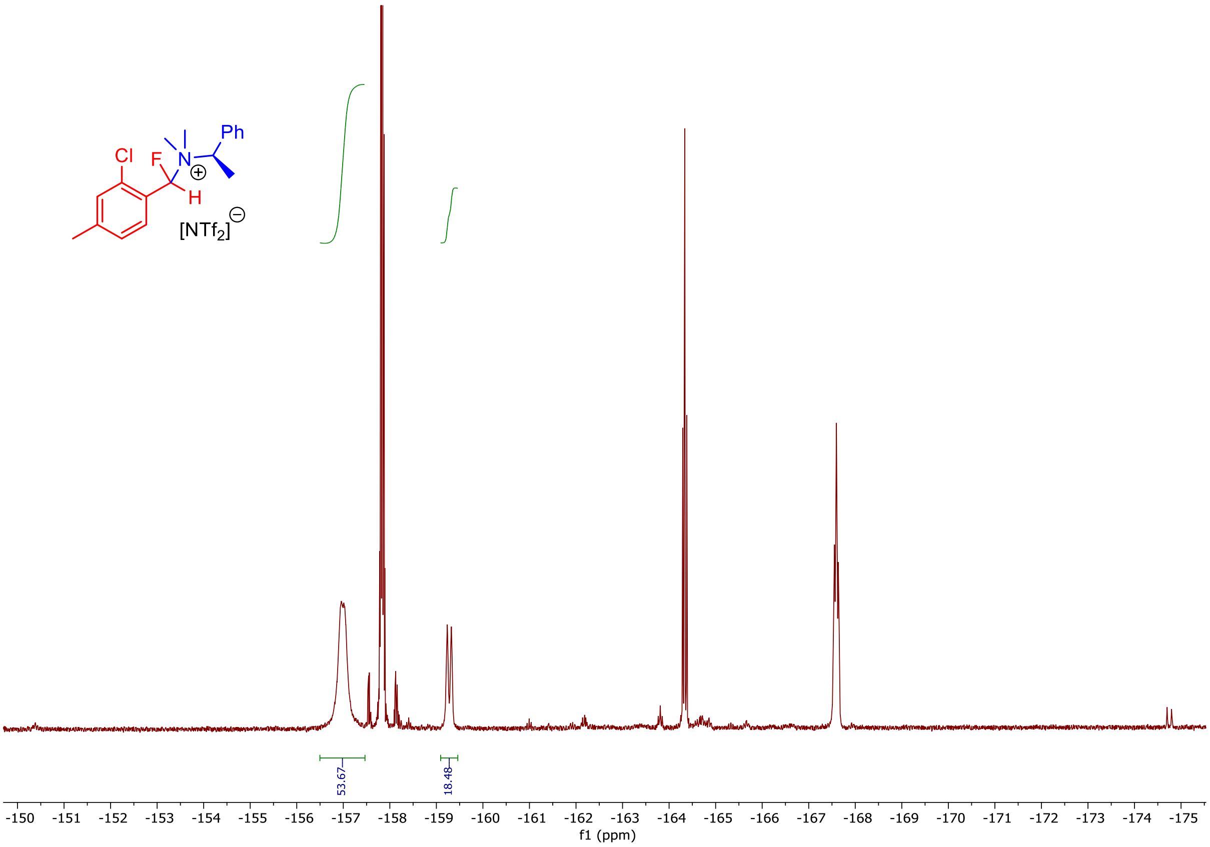

Figure S41. ^{19}F NMR spectrum of crude reaction for **2i-[N*s*]** in DCM-d$_2$ 5 hours after addition of **N*s***.

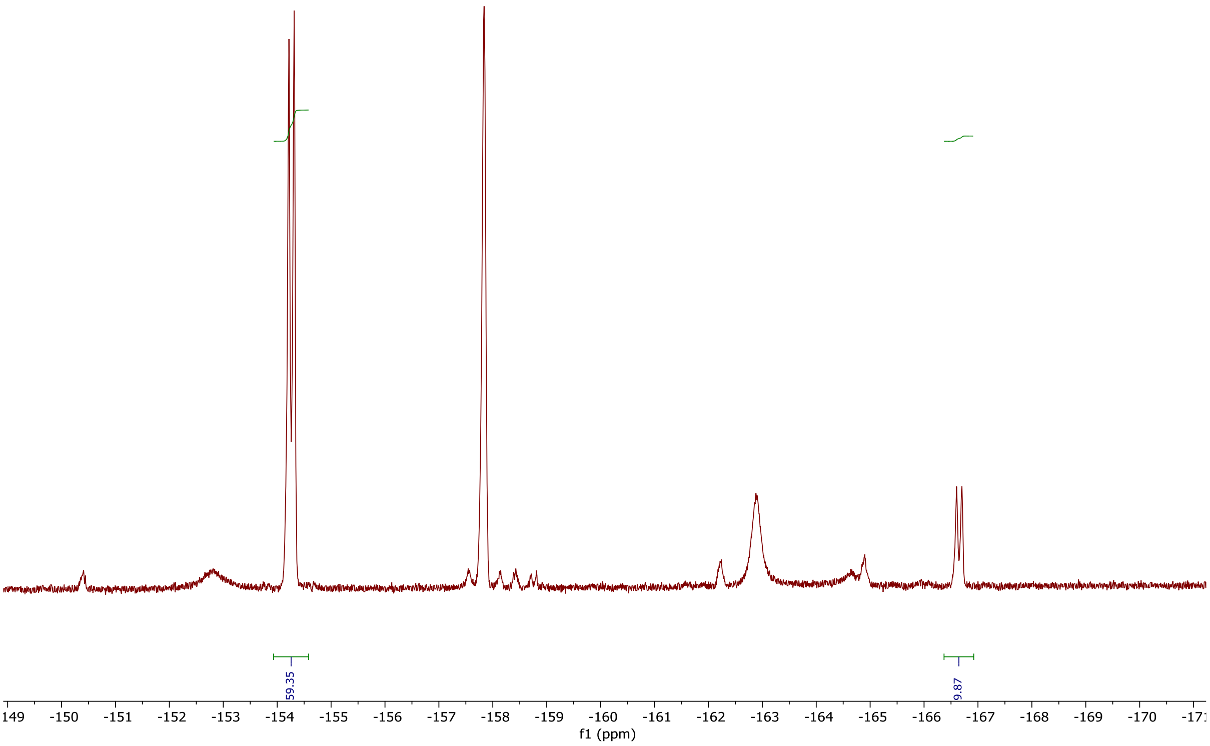

Figure S42. ^{19}F NMR spectrum of crude reaction for **2h-[A]** in DCM-d_2 (used for reaction with **N*S*** in Figure S43).

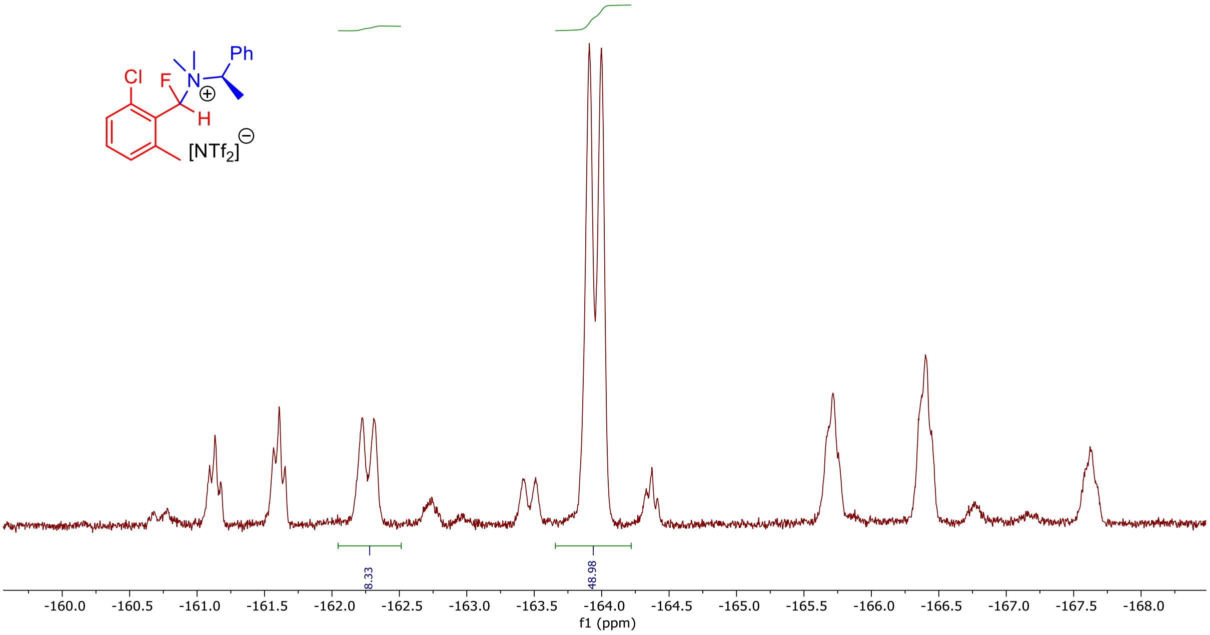

Figure S43. ^{19}F NMR spectrum of crude reaction for **2h-[N*S*]** in DCM-d_2 5 hours after addition of **N*S***. Signals at δ_F 160.7 and 163.4 are suspected conformational isomers for the two diastereomers of **2h-[N*S*]**.

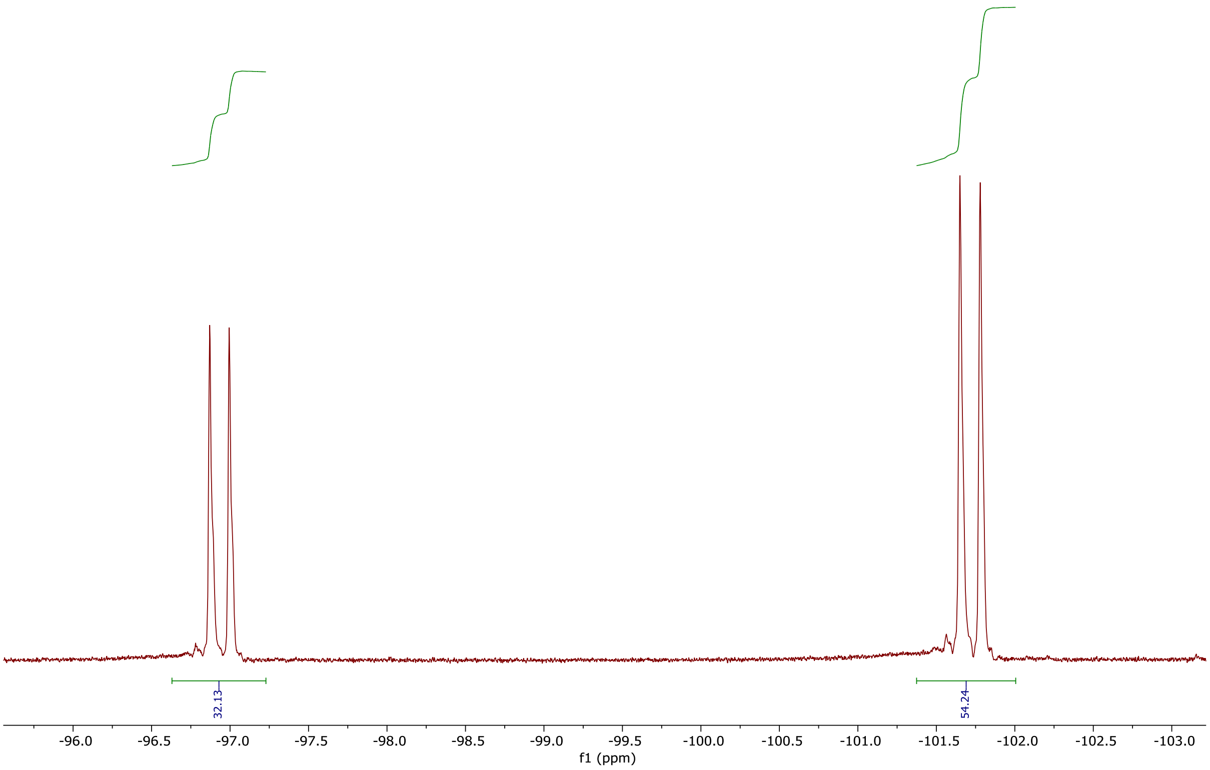

Figure S44. ^{19}F NMR spectrum of crude reaction for **2u-[A]** in DCM-d$_2$ after 12 hours (used for reaction with **N*S*** in Figure S45).

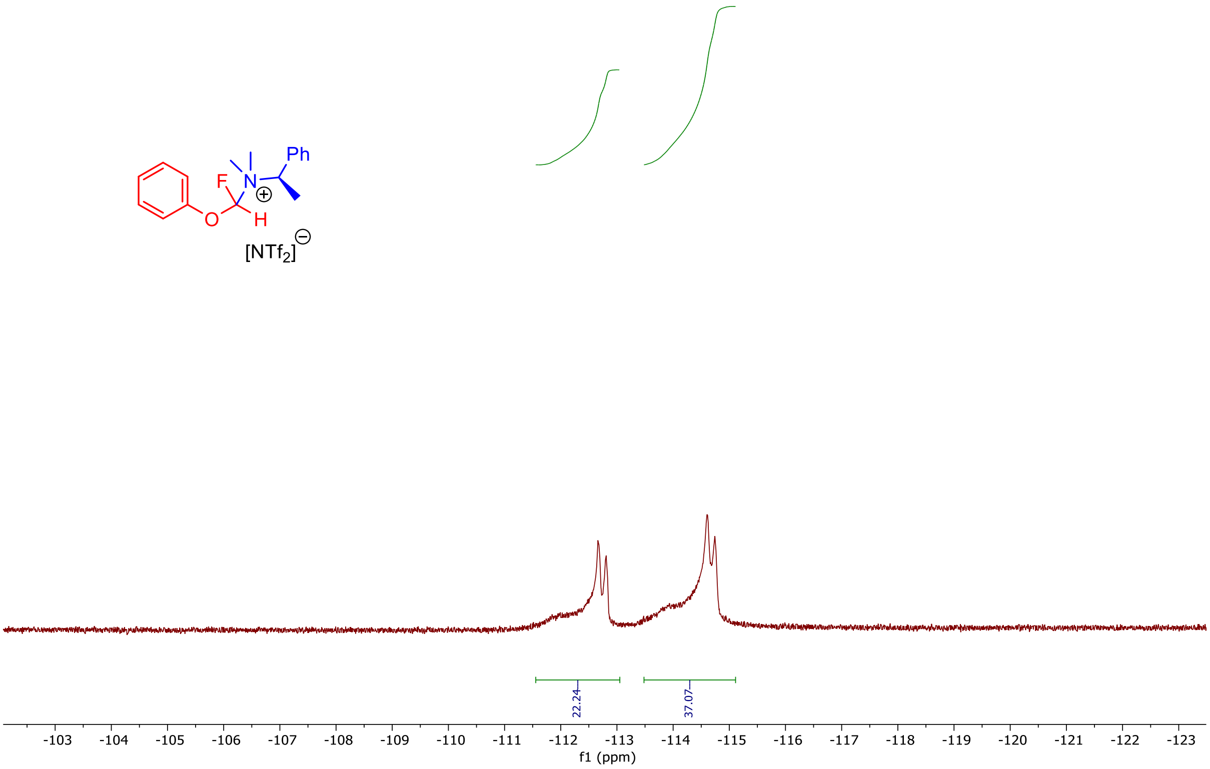

Figure S45. ^{19}F NMR spectrum of crude reaction for **2u-[N*S*]** in DCM-d$_2$ 5 hours after addition of **N*S***.

Synthesis and characterization of enantioenriched neutral compounds 2a-[Nu] (*er* determined by chiral HPLC)

General procedure for synthesis of products 2a-[Nu]

To a mixture of BCF (1.5 mg, 0.003 mmol, 0.05 equiv.), sulfide **A** (8.3 mg, 0.07 mmol, 1.2 equiv.), $TMSNTf_2$ (25.6 mg, 0.07 mmol, 1.2 equiv.) and $PhOCF_3$ (9.7 mg, 0.06 mmol, 1 equiv., IS) in DCM (0.1 mL) in a J. Young's NMR tube was added the difluoride **1a** (0.06 mmol, 12.4 mg, 1.0 equiv.). The reaction was heated at 40 ˚C for 24 hours before ^{19}F NMR analysis was performed to determine NMR yield and selectivity. The nucleophile tetrabutylammonium thiocyanate {[N^nBu_4][SCN]}, tetrabutylammonium benzoate {[N^nBu_4][OBz]} or tetrabutylammonium phthalimide {[N^nBu_4][Pth]} (0.15 mmol, 2.5 equiv.) was added and the reaction left for another 5 hours before NMR analysis to determine NMR yield. The reaction mixture was subject to column chromatography (DCM/hexanes eluent, 1:9) to isolate the product. Enantioselectivity was determined using chiral HPLC.

Compound 2a-[SCN]

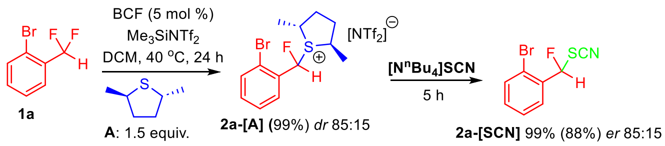

NMR Yield (based on **1a**): 99%. Isolated Yield (based on **1a**): 13 mg 88%, *er* = 85:15.

^{1}H NMR 7.70-7.61 (m, 2 H), 7.48 (td, *J* = 7.8, 0.8 Hz, 1 H), 7.35 (td, *J* = 7.7, 1.6 Hz, 1 H), 7.13 (d, *J* = 49.0 Hz, 1 H); ^{13}C{^{1}H} NMR 133.3 (d, *J* = 0.6 Hz, 1 C), 133.1 (d, *J* = 22.4 Hz, 1 C), 132.1 (d, *J* = 0.3 Hz, 1 C), 128.2 (s, 1 C), 127.4 (d, *J* = 8.5 Hz, 1 C), 120.3 (d, *J* = 4.5 Hz, 1 C), 108.1 (d, *J* = 1.5 Hz, 1 C), 97.5 (d, *J* = 237.4 Hz, 1 C); ^{19}F NMR 151.3 (d, *J* = 49.0 Hz, 1 F); HPLC spectra information: OB-H column, ^{i}PrOH: Hexane 5:95, 254 nm, t_{major} = 24.0 min, t_{minor} = 19.3 min.

Compound 2a-[OBz]

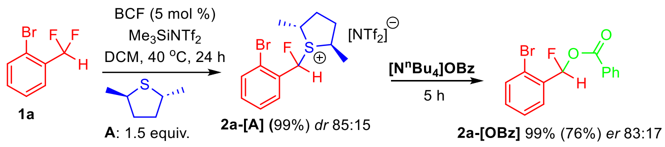

NMR Yield (based on **1a**): 99%. Isolated Yield (based on **1a**): 14 mg 76%, *er* = 83:17.

^{1}H NMR 8.15-8.12 (m, 2 H), 7.83 (dd, *J* = 7.8, 1.7 Hz, 1 H), 7.71 (d, *J* = 54.0 Hz, 1 H), 7.66-7.62 (m, 2 H), 7.52-7.46 (m, 3 H), 7.37 (td, *J* = 7.7, 1.6 Hz, 1 H); ^{13}C{^{1}H} NMR 164.3 (s, 1 C), 134.2 (d, *J* = 20.2 Hz, 1 C), 134.0 (s, 1 C), 133.2 (s, 1 C), 131.6 (s, 1 C), 130.3 (s, 2 C), 128.6 (s, 2 C), 128.5 (s, 1 C), 127.8 (d, *J* = 8.0 Hz, 1 C), 127.7 (s, 1 C), 121.9 (d, *J* = 6.3 Hz, 1 C), 101.2 (d, *J* = 223.3 Hz, 1 C); ^{19}F NMR 129.6 (d, *J* = 54.0 Hz, 1 F); Found: C, 54.9; H, 3.0. Calc. for $C_{14}H_{10}BrFO_2$: C, 54.4; H, 3.3%.; HPLC spectra information: OB-H column, ^{i}PrOH: Hexane 1:99, 254 nm, t_{major} = 20.2 min, t_{minor} = 17.8 min.

Compound 2a-[Pth]

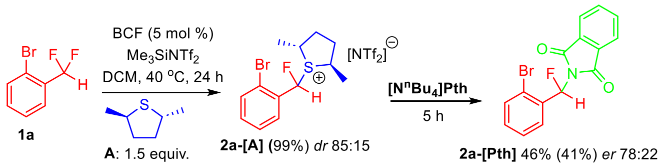

NMR Yield (based on **1a**): 46%. Isolated Yield (based on **1a**): 6 mg 41%, *er* = 78:22.

^{1}H NMR 7.98 (d, *J* = 7.5 Hz, 1 H), 7.90-7.50 (m, 4 H), 7.52 (d, *J* = 7.5 Hz, 1 H), 7.48 (t, *J* = 7.5 Hz, 1 H), 7.30-7.26 (m, 1 H), 7.19 (d, *J* = 47.2 Hz, 1 H); ^{13}C{^{1}H} NMR 165.9 (s, 2 C), 134.8 (s, 2 C), 133.3 (d, *J* = 29.4 Hz, 1 C), 132.6 (s, 1 C), 131.3 (s, 1 C), 130.7 (s, 2 C), 129.3 (d, *J* = 10.8 Hz, 1 C), 127.1 (s, 1 C), 124.0 (s, 2 C), 119.9 (d, *J* = 5.7 Hz, 1 C), 87.6 (d, *J* = 202.9 Hz, 1 C); ^{19}F NMR 153.5 (d, *J* = 47.1 Hz, 1 F); HPLC spectra information: OB-H column, ^{i}PrOH: Hexane 10:90, 254 nm, t_{major} = 22.5 min, t_{minor} = 19.0 min.

Synthesis of compounds 2a-[N3] and 2a-[Trz]

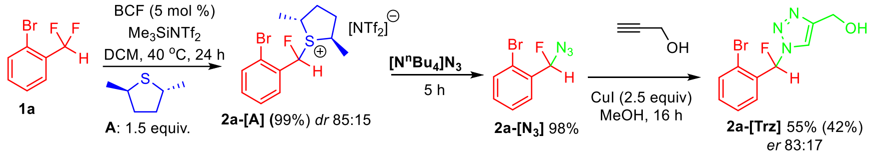

To a mixture of BCF (1.5 mg, 0.003 mmol, 0.05 equiv.), sulfide **A** (8.3 mg, 0.07 mmol, 1.2 equiv.), $TMSNTf_2$ (25.6 mg, 0.07 mmol, 1.2 equiv.) and $PhOCF_3$ (9.7 mg, 0.06 mmol, 1 equiv., IS) in DCM (0.1 mL) in a J. Young's NMR tube was added the difluoride **1a** (0.06 mmol, 12.4 mg, 1.0 equiv.). The reaction was heated at 40 ˚C for 24 hours before ^{19}F NMR analysis was performed to determine NMR yield and selectivity. The nucleophile tetrabutylammonium azide {$[N^nBu_4][N_3]$} (0.15 mmol, 2.5 equiv.) was added and the reaction left for another 5 hours before NMR analysis to determine NMR yield. The solvent was evapourated and copper iodide (2.5 equiv.), propargyl alcohol (2.5 equiv.) and methanol (0.4 mL) were added and the reaction left overnight. NMR analysis was performed to determine the yield before the reaction mixture was subject to column chromatography (dcm/hexanes eluent, 1:9) to isolate the product. Enantioselectivity was determined using chiral HPLC.

NMR Yield (based on **1a**): 54%. Isolated Yield (based on **1a**): 4 mg 41%, *er* = 83:17.

^{1}H NMR 7.9 (d, (d, *J* = 7.8 Hz, 1 H), 7.70 (d, *J* = 49.8 Hz, 7.63 (d, *J* = 7.8 Hz, 1 H), 7.54 (t, *J* = 7.8 Hz, 1 H), 7.40, td, *J* = 7.8, 1.2 Hz, 1 H), 7.38 (s, 1 H), 4.80 (s, 2 H); ^{13}C{^{1}H} NMR 148.0 (s, 1 C), 133.6 (s, 1 C), 132.4 (d, *J* = 26.0 Hz, 1 C), 132.1 (s, 1 C), 128.1 (d, *J* = 1.2 Hz, 1 C), 127.4 (d, 10.3 Hz, 1 C), 121.6 (d, *J* = 5.8 Hz, 1 C), 120.6 (d, *J* = 1.5 Hz, 1 C), 94.3 (d, *J* = 208.8 Hz, 1 C), 56.7 (s, 1 C); ^{19}F NMR 141.7 (d, *J* = 49.6 Hz, 1 F); HPLC spectra information: OB-H column, ^{i}PrOH: Hexane 10:90, 254 nm, t_{major} = 22.5 min, t_{minor} = 19.0 min.

Hydrodebromination of 2a-[OBz] to generate 2b-[OBz]

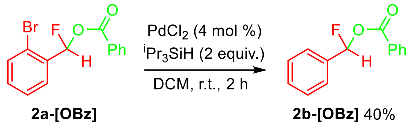

To a vial containing compound **2a-[OBz]** (10.0 mg, 0.03 mmol) was added $PdCl_2$ (0.2 mg, 0.001 mmol, 0.04 equiv.), $^iPr_3SiH$ (9.5 mg, 0.06 mmol, 2.0 equiv.), $PhOCF_3$ (4.8 mg, 0.03 mmol, 1.0 equiv.) and DCM (0.1 mL). The reaction vial was left at room temperature for 2 hours before ^{19}F NMR analysis was performed to determine the reaction yield. The spectroscopic data matched those previously reported for **2b-[OBz]**.[6]

Evidence that *dr* in products 2-[N*S*] does not arise from amine exchange

i. Control reaction with THT

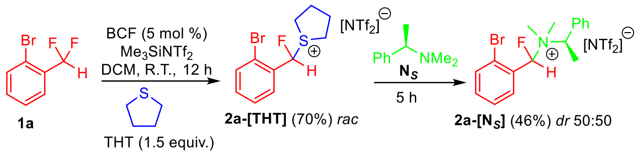

To a mixture of BCF (5.1 mg, 0.01 mmol, 0.05 equiv.), THT (26.5 mg, 0.3 mmol, 1.5 equiv.), TMSNTf$_2$ (106.0 mg, 0.3 mmol, 1.5 equiv.) and PhOCF$_3$ (32.4 mg, 0.2 mmol, 1 equiv., IS) in DCM (0.6 mL) in a J. Young's NMR tube was added the difluoride substrate **1a** (41.4 mg, 0.2 mmol, 1 equiv.). The reaction was left for 12 hours at room temperature before ^{19}F NMR analysis was performed to determine NMR yield. The chiral amine **N*S*** {(*S*)-*N,N*-dimethyl-1-phenylethylamine} (74.5 mg, 0.5 mmol, 2.5 equiv.) was added and the reaction left for another 5 hours before NMR analysis to determine yield and selectivity.

NMR Yield of **2-[THT]** = 70%; NMR Yield of **2a-[N*S*]** (based on **1a**) = 46%, *dr* 50:50.

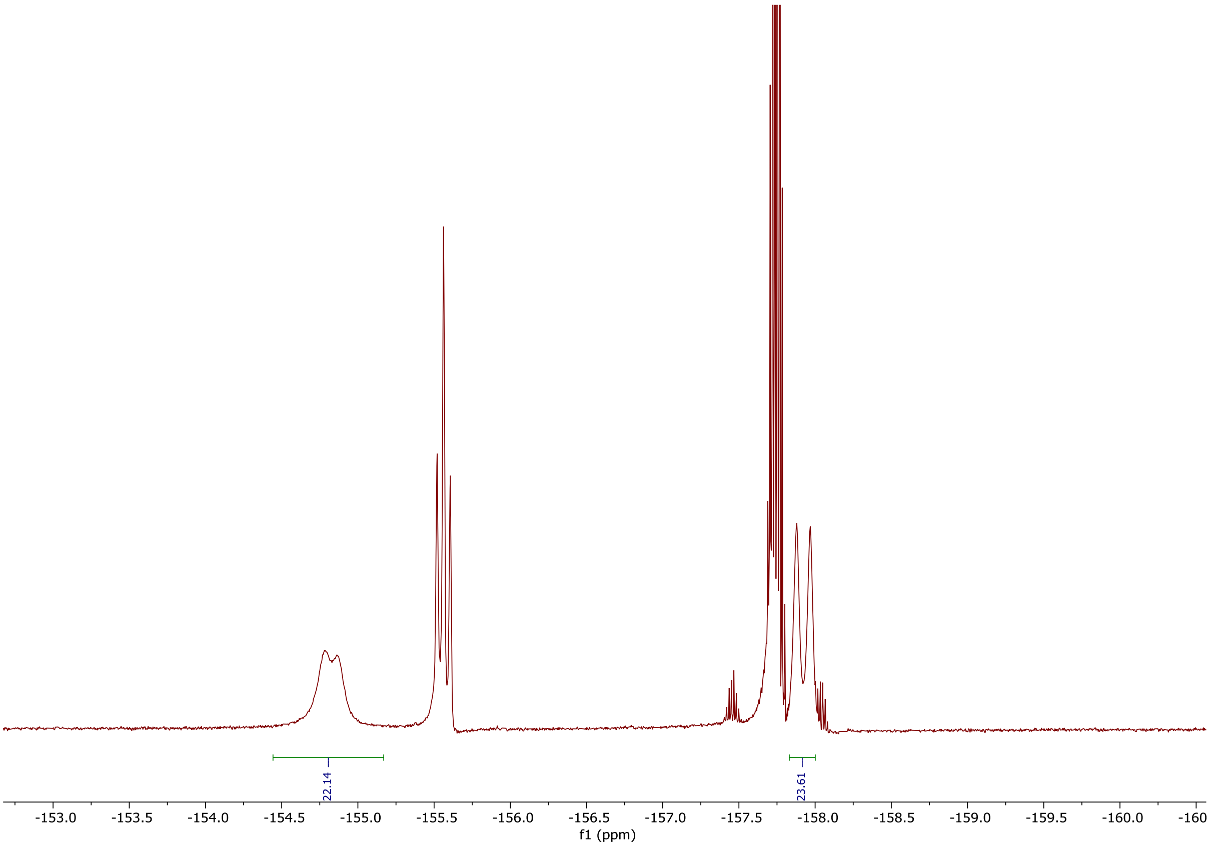

Figure S46. ^{19}F NMR spectrum of crude reaction for **2a-[N*S*]** in DCM-d$_2$ formed from **2a-[THT]**.

ii. Reaction of N*s* with 2a-[A] with different *dr*

A sample of **2a-[A]** taken at 2 hours reaction time (*dr* of 60:40, Figure S47) was reacted with **Ns** to give **2a-[N*s*]** with a *dr* of 60:40 (Figure S48), confirming that substitution of **A** with **N*s*** arrests the equilibrium and fixes the *dr*.

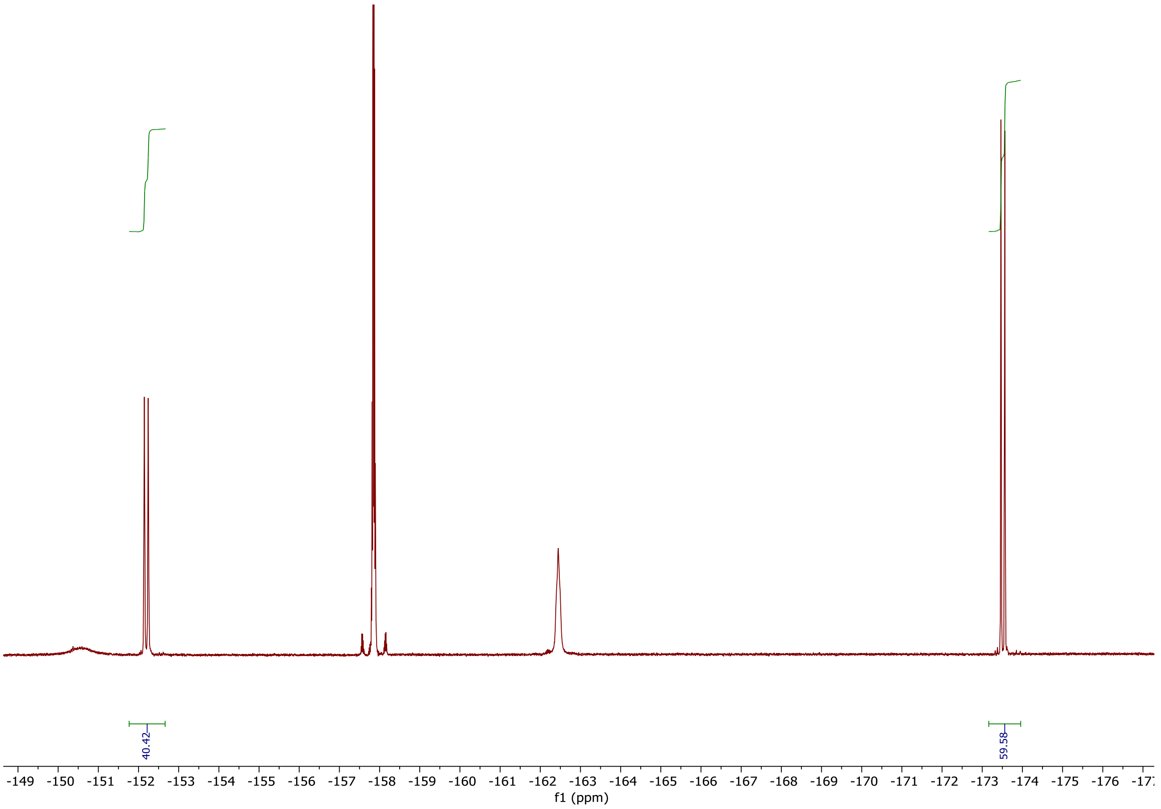

Figure S47. ^{19}F NMR spectrum of crude reaction for **2a-[A]** in DCM-d$_2$ after 2 hours (used for reaction with **N*s*** in Figure S46).

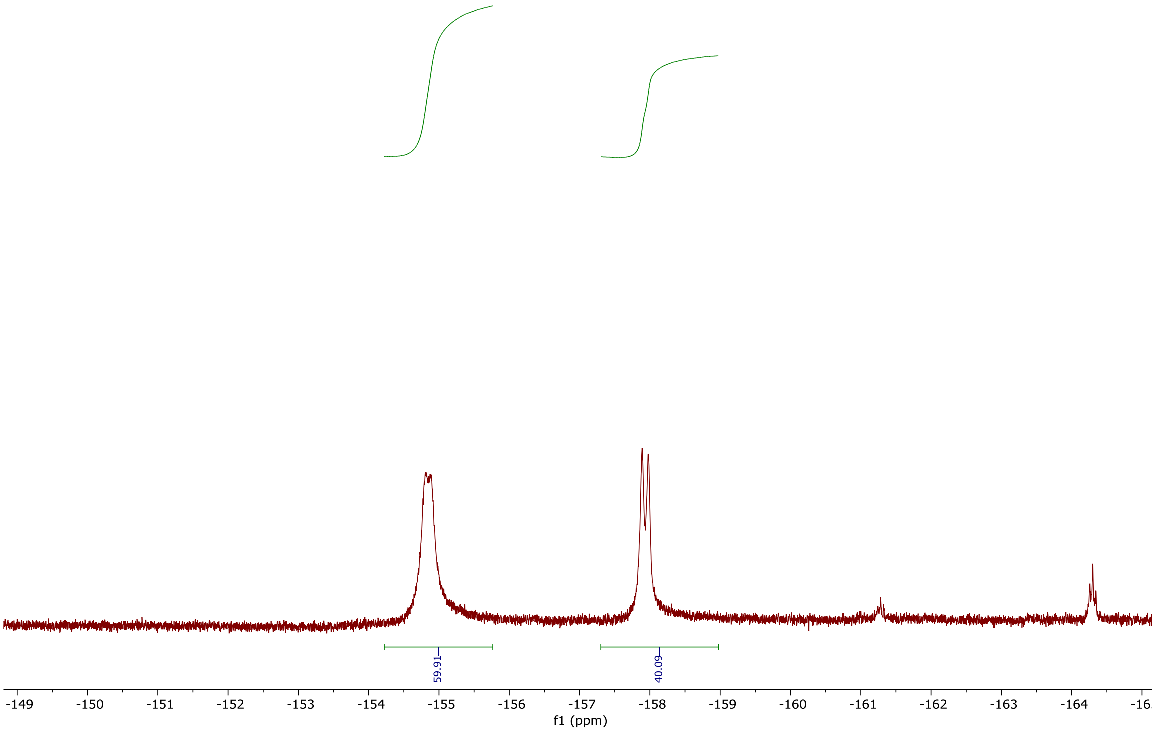

Figure S48. ^{19}F NMR spectrum of reaction for **2a-[N*s*]** in DCM-d$_2$ 5 hours after addition of **N*s***. Reaction was filtered and evapourated to dryness before redissolution due to overlap with TMSF.

iii. **Attempted reaction of 2a-[N*S*] with NEt₃**

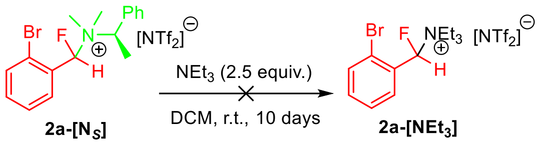

To a sample of **2a-[N*S*]** (0.06 mmol) in DCM (0.5 mL) was added triethyl amine (15 mg, 0.15 mmol, 2.5 equiv.). The reaction was left to stir for 10 days at room temperature, after which time no evidence of **2a-[NEt$_3$]** was observed.

iv. **Calculated barrier for exchange of N*S***

The exchange of **N*S*** groups was calculated to be kinetically unfeasible at over 33 kcal mol^{-1} (Figure S148-S149).

Monitoring *dr* of 2a-[A] after completion of C–F activation reaction

To a mixture of BCF (2.6 mg, 0.005 mmol, 0.05 equiv.), sulfide **A** (17.4 mg, 0.15 mmol, 1.5 equiv.), $TMSNTf_2$ (53.0 mg, 0.15 mmol, 1.5 equiv.) and $PhOCF_3$ (16.2 mg, 0.1 mmol, 1 equiv., IS) in DCM (0.3 mL) in a J. Young's NMR tube was added the difluoride **1a** (20.6 mg, 0.1 mmol, 1 equiv.). The reaction was monitored via ^{19}F NMR analysis over the period of 6 days. After 48 hours, additional sulfide **A** was added to the reaction (29 mg, 0.25 mmol, 2.5 equiv.).

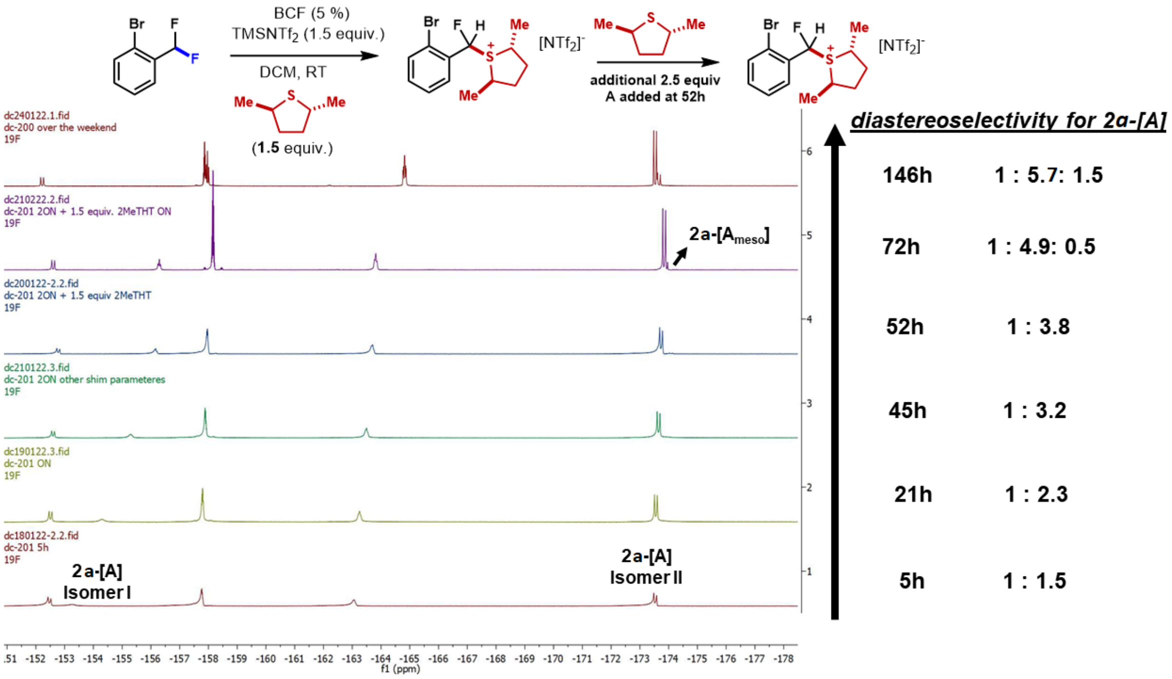

Figure S49. Stacked ^{19}F NMR spectra of reaction mixture of **2a-[A]** in DCM-d_2 at different time intervals. Additional **A** was added after 48 hours.

Chart S1. *dr* ratio of **2a-[A]** over time after reaction completion.

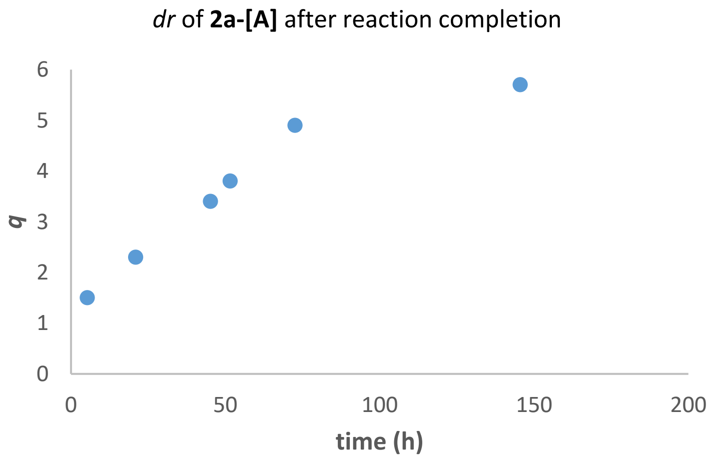

Hours after reaction started	*q*
5	1.5
21	2.3
45	3.4
52	3.8
72	4.9
144	5.7

Reaction of 2a-[THT] with A

To a mixture of BCF (2.6 mg, 0.005 mmol, 0.05 equiv.), THT (13.3 mg, 0.15 mmol, 1.5 equiv.), $TMSNTf_2$ (53.0 mg, 0.15 mmol, 1.5 equiv.) and $PhOCF_3$ (16.2 mg, 0.1 mmol, 1 equiv., IS) in DCM (0.3 mL) in a J. Young's NMR tube was added the difluoride **1a** (20.6 mg, 0.1 mmol, 1 equiv.). After 12 h sulfide **A** was added to the reaction (29 mg, 0.25 mmol, 2.5 equiv.) and the reaction was monitored via ^{19}F NMR analysis over the period of 3 days.

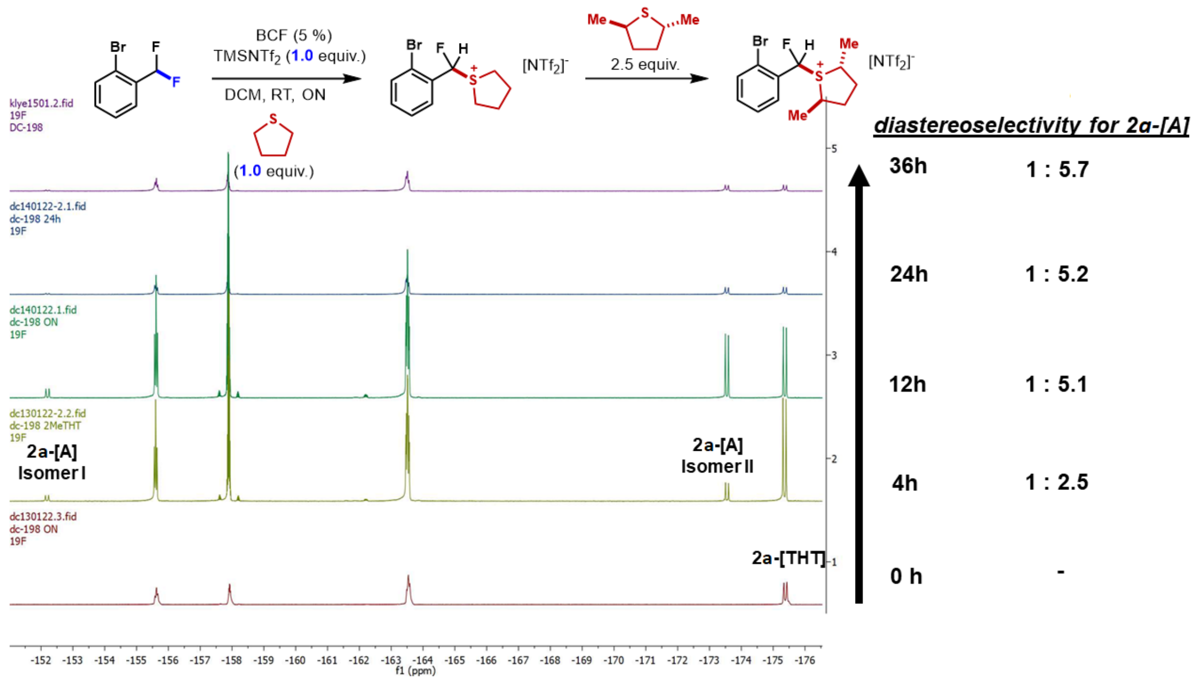

Figure S50. Stacked ^{19}F NMR spectra of reaction mixture between **2a-[THT]** and **A** in DCM-d_2 at different time intervals.

Attempts to isomerize free A to $\mathbf{A_{meso}}$

Reaction A: A mixture of **A** (11.6 mg, 0.1 mmol) and BCF (5.1 mg, 0.1 equiv.) were dissolved in DCM-d_2 (0.4 mL). The mixture was monitored via ^1H and ^{13}C NMR spectroscopies over 7 days, after which time no evidence of $\mathbf{A_{meso}}$ was observed.

Reaction B: A mixture of **A** (11.6 mg, 0.1 mmol) and BCF (51.3 mg, 1.0 equiv.) were dissolved in DCM-d_2 (0.4 mL). The mixture was monitored via ^1H and ^{13}C NMR spectroscopies over 7 days, after which time no evidence of $\mathbf{A_{meso}}$ was observed.

Reaction C: A mixture of **A** (11.6 mg, 0.1 mmol) and Me_3SiNTf_2 (35 mg, 1.0 equiv.) were dissolved in DCM-d_2 (0.4 mL). The mixture was monitored via ^1H and ^{13}C NMR spectroscopies over 7 days, after which time no evidence of $\mathbf{A_{meso}}$ was observed.

Crystallographic details

X-Ray diffraction analysis was performed by the XRAY department at NUS. The X-ray intensity data were measured on a Bruker D8 Venture dual source diffractometer. The crystal structures were solved by direct methods using SHELXS-97 and refined with SHELXL-2014 using Olex2. Crystals suitable for X-ray diffraction were grown from saturated solutions of DCM layered with *n*-hexane. Structures for compounds **2h-[B]**, **2x-[B]** and **2a-[Trz]** can be found at ccdc.cam.ac.uk under CCDC numbers 2226095, 2226096 and 2271389 respectively.

Data	2h-[B]	2x-[B]	2a-[Trz]
**Formula**	$C_{18}H_{23}ClF_7NO_4S_3$	$C_{20}H_{28}F_7NO_4S_3$	$C_{10}H_9BrFN_3O$
**Formula weight**	582.00	575.61	286.11
**Colour**	Colourless	Colourless	Colourless
**Crystal size / mm³**	0.385 × 0.191 × 0.132	0.191 × 0.107 × 0.055	0.169 x 0.166 x 0.151
**Temperature / K**	100	100	100
**Crystal system**	triclinic	triclinic	Orthorhombic
**Space group**	P-1	P-1	P_{21}
**a / Å**	8.9260(3)	9.8683(3)	5.7873(10)
**b / Å**	9.9926(4)	11.3605(3)	10.1017(16)
**c / Å**	14.7271(6)	13.8350(4)	18.377(3)
**α / °**	100.080(2)	97.5980(10)	90
**β / °**	92.075(2)	91.0450(10)	90
**γ / °**	110.953(2)	112.3920(10)	90
**V / Å³**	1200.78(8)	1417.50(7)	1074.3(3)
**Z**	2	2	4
ρ_{calcd} **/ g cm⁻³**	1.610	1.349	1.769
**Radiation used**	Mo-Kα	Mo-Kα	Mo-Kα
μ **/ mm⁻¹**	0.501	0.332	3.821
2θ ***max*** **/ °**	50.052	50.484	50.484
**No. of unique reflns**	4230	7340	2985
**No. of variables**	309	320	149
**GoF (S)**	1.050	1.037	1.056
**R factor (I > 2σ)**	0.0634 (4038 reflections)	0.0459 (5701 reflections)	0.0182 (2918 refelctions)
**Flack parameter (x)**	-	-	-0.012(4)

Table S1. Crystal Data, Data Collection and Refinement Parameters for the structure of **2h-[B]**, **2x-[B]** and **2a-[Trz]**.

Characterisation data

NMR spectra for isolated compounds

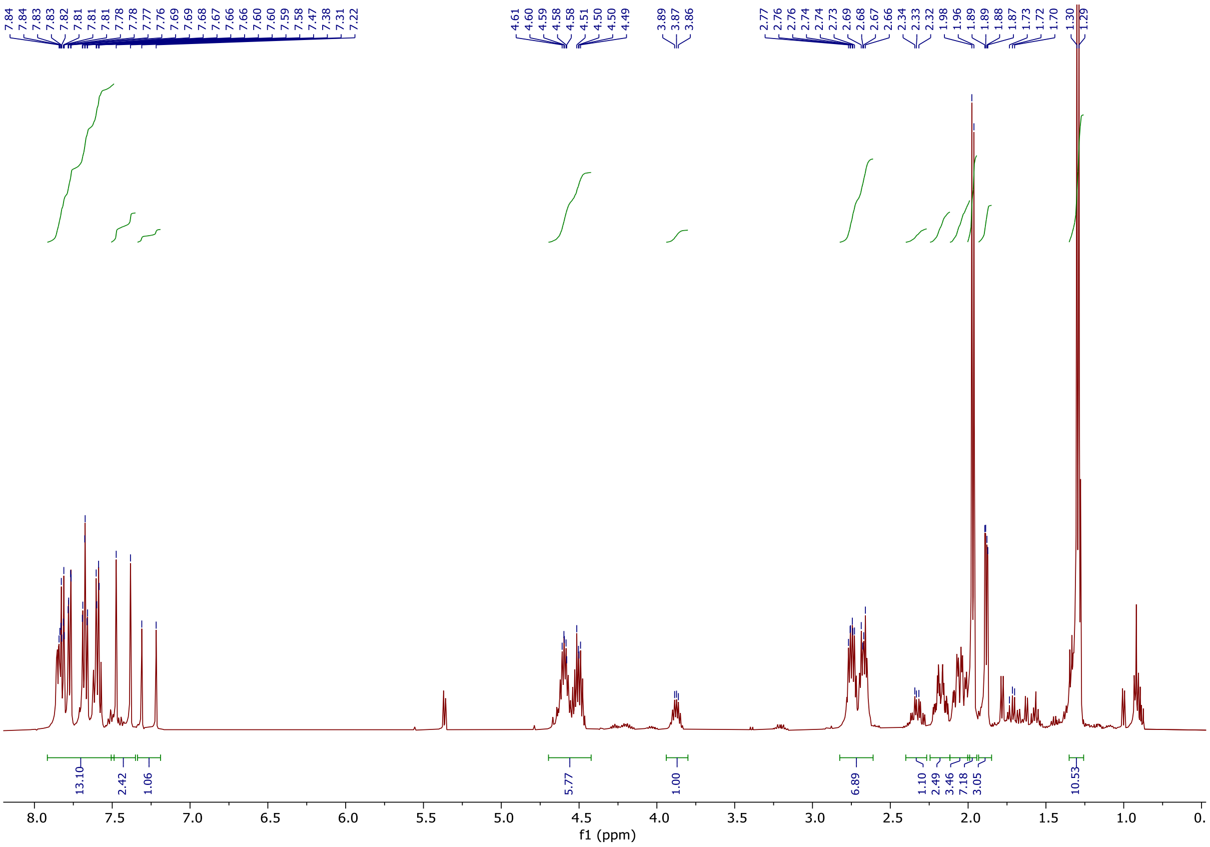

Figure S51. ^{1}H NMR spectrum of isolated **2a-[A]** in DCM.

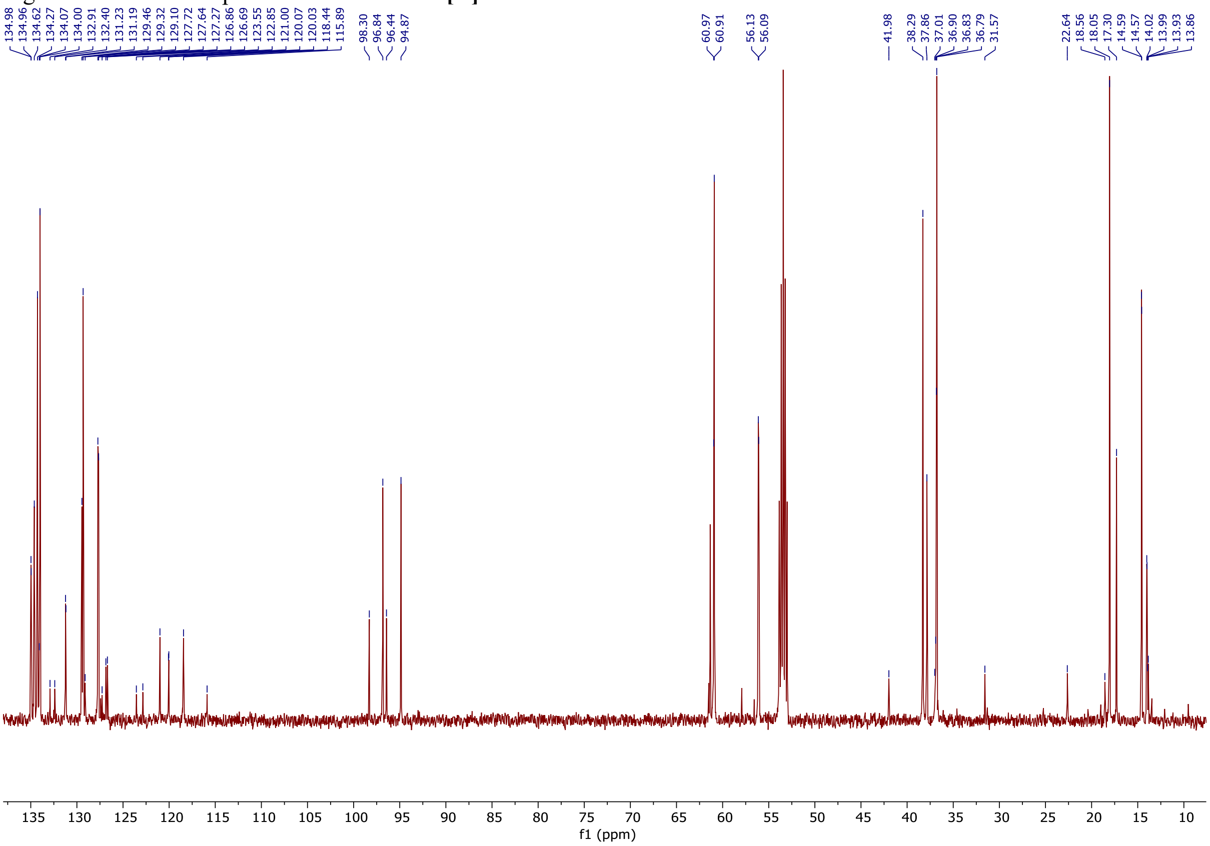

Figure S52. $^{13}C\{^{1}H\}$ NMR spectrum of isolated **2a-[A]** in DCM-d_2.

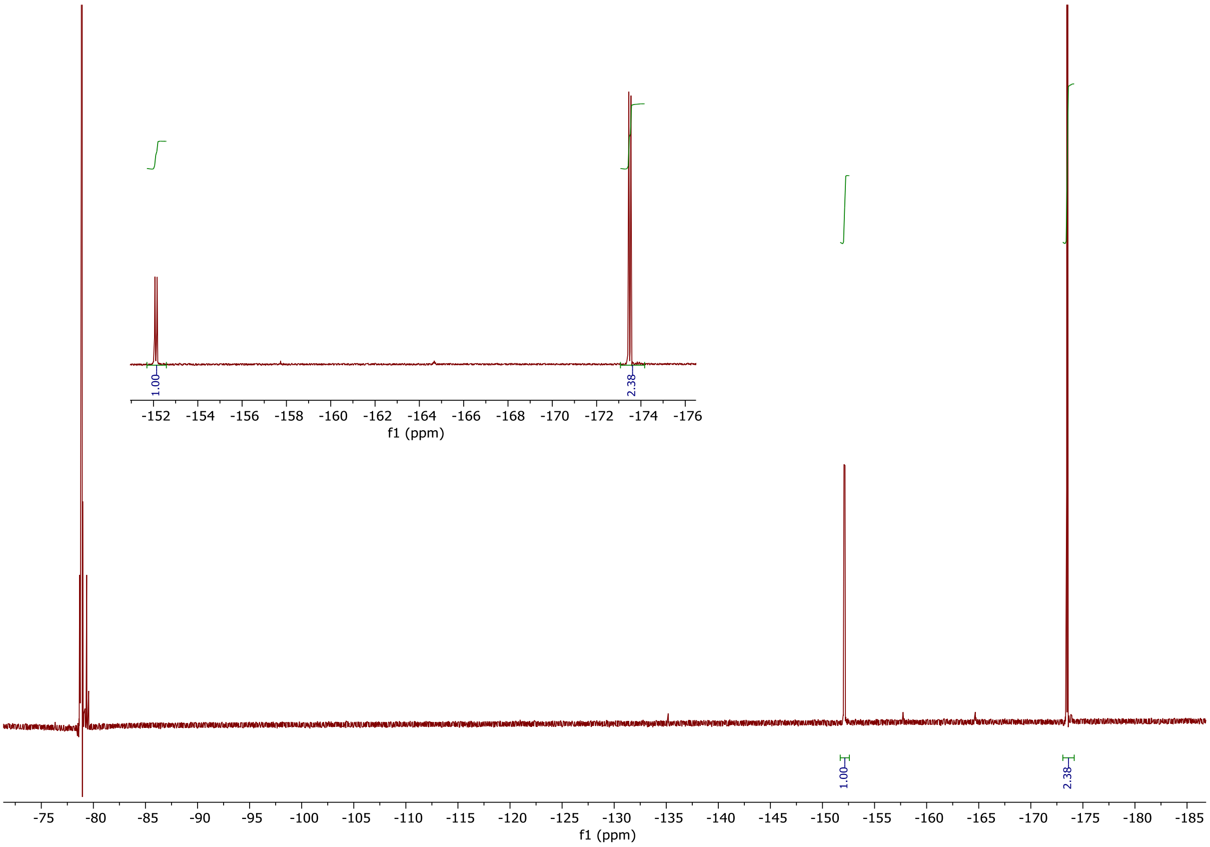

Figure S53. ^{19}F NMR spectrum of isolated **2a-[A]** in DCM-d$_2$ (immediately after redissolution).

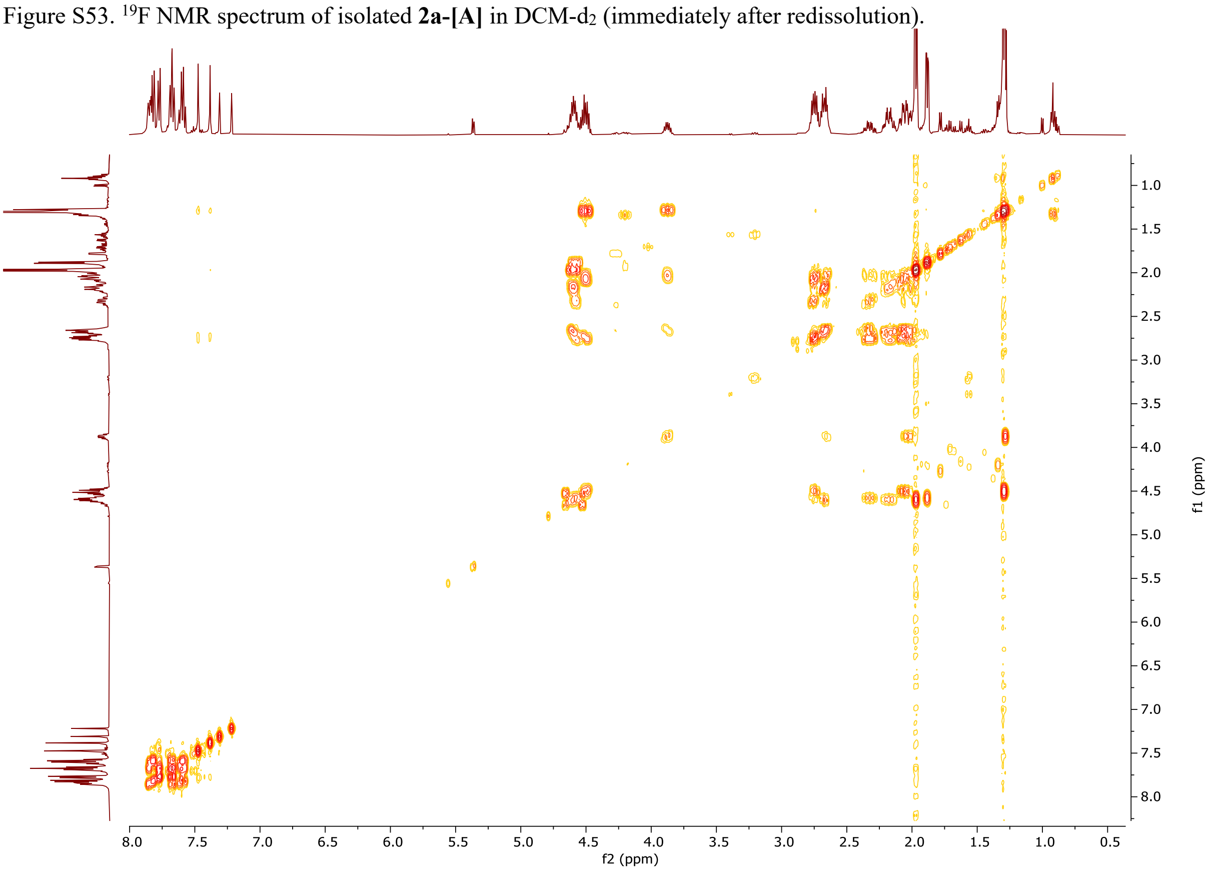

Figure S54. ^{1}H-^{1}H COSY NMR spectrum of isolated **2a-[A]** in DCM-d$_2$.

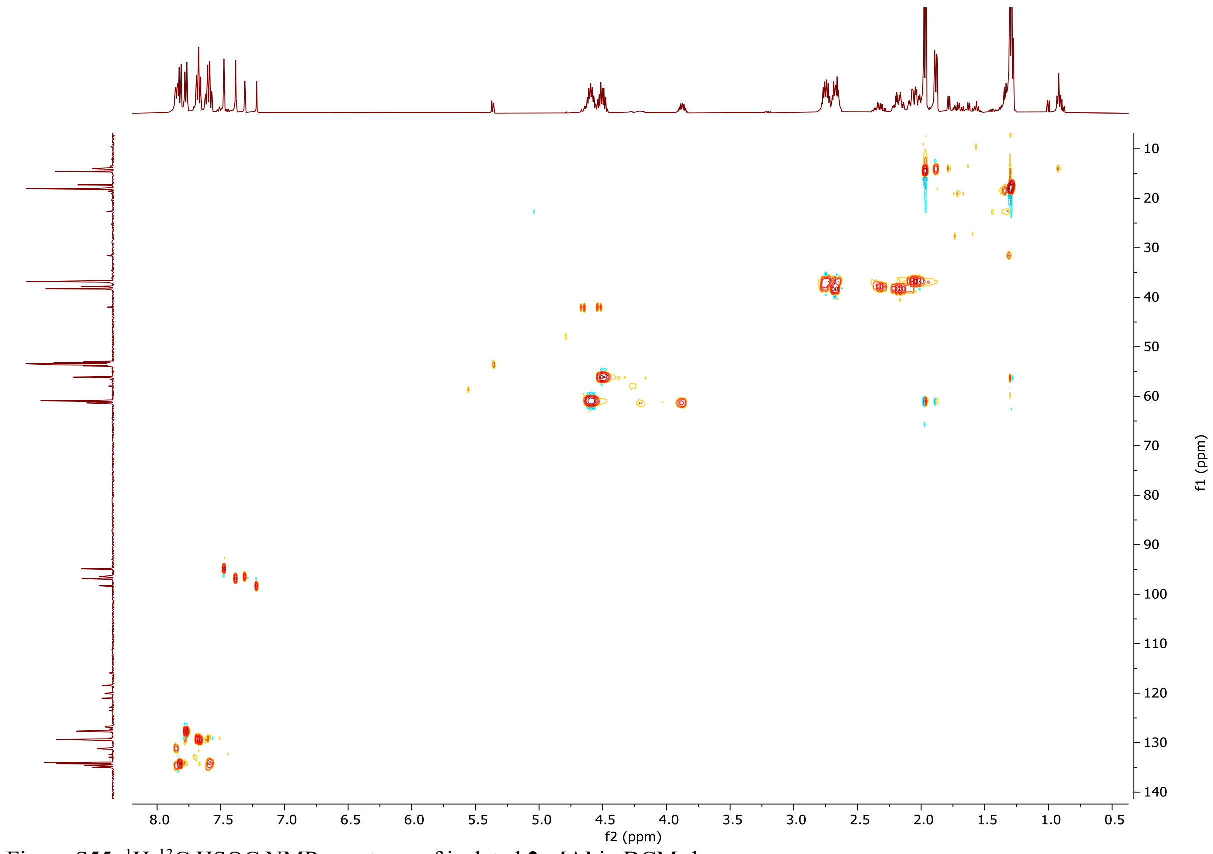

Figure S55. ^{1}H-^{13}C HSQC NMR spectrum of isolated **2a-[A]** in DCM-d_2.

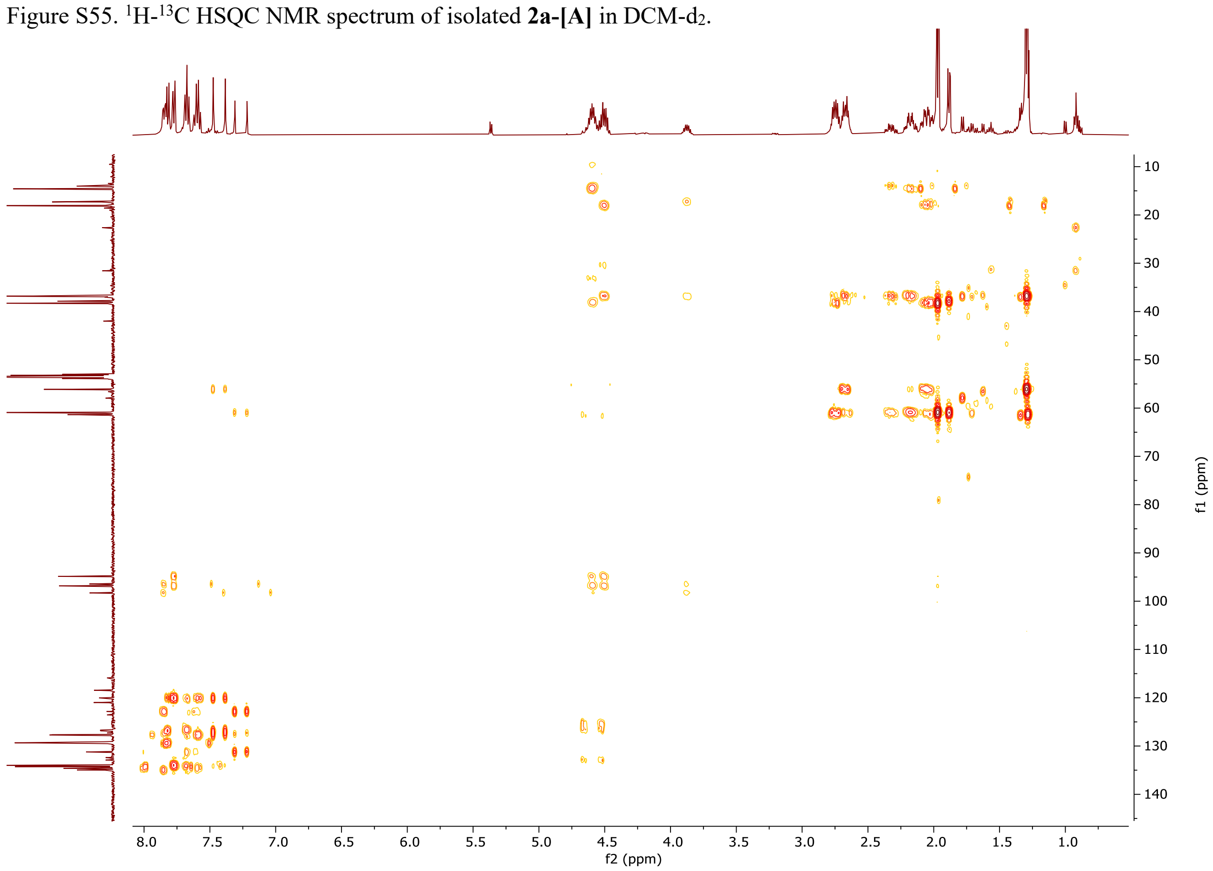

Figure S56. ^{1}H-^{13}C HMBC NMR spectrum of isolated **2a-[A]** in DCM-d_2.

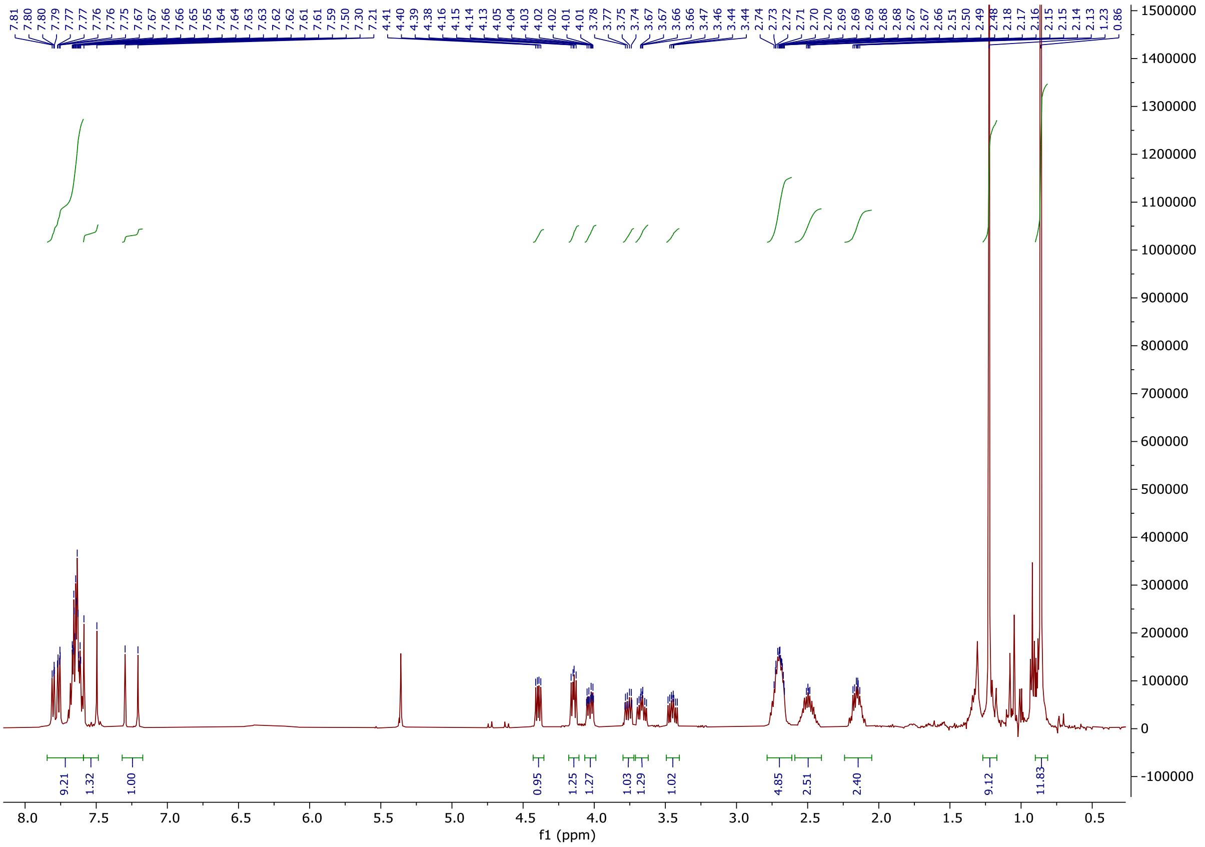

Figure S57. ^{1}H NMR spectrum of isolated **2c-[B]** in DCM-d2.

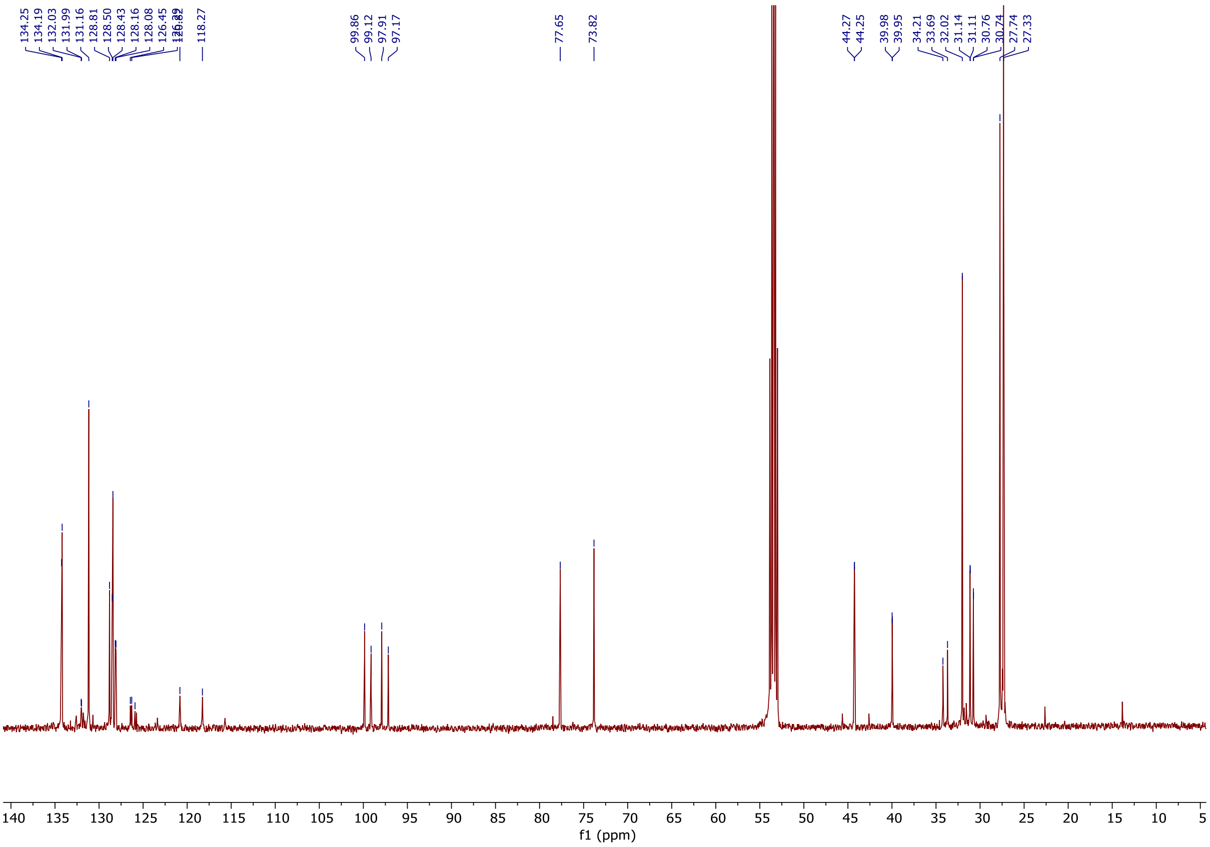

Figure S58. ^{13}C NMR spectrum of isolated **2c-[B]** in DCM-d2.

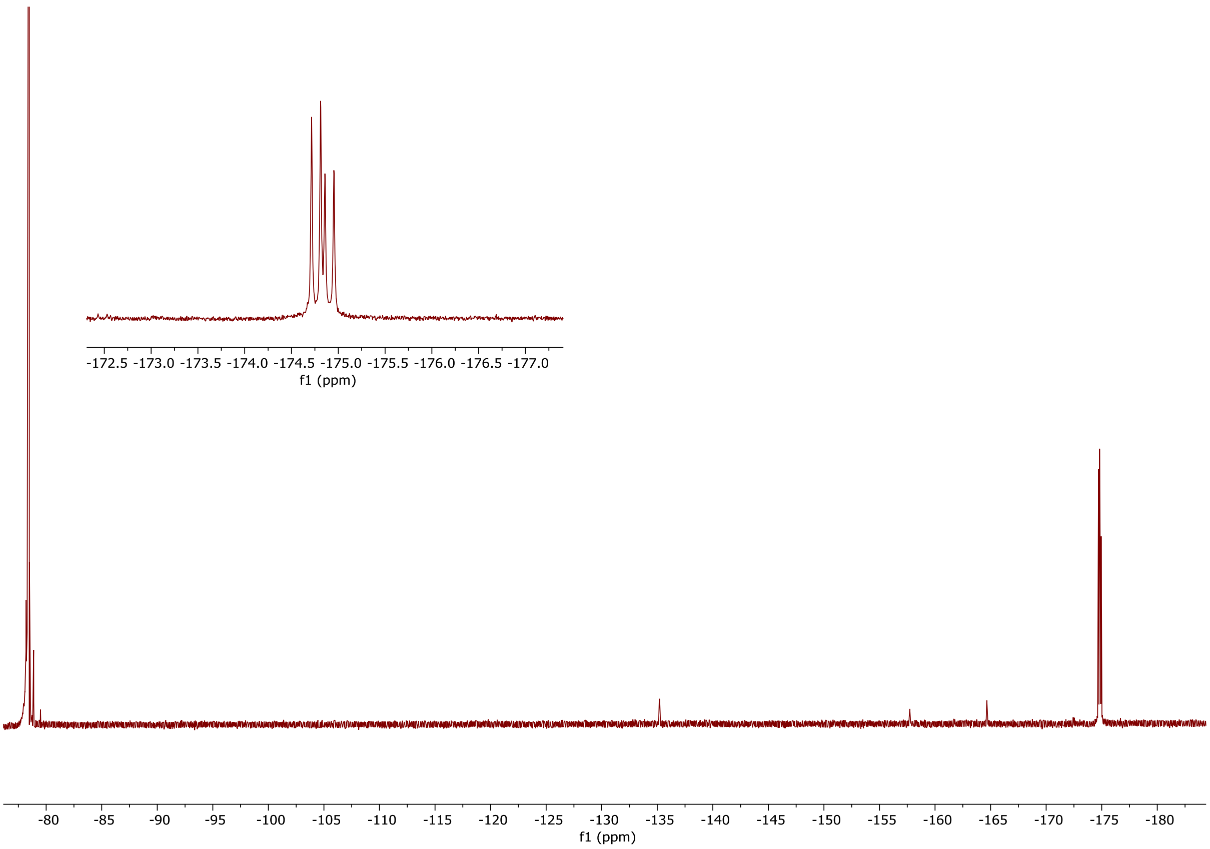

Figure S59. ^{19}F NMR spectrum of isolated **2c-[B]** in DCM-d2. Minor signals at 135, 157 and 165 ppm due to $[B(OH)(C_6F_5)_3]^-$ or $[(OH)\{B(C_6F_5)_3\}_2]^-$ anions.

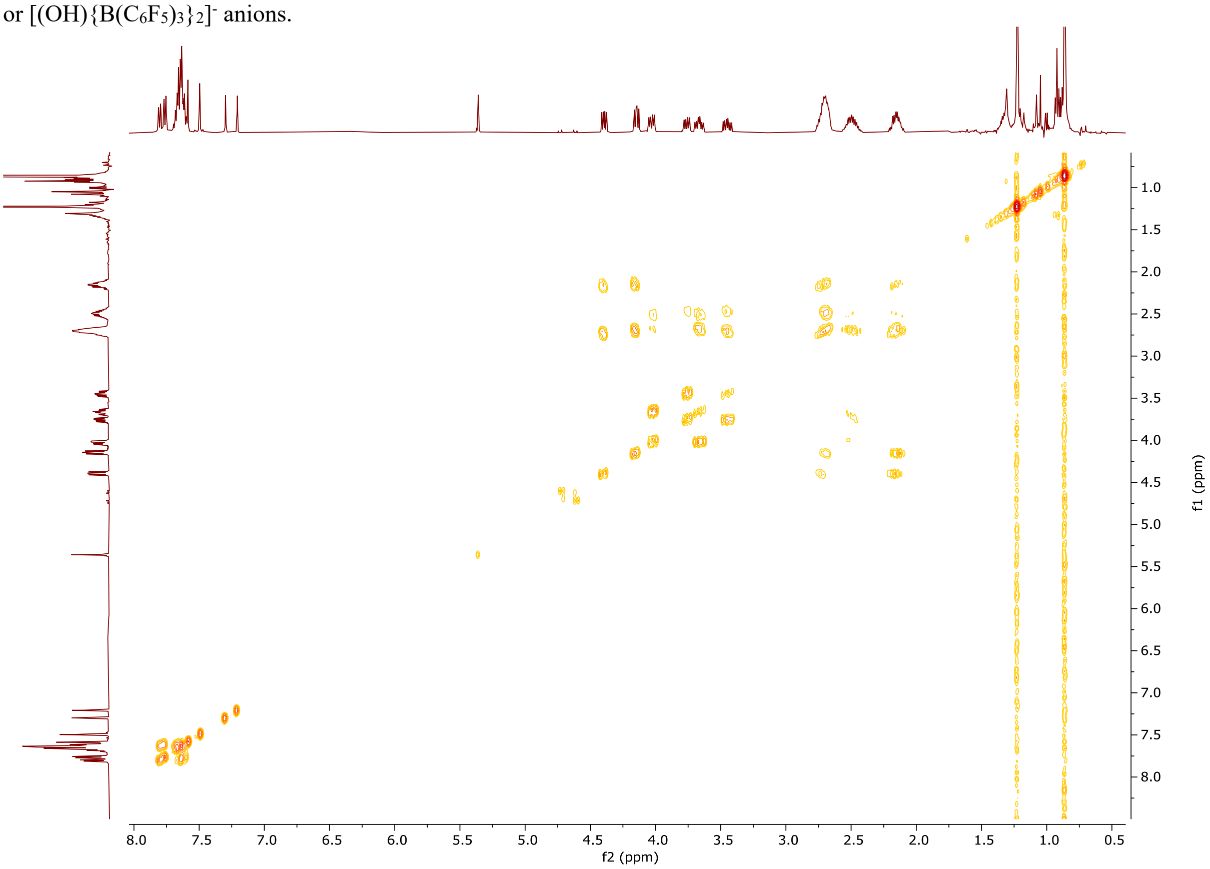

Figure S60. ^{1}H-^{1}H COSY NMR spectrum of isolated **2c-[B]** in DCM-d2.

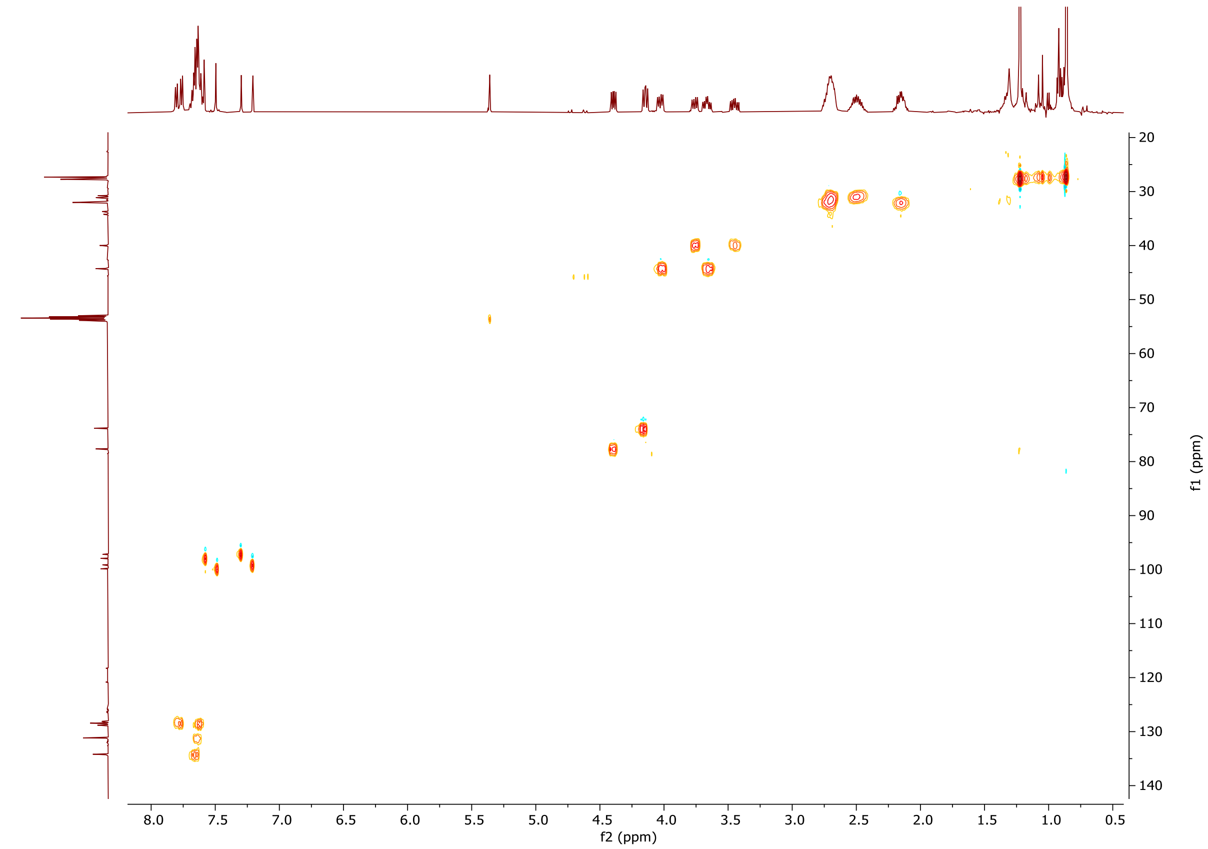

Figure S61. ^{1}H-^{13}C HSQC NMR spectrum of isolated **2c-[B]** in DCM-d_2.

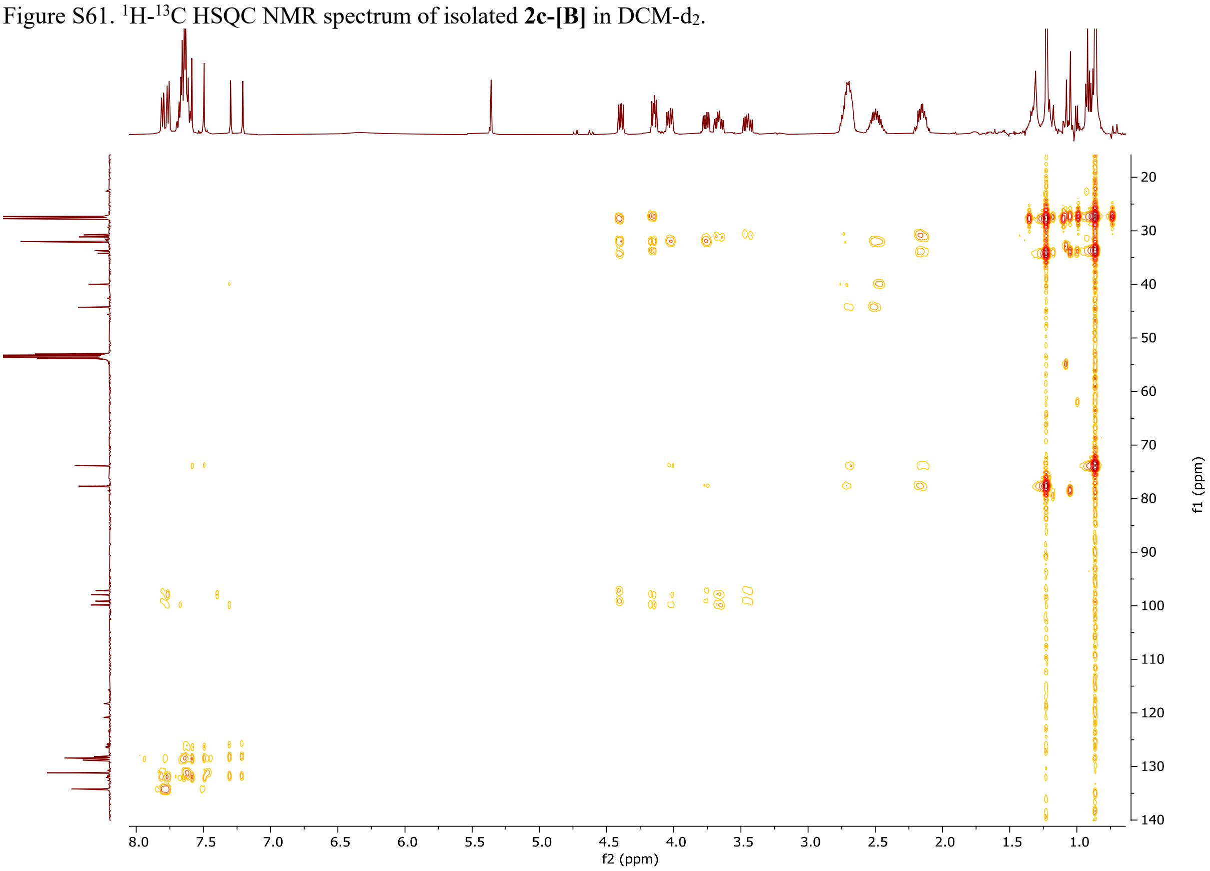

Figure S62. ^{1}H-^{13}C HMBC NMR spectrum of isolated **2c-[B]** in DCM-d_2.

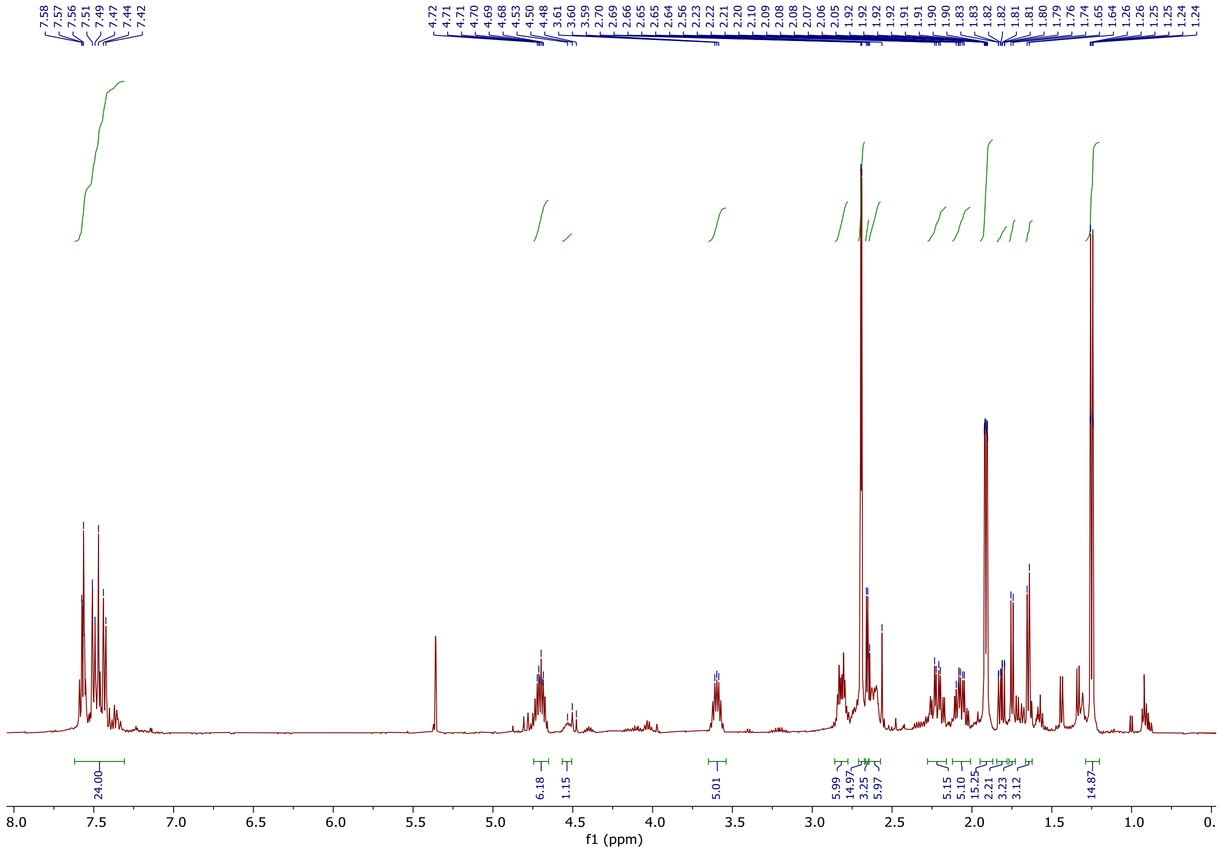

Figure S63. ^{1}H NMR spectrum of isolated **2h-[A]** in DCM-d_2.

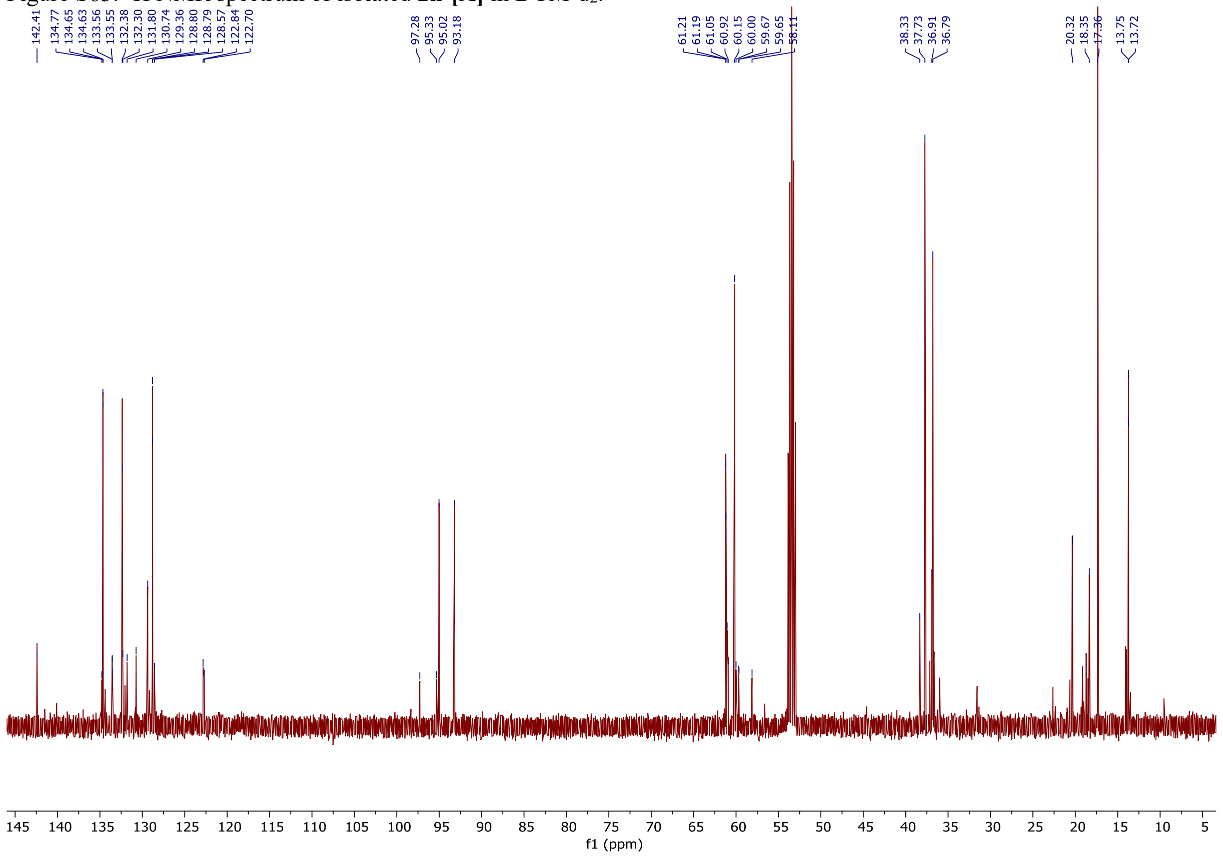

Figure S64. $^{13}C\{^{1}H\}$ NMR spectrum of isolated **2h-[A]** in DCM-d_2.

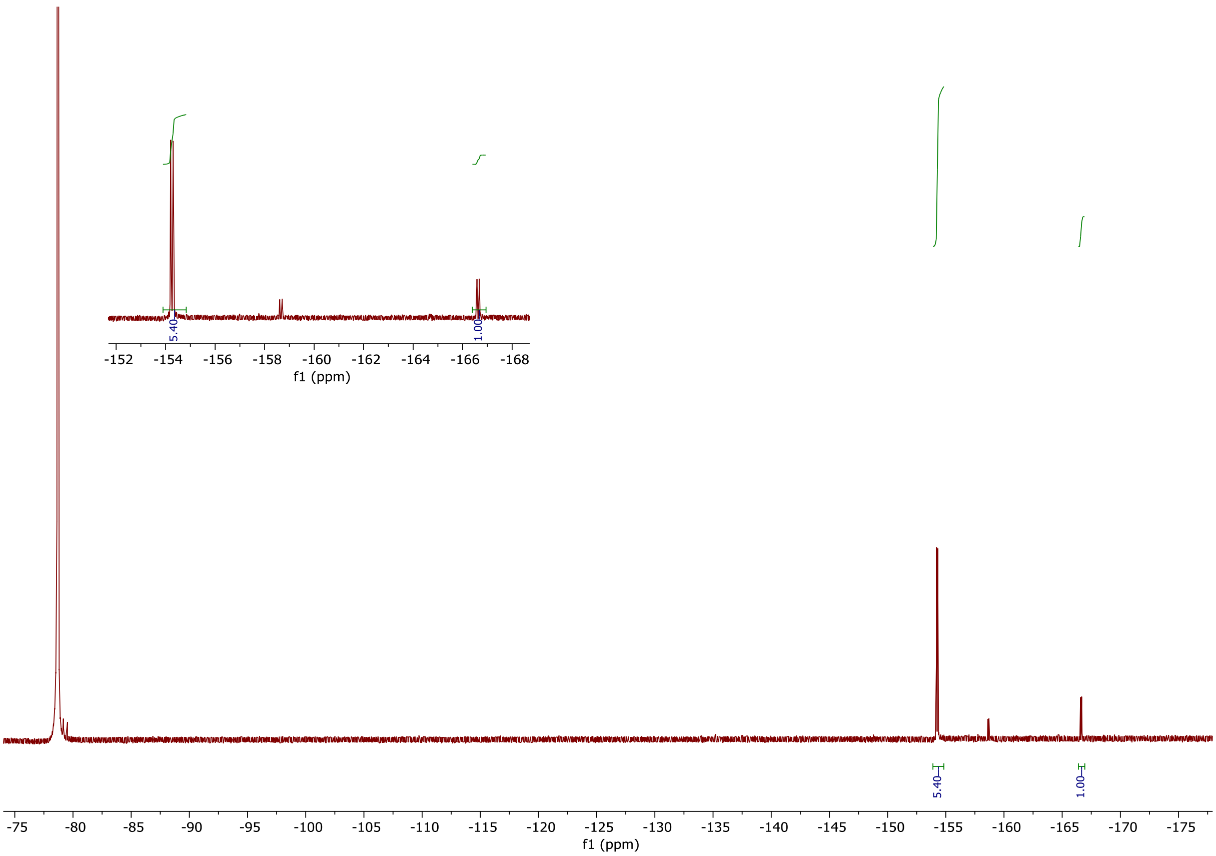

Figure S65. ^{19}F NMR spectrum of isolated **2h-[A]** in DCM-d_2.

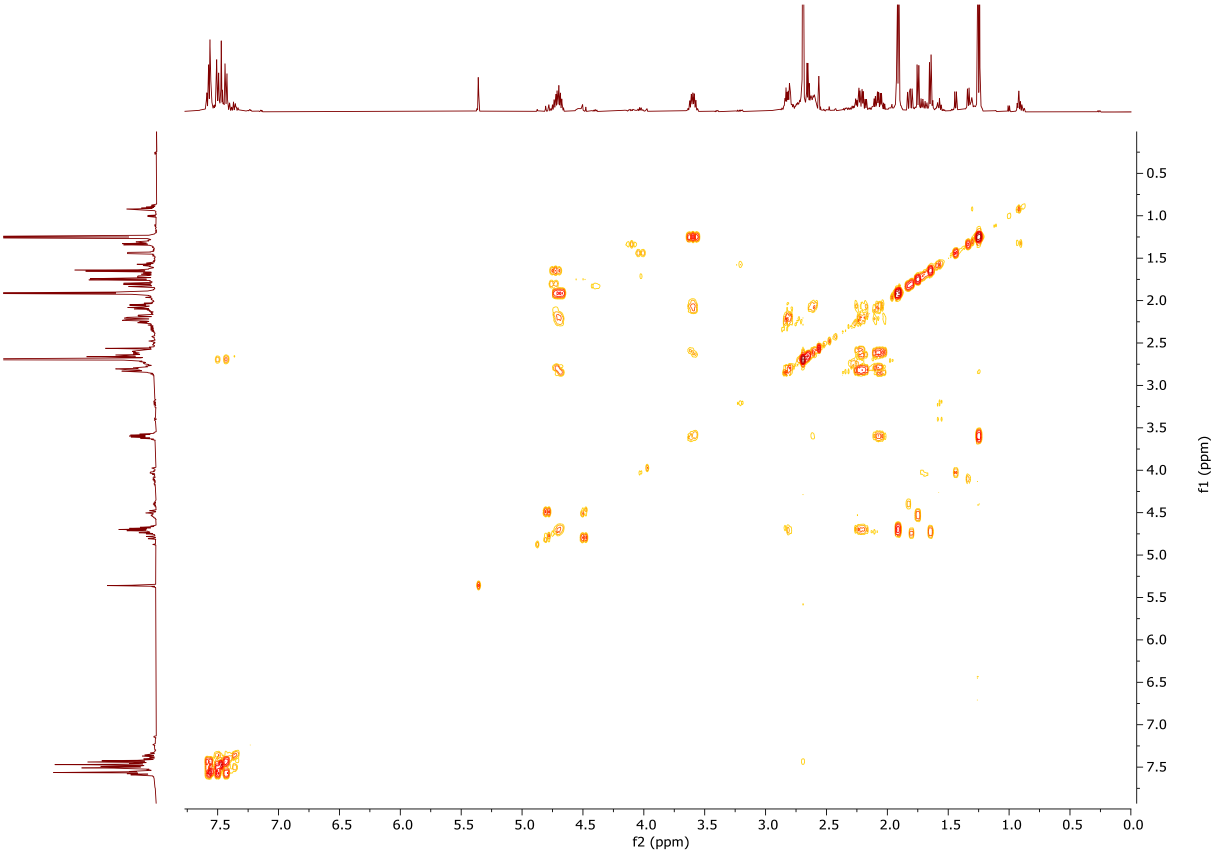

Figure S66. ^{1}H-^{1}H COSY NMR spectrum of isolated **2h-[A]** in DCM-d_2.

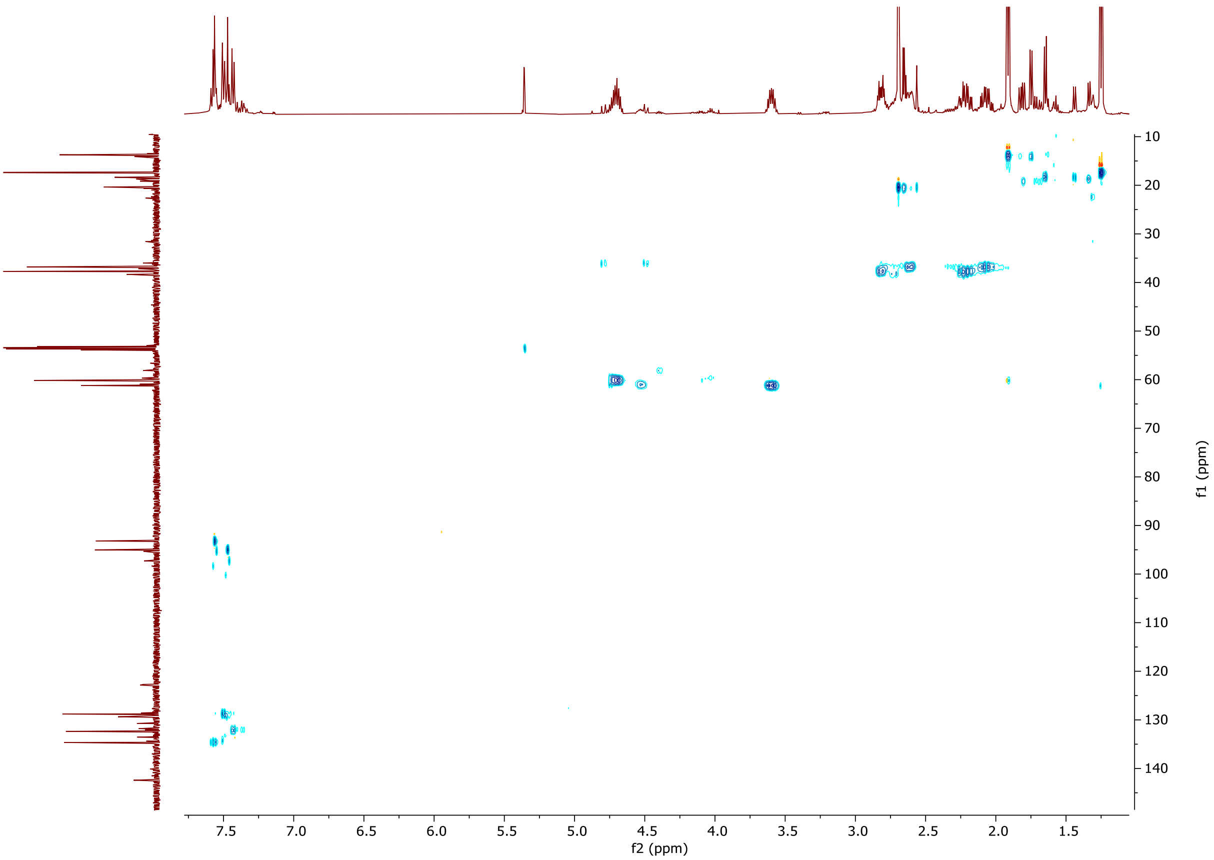

Figure S67. ^{1}H-^{13}C HSQC NMR spectrum of isolated **2h-[A]** in DCM-d_2.

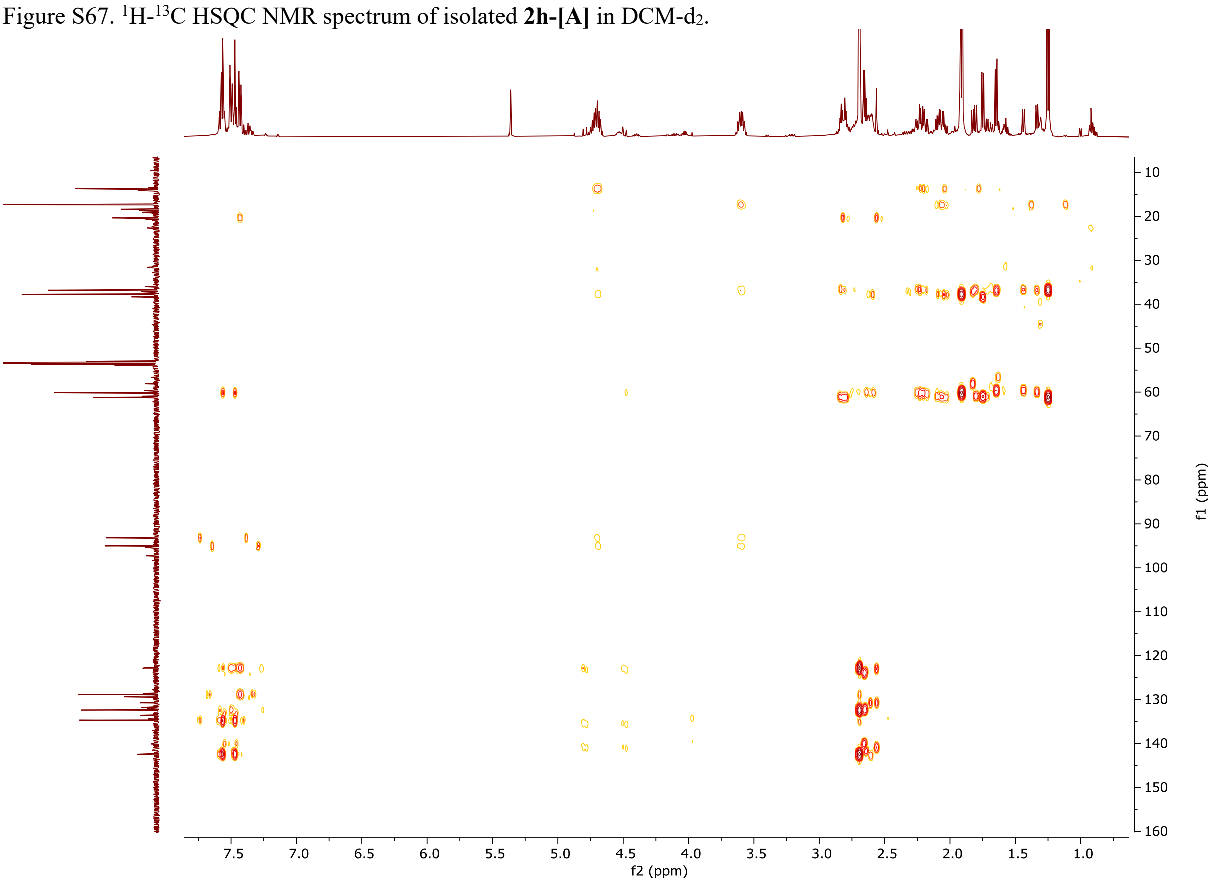

Figure S68. ^{1}H-^{13}C HMBC NMR spectrum of isolated **2h-[A]** in DCM-d_2.

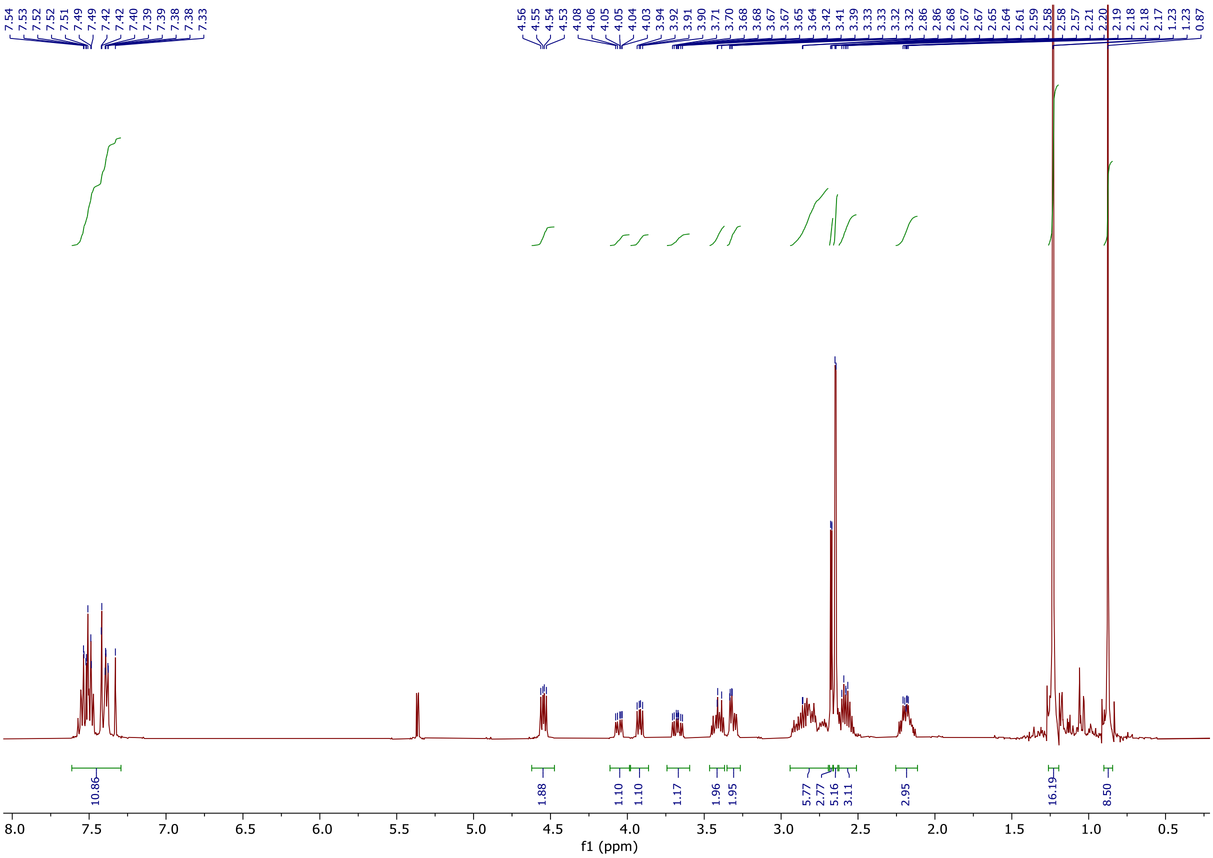

Figure S69. ^{1}H NMR spectrum of isolated **2h-[B]** in DCM-d2.

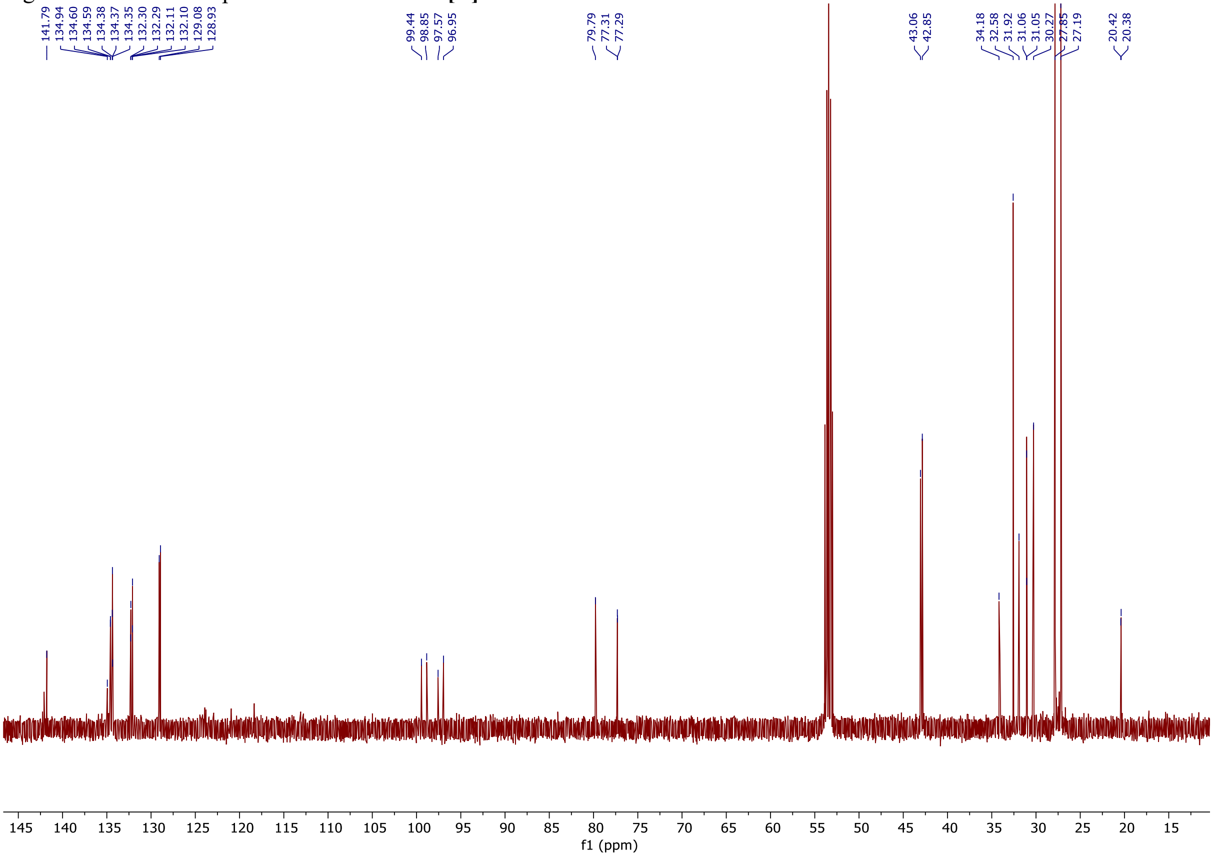

Figure S70. ^{13}C{^{1}H} NMR spectrum of isolated **2h-[B]** in DCM-d2.

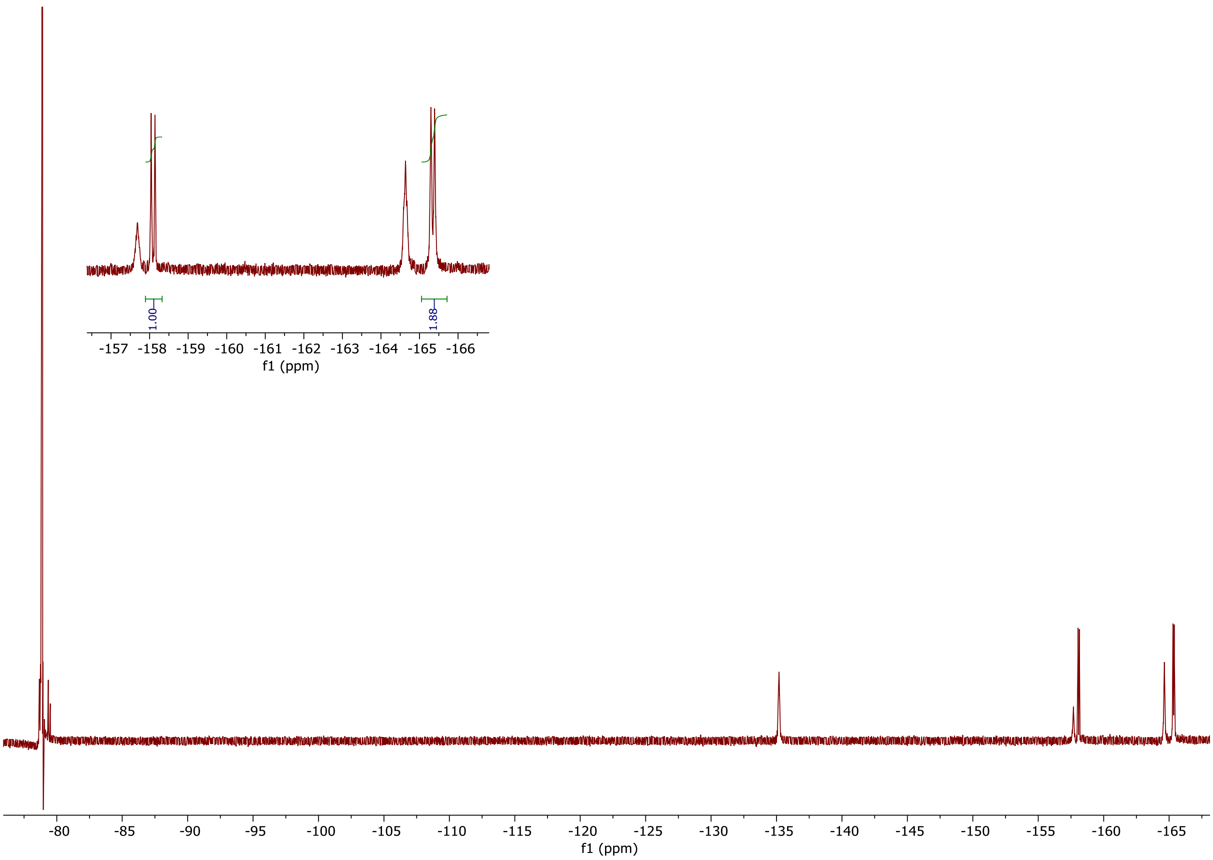

Figure S71. ^{19}F NMR spectrum of isolated **2h-[B]** in DCM-d2.

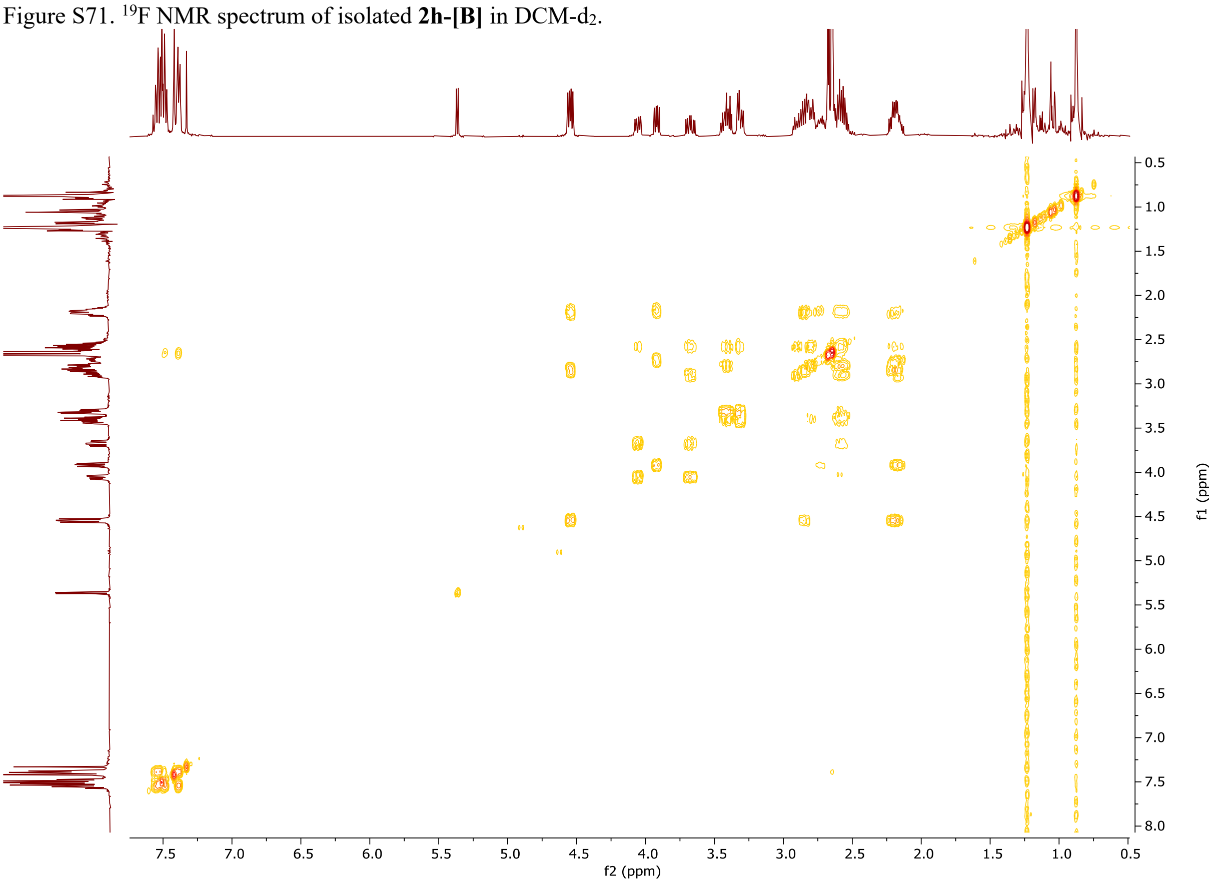

Figure S72. ^{1}H-^{1}H COSY NMR spectrum of isolated **2h-[B]** in DCM-d2.

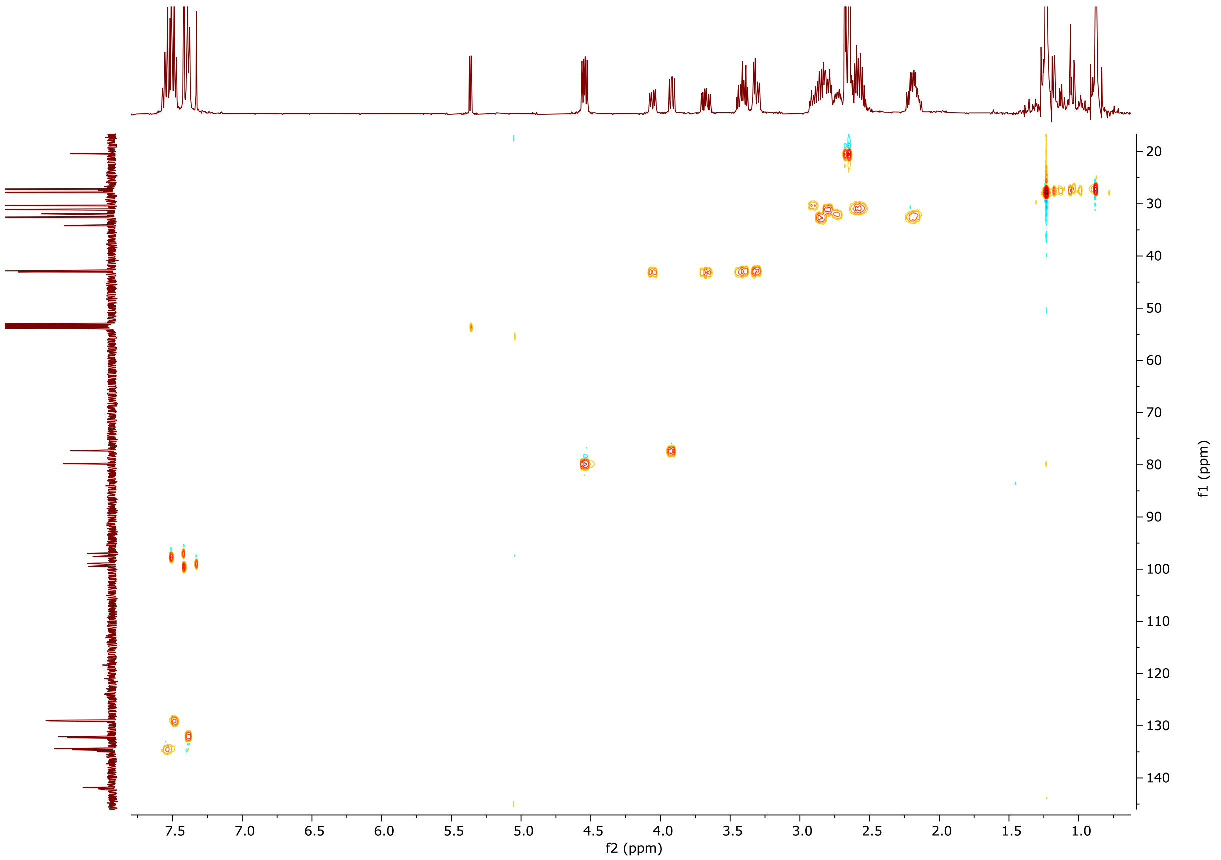

Figure S73. ^{1}H-^{13}C HSQC NMR spectrum of isolated **2h-[B]** in DCM-d2.

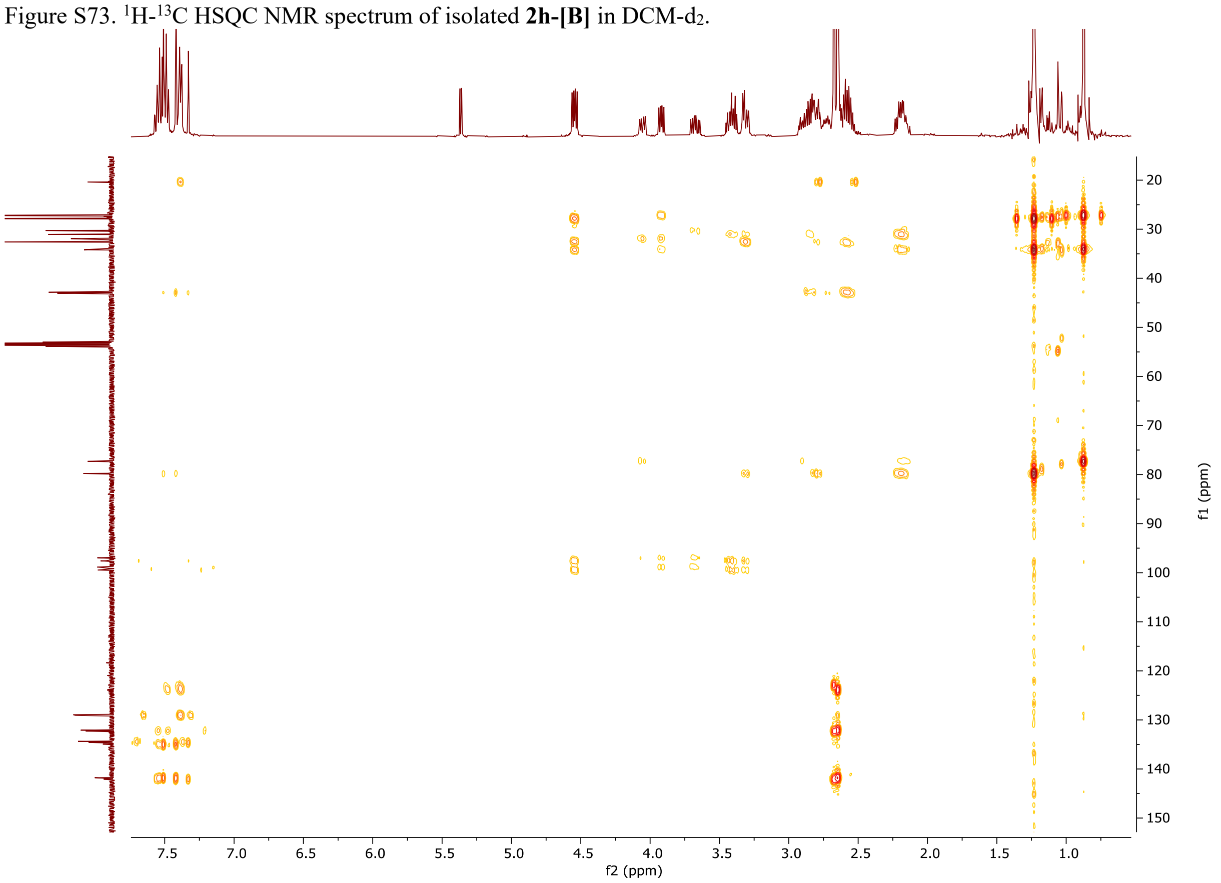

Figure S74. ^{1}H-^{13}C HMBC NMR spectrum of isolated **2h-[B]** in DCM-d2.

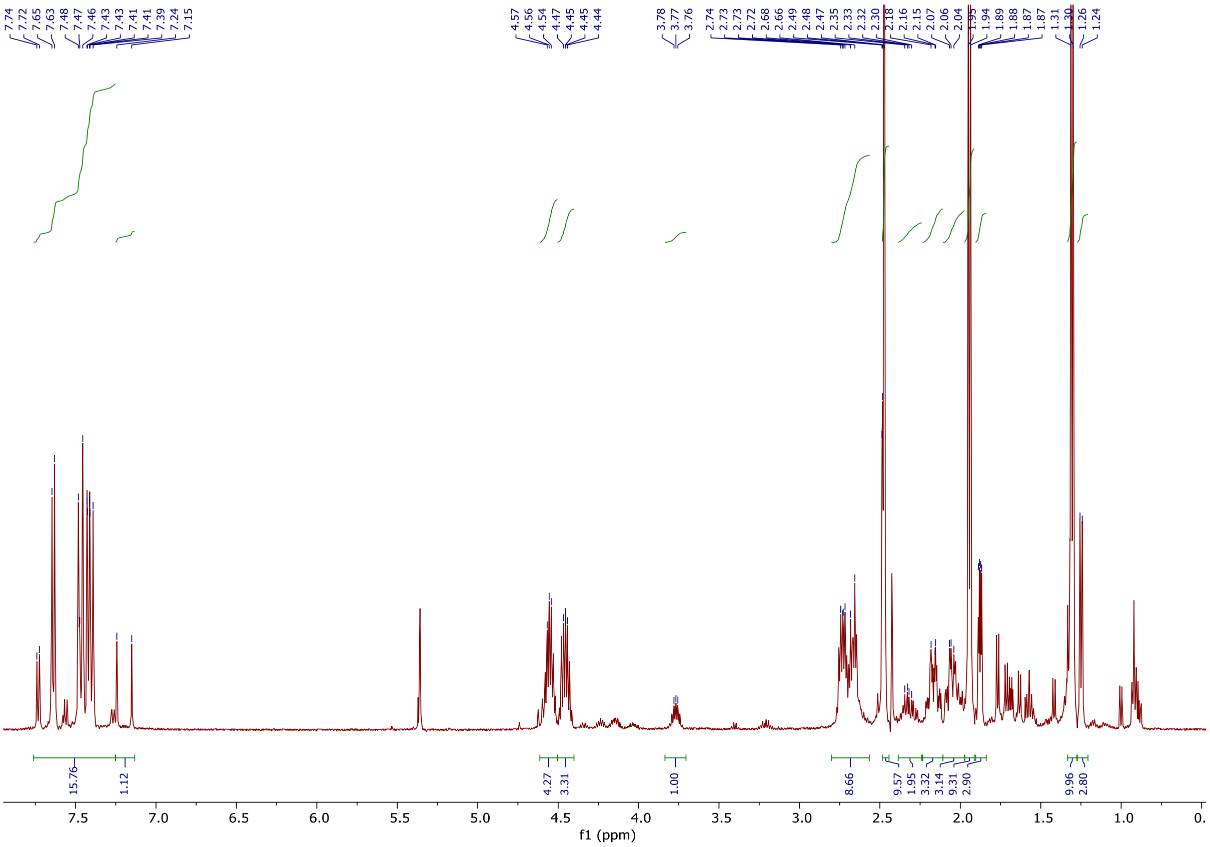

Figure S75. ^{1}H NMR spectrum of isolated **2i-[A]** in DCM-d$_2$.

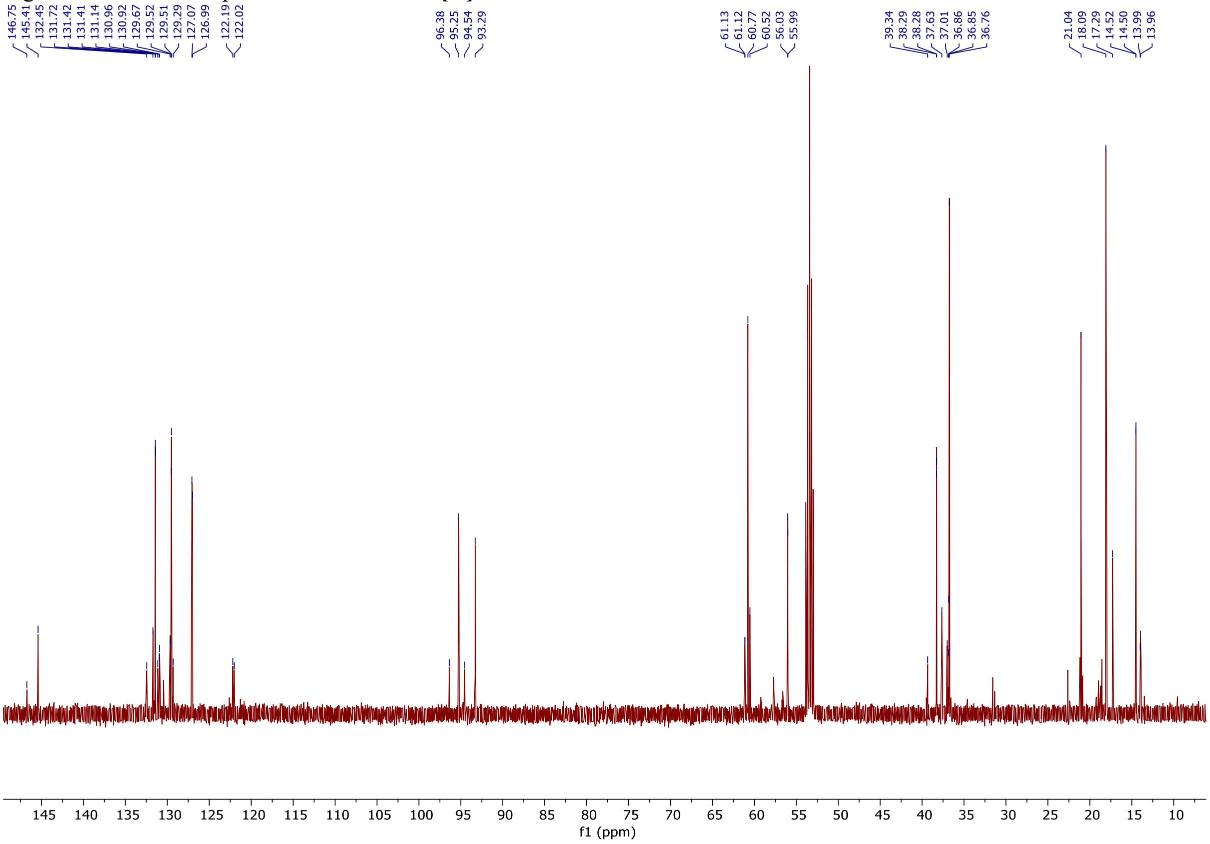

Figure S76. $^{13}C\{^{1}H\}$ NMR spectrum of isolated **2i-[A]** in DCM-d$_2$.

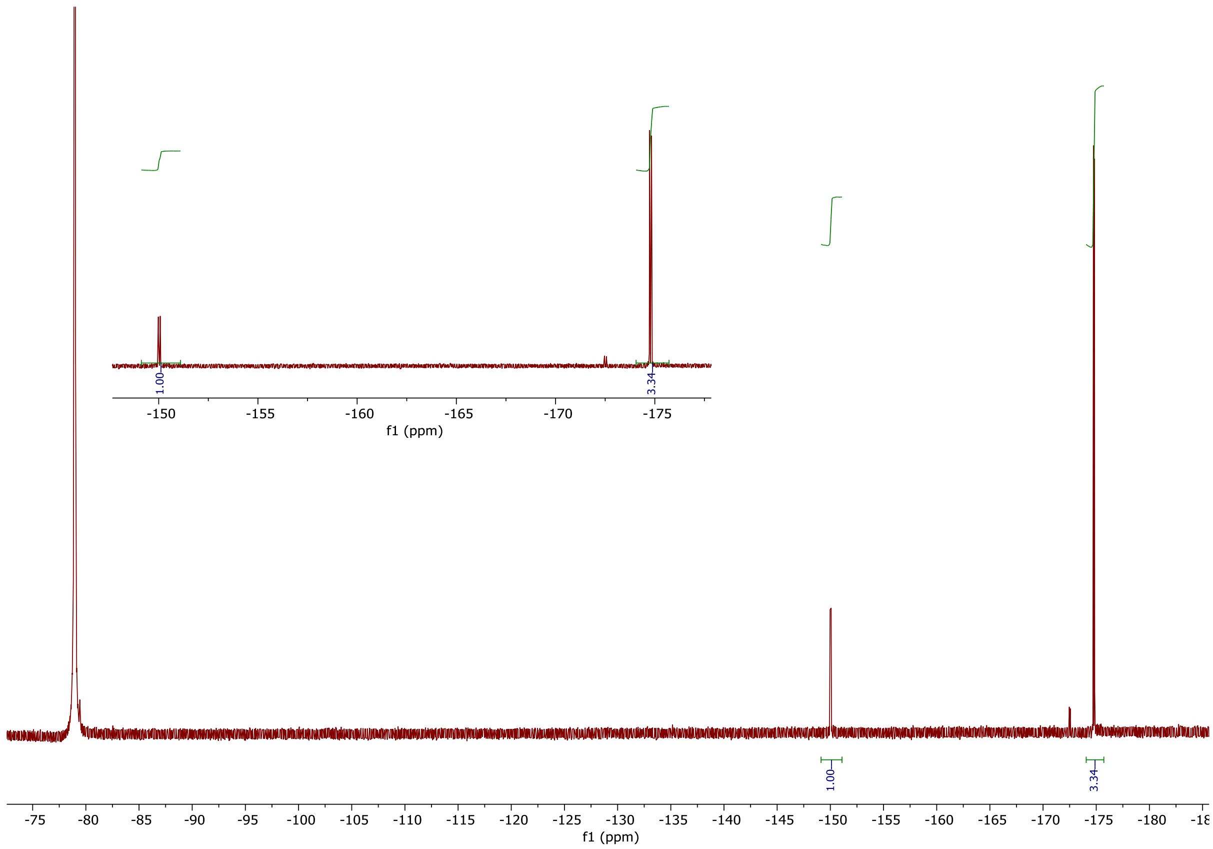

Figure S77. ^{19}F NMR spectrum of isolated **2i-[A]** in DCM-d_2 (immediately after redissolution).

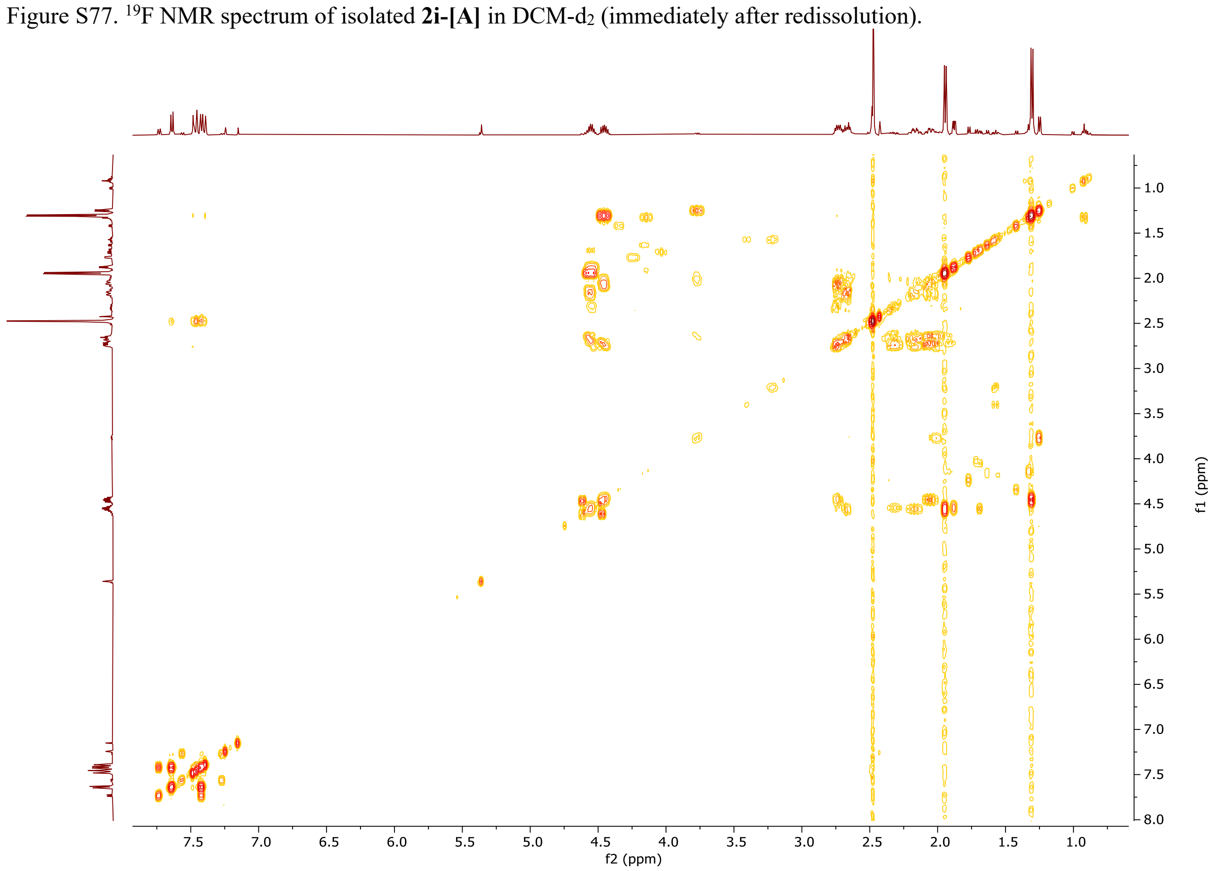

Figure S78. ^{1}H-^{1}H COSY NMR spectrum of isolated **2i-[A]** in DCM-d_2.

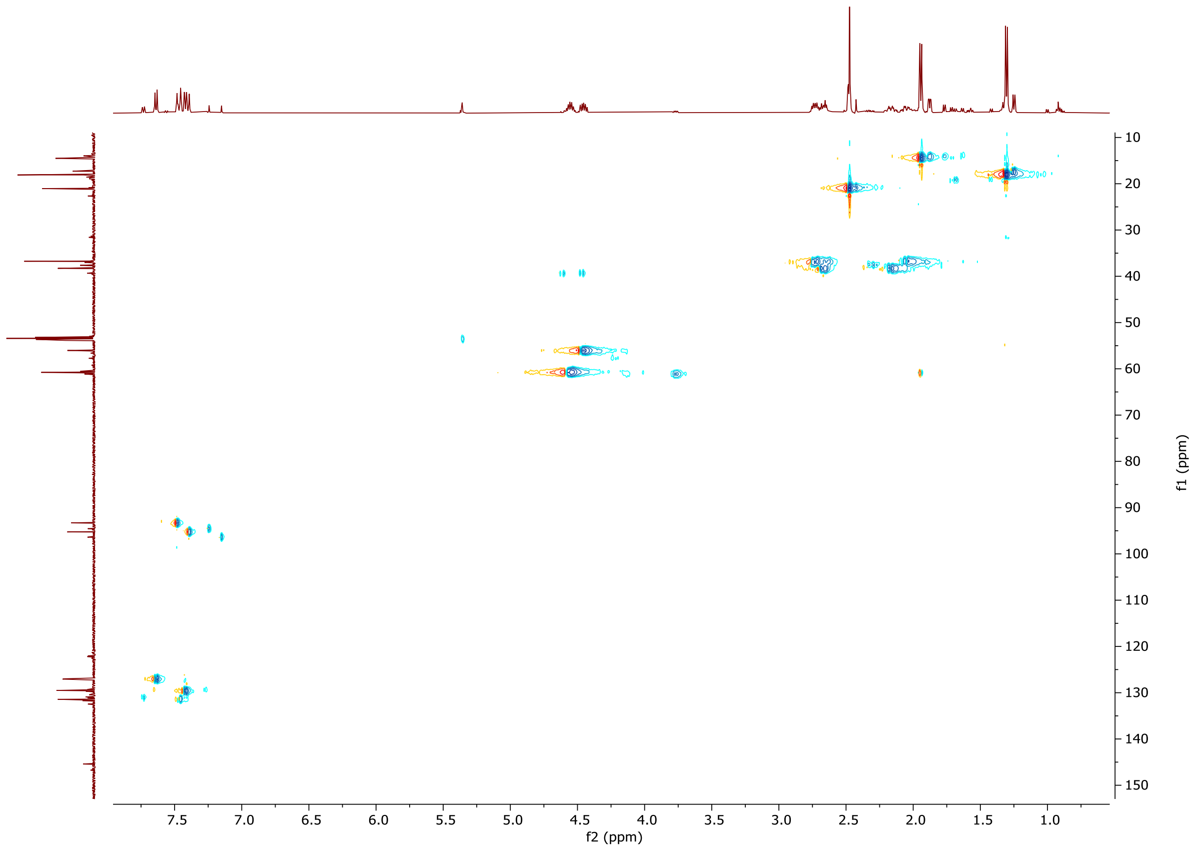

Figure S79. ^{1}H-^{13}C HSQC NMR spectrum of isolated **2i-[A]** in DCM-d_2.

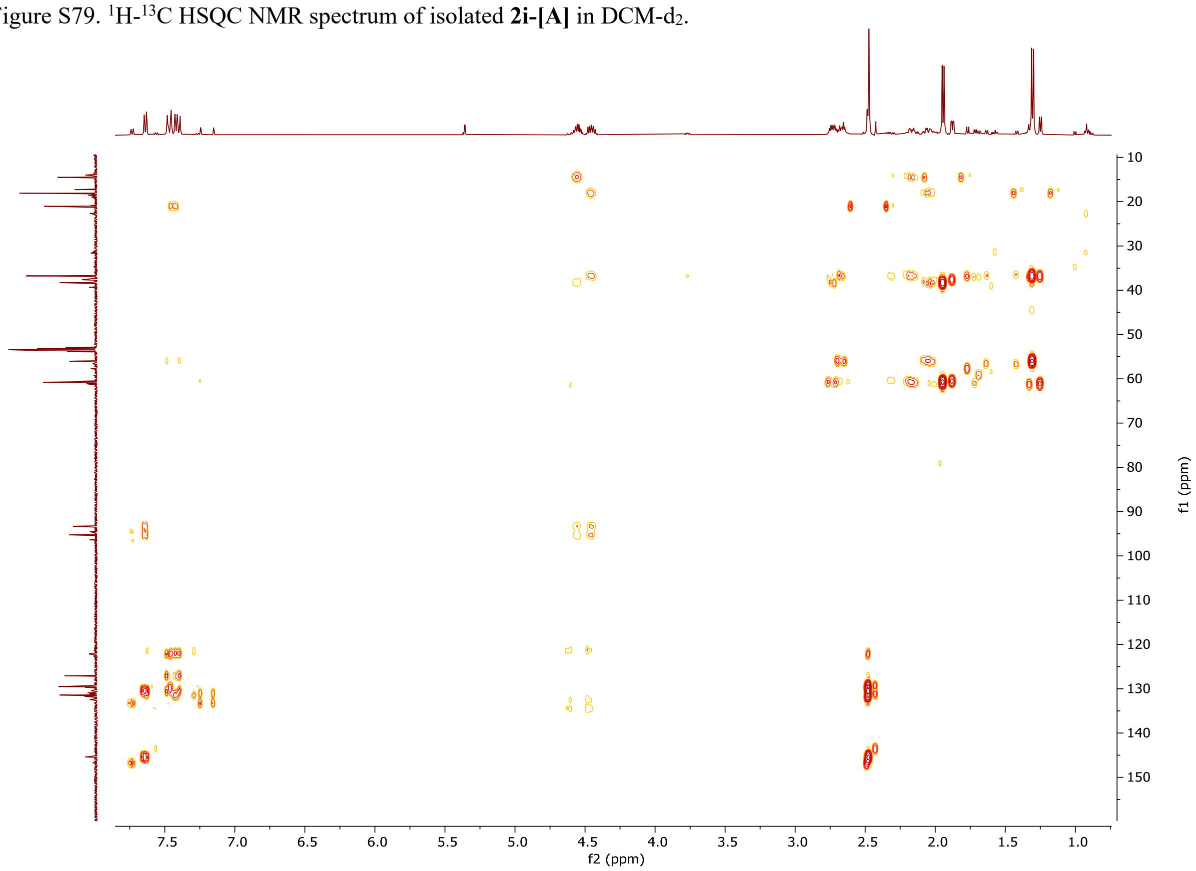

Figure S80. ^{1}H-^{13}C HMBC NMR spectrum of isolated **2i-[A]** in DCM-d_2.

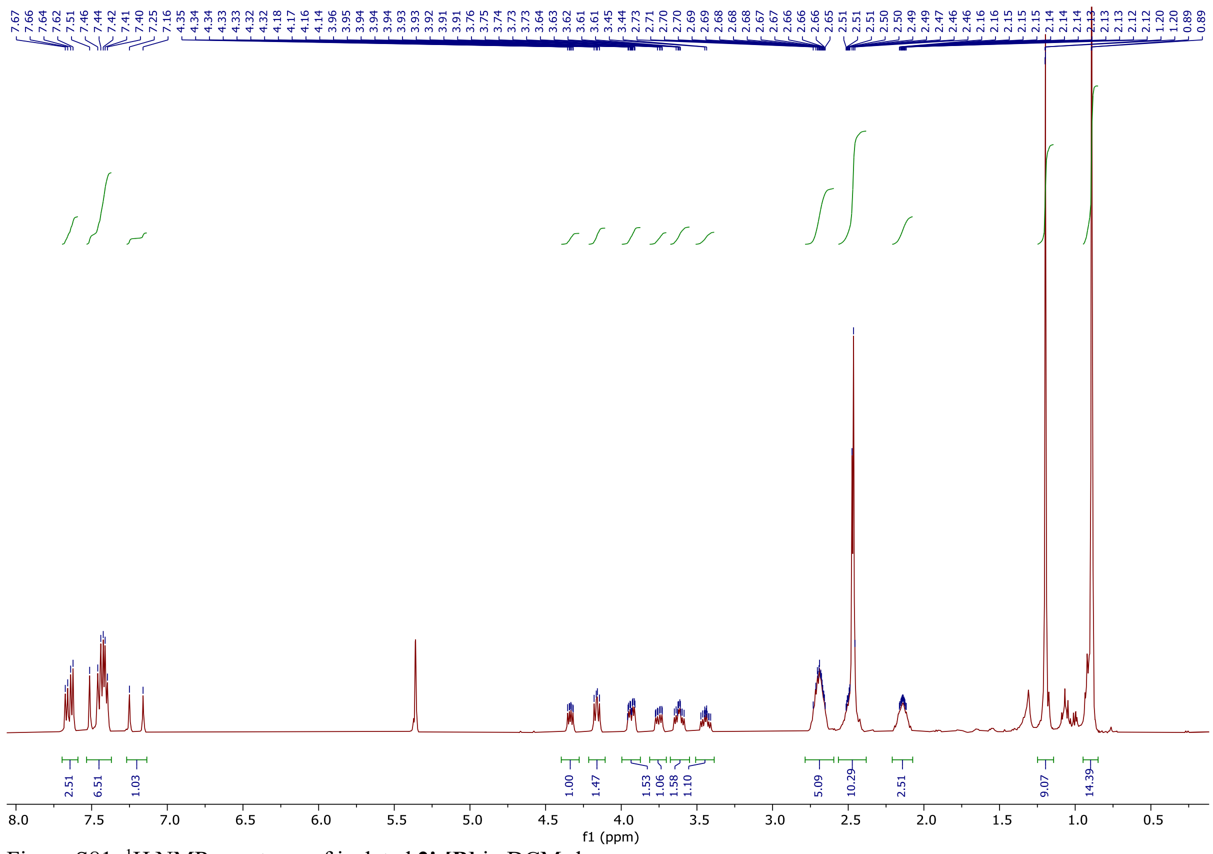

Figure S81. ^{1}H NMR spectrum of isolated **2i-[B]** in DCM-d_2.

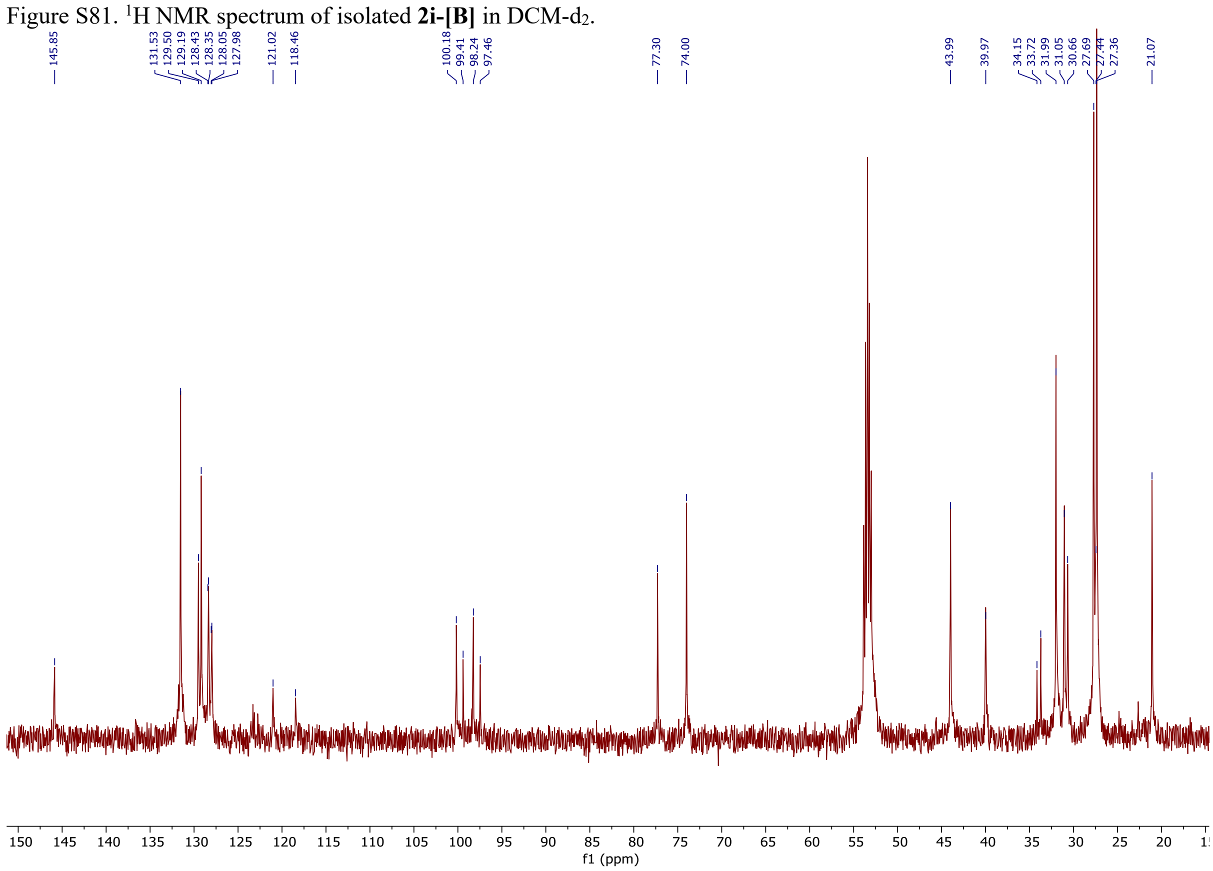

Figure S82. $^{13}C\{^{1}H\}$ NMR spectrum of isolated **2i-[B]** in DCM-d_2.

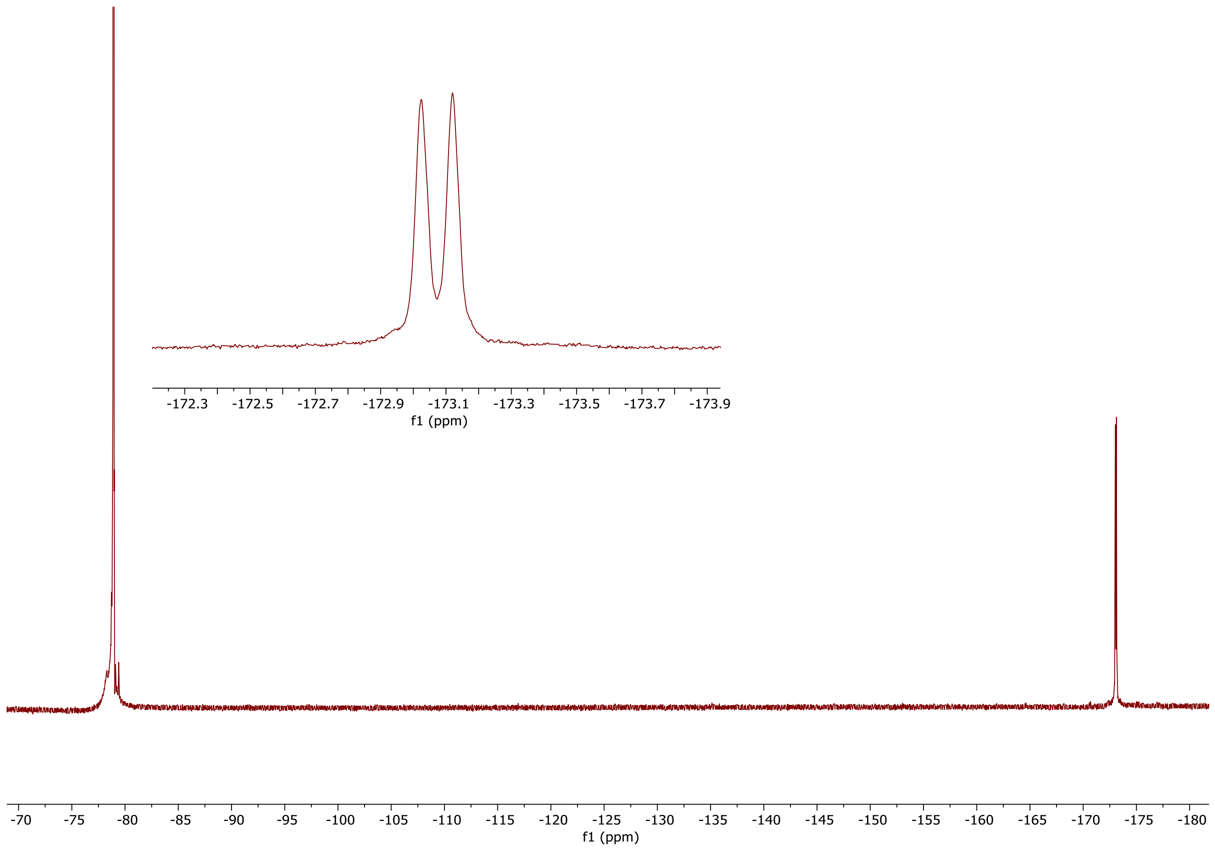

Figure S83. ^{19}F NMR spectrum of isolated **2i-[B]** in DCM-d_2 (immediately after redissolution).

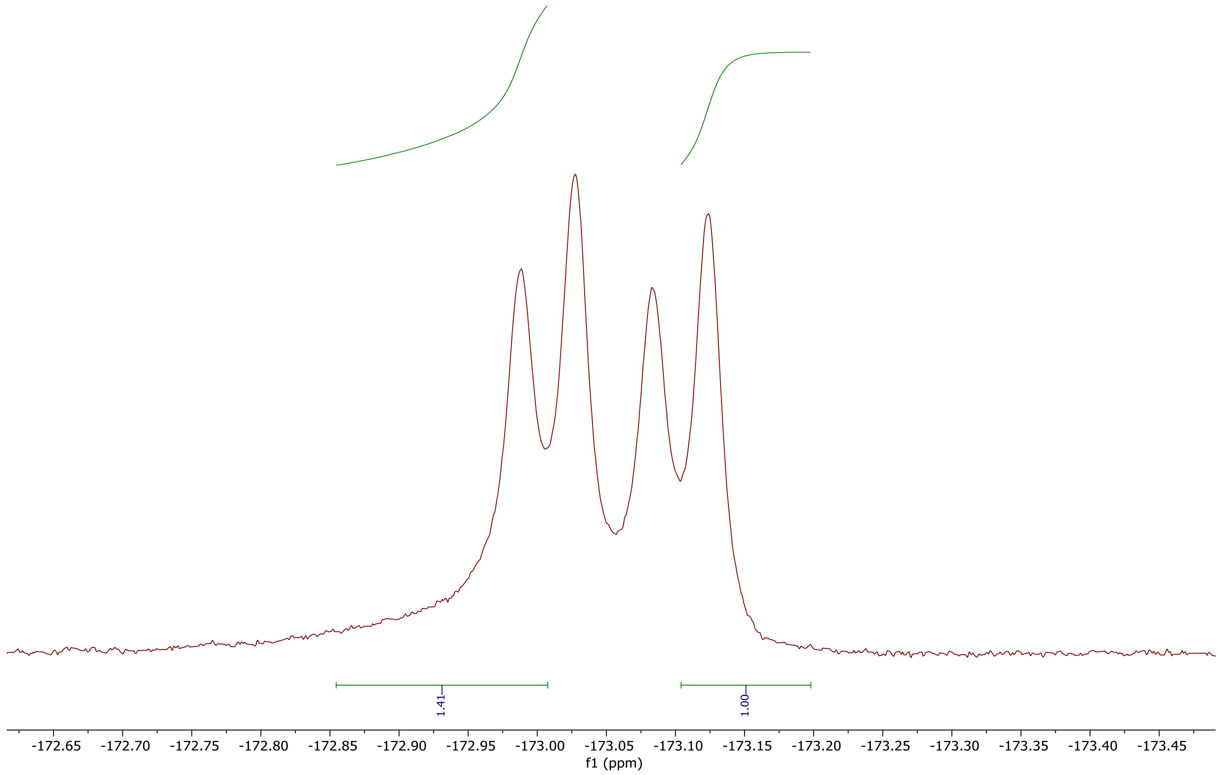

Figure S84. ^{19}F NMR spectrum of isolated **2i-[B]** in DCM-d_2 (12 hours after redissolution).

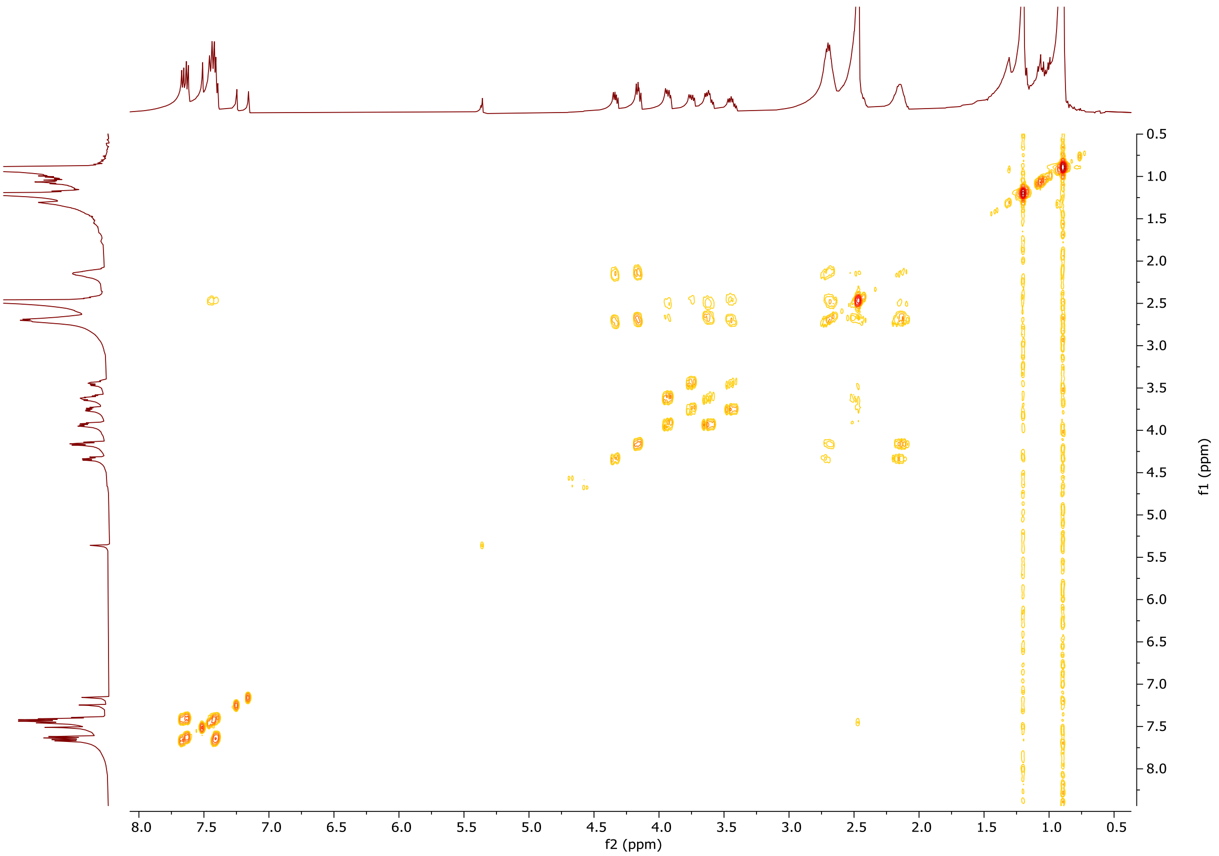

Figure S85. ^{1}H-^{1}H COSY NMR spectrum of isolated **2i-[B]** in DCM-d$_2$.

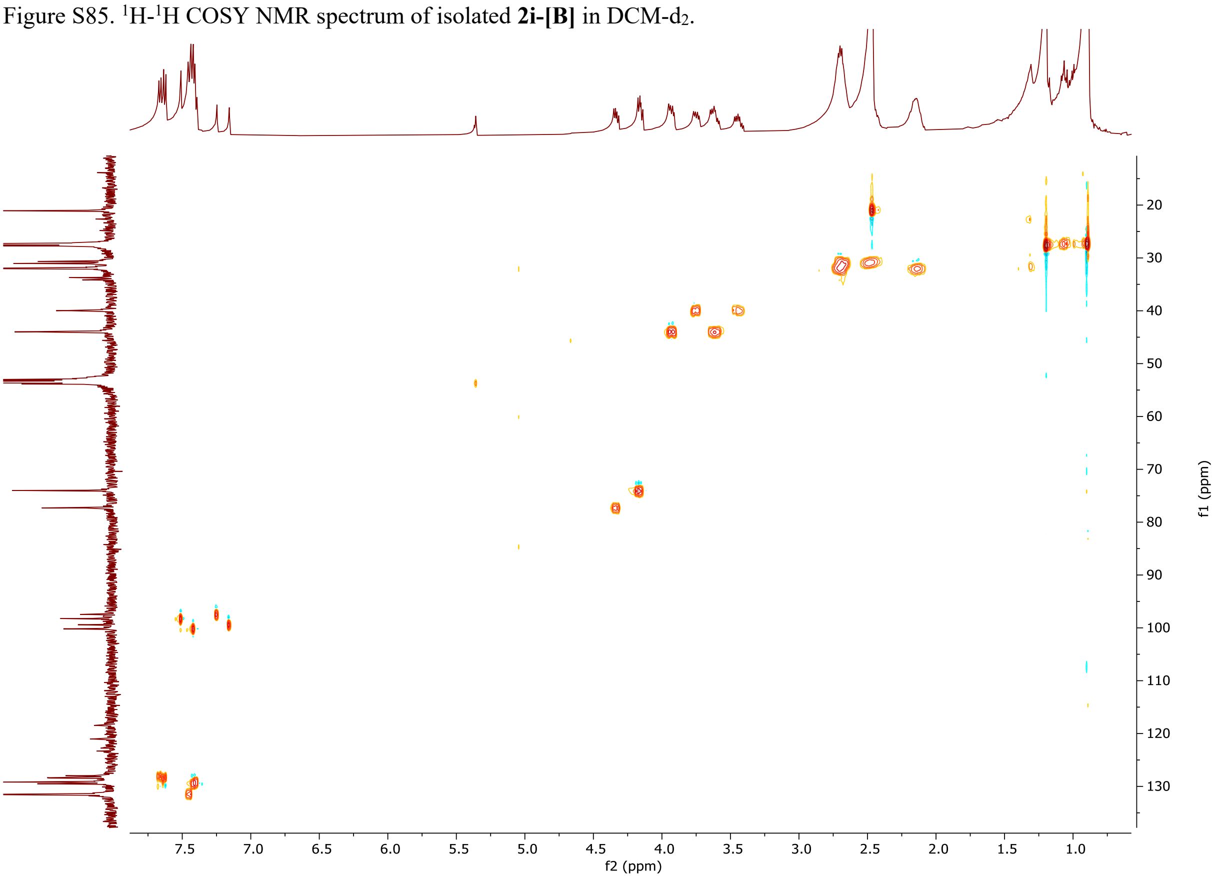

Figure S86. ^{1}H-^{13}C HSQC NMR spectrum of isolated **2i-[B]** in DCM-d$_2$.

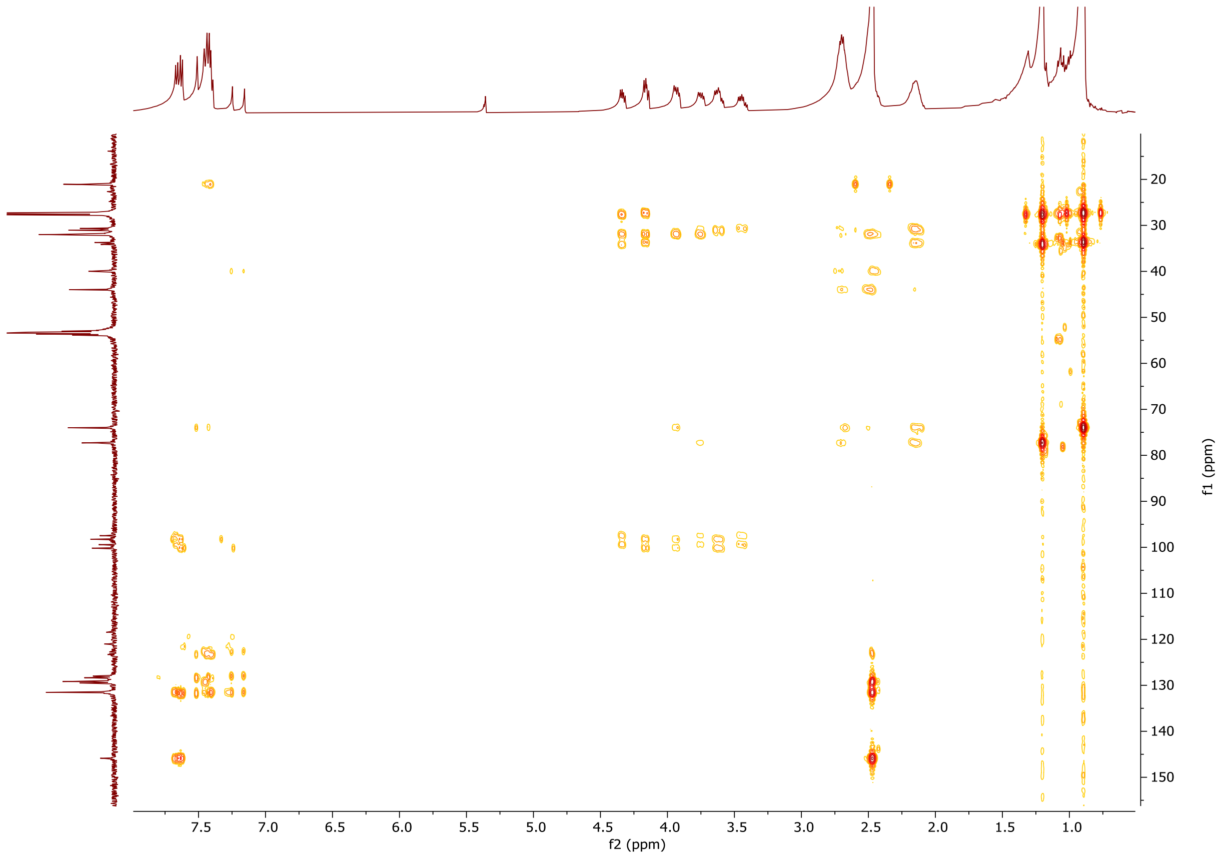

Figure S87. ^{1}H-^{13}C HMBC NMR spectrum of isolated **2i-[B]** in DCM-d_2.

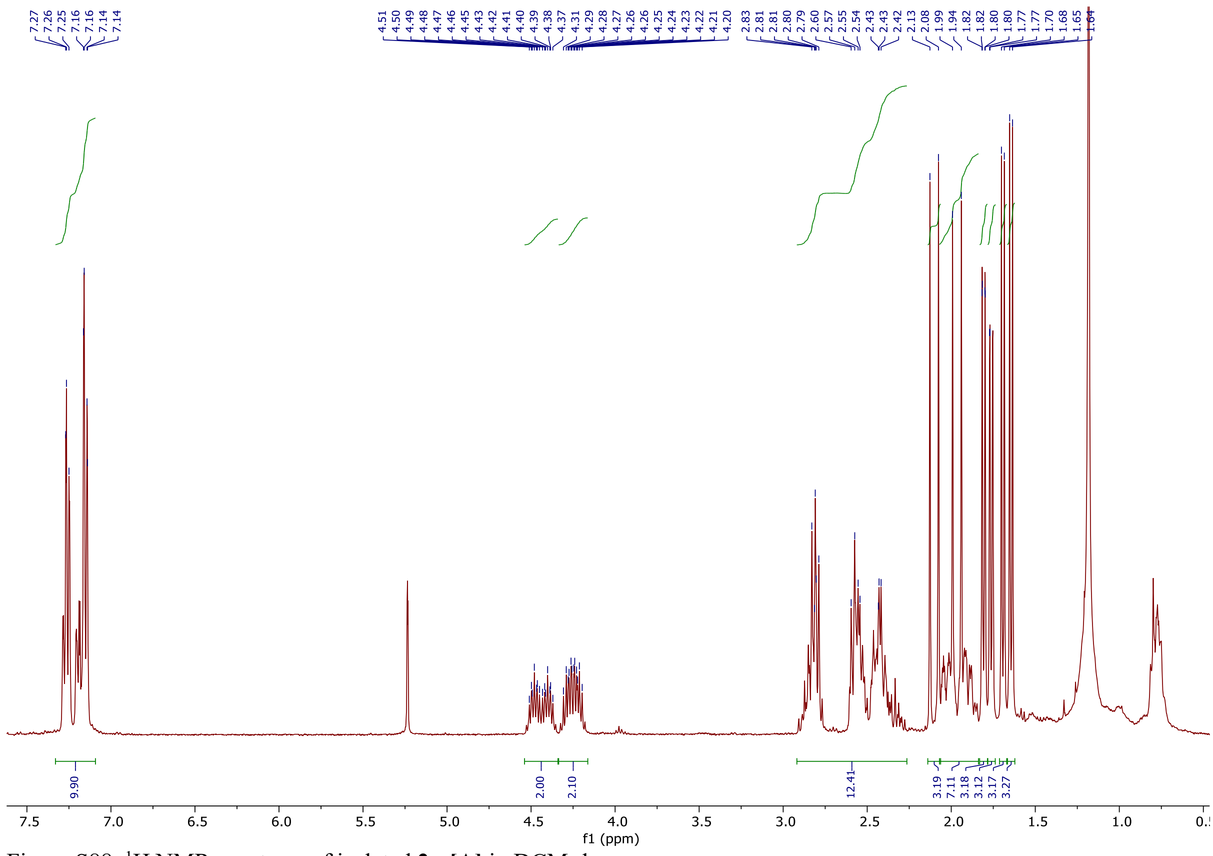

Figure S88. ^{1}H NMR spectrum of isolated **2x-[A]** in DCM-d_2.

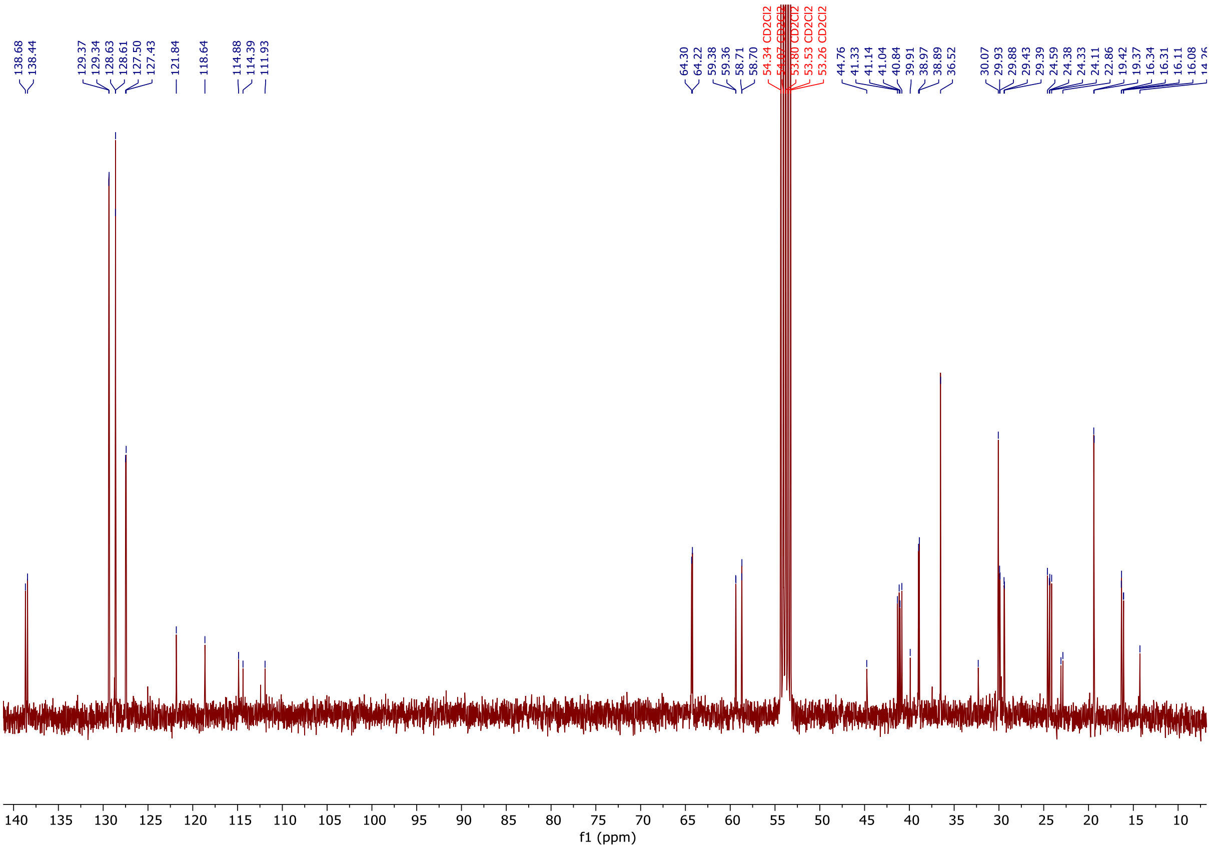

Figure S89. $^{13}C\{^{1}H\}$ NMR spectrum of isolated **2x-[A]** in DCM-d2.

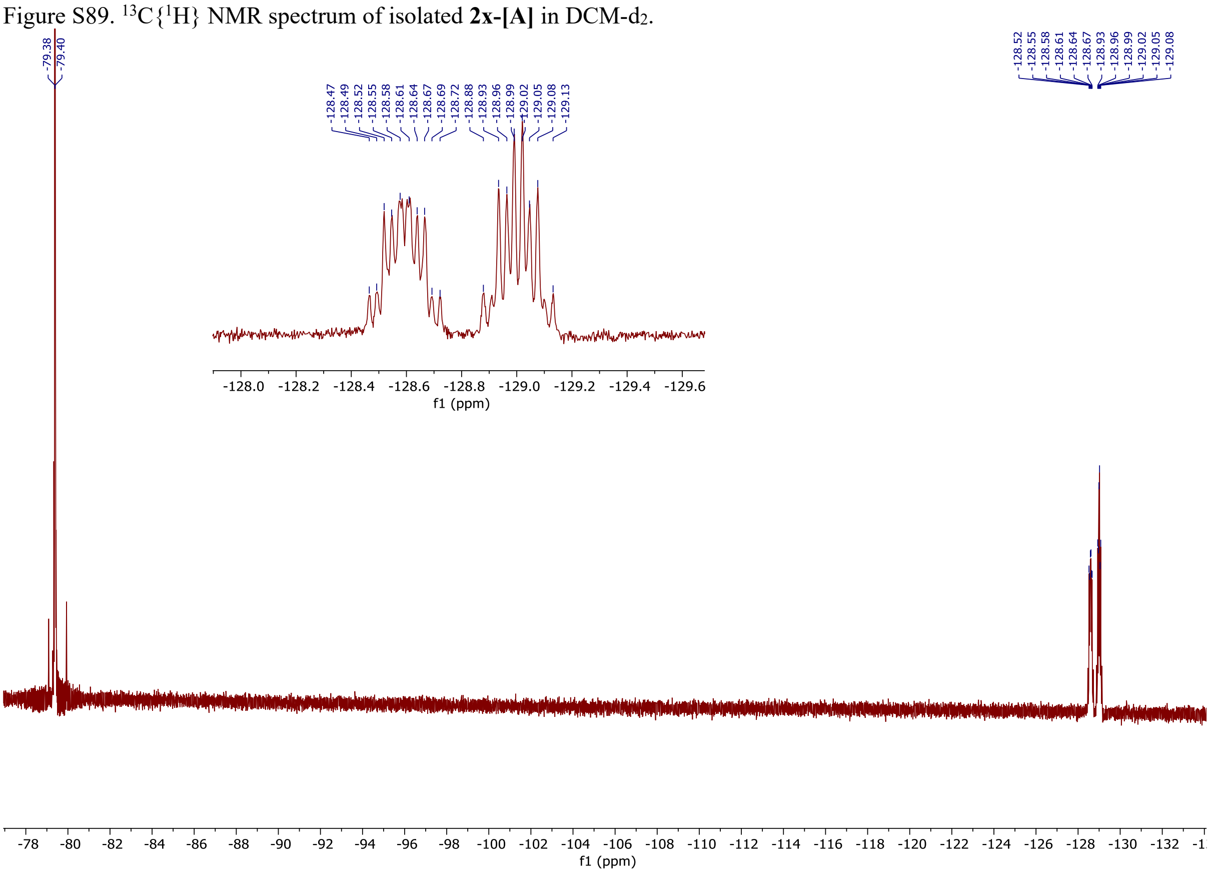

Figure S90. ^{19}F NMR spectrum of isolated **2x-[A]** in DCM-d2.

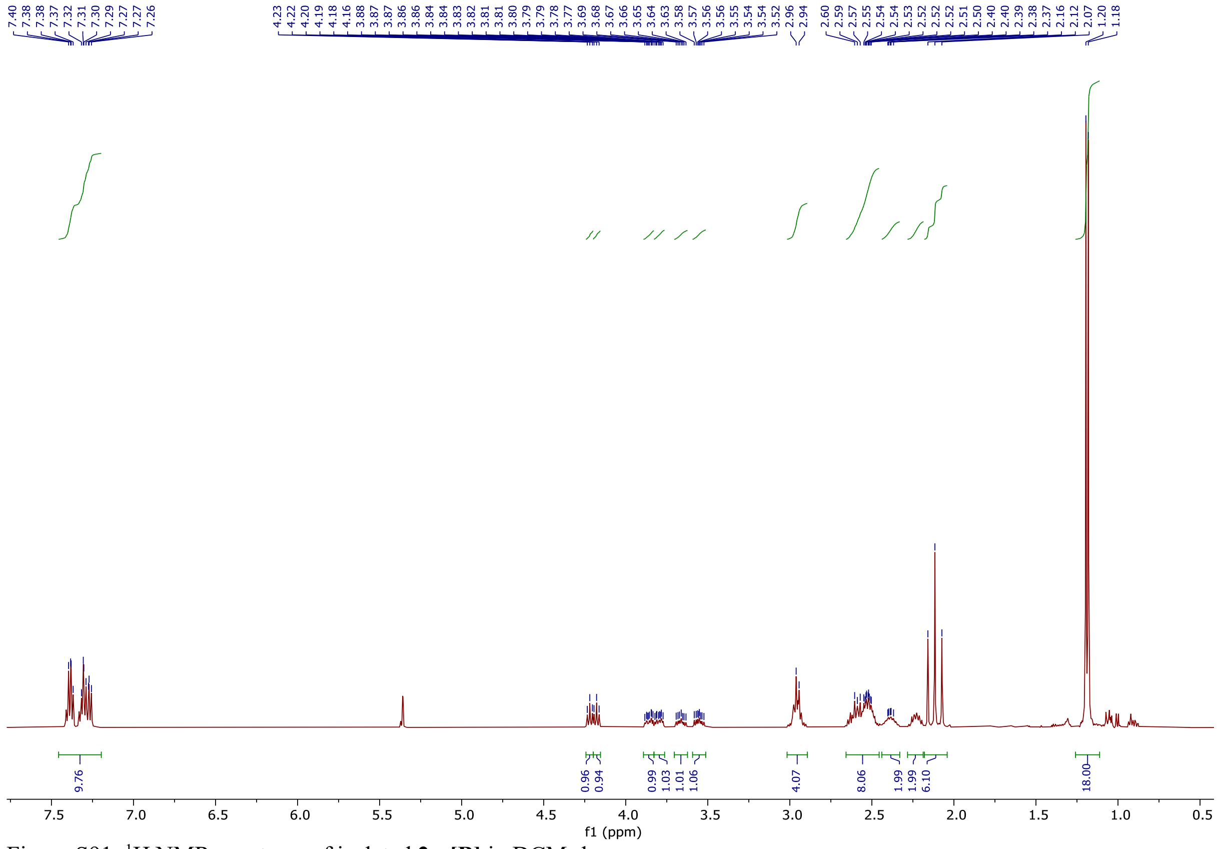

Figure S91. ^{1}H NMR spectrum of isolated **2x-[B]** in DCM-d2.

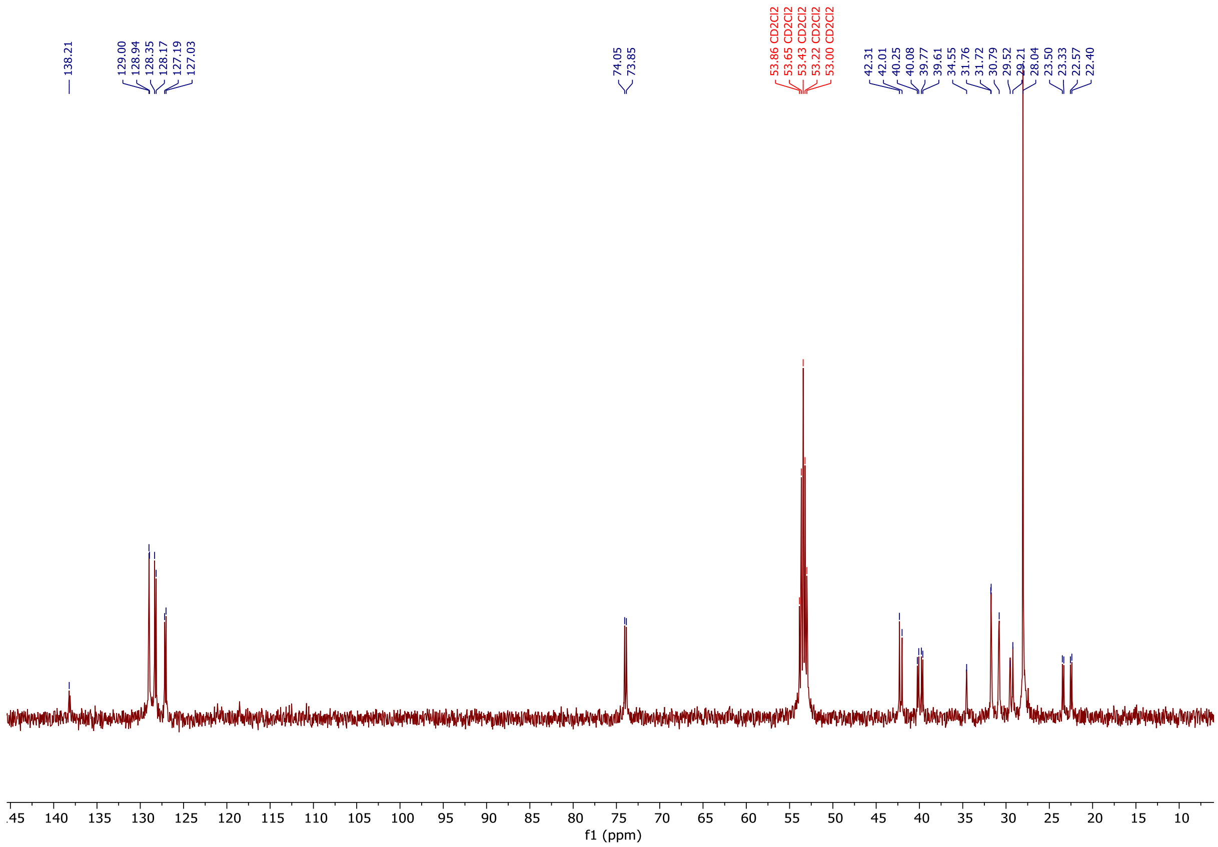

Figure S92. ^{13}C{^{1}H} NMR spectrum of isolated **2x-[B]** in DCM-d2.

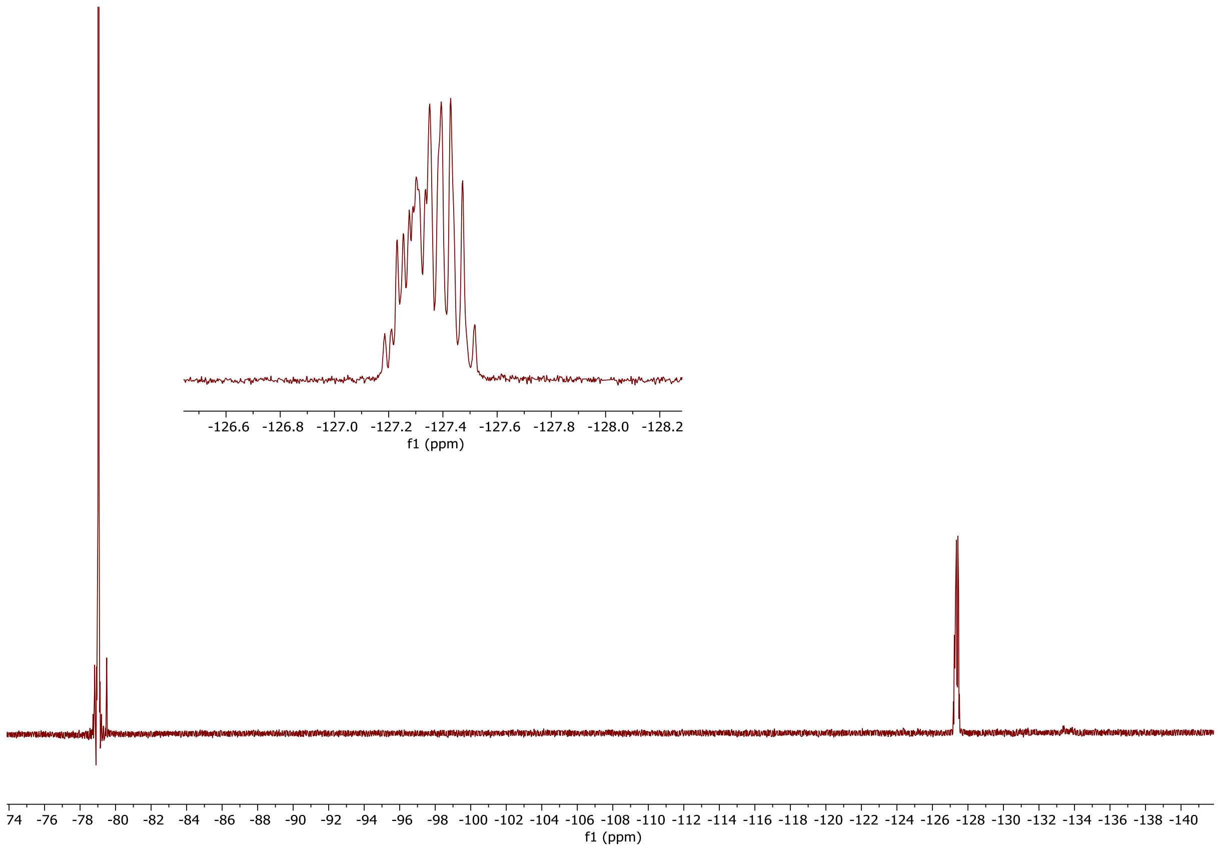

Figure S93. ^{19}F NMR spectrum of isolated **2x-[B]** in DCM-d2.

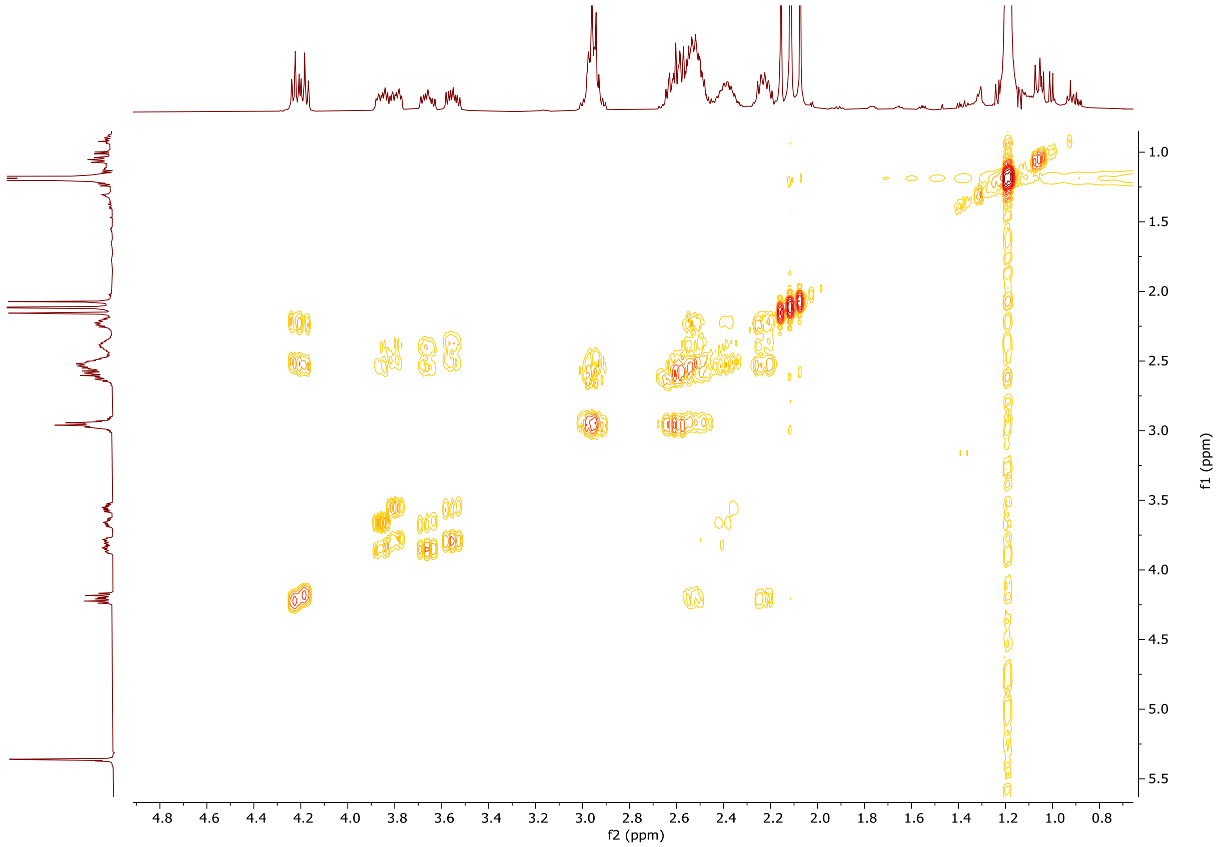

Figure S94. ^{1}H-^{1}H COSY NMR spectrum of isolated **2x-[B]** in DCM-d2.

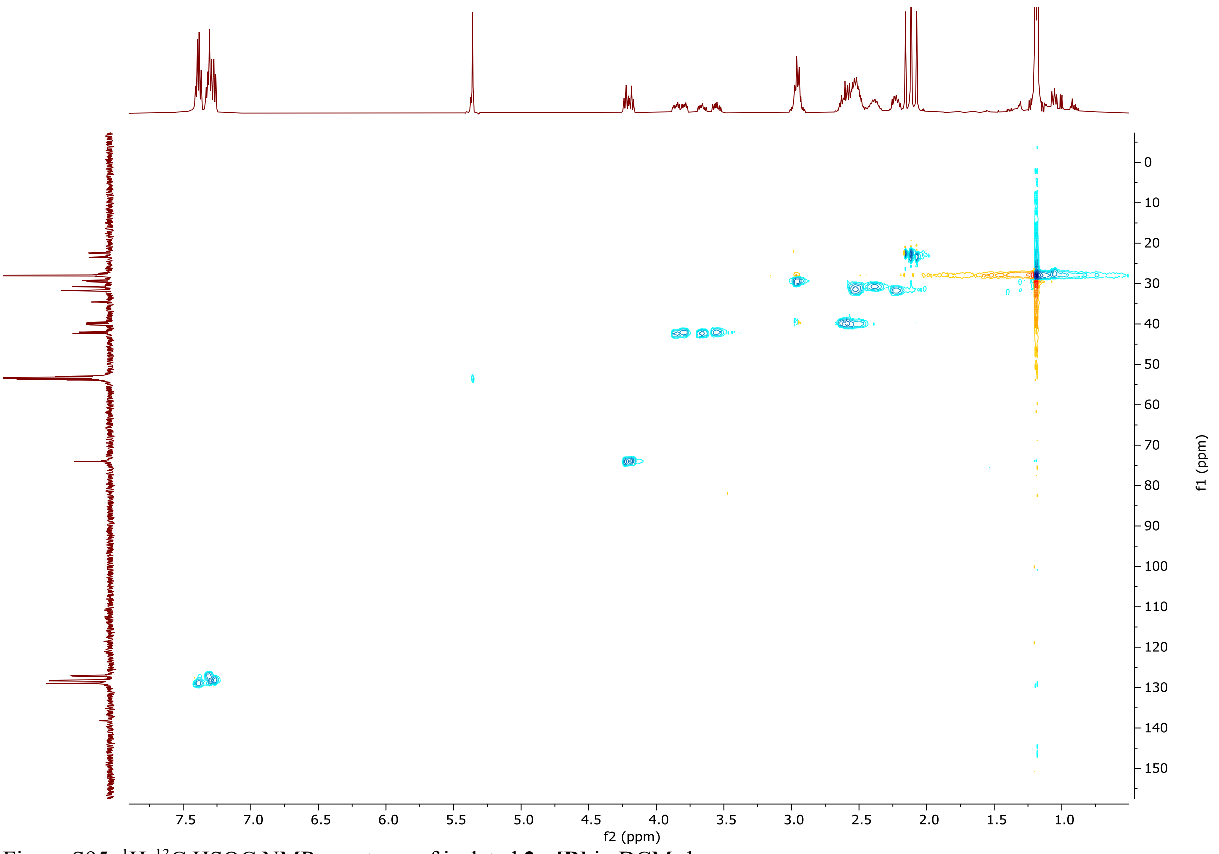

Figure S95. ^{1}H-^{13}C HSQC NMR spectrum of isolated **2x-[B]** in DCM-d2.

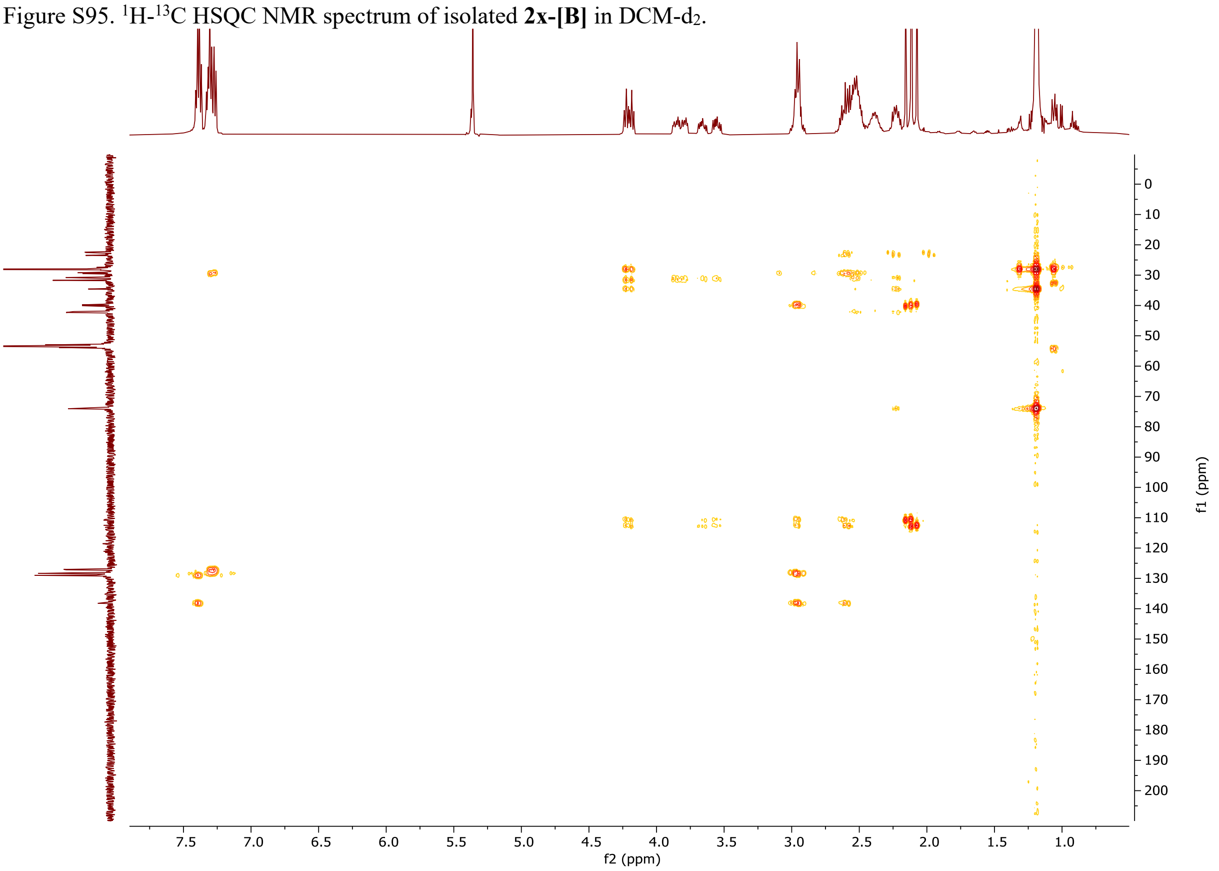

Figure S96. ^{1}H-^{13}C HMBC NMR spectrum of isolated **2x-[B]** in DCM-d2.

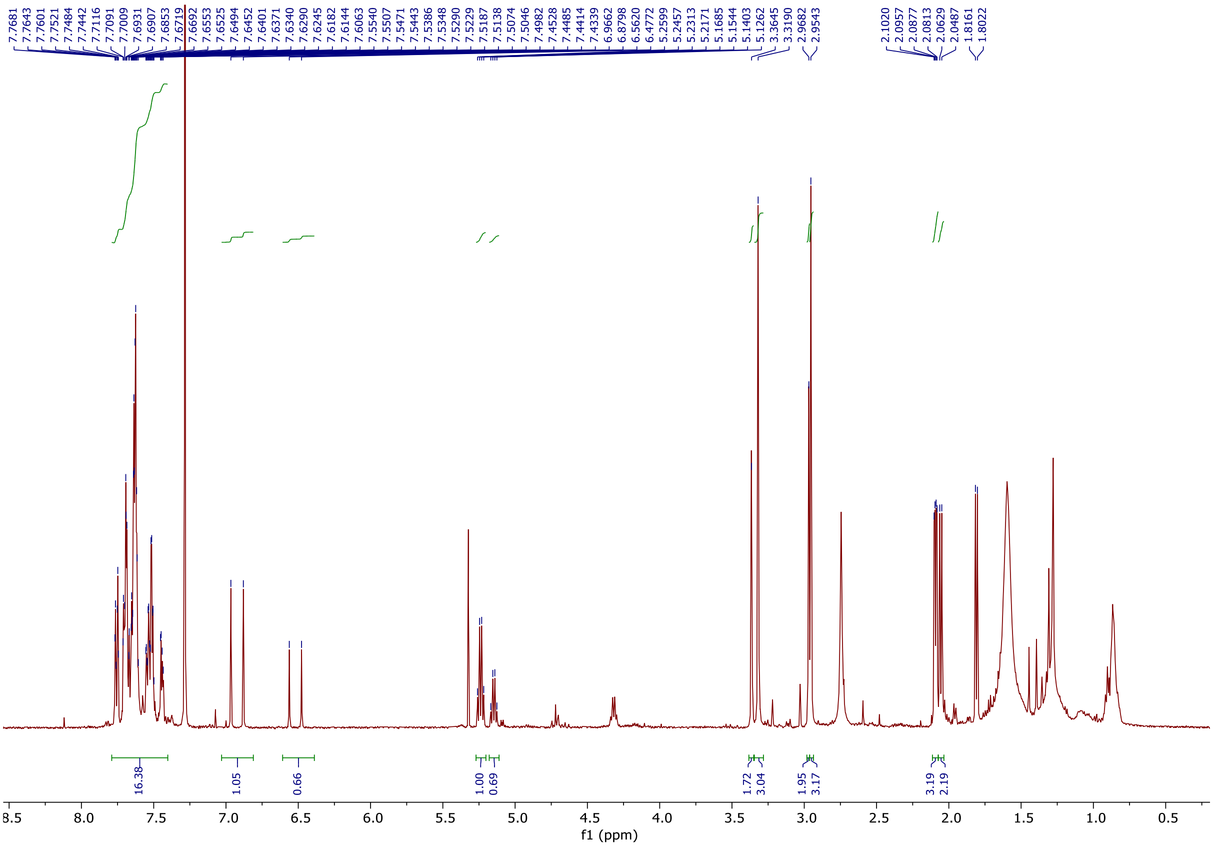

Figure S97. ^{1}H NMR of isolated **2a-[N*s*]** (mixture of both isomers) in $CDCl_3$. Residual H_2O and hexanes at 1.5, 1.2 and 0.8 ppm.

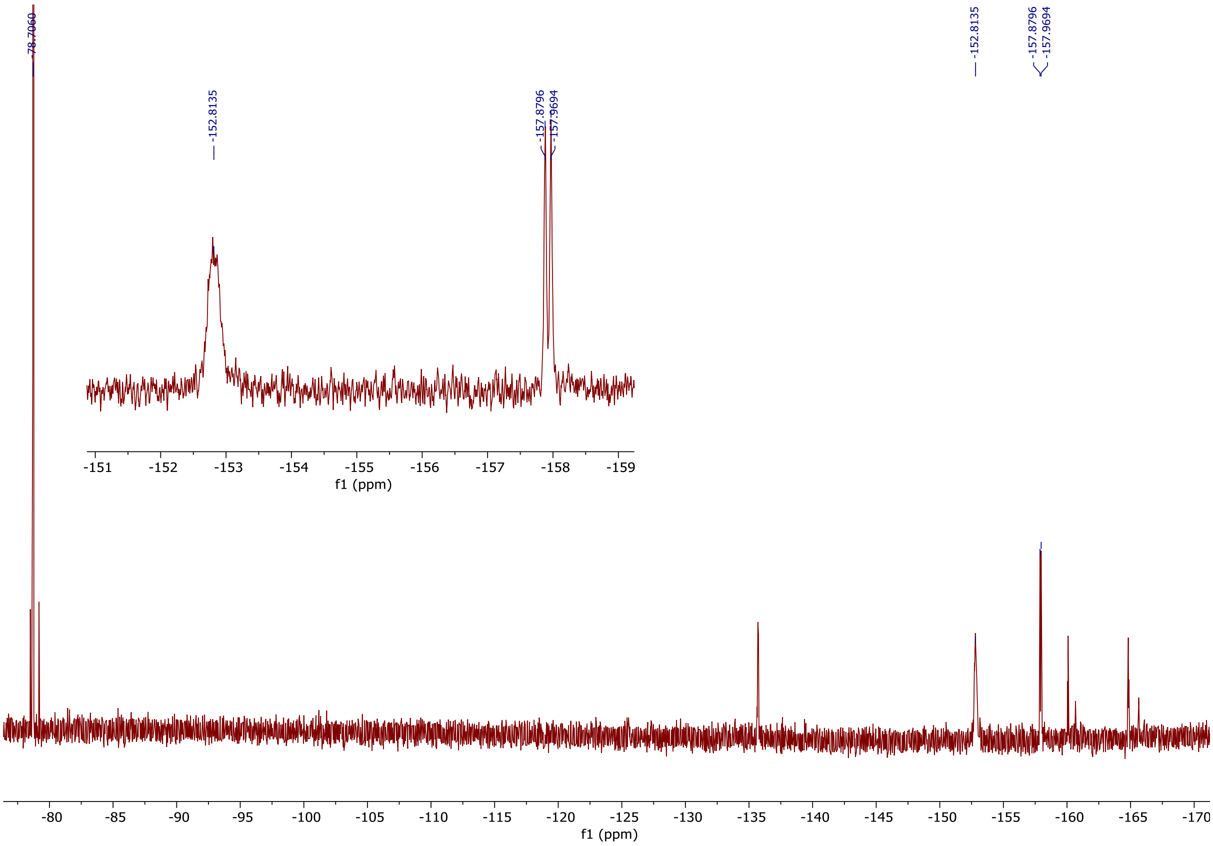

Figure S98. ^{19}F NMR of isolated **2a-[N*s*]** (mixture of both isomer) in $CDCl_3$. Minor signals at 135, 160 and 165 ppm due to $[B(OH)(C_6F_5)_3]^-$ or $[(OH)\{B(C_6F_5)_3\}_2]^-$ anions.

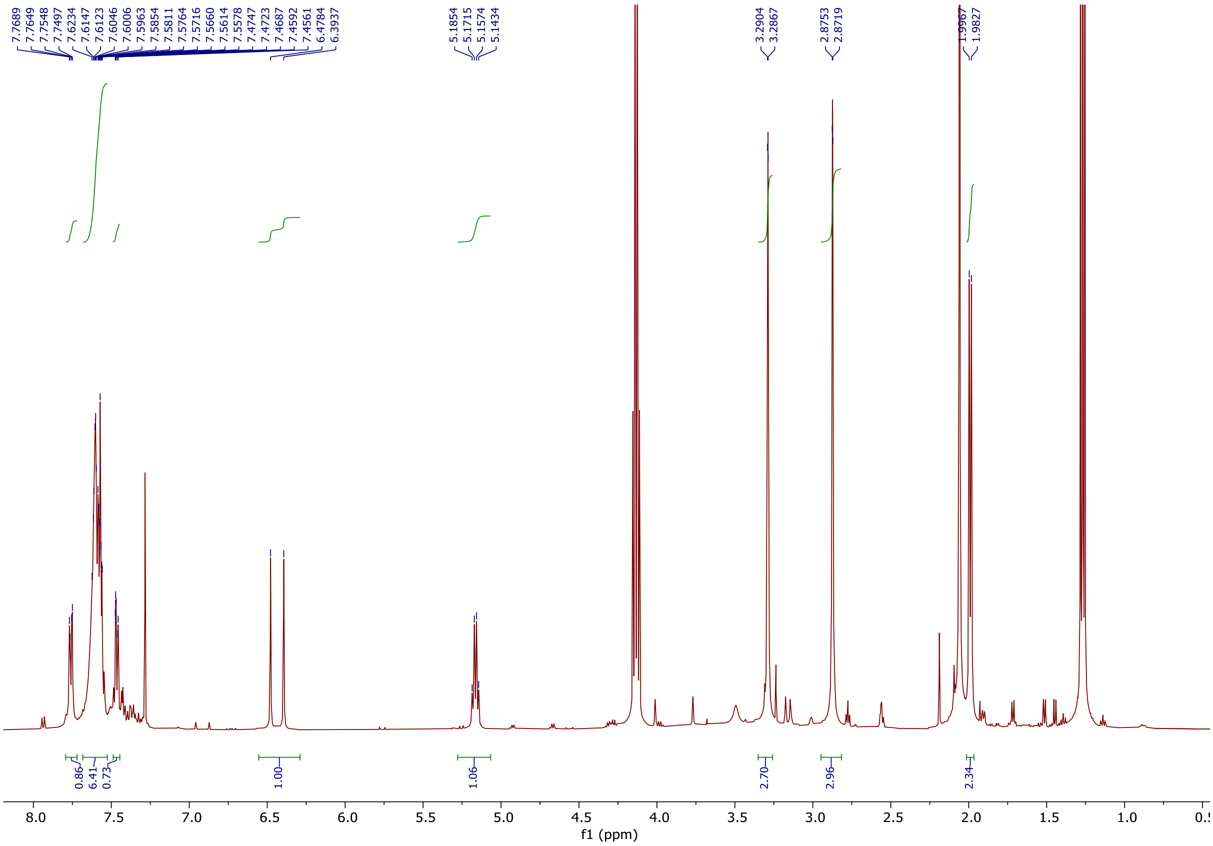

Figure S99. ^{1}H NMR of isolated **2c-[N*s*]** (major isomer) in $CDCl_3$. Residual ethyl acetate at 4.1, 2.0 and 1.4 ppm.

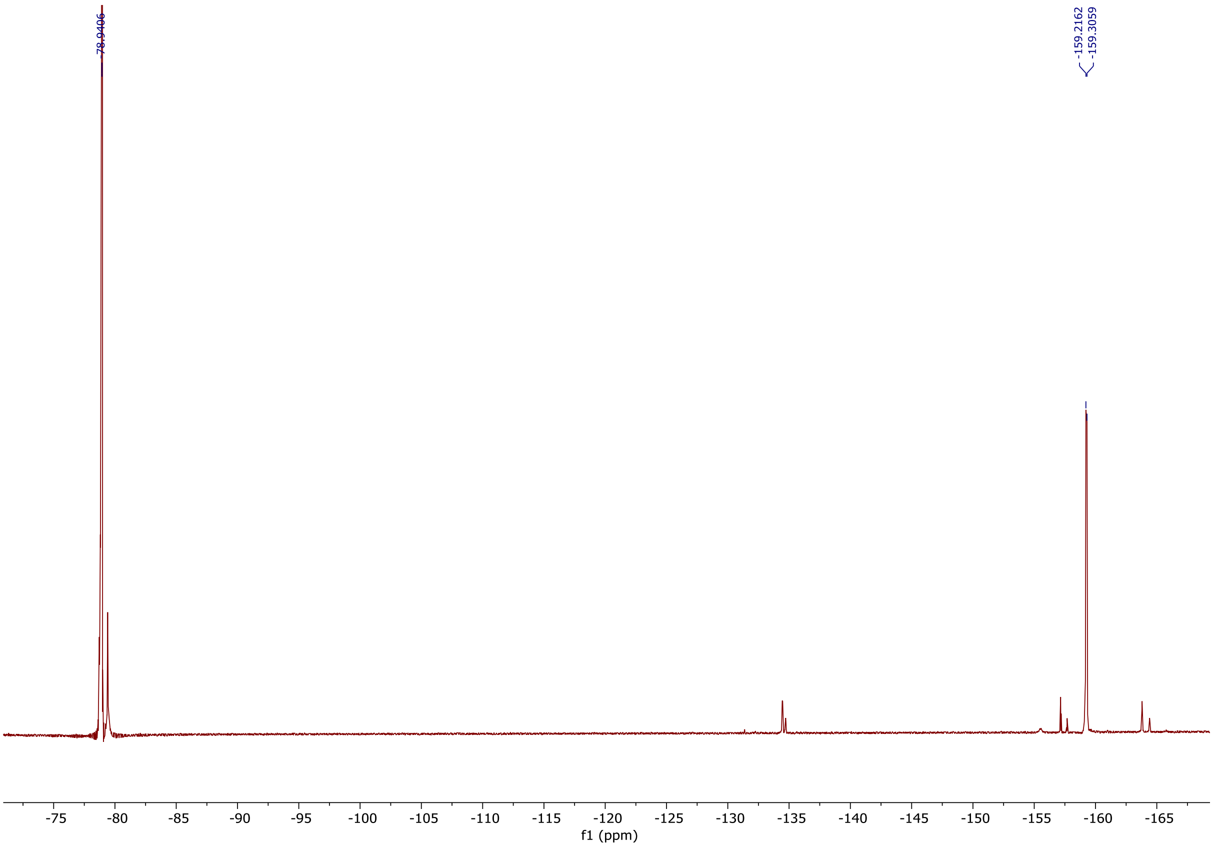

Figure S100. ^{19}F NMR of isolated **2c-[N*s*]** (major isomer) in $CDCl_3$. Minor signals at 135, 156 and 164 ppm due to $[B(OH)(C_6F_5)_3]^-$ or $[(OH)\{B(C_6F_5)_3\}_2]^-$ anions.

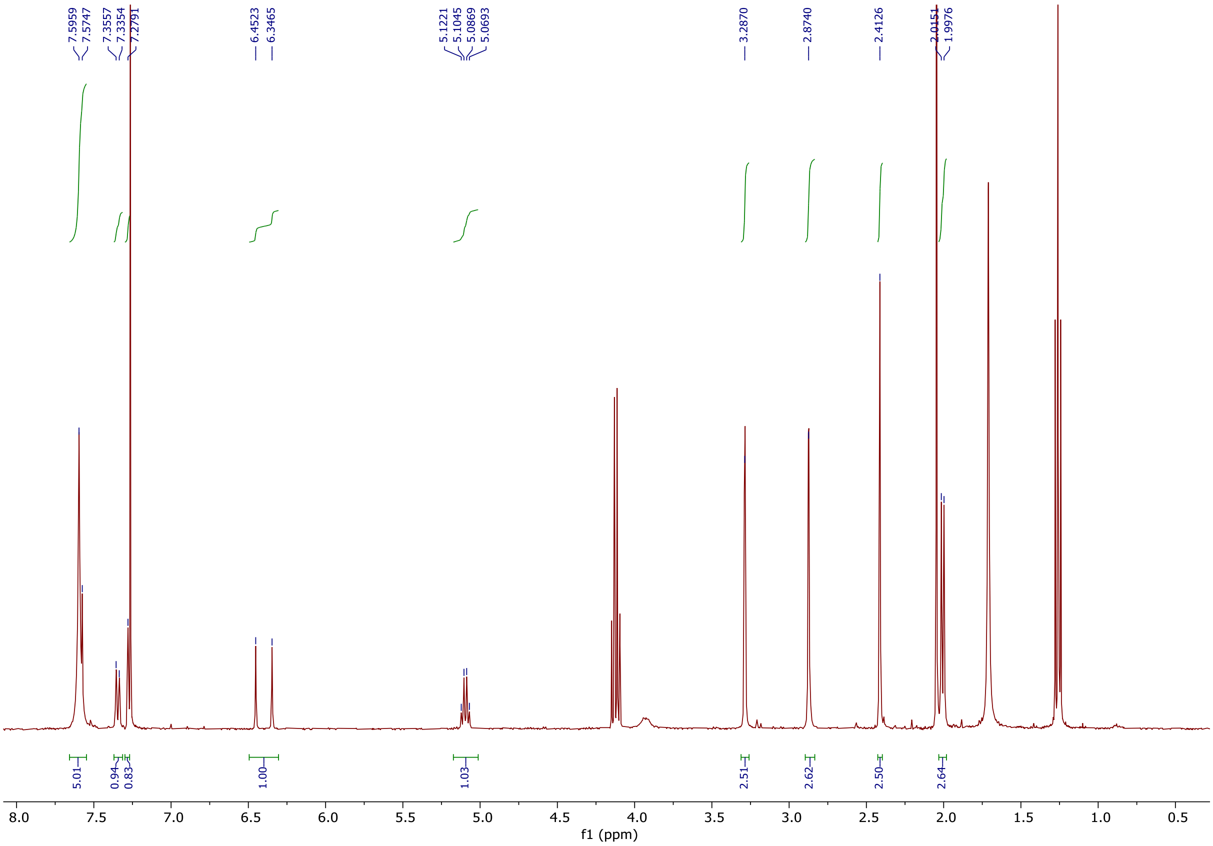

Figure S101. ^{1}H NMR of isolated **2i-[N*S*]** (major isomer) in $CDCl_3$. Residual ethyl acetate and H_2O at 4.1, 2.0, 1.7 and 1.3 ppm.

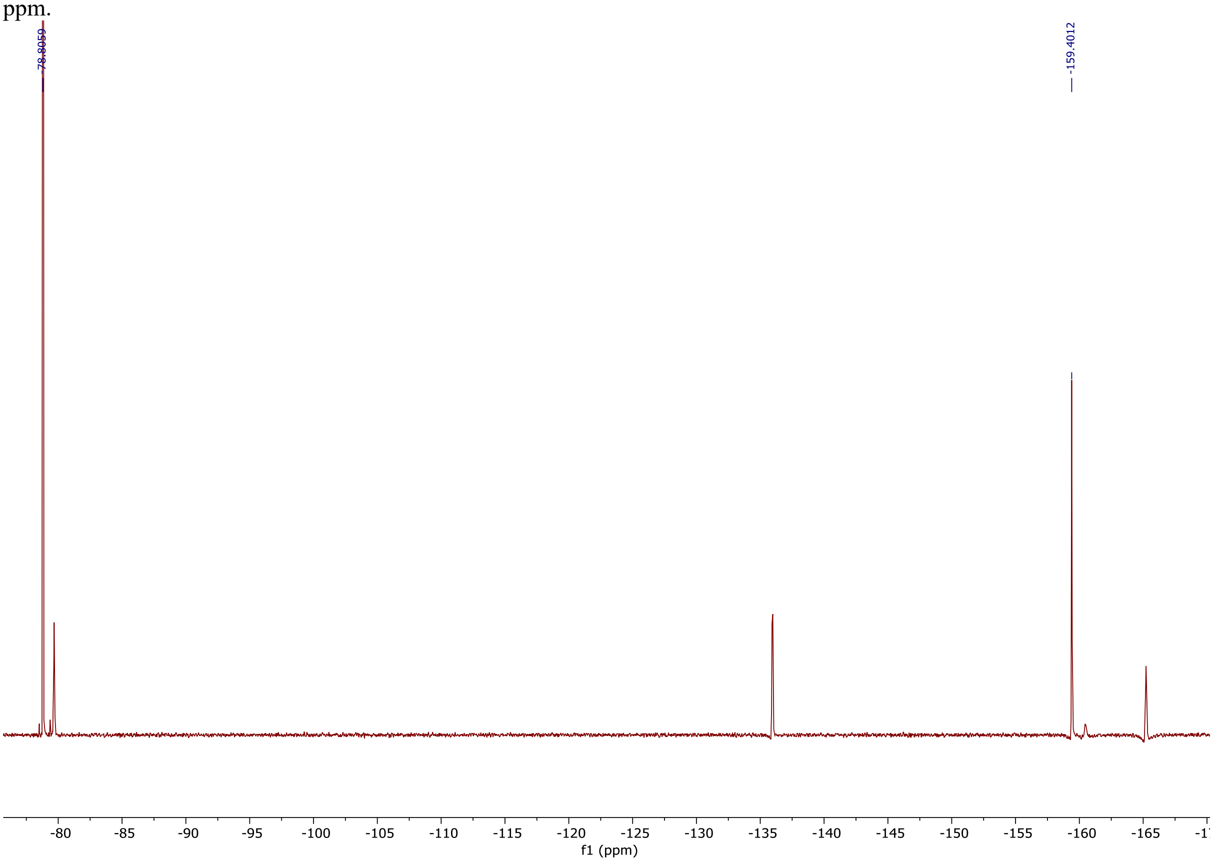

Figure S102. ^{19}F NMR of isolated **2i-[N*S*]** (major isomer) in $CDCl_3$. Minor signals at 136, 160 and 165 ppm due to $[B(OH)(C_6F_5)_3]^-$ or $[(OH)\{B(C_6F_5)_3\}_2]^-$ anions.

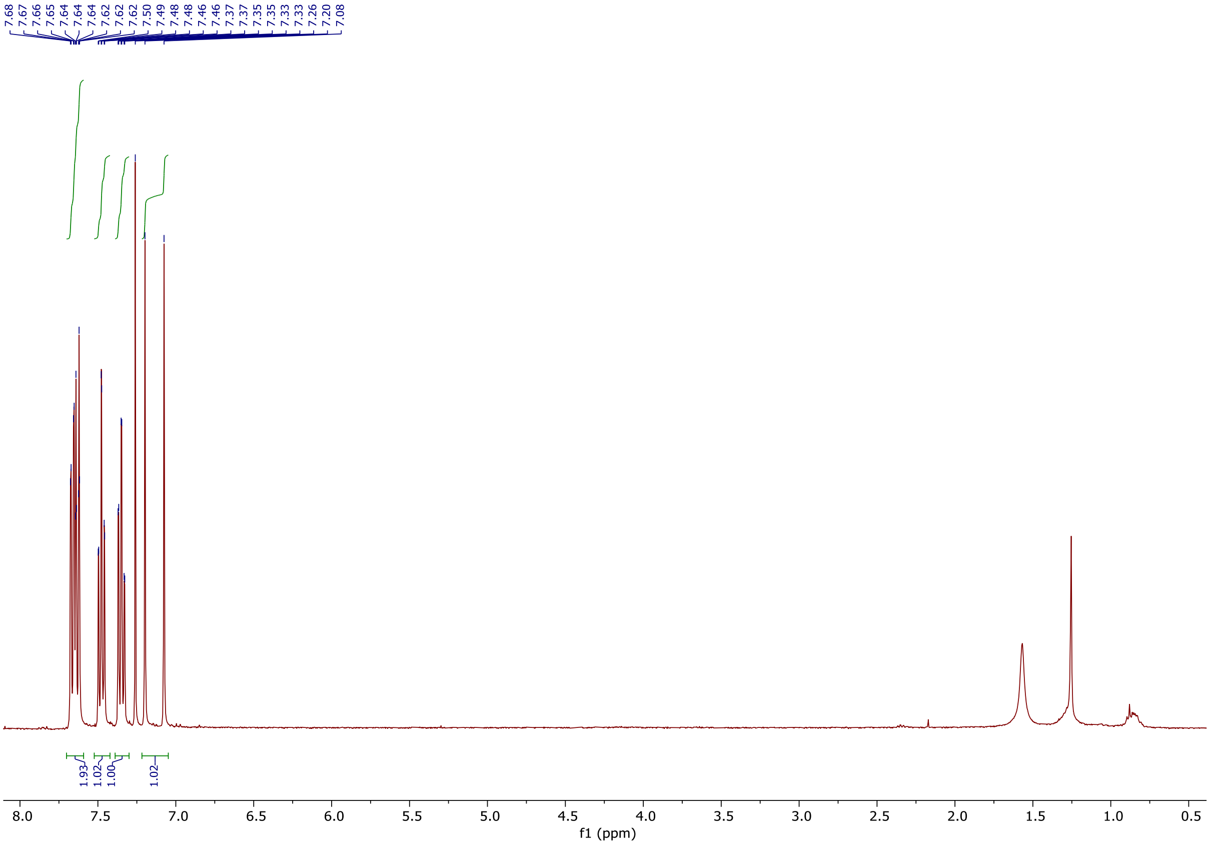

Figure S103. ^{1}H NMR of isolated **2a-[SCN]** in $CDCl_3$.

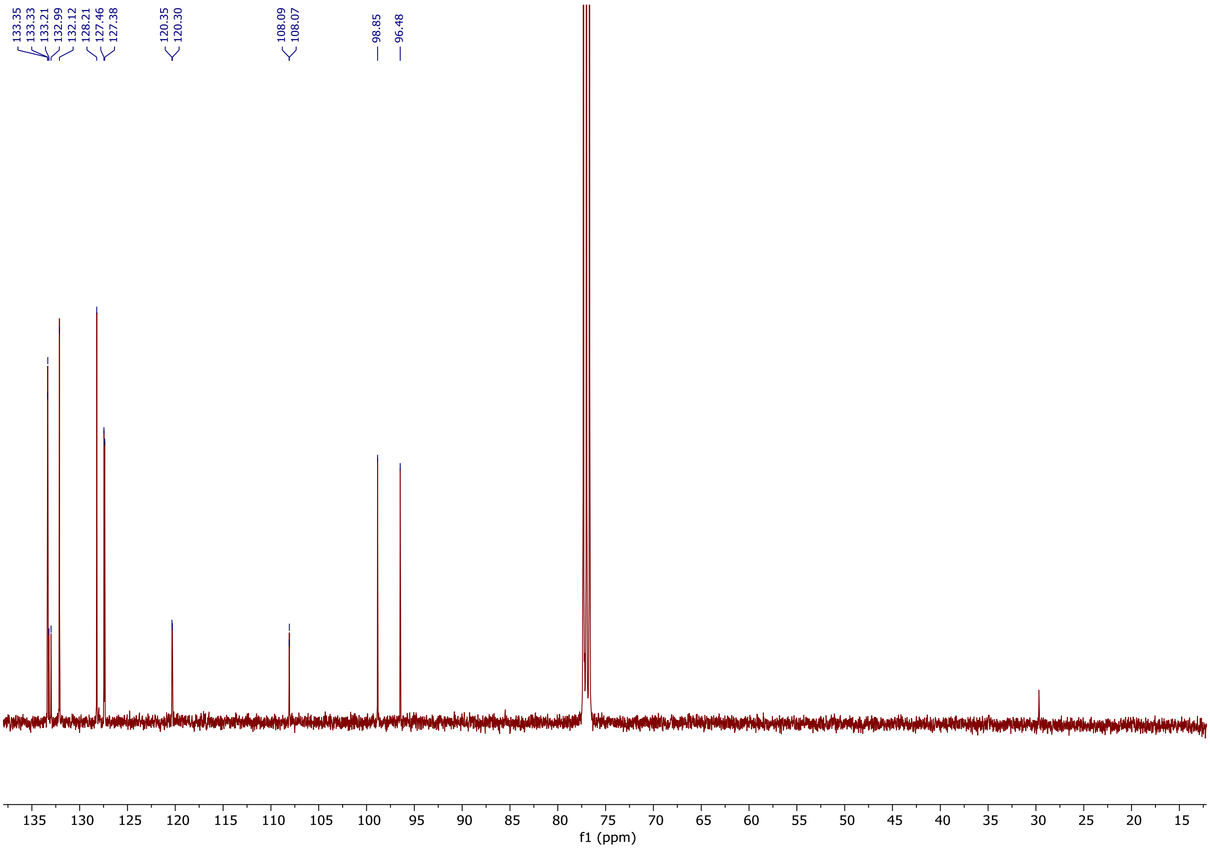

Figure S104. $^{13}C\{^1H\}$ NMR of isolated **2a-[SCN]** in $CDCl_3$.

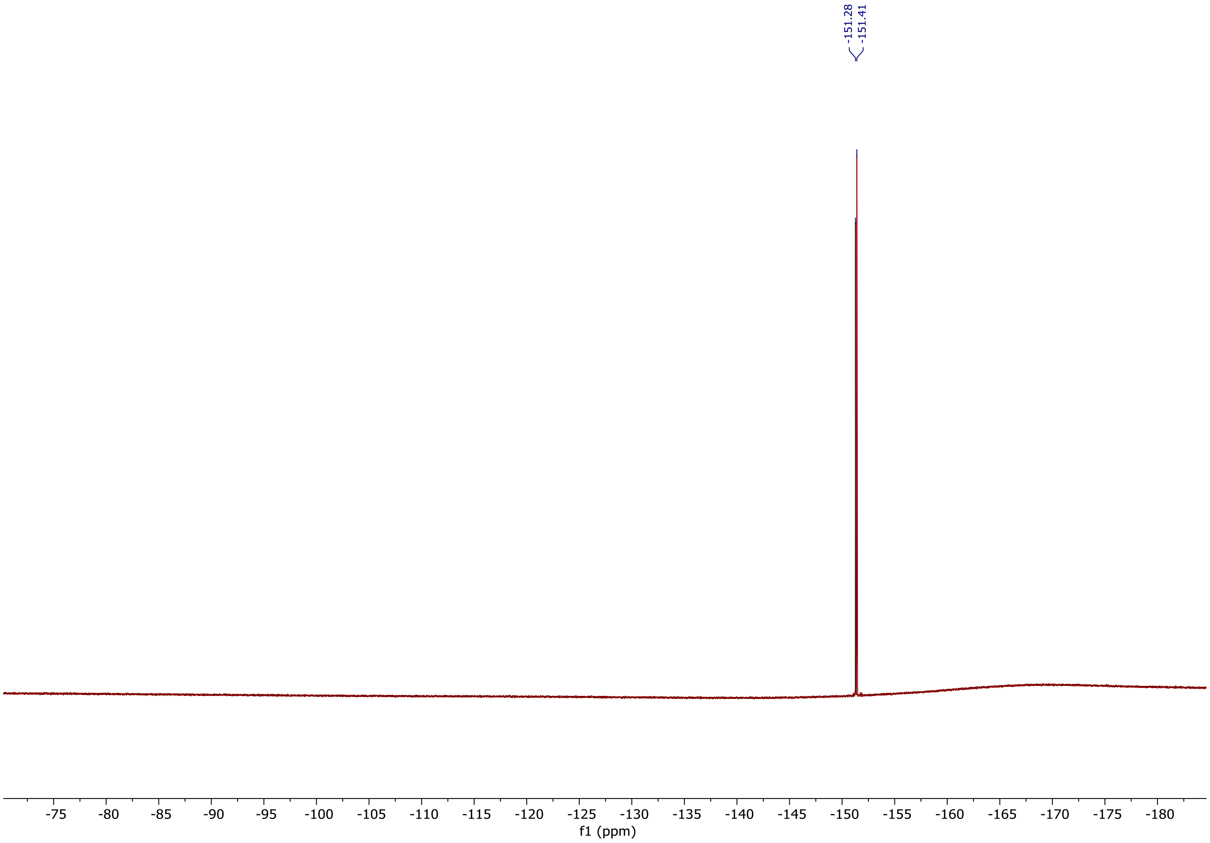

Figure S105. ^{19}F NMR of isolated **2a-[SCN]** in $CDCl_3$.

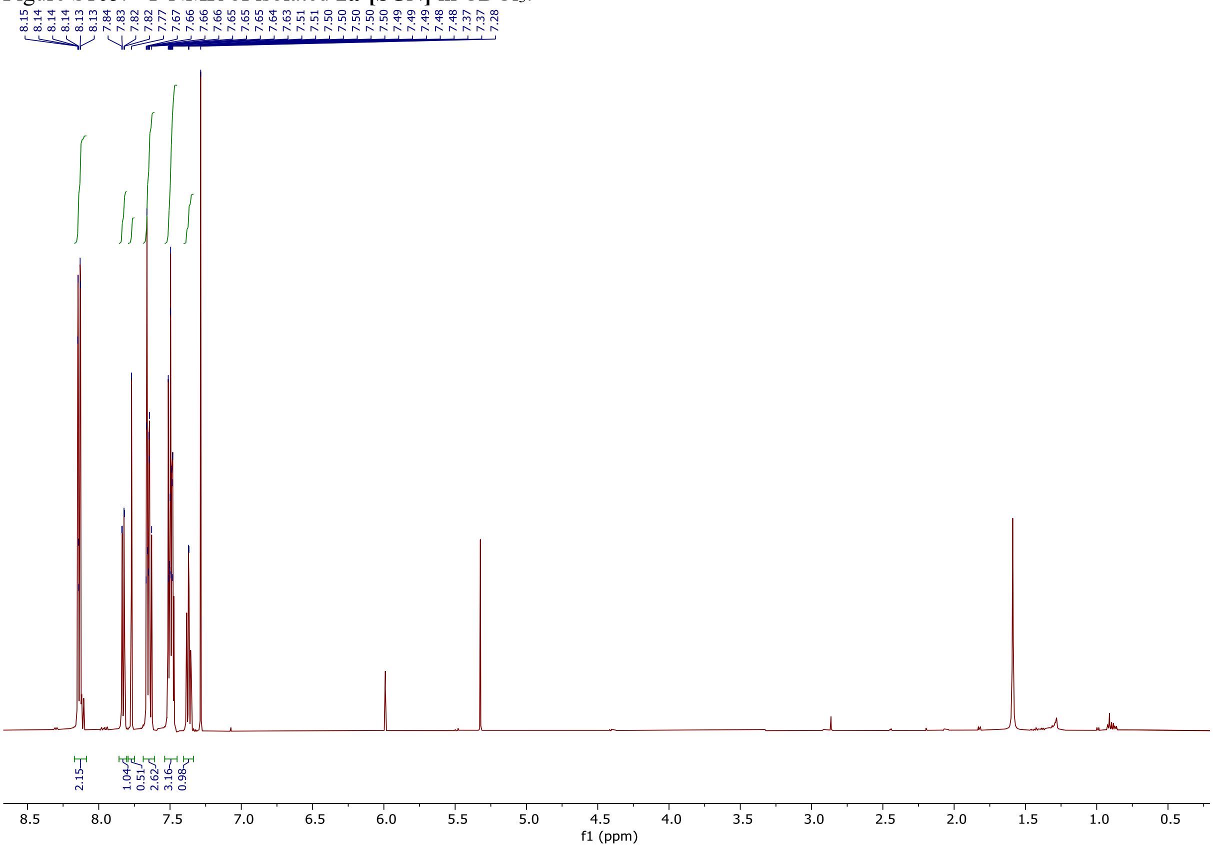

Figure S106. ^{1}H NMR of isolated **2a-[OBz]** in $CDCl_3$.

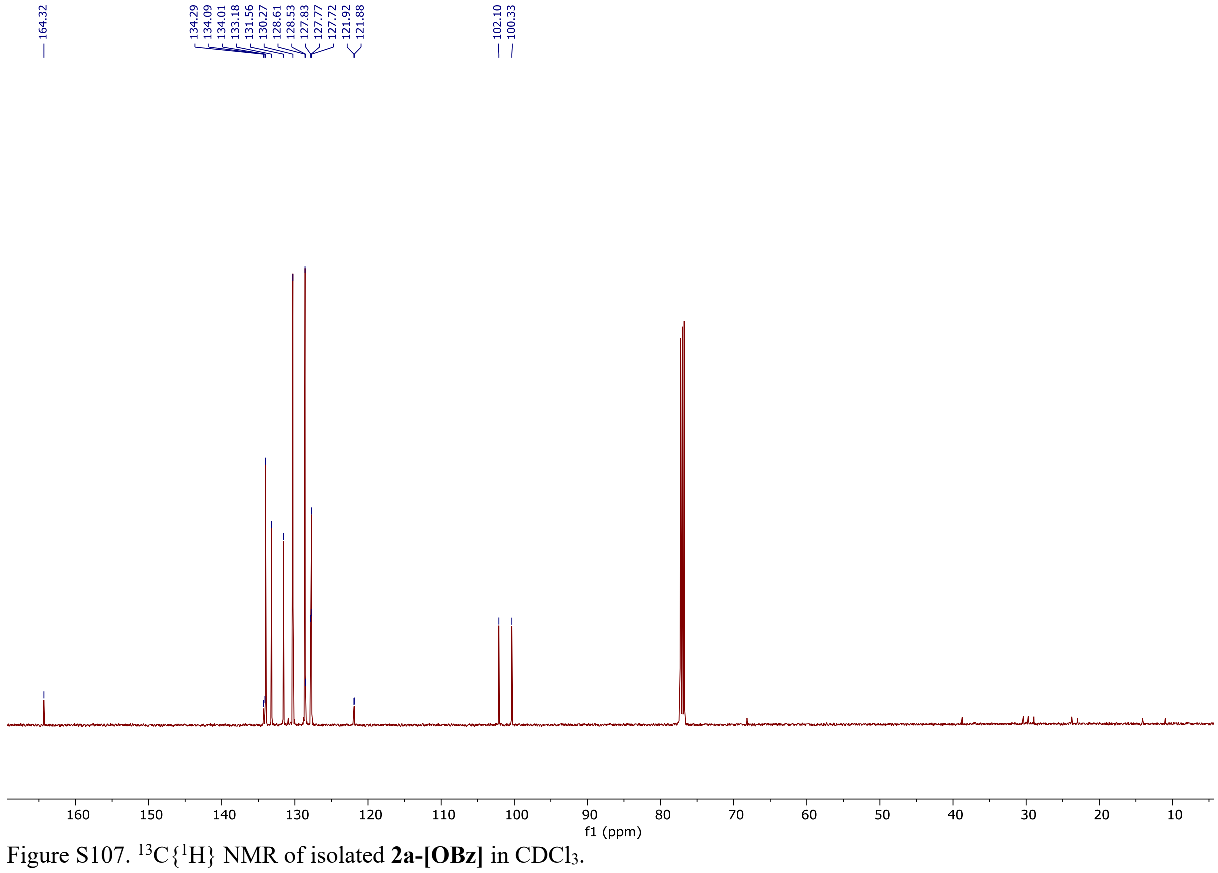

Figure S107. $^{13}C\{^1H\}$ NMR of isolated **2a-[OBz]** in $CDCl_3$.

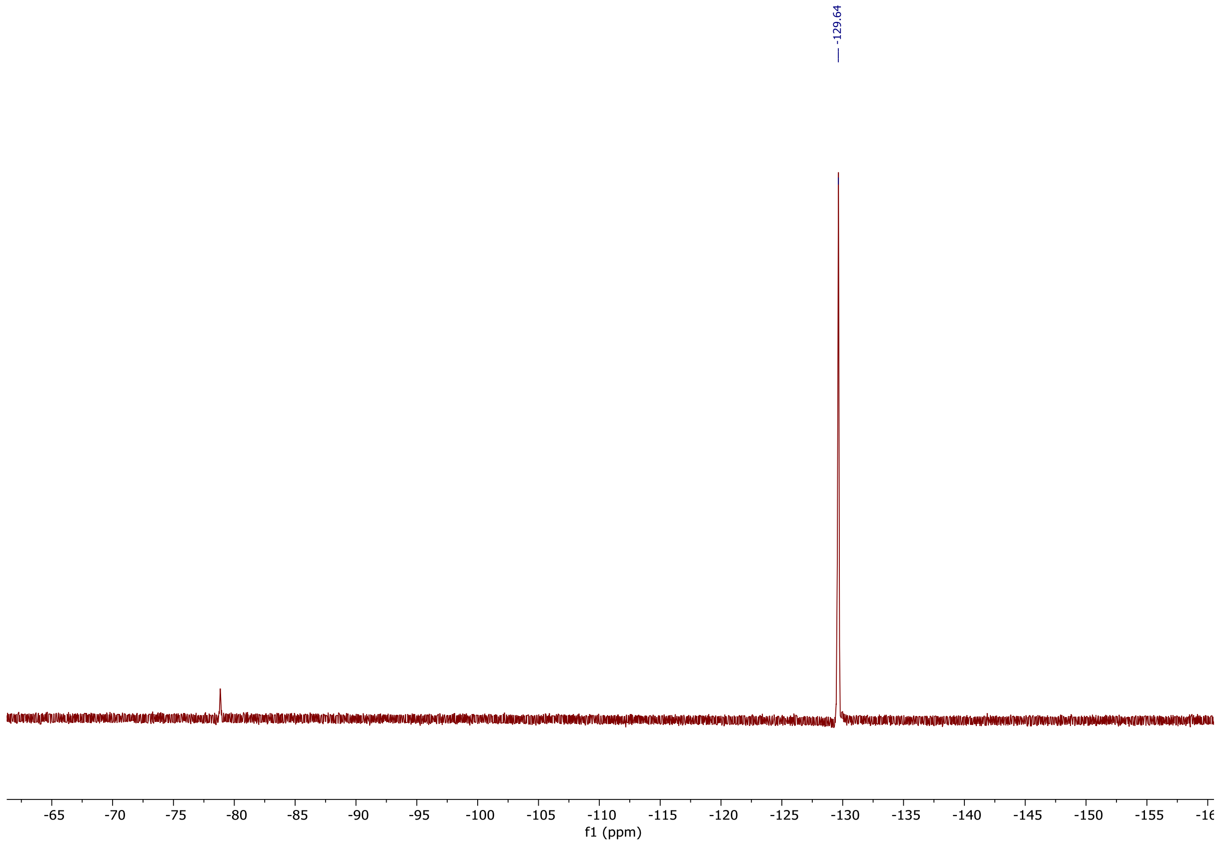

Figure S108. $^{19}F\{^1H\}$ NMR of isolated **2a-[OBz]** in $CDCl_3$.

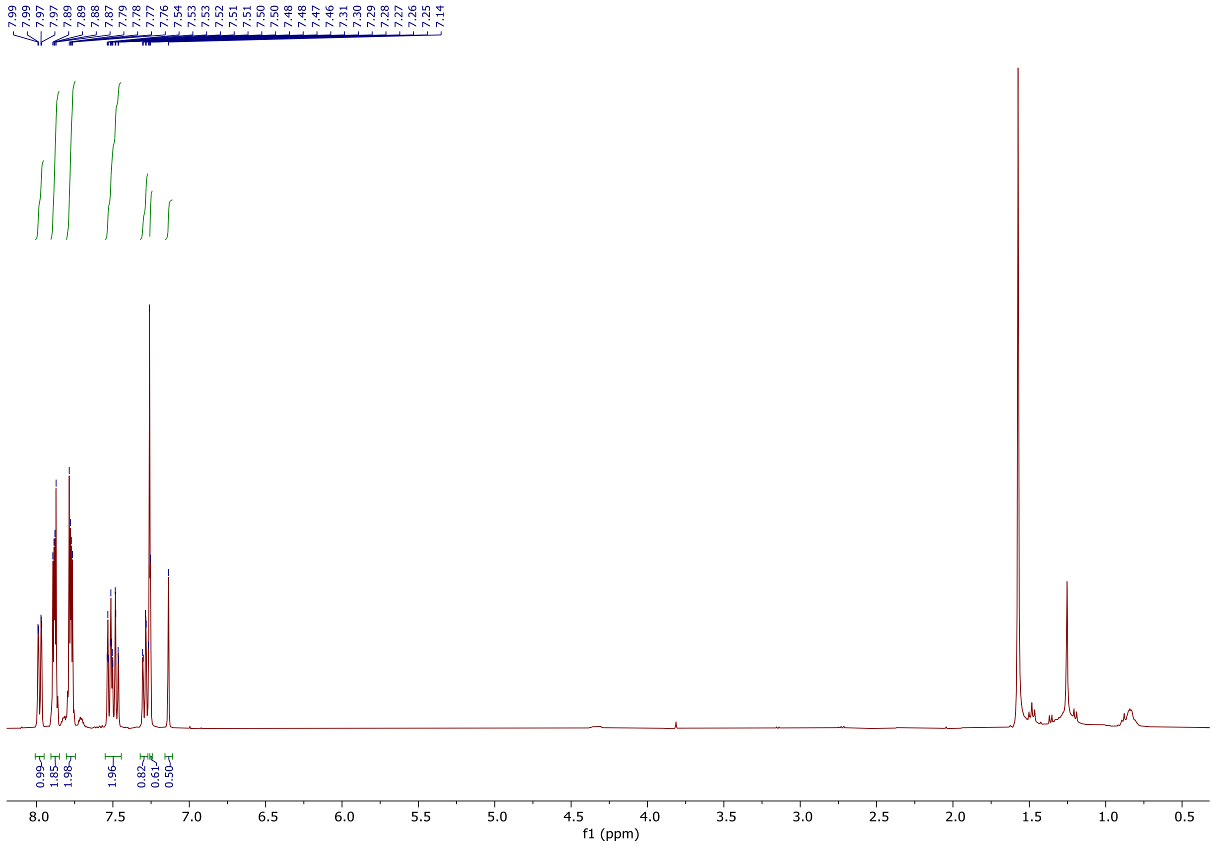

Figure S109. ^{1}H NMR of isolated **2a-[Pth]** in $CDCl_3$.

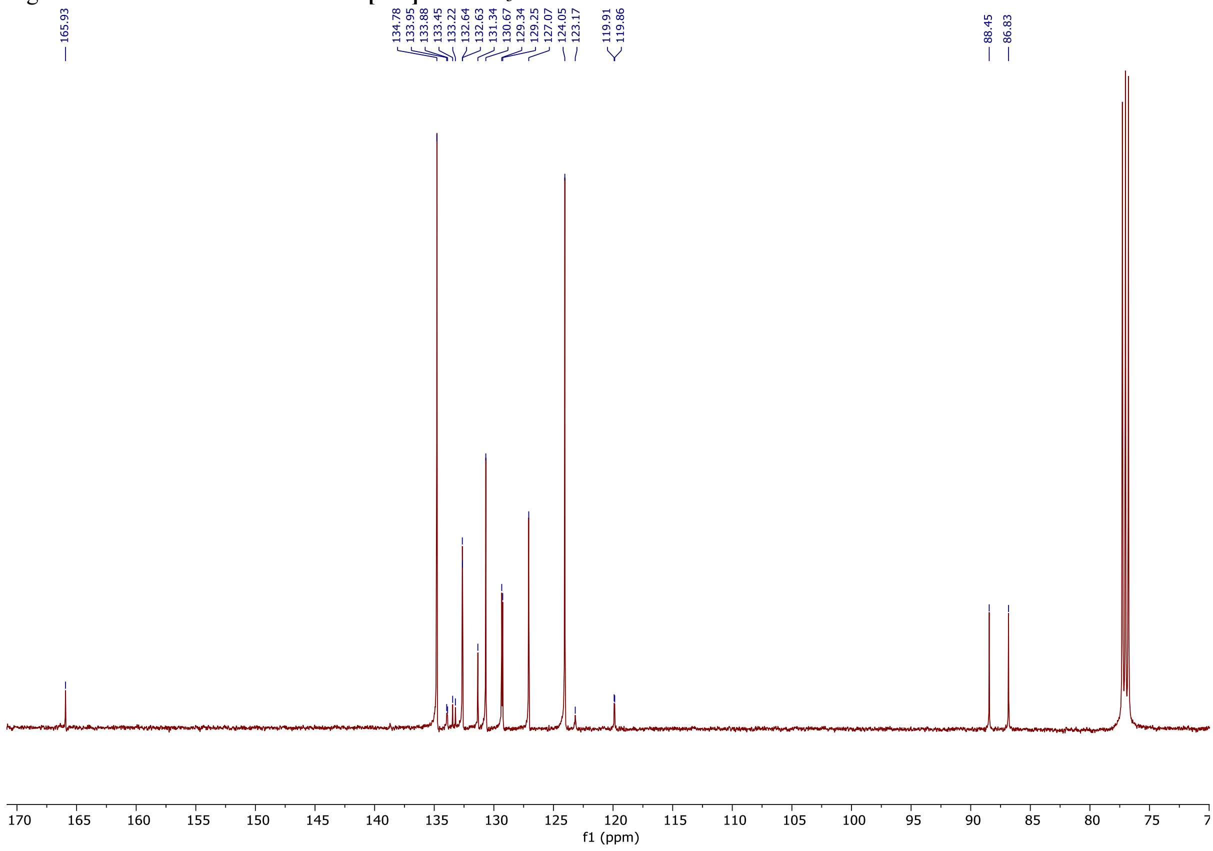

Figure S110. ^{13}C{^{1}H} NMR of isolated **2a-[Pth]** in $CDCl_3$.

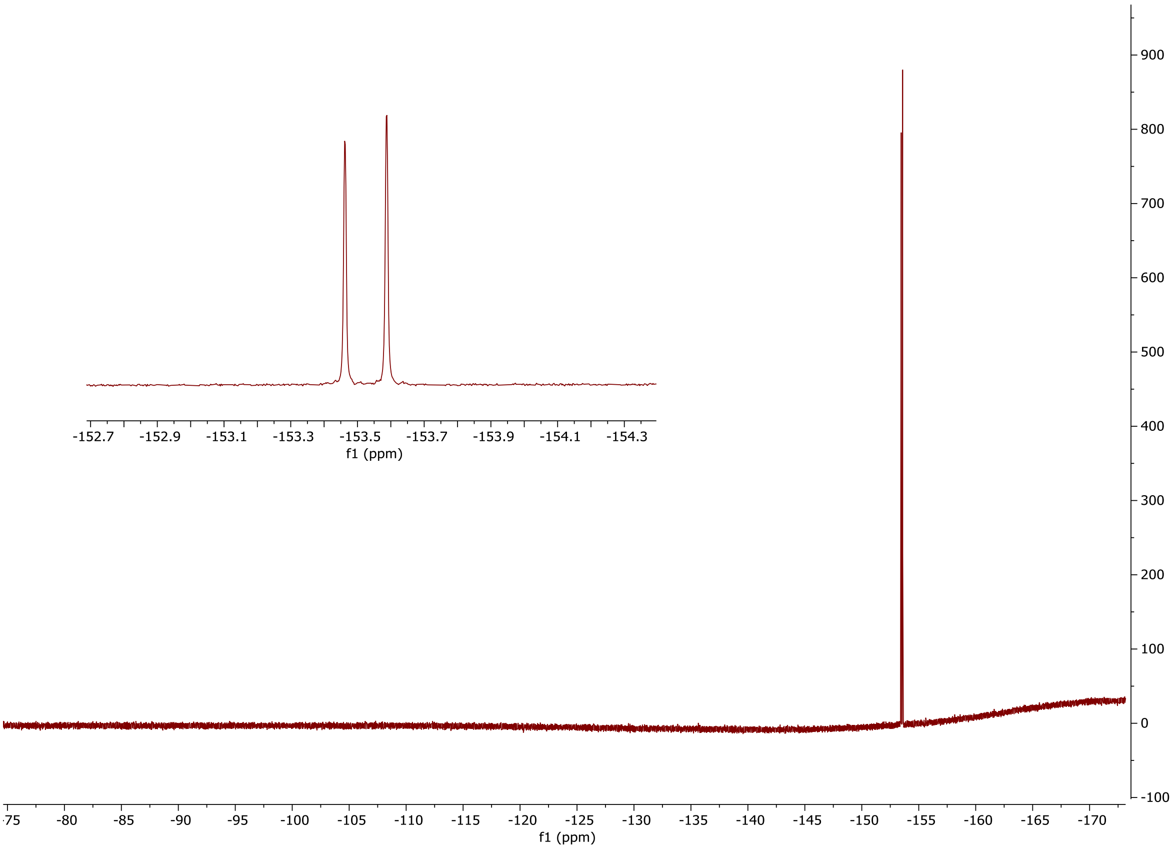

Figure S111. ^{19}F NMR of isolated **2a-[Pth]** in $CDCl_3$.

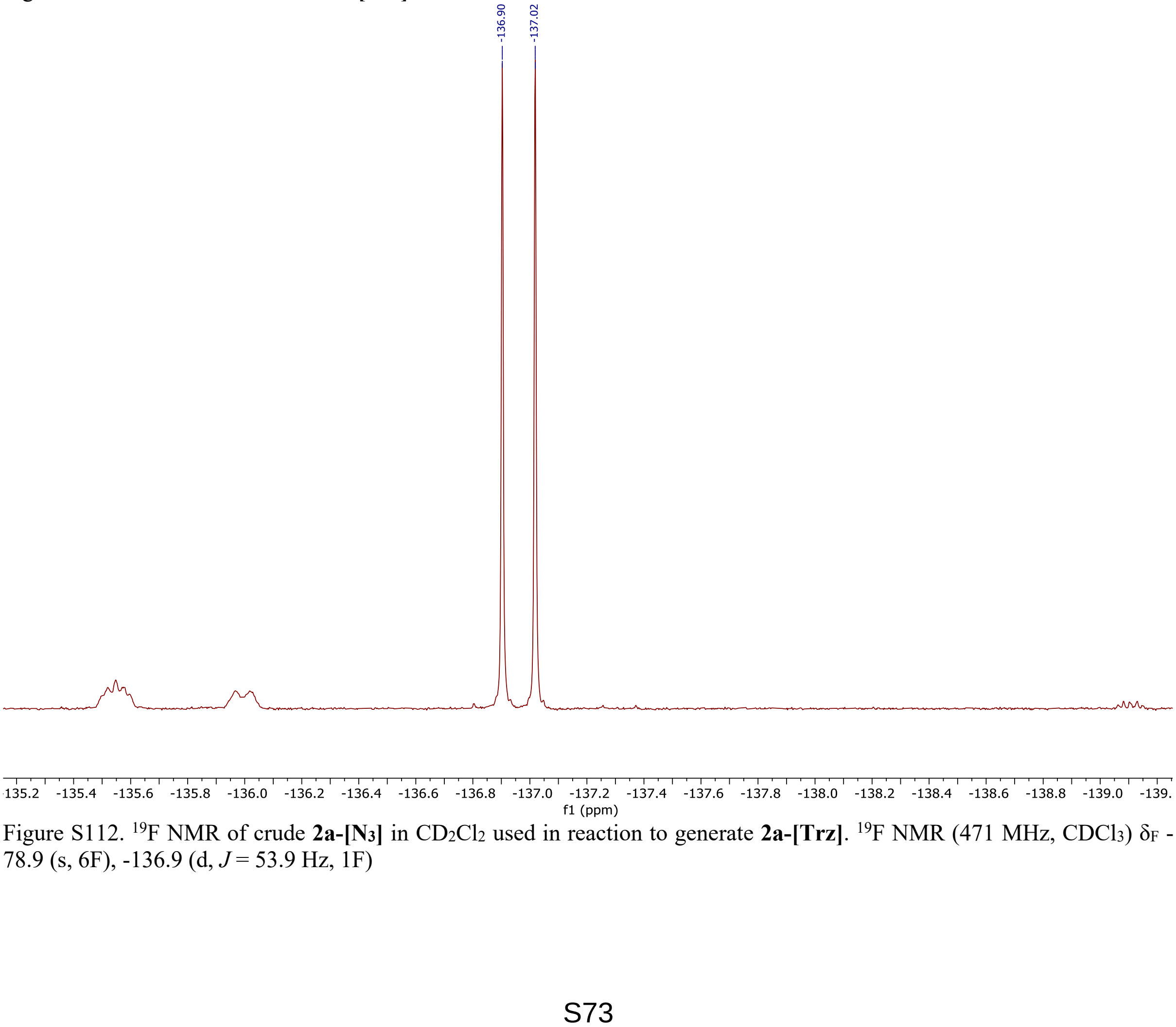

Figure S112. ^{19}F NMR of crude **2a-[N3]** in CD_2Cl_2 used in reaction to generate **2a-[Trz]**. ^{19}F NMR (471 MHz, $CDCl_3$) δ_F -78.9 (s, 6F), -136.9 (d, J = 53.9 Hz, 1F)

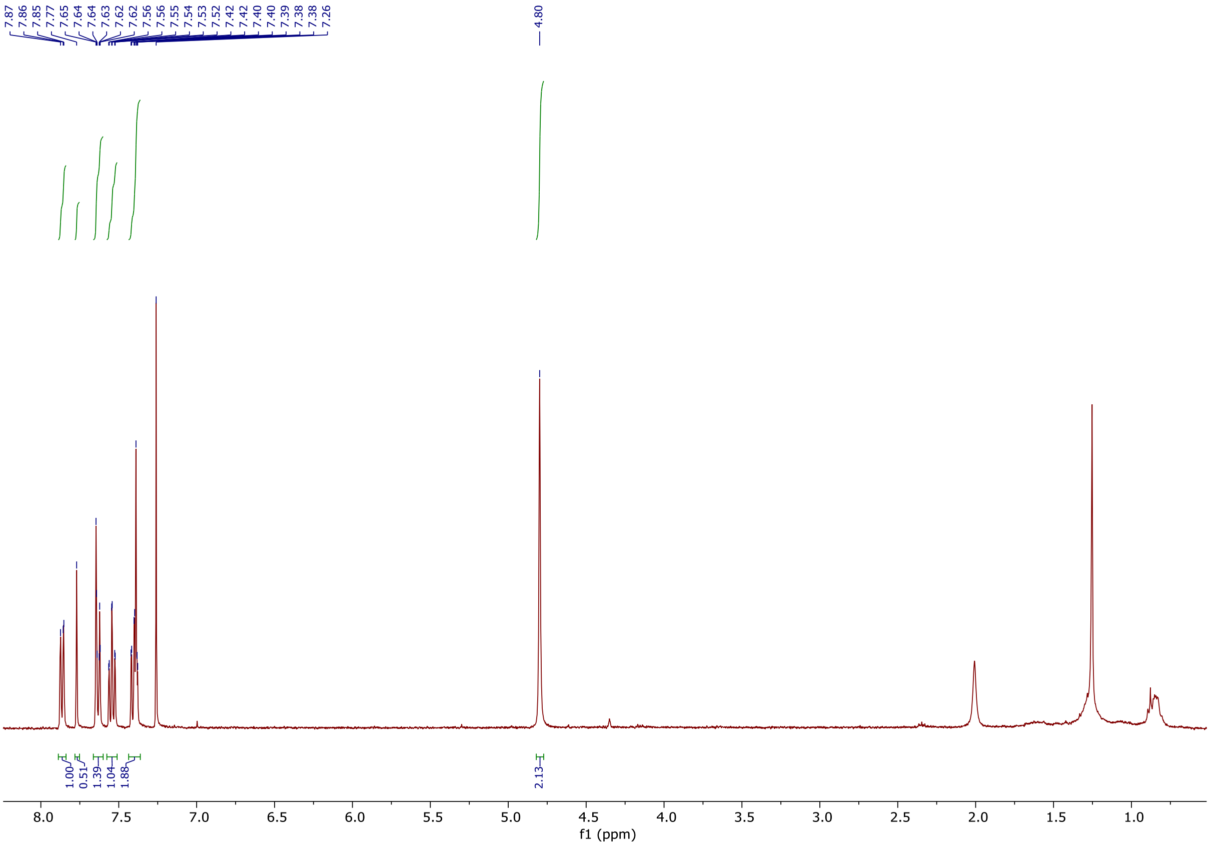

Figure S113. ^{1}H NMR of isolated **2a-[Trz]** in $CDCl_3$.

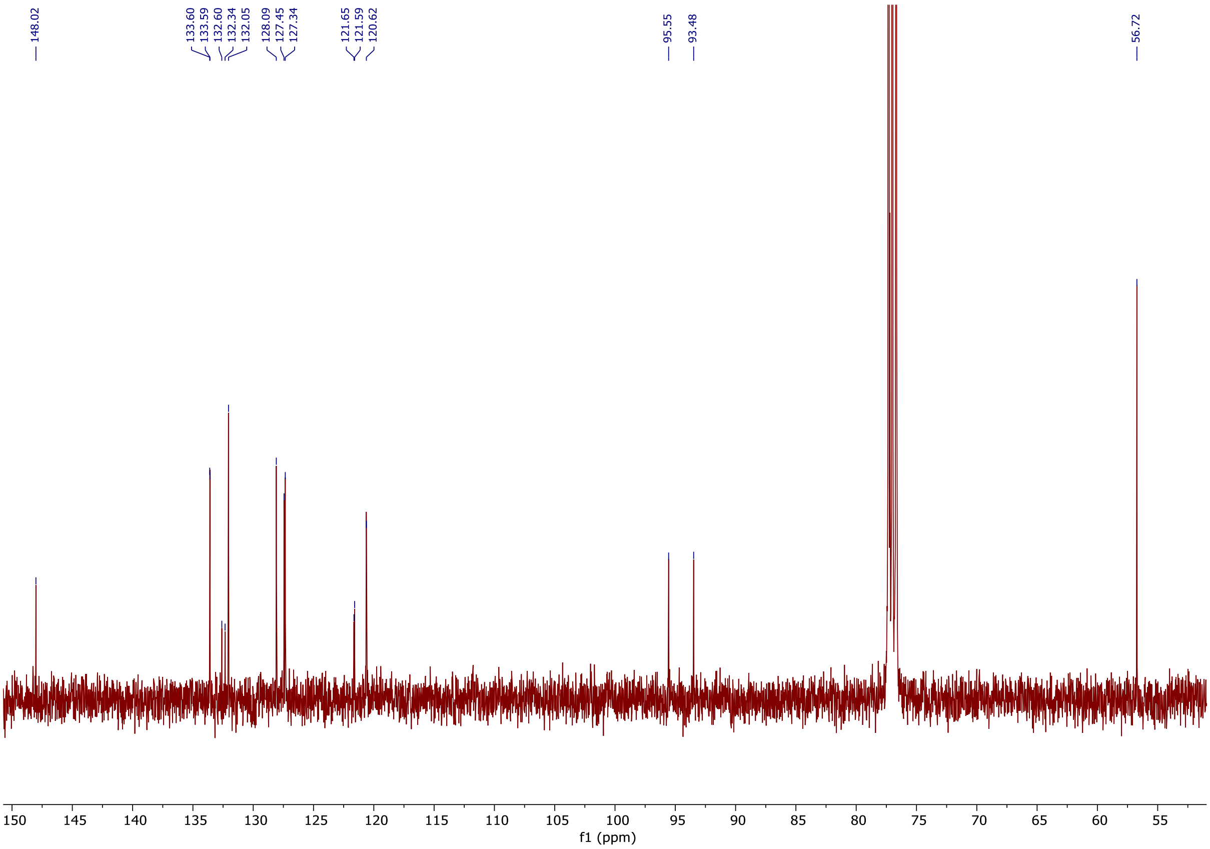

Figure S114. $^{13}C\{^{1}H\}$ NMR of isolated **2a-[Trz]** in $CDCl_3$.

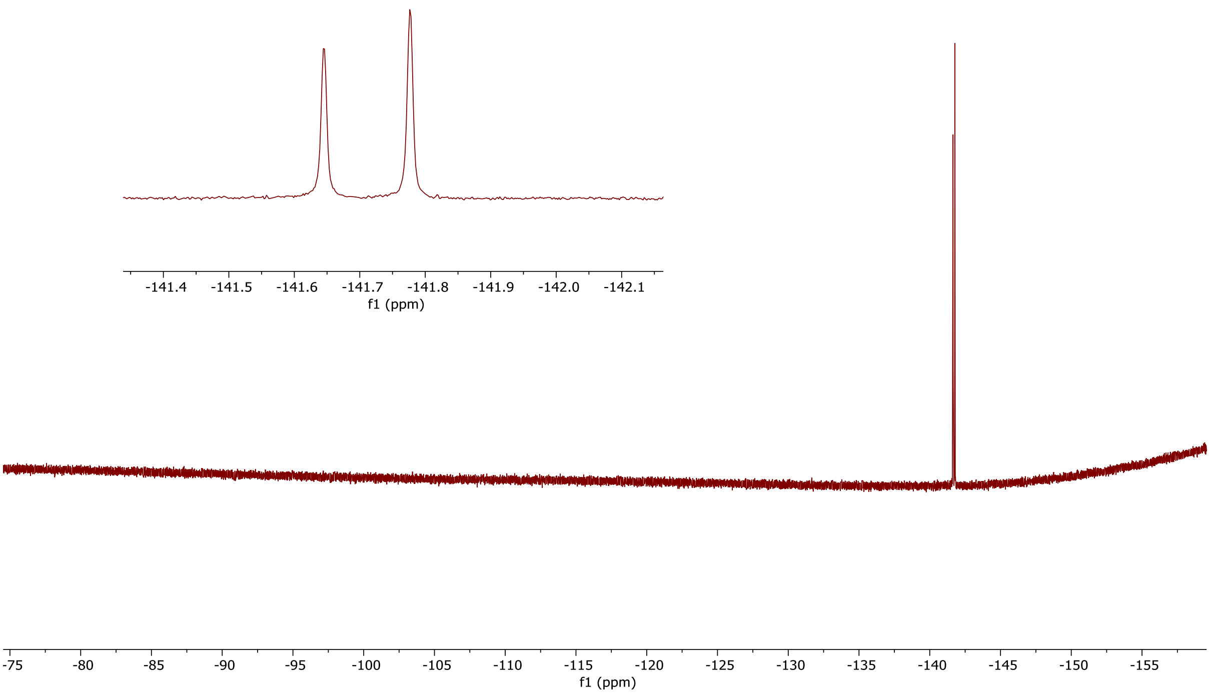

Figure S115. $^{19}F\{^{1}H\}$ NMR of isolated **2a-[Trz]** in $CDCl_3$.

Chiral HPLC traces for compounds 2a-[Nu]

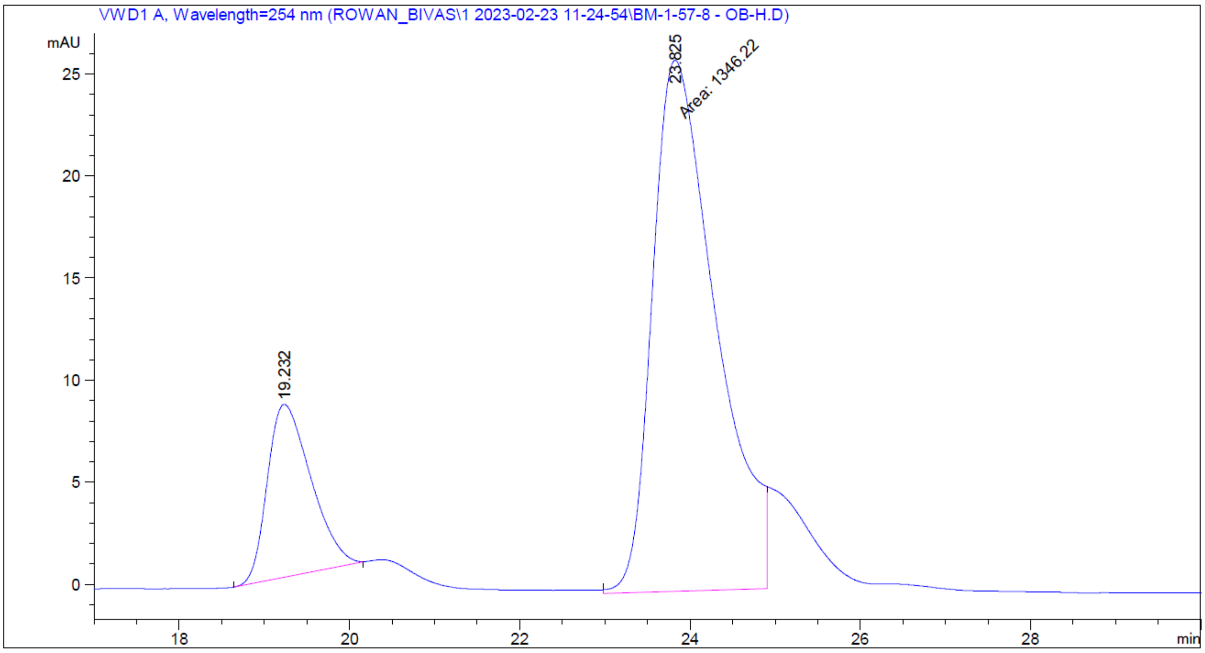

Peak #	RetTime [min]	Type	Width [min]	Area [mAU*s]	Height [mAU]	Area %
1	19.232	BB	0.5464	300.21985	8.47339	18.2344
2	23.825	MF	0.8622	1346.22498	26.02321	81.7656

Figure S116. Chiral HPLC trace for enantioenriched **2a-[SCN]**.

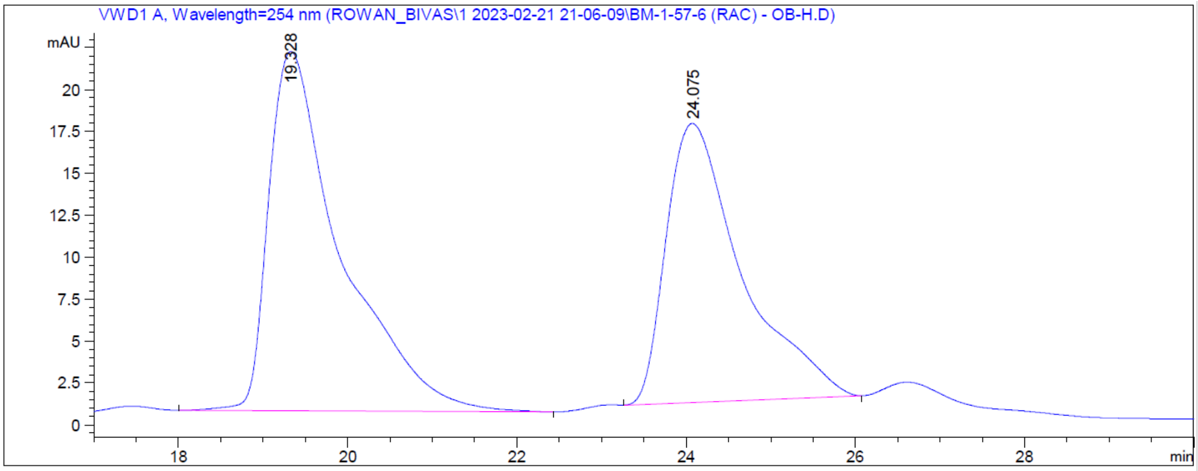

Peak #	RetTime [min]	Type	Width [min]	Area [mAU*s]	Height [mAU]	Area %
1	19.328	BB	0.8562	1270.44666	21.44287	55.2230
2	24.075	BB	0.9067	1030.13037	16.65306	44.7770

Figure S117. Chiral HPLC trace for racemic **2a-[SCN]**.

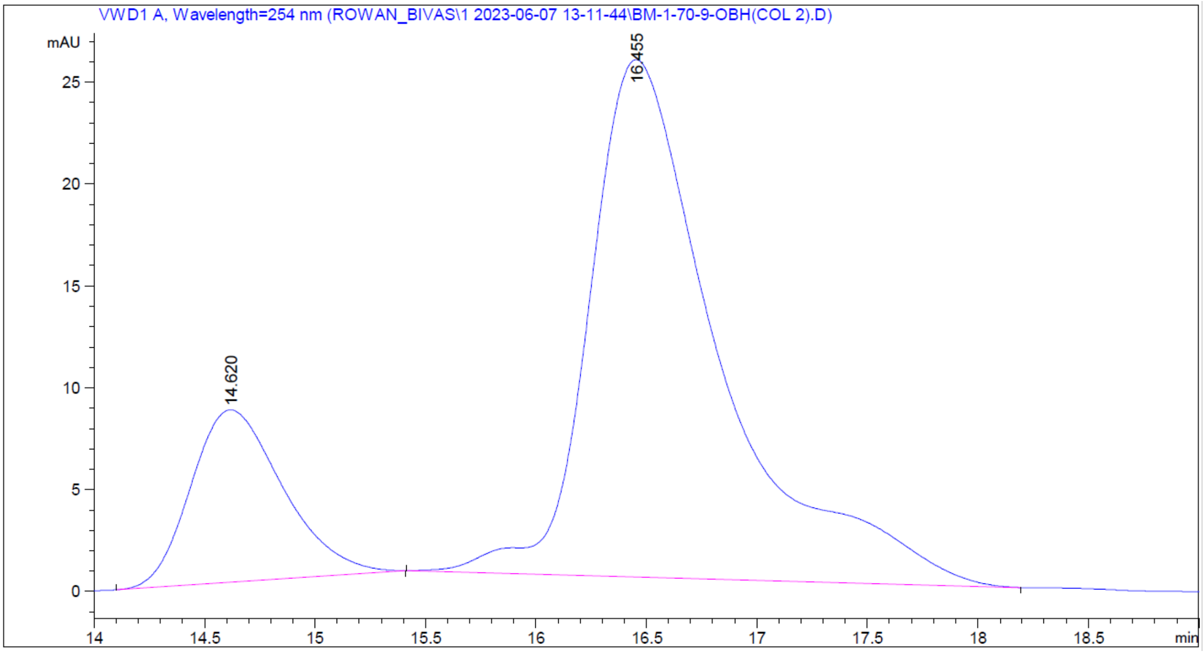

Peak #	RetTime [min]	Type	Width [min]	Area [mAU*s]	Height [mAU]	Area %
1	14.620	BB	0.4462	244.43747	8.45708	18.9547
2	16.455	BB	0.6023	1045.15259	25.38726	81.0453

Figure S118. Chiral HPLC trace for enantioenriched **2a-[OBz]**.

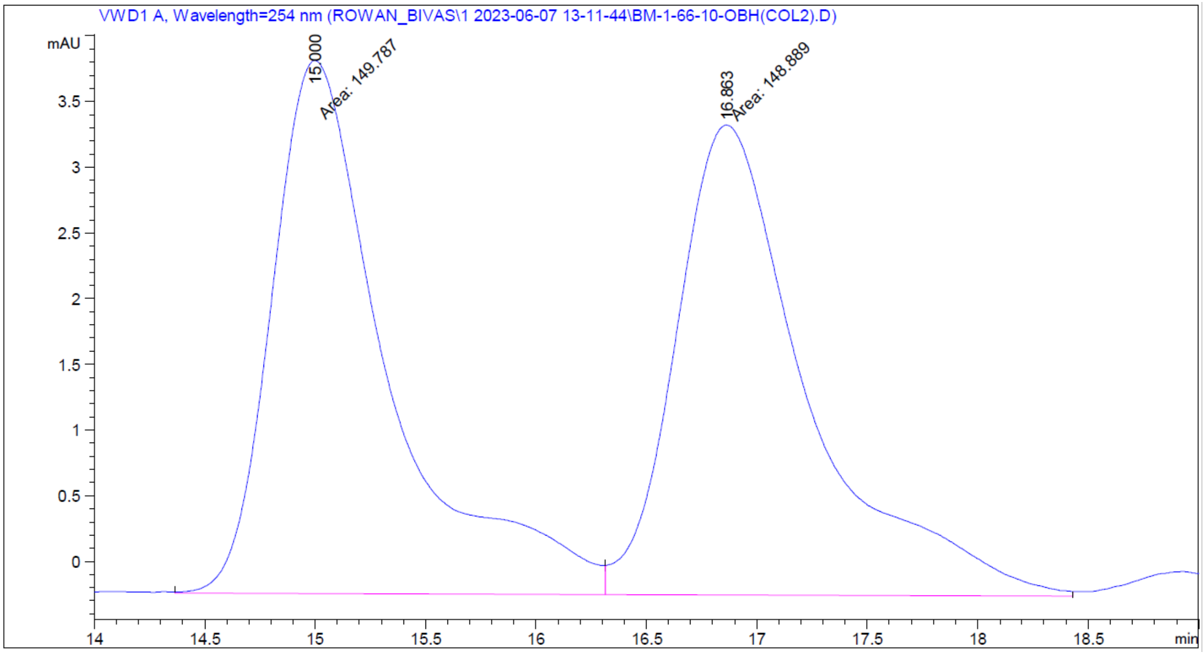

Peak #	RetTime [min]	Type	Width [min]	Area [mAU*s]	Height [mAU]	Area %
1	15.000	MF	0.6152	149.78687	4.05782	50.1502
2	16.863	FM	0.6938	148.88943	3.57680	49.8498

Figure S119. Chiral HPLC trace for racemic **2a-[OBz]**.

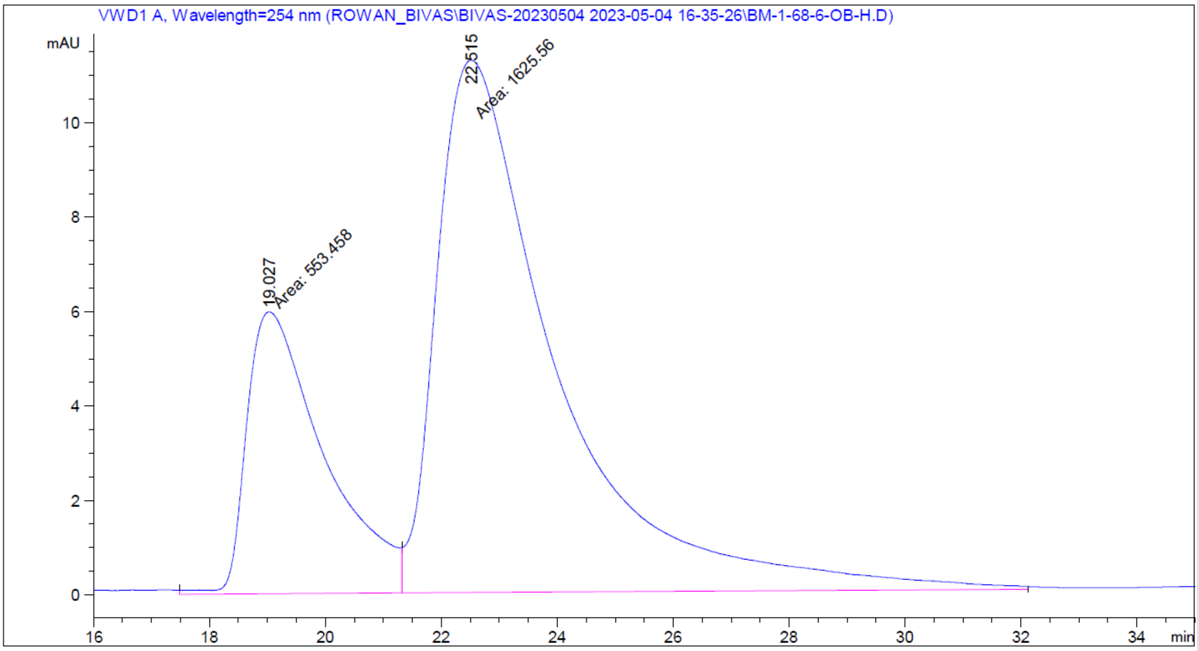

Peak #	RetTime [min]	Type	Width [min]	Area [mAU*s]	Height [mAU]	Area %
1	19.027	MF	1.5453	553.45776	5.96922	25.3994
2	22.515	FM	2.4028	1625.55713	11.27527	74.6006

Figure S120. Chiral HPLC trace for enantioenriched **2a-[Pth]**.

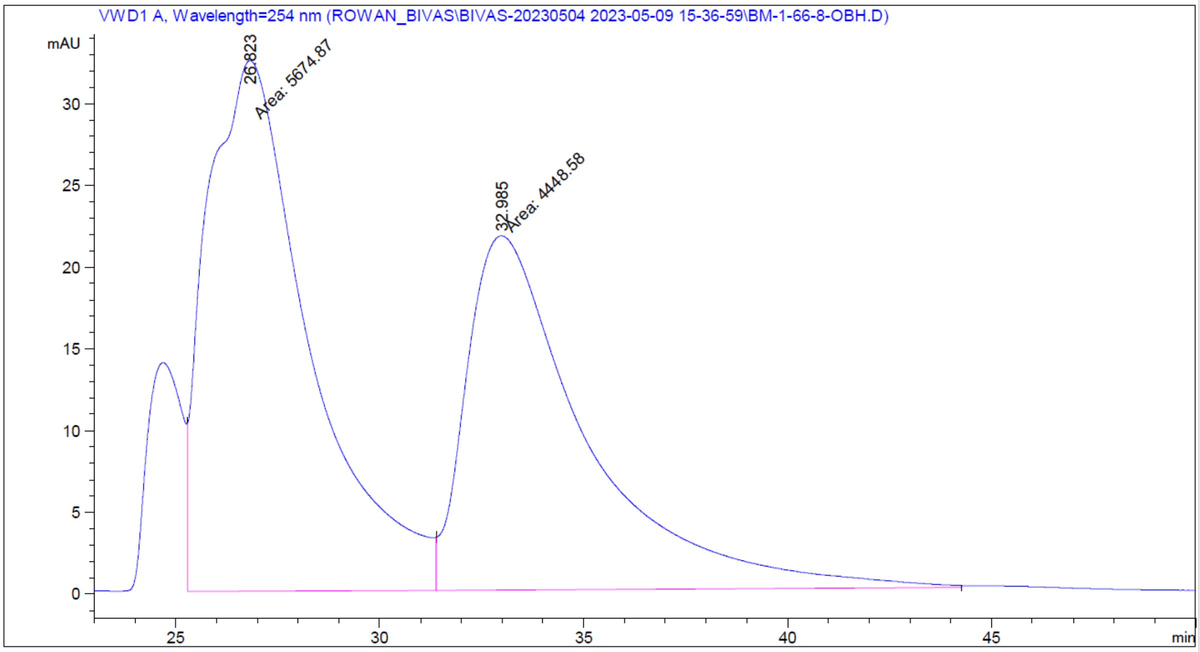

Peak #	RetTime [min]	Type	Width [min]	Area [mAU*s]	Height [mAU]	Area %
1	26.823	MF	2.9143	5674.87207	32.45425	56.0567
2	32.985	FM	3.4239	4448.57861	21.65431	43.9433

Figure S121. Chiral HPLC trace for racemic **2a-[Pth]**.

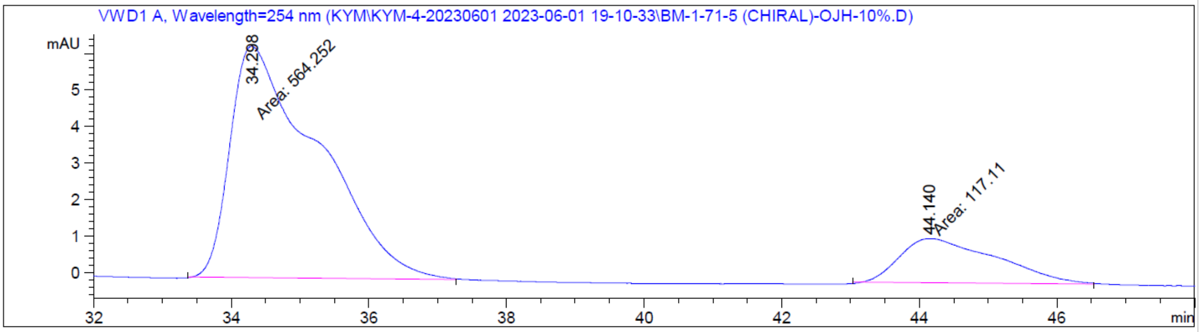

Peak #	RetTime [min]	Type	Width [min]	Area [mAU*s]	Height [mAU]	Area %
1	34.298	MM	1.4823	564.25208	6.34421	82.8124
2	44.140	MM	1.6183	117.10983	1.20611	17.1876

Figure S122. Chiral HPLC trace for enantioenriched **2a-[Trz]**.

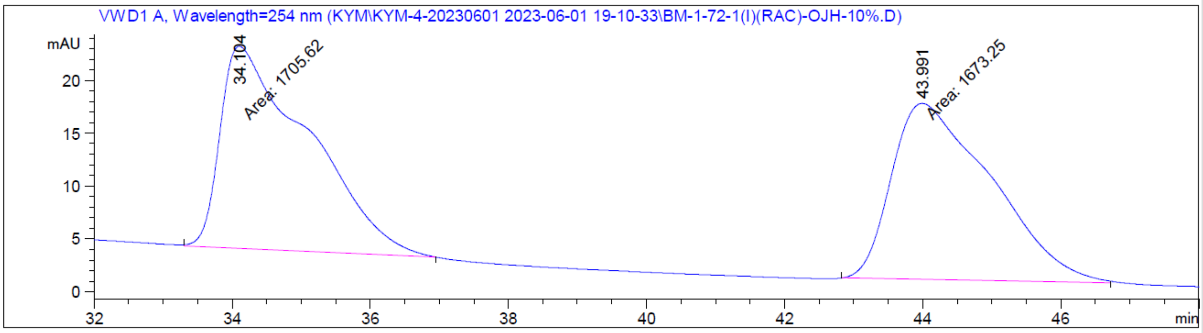

Peak #	RetTime [min]	Type	Width [min]	Area [mAU*s]	Height [mAU]	Area %
1	34.104	MM	1.4867	1705.62329	19.12133	50.4791
2	43.991	MM	1.6744	1673.25024	16.65547	49.5209

Figure S123. Chiral HPLC trace for racemic **2a-[Trz]**.

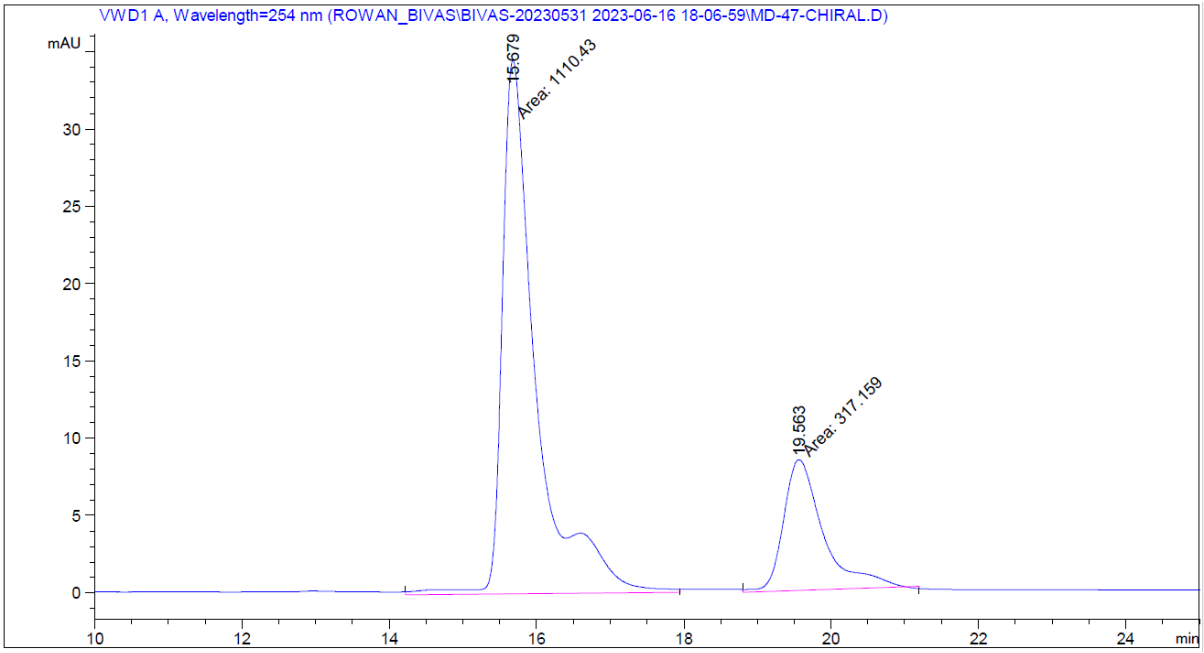

Peak #	RetTime [min]	Type	Width [min]	Area [mAU*s]	Height [mAU]	Area %
1	15.679	MM	0.5363	1110.43347	34.51038	77.7836
2	19.563	MM	0.6253	317.15939	8.45292	22.2164

Figure S124. Chiral HPLC trace for enantioenriched **2b-[OBz]**.

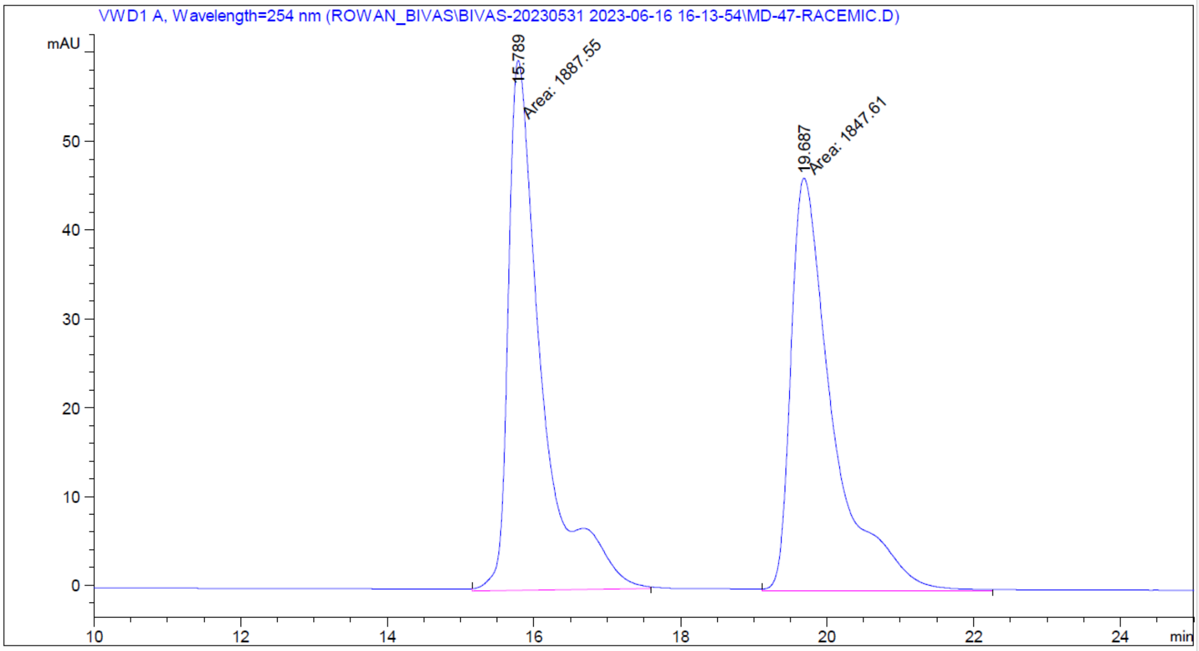

Peak #	RetTime [min]	Type	Width [min]	Area [mAU*s]	Height [mAU]	Area %
1	15.789	MM	0.5274	1887.54590	59.65335	50.5346
2	19.687	MM	0.6625	1847.61145	46.47958	49.4654

Figure S125. Chiral HPLC trace for racemic **2b-[OBz]**.

Mass Spectra

Meas. m/z	#	Formula	Calc. Mass	Err [ppm]
303.0212	1	C13 H17 Br F S	303.0213	0.33

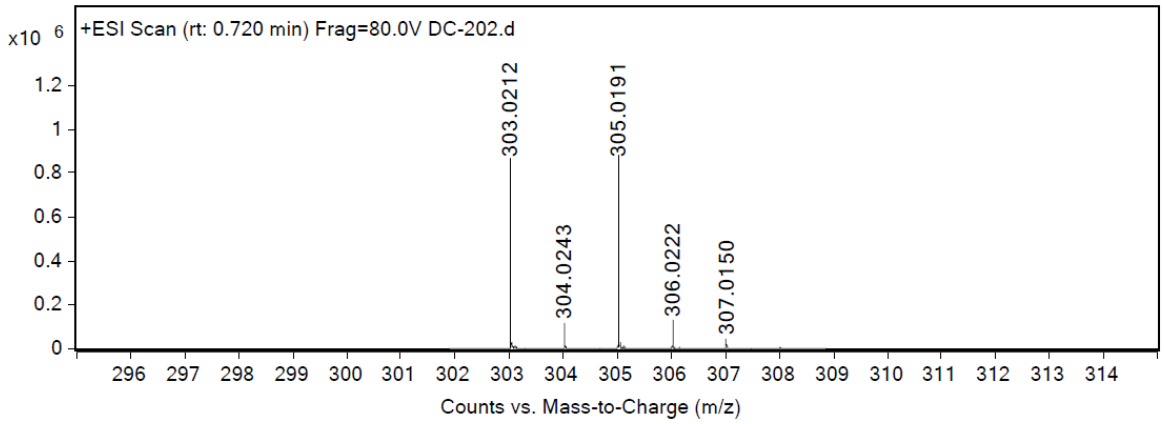

Figure S126. HR-ESI-MS spectrum of **2a-[A]** cationic fragment (positive mode).

Meas. m/z	#	Formula	Calc. Mass	Err [ppm]
336.0756	1	C17 H20 Br F N	336.0758	0.60

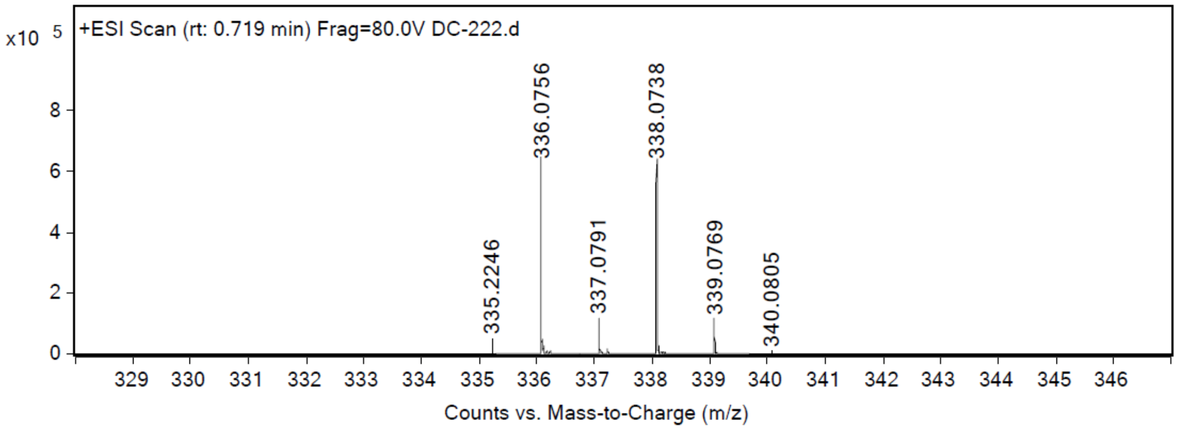

Figure S127. HR-ESI-MS spectrum of **2a-[N*s*]** cationic fragment (positive mode).

Meas. m/z	#	Formula	Calc. Mass	Err [ppm]
292.1263	1	C17 H20 Cl F N	292.1263	0.00

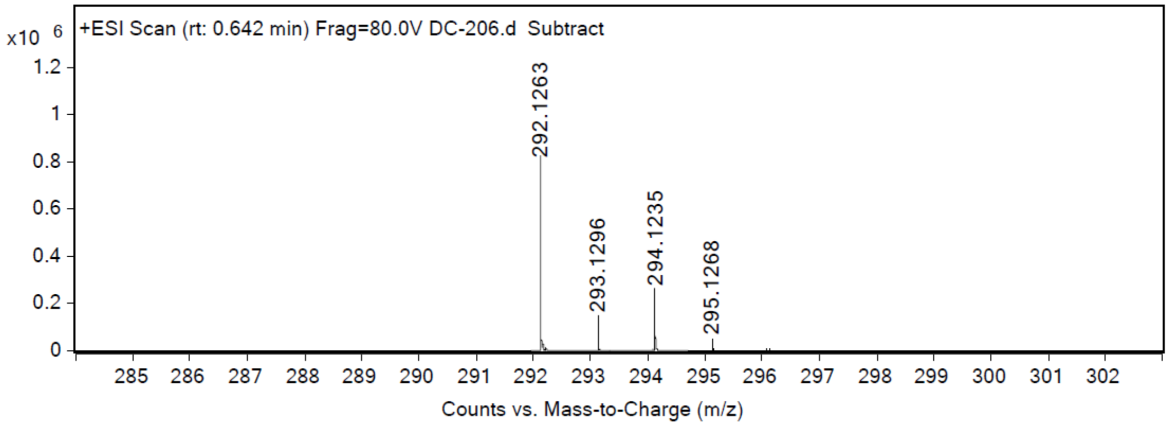

Figure S128. HR-ESI-MS spectrum of **2c-[N*s*]** cationic fragment (positive mode).

Meas. m/z	#	Formula	Calc. Mass	Err [ppm]
287.1032	1	C15 H21 Cl F S	287.1031	0.35

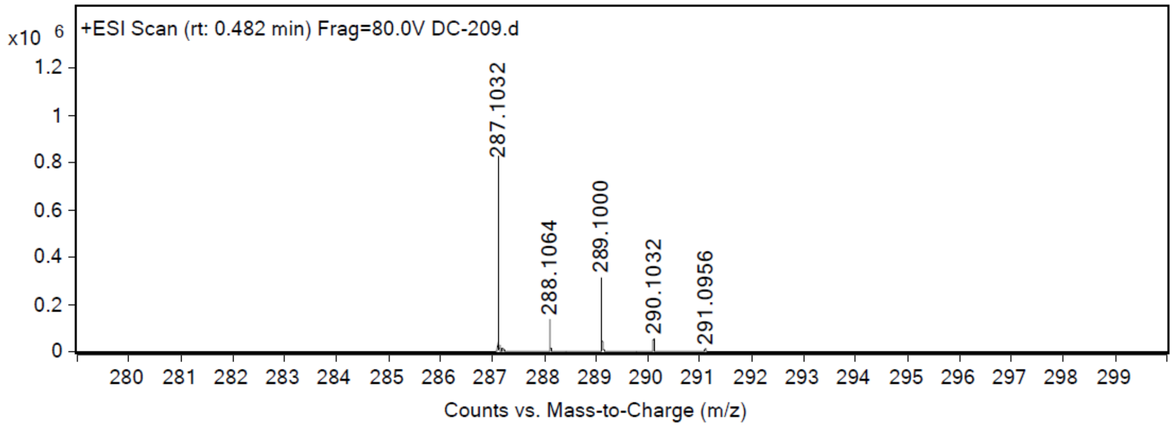

Figure S129. HR-ESI-MS spectrum of **2c-[B]** cationic fragment (positive mode).

Meas. m/z	#	Formula	Calc. Mass	Err [ppm]
297.1507	1	C16 H26 F S Si	297.1503	1.35

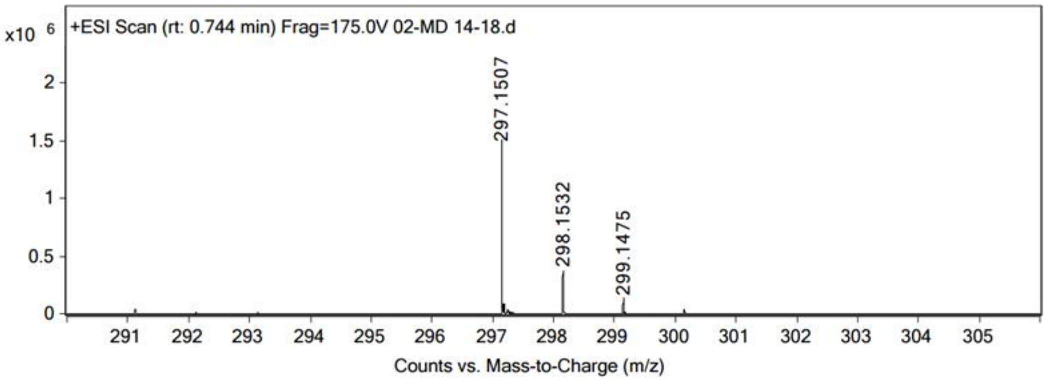

Figure S130. HR-ESI-MS spectrum of **2e-[A]** cationic fragment (positive mode).

Meas. m/z	#	Formula	Calc. Mass	Err [ppm]
330.2053	1	C20 H29 F N Si	330.2048	1.51

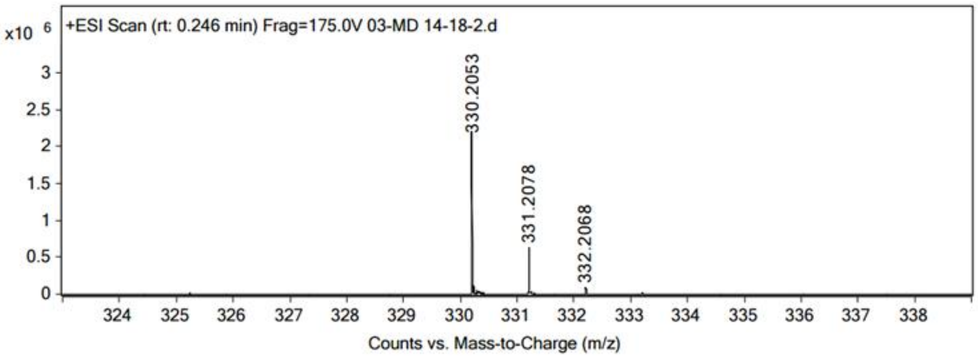

Figure S131. HR-ESI-MS spectrum of **2e-[N*s*]** cationic fragment (positive mode).

Meas. m/z	#	Formula	Calc. Mass	Err [ppm]
270.0961	1	C13 H17 F N O2 S	270.0959	0.74

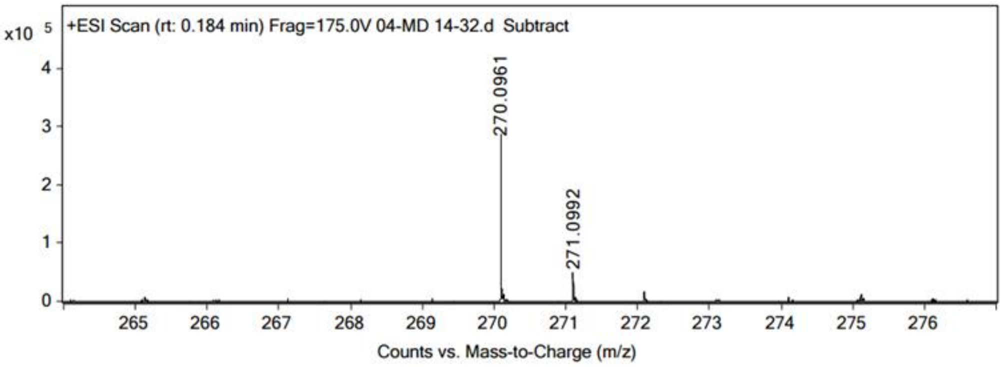

Figure S132. HR-ESI-MS spectrum of **2g-[A]** cationic fragment (positive mode).

Meas. m/z	#	Formula	Calc. Mass	Err [ppm]
273.0874	1	C14 H19 Cl F S	273.0875	0.37

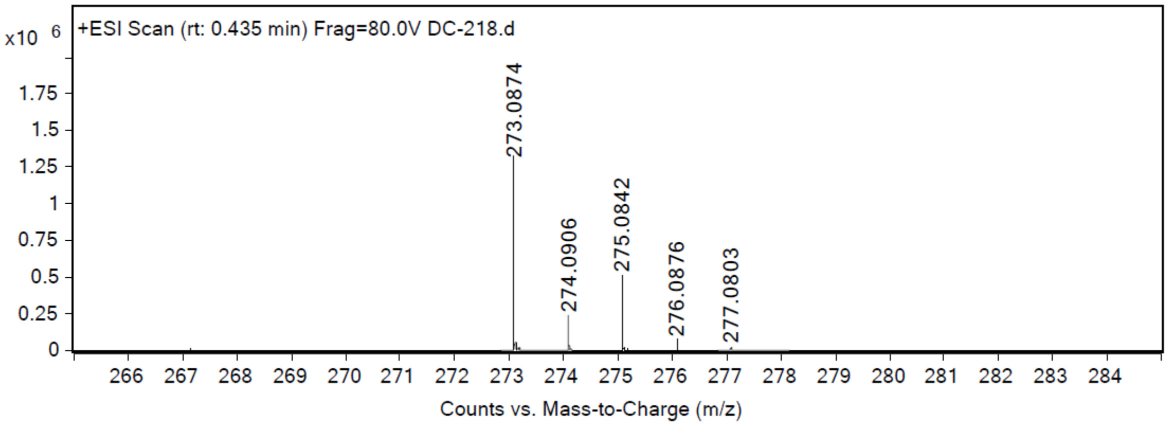

Figure S133. HR-ESI-MS spectrum of **2h-[A]** cationic fragment (positive mode).

Meas. m/z	#	Formula	Calc. Mass	Err [ppm]
301.1188	1	C16 H23 Cl F S	301.1188	0.00

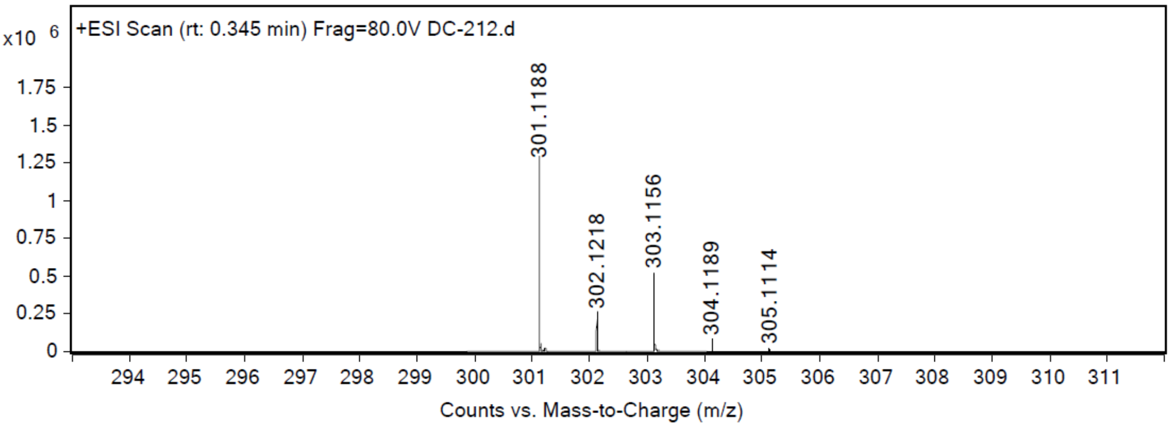

Figure S134. HR-ESI-MS spectrum of **2h-[B]** cationic fragment (positive mode).

Meas. m/z	#	Formula	Calc. Mass	Err [ppm]
306.1421	1	C18 H22 Cl F N	306.1419	0.65

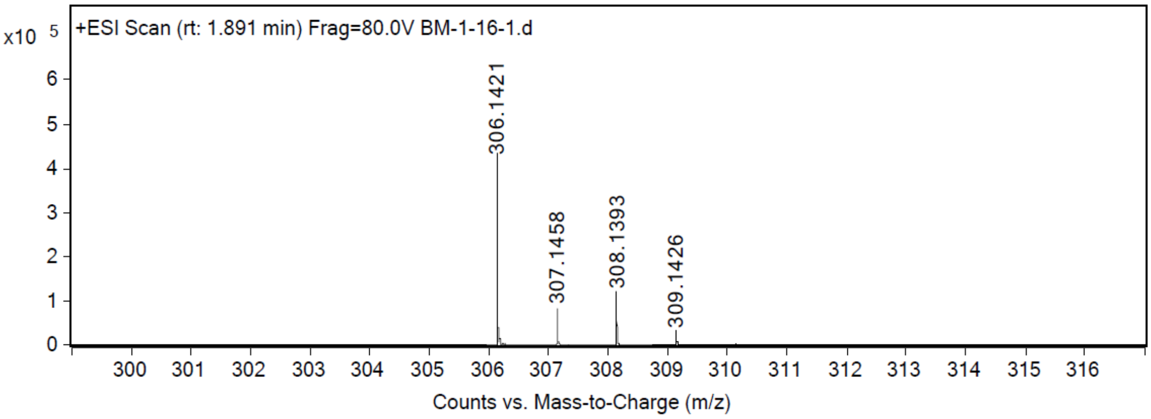

Figure S135. HR-ESI-MS spectrum of **2h-[N*s*]** cationic fragment (positive mode).

Meas. m/z	#	Formula	Calc. Mass	Err [ppm]
273.0876	1	C14 H19 Cl F S	273.0875	0.37

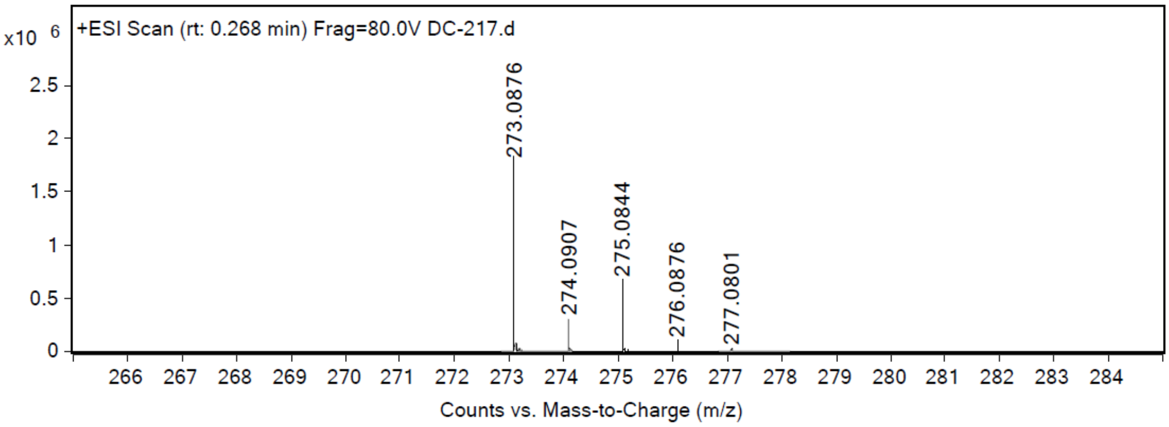

Figure S136. HR-ESI-MS spectrum of **2i-[A]** cationic fragment (positive mode).

Meas. m/z	#	Formula	Calc. Mass	Err [ppm]
306.142	1	C18 H22 Cl F N	306.1419	0.33

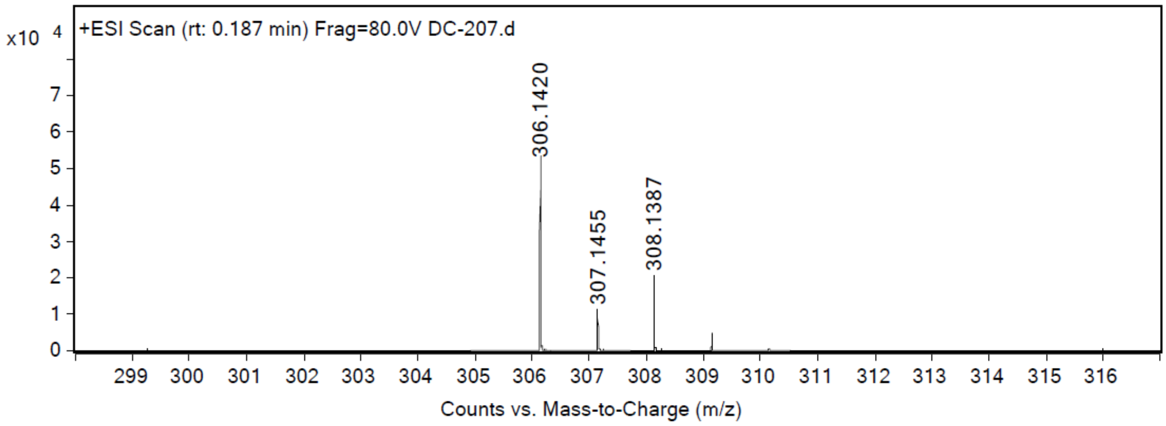

Figure S137. HR-ESI-MS spectrum of **2i-[N*s*]** cationic fragment (positive mode).

Meas. m/z	#	Formula	Calc. Mass	Err [ppm]
301.1187	1	C16 H23 Cl F S	301.1188	0.33

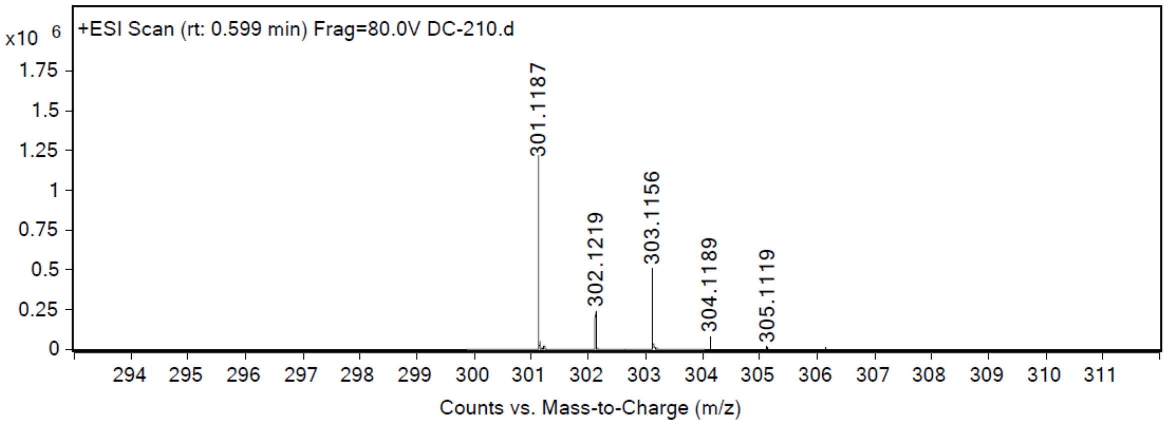

Figure S138. HR-ESI-MS spectrum of **2i-[B]** cationic fragment (positive mode).

Meas. m/z	#	Formula	Calc. Mass	Err [ppm]
274.1607	1	C17 H21 F N O	274.1602	1.82

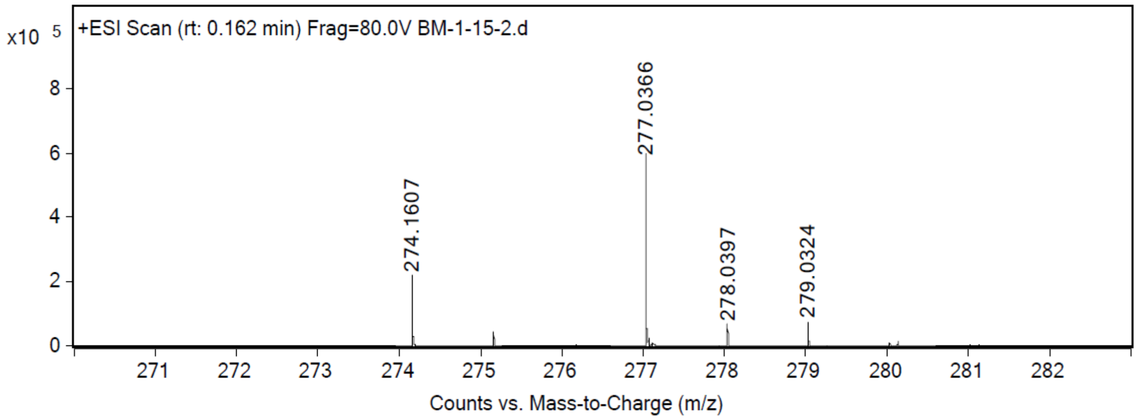

Figure S139. HR-ESI-MS spectrum of **2u-[N*s*]** cationic fragment (positive mode).

Meas. m/z	#	Formula	Calc. Mass	Err [ppm]
221.1188	1	C10 H22 F S Si	221.119	0.90

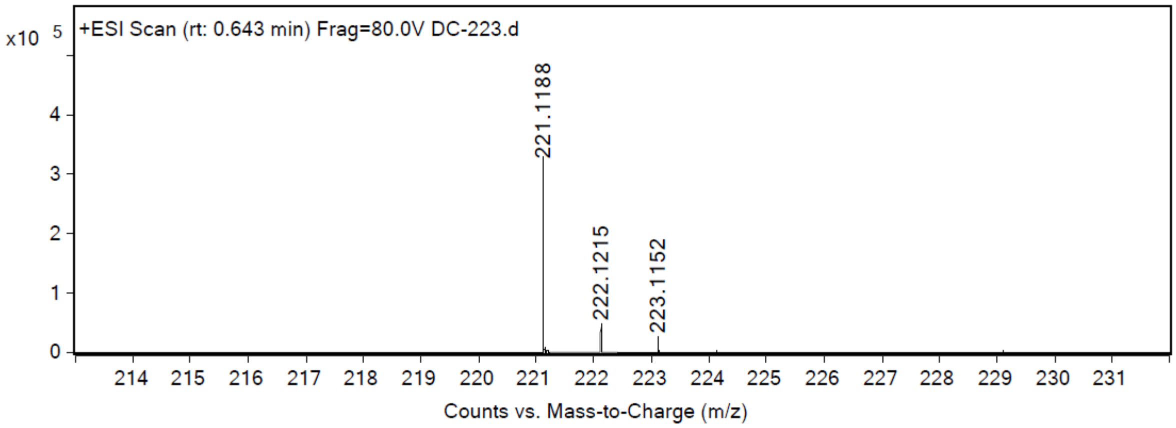

Figure S140. HR-ESI-MS spectrum of **2w-[A]** cationic fragment (positive mode).

Meas. m/z	#	Formula	Calc. Mass	Err [ppm]
267.1578	1	C16 H24 F S	267.1577	0.37

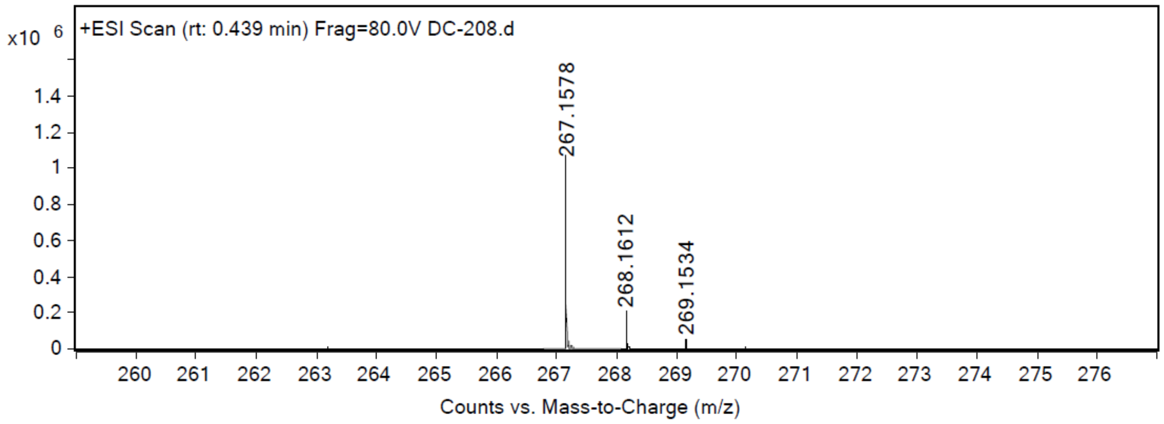

Figure S141. HR-ESI-MS spectrum of **2x-[A]** cationic fragment (positive mode).

DFT Studies

Computational methodology

To gain insight into the mechanism of the C-F bond activation, we performed DFT calculations with the *Gaussian 16* suite of programs (Revision A.03).[7] The calculations were performed using dispersion-corrected ωB97X-D exchange-correlation functional.[8] The global solvation effects were taken into account by computing the solvation free energies employing SMD continuum solvation model (DCM; ε = 8.93).[9] Geometry optimisations and transition state locations were performed with SMD solvation method.

The nature of the stationary points located on PES was identified by harmonic vibrational frequency calculations. The vibrational analysis revealed that no imaginary frequencies were found for the reported minima whereas all located transition states had only one imaginary frequency. The connection between the reactant and the product through a located transition state was further confirmed by optimizing slightly altered bond distance along the two directions which were associated with the imaginary frequency found in the vibrational frequency calculation.

Overall, the reported Gibbs free energies (*vide infra*) were obtained by combining SMD(DCM)-ωB97X-D/Def2TZVPP electronic energies with the thermal and entropic contributions computed at the SMD(DCM)-ωB97X-D/Def2SVP level (T = 298.15 K).[10] The thermal and entropic contributions were estimated within the ideal gas−rigid rotor−harmonic oscillator (RRHO) approximation. In this formula, the value of ΔG_{conc} (0.0030119 Hartree at 298.15 K ≈ 1.89 kcal/mol) corresponds to concentration correction to the Gibbs free energy when shifting from ideal gas standard state (p = 1 atm) to the standard concentration in solution phase (c = 1 mol/dm^3).

$$G = E_0'(\mathrm{DCM}) + (G_0(\mathrm{DCM}) - E_0(\mathrm{DCM})) + \Delta G_{conc}$$

Computational results

We performed DFT calculations to shed light on mechanistic aspects on the stereoselective C-F activation. Based on preliminary mechanistic control experiments, we argued that the C-F activation begins with the reaction of the geminal difluoro-substrate with the **BCF** to form a carbocation intermediate and **[BCFF]**$^-$ anion, corresponding to an S$_N$1 reaction mechanism. The computed relative free energy profile along with the structures of the transition state and relevant ground state species are shown in Figure S143. The reactive complex **2**···**BCF** lies at 7.3 kcal/mol with respect to the reactant state. The transition states **TS-1** related to the S$_N$1 pathway was found to be at 22.7 kcal/mol in free energy. Once the high-lying intermediate **[2a]**$^+$···**[BCFF]**$^-$ formed**,** this intermediate complex then serves as carbocation donor, which can lead to the facile formation of the sulfonium salt. A barrier is not reported for the reaction between **[2a]**$^+$···**[BCFF]**$^-$ and the chiral sulfide **A** as a first-order saddle point could not be located, and this step is assumed to proceed with a very small barrier. Attempts to locate the transition state leading to **[2a-A]**$^+$···**[BCFF]**$^-$ resulted in the direct formation of the product.

To explore the kinetic feasibility of the carbonation addition onto sulfide A, we carried out constrained geometry optimizations (energy scans) starting from the naked carbonation and reducing the distance between the carbon and the sulfur atoms (see Figure S142). The potential energy curve revealed no energy maximum, and hence, the transition state corresponding to the addition could not be located. Instead, the obtained potential energy curve showed a gradual decrease in energy, leading to the direct formation of the **2h-[A]**$^+$ product.

The constraint geometry optimisations revealed that a small conformational change in complex **A**···**[2a]**$^+$···**[BCFF]**$^-$ can lead to the facile formation of **[2a-A]**$^+$···**[BCFF]**$^-$. These calculations also suggest that the barrier of the carbocation transfer from **A**···**[2a]**$^+$···**[BCFF]**$^-$ lies close to **TS-1** and the separate state **[2a]**$^+$ + **[BCFF]**$^-$ in free energy. This shows that the cationic intermediate has an enhanced reactivity towards the neutral sulfide.

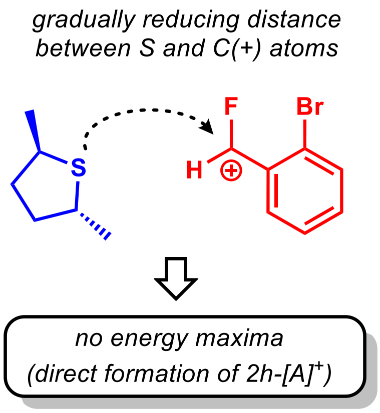

Figure S142. Energy scans at decreasing C···S distances supported a barrierless addition of sulfide to carbocation [**2a**]$^+$.

Initially, we expected the addition of carbocation **[2a]**$^+$ to chiral sulfide **A** to be the stereodetermining step, however, this assumption was proved incorrect (see below). The stability of the intermediate **[2a-A]**$^+$···**[BCFF]**$^-$ was computed to be -1.1

kcal/mol with respect to the **2a** + **BCF** reactant state, suggesting a slightly exergonic process, and its dissociated form **[2a-A]**$^+$ + **[BCFF]**$^-$ was calculated to be slightly more favored (by 0.6 kcal/mol).

Based on control experiments, we found that the *dr* of the product improves with prolonged reaction time and additional sulfide does not facilitate the isomerization. This suggests that the observed *dr* may arise from a thermodynamic equilibrium rather than from kinetic control, and that the sulfide is in not involved in the rate-determining step of the isomerisation. The latter implies that the barrier associated with the carbocation **[2a]**$^+$ migration starting from the complex **A**···**[2a]**$^+$···**[BCFF]**$^-$ should be higher than the separate state **A** + **[2a]**$^+$ + **[BCFF]**$^-$ where the free carbocation **[2a]**$^+$ can react with the chiral sulfide **A**. Our computations also suggest that the isomerization is likely to operate via an S_N1 pathway. We found that the reaction of **[2a]**$^+$ with **A** is practically barrierless, as no energy maximum on the potential energy curve was found when gradually decreasing the distance between **[2a]**$^+$ and **A** during constraint geometry optimisations. Thus, the barrier for the isomerisation is estimated by the free energy required to form the separate **[2a]**$^+$ cation, which is 24.8 kcal/mol from the most stable diastereomer and 22.5 kcal/mol from the minor diastereomer (see Figure S141). The calculated trend compares well with the observed reaction rates, albeit the isomerisation was found to be somewhat slower than the process of C-F activation in our experiments.

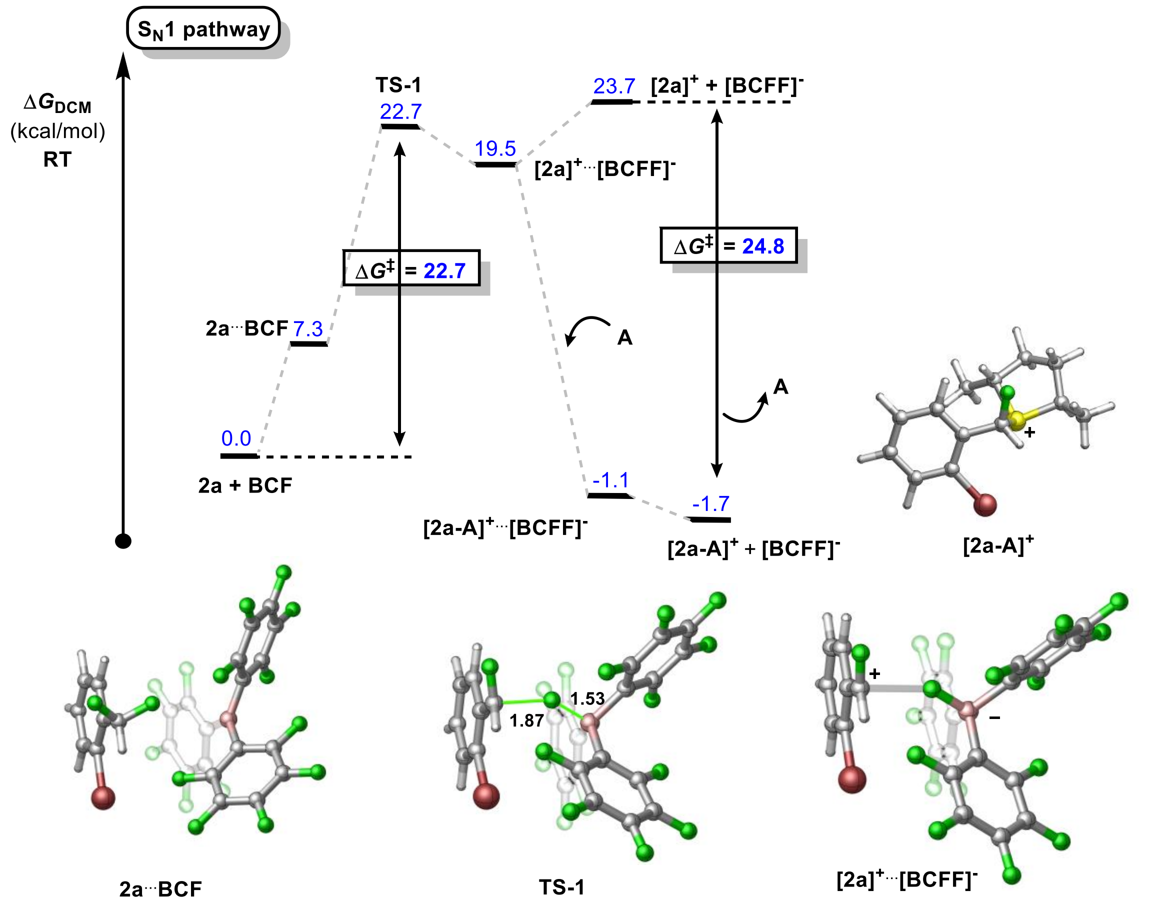

Figure S143. Computed Gibbs free energy profile (ΔG in kcal/mol) for C-F activation in substrate **2a** via S_N1 mechanism. The relative stability (in blue) is given with respect to **2a** + **BCF** + **A** reference state.

In the experiments, the formation of **TMSF** from the reaction between **[BCFF]**$^-$ and **TMSNTf$_2$** drives the reaction to the product side, as its formation is thermodynamically highly favourable, leading to **[2a-A]**$^+$ + **[NTf$_2$]**$^-$. Generally, the ion pairs **[2a-A]**$^+$···**[NTf$_2$]**$^-$ are predicted to be higher in free energy than the separate state **[2a-A]**$^+$ + **[NTf$_2$]**$^-$. Some representative ion pairs structures are shown in Figure S144. This means that the relative rate of the C-F activation and the isomerisation is not affected by the counter anion.

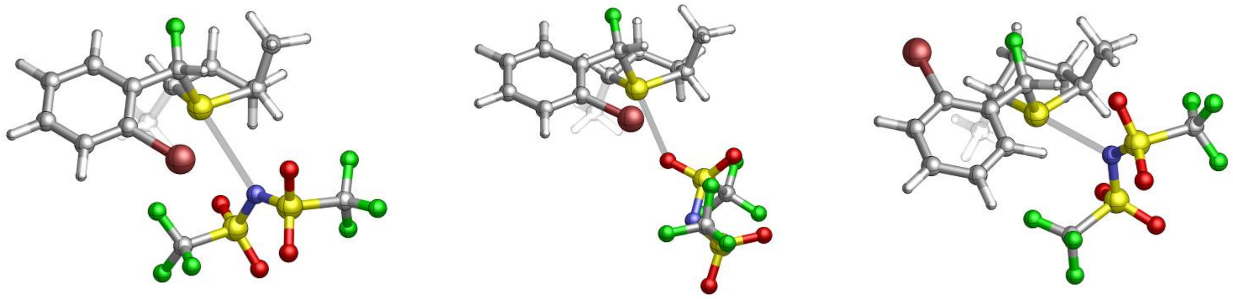

[2a-A]$^+$··· [NTf2]$^-$ (2.0) **[2a-A]$^+$··· [NTf2]$^-$-II** (3.7) **[2a-A]$^+$··· [NTf2]$^-$-III** (6.3)

Figure S144. DFT-optimized structures of intermediate **[2a-A]**$^+$···**[NTf$_2$]**$^-$. The relative stability (in blue) is given in kcal/mol with respect to the separate state **[2a-A]**$^+$ + **[NTf$_2$]**$^-$.

We performed conformational analysis for the diastereomer products. The computationally obtained structures for diastereomers are depicted in Figure S145. The isomer **[2a^R-A]**$^+$ having (*R*) configuration on the benzylic carbon atom was found to be 2.3 kcal/mol more stable than the other diastereomer.

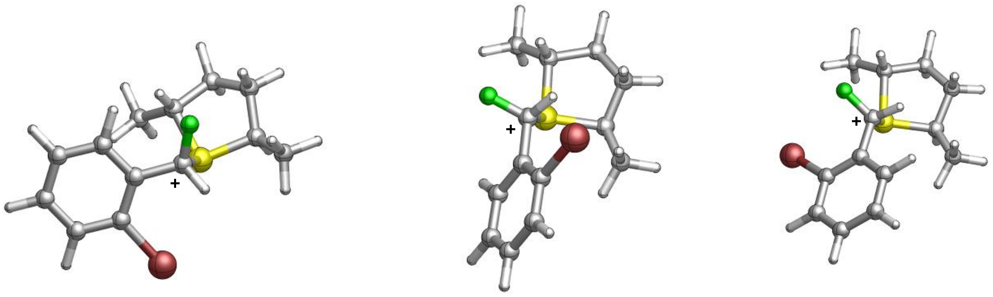

[2a^R-A]$^+$ (= **[2a-A]$^+$**) (0.0) **[2a^R-A]$^+$-II** (3.2) **[2a^R-A]$^+$-III** (3.9)

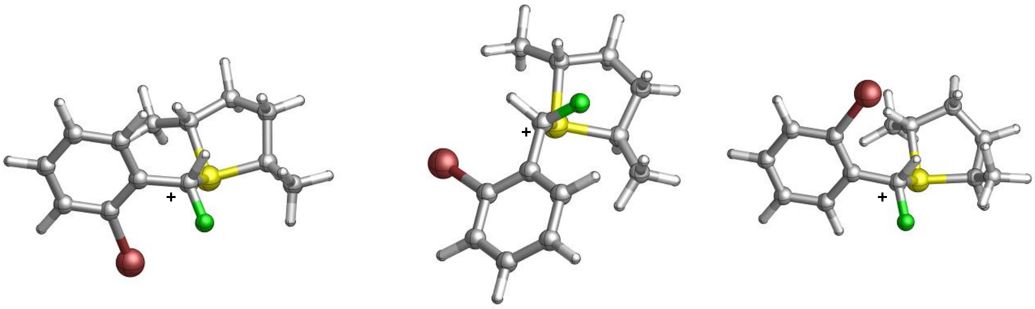

[2a^S-A]$^+$ (2.3) **[2a^S-A]$^+$-II** (3.8) **[2a^S-A]$^+$-III** (5.1)

Figure S145. DFT-optimized structures of **[2a-A]**$^+$ isomers. The relative stability (in blue) is given in kcal/mol with respect to isomer **[2a^R-A]$^+$.**

The barrier of C-F bond cleavage with the assistance of chiral sulfide **A** was also assessed by calculating the free energy difference between the respective transition state **TS-2** and the most stable reactant state **A** + **1a** + **BCF** (Figure S146). The reactive ternary complex of **A**···**1a**···**BCF** was found to be 14.0 kcal/mol above the reactant state. The C-F activation step with the involvement of **A** has a 6.5 kcal/mol higher barrier than that on the S_N1 reaction pathway. Along the S_N2 reaction path, the intermediate **A**···**[1a]**$^+$···**[BCFF]**$^-$ was not be found, rather the direct formation of **[2a-A]**$^+$···**[BCFF]**$^-$ was observed.

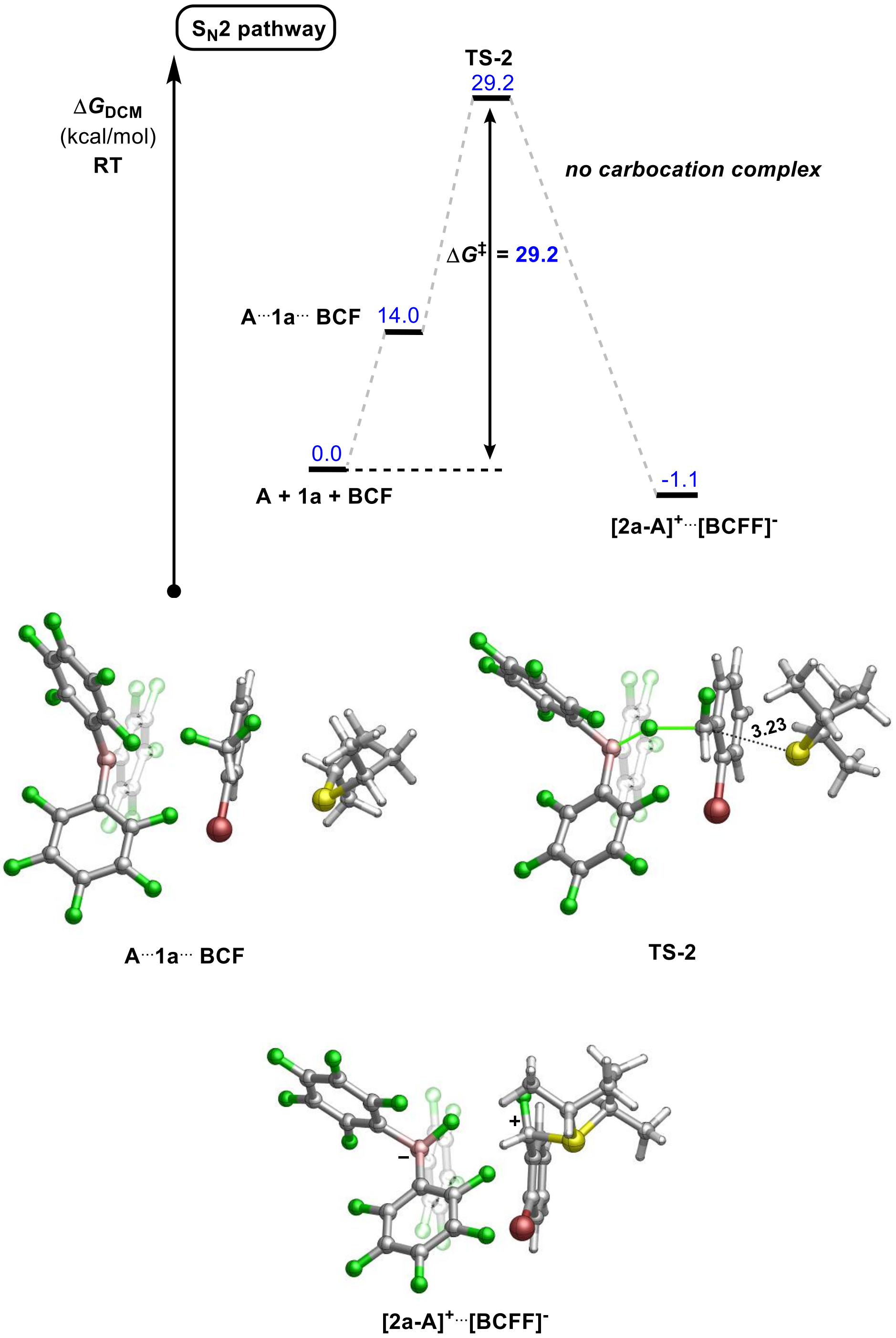

Figure S146. Computed Gibbs free energy profile (Δ*G* in kcal/mol) for C-F activation in substrate **1a** via S_N2 mechanism. The relative stability (in blue) is given with respect to **1a** + **BCF** + **A** reference state.

Consistent with our experiment results, the transition state identified for isomerisation via S_N2 mechanism possesses a higher barrier when compared to the free energy required for the generation of the free **[2a]**$^+$ intermediate from the product **[2a-A]**$^+$ (24.8 kcal/mol from the major **[2a^R-A]**$^+$ and 22.5 kcal/mol from the minor **[2a^S-A]**$^+$). The transition state **TS-3** encompassing two chiral sulfides **A** are shown in Figure S147.

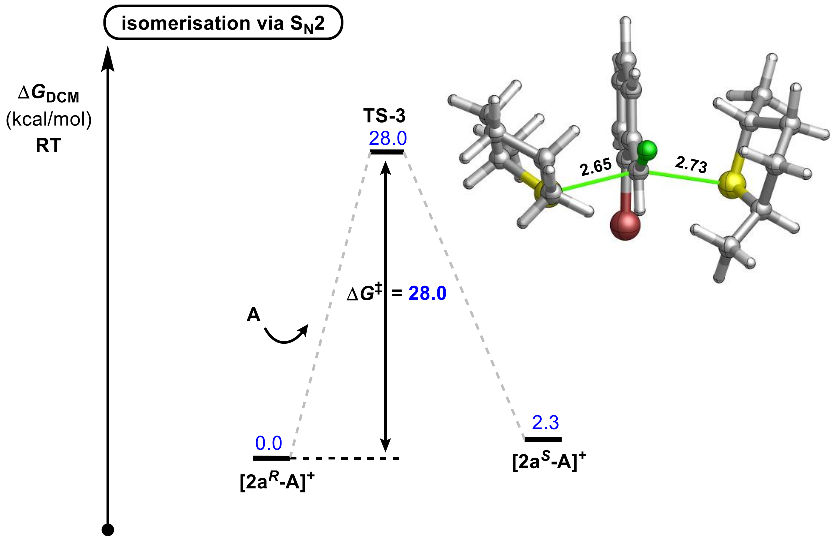

Figure S147. Computed Gibbs free energy profile (ΔG in kcal/mol) for the isomerisation of product **[2a-A]**$^+$ via S_N2 mechanism. The relative stability (in blue) is given with respect to **[2a^R-A]**$^+$ + **A** reference state.

The role of the **[NTf$_2$]**$^-$ in the isomerisation was also considered. The transition state involving **[NTf$_2$]**$^-$ anion as the nucleophile was located with activation barriers 27.2 kcal/mol (**TS-4**), affording the intermediate **2a-NTf$_2$** (Figure S148). This provided the observation that isomerisation is unlikely to proceed through transient intermediate **2a-NTf$_2$**, as the barrier height is lower in the case of the generation of free **[2a]**$^+$.

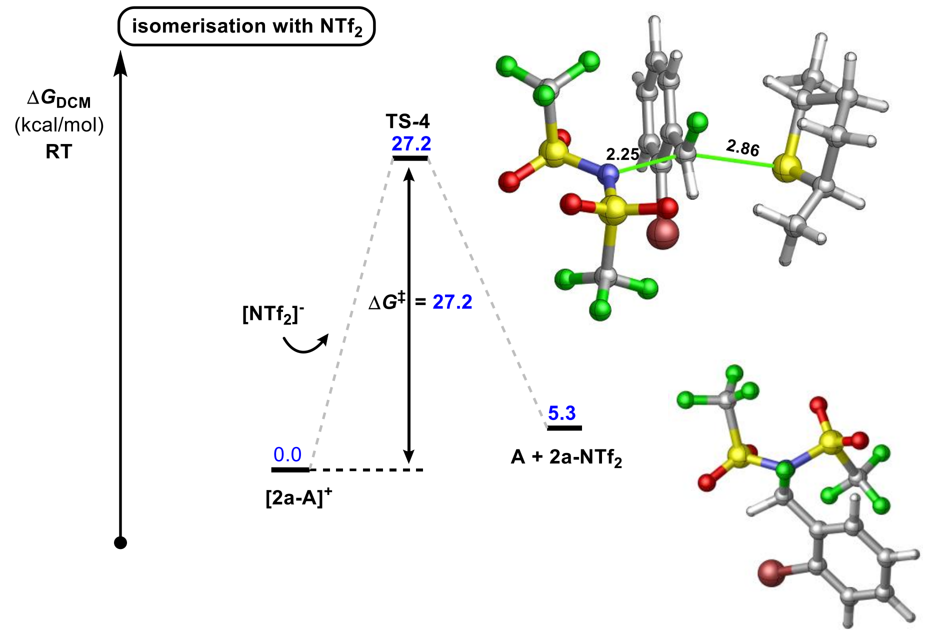

Figure S148. Computed Gibbs free energy profile (ΔG in kcal/mol) for the isomerisation of product **[2a-A]**$^+$ with the involvement of **[NTf2]**$^-$.via S_N2 mechanism. The relative stability (in blue) is given with respect to **[2a- A]**$^+$ + **[NTf2]**$^-$. reference state.

Next, the conformational space of the **[2a-N*S*]**$^+$ resulted from the substitution of **[2a^R-A]**$^+$ with the enantiopure chiral anime (*S*)-N,N-dimethyl-1-phenylethylamine (**N*S***) was surveyed. Representative conformers are presented in Figure S149.

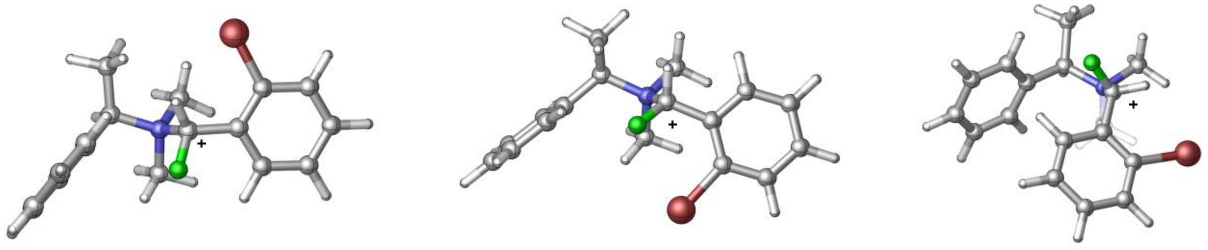

[2a-N$_S$]$^+$ (0.0) **[2a-N$_S$]$^+$-II** (2.4) **[2a-N$_S$]$^+$-III** (5.8)

Figure S149. DFT-optimized structures of intermediate **[2a-N$_S$]**$^+$. The relative stability (in blue) is given in kcal/mol with respect to isomer **[2a-N*S*]**$^+$.

In agreement with experiments, the substitution **[2a-N*S*]**$^+$ with **N*S*** is kinetically rather unfavorable (Figure S150). This step proceeds via **TS-5** and we obtained a barrier of 33.9 kcal/mol, which strongly suggests that the **[2a-N*S*]**$^+$ does not go through isomerisation under the applied reaction conditions, and hence, no erosion of stereochemical integrity is expected once formed.

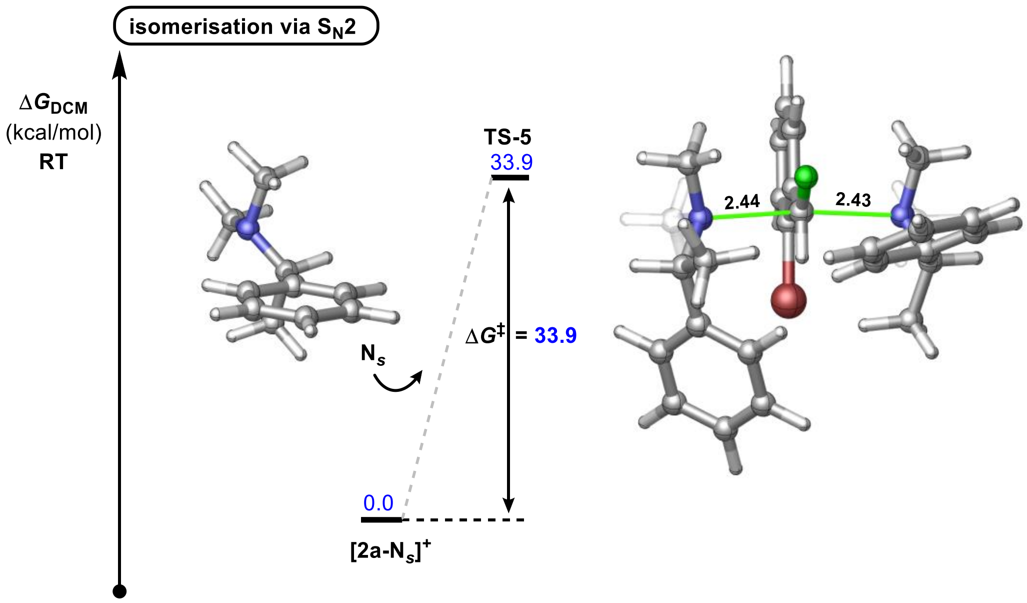

Figure S150. Computed Gibbs free energy profile (ΔG in kcal/mol) for the isomerisation of product **[2a-N*s*]**$^+$ via S_N2 mechanism. The relative stability (in blue) is given with respect to **[2a- N*s*]**$^+$ + **N*s*** reference state.

The barrier height for the isomerisation taking place through carbocation intermediate was is estimated to be at 34.4 kcal/mol with respect to **[2a-N*s*]**$^+$ reactant state, indicating that isomerisation of **[2a-N*s*]**$^+$ via S_N1 mechanism is also very unlikely to take place (Figure S151).

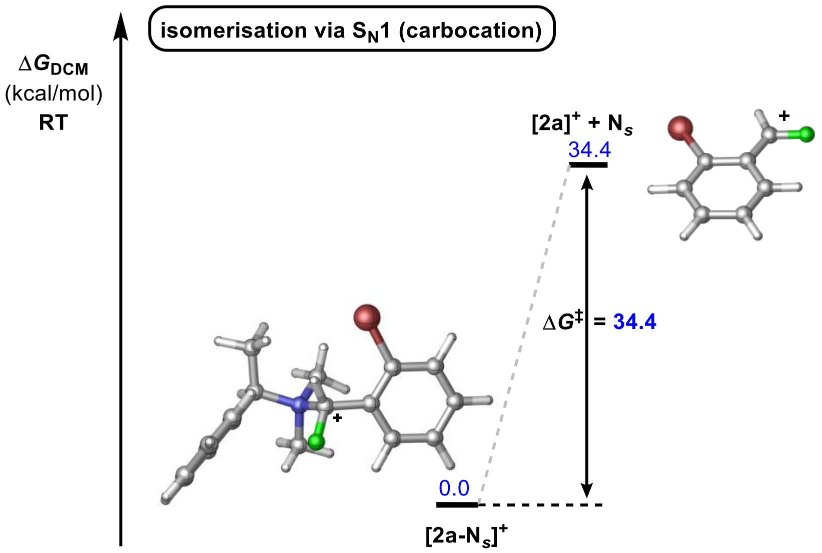

Figure S151. Computed Gibbs free energy profile (ΔG in kcal/mol) for the isomerisation of product **[2a- N*s*]**$^+$ via S_N1 mechanism. The relative stability (in blue) is given with respect to **[2a-N*s*]**$^+$ reference state.

Computed energy components of the reported structures.

Table S2. Summary of energy data (given in Hartree) computed for optimized structures at the SMD(DCM)-ωB97X-D/Def2SVP level of theory. Note that G contains concentration correction. For the definition of various energy components, see Computational details section (*vide supra*).

**Structure**	E_0'(DCM)	G_0(DCM)	E_0(DCM)	G(DCM)
**BCF**	-2208.5655	-2205.8854	-2205.9808	-2208.4671
**A**	-634.1087	-633.5783	-633.7166	-633.9674
**A···BCF**	-2842.6900	-2839.4641	-2839.7254	-2842.4257
**2a···BCF**	-5252.2804	-5248.6715	-5248.8593	-5252.0896
**TS-1**	-5252.2576	-5248.6505	-5248.8400	-5252.0650
**[2a]**$^{+}$**···[BCFF]**$^{-}$	-5252.2603	-5248.6537	-5248.8409	-5252.0701
**[2a-A]**$^{+}$**···[BCFF]**$^{-}$	-5886.4232	-5882.2644	-5882.6142	-5886.0703
**[BCFF]**$^{-}$	-2308.6308	-2305.8288	-2305.9244	-2308.5321
**[2a**^{R}**-A]**$^{+}$ **(= [2a-A]**$^{+}$**)**	-3577.7720	-3576.4301	-3576.6597	-3577.5394
**[2h**^{R}**-A]**$^{+}$**-II**	-3577.7678	-3576.4227	-3576.6533	-3577.5342
**[2a**^{R}**-A]**$^{+}$**-III**	-3577.7671	-3576.4224	-3576.6534	-3577.5331
**[2a**^{S}**-A]**$^{+}$	-3577.7696	-3576.4243	-3576.6552	-3577.5357
**[2a**^{S}**-A]**$^{+}$**-II**	-3577.7686	-3576.4239	-3576.6561	-3577.5333
**[2a**^{S}**-A]**$^{+}$**-III**	-3577.7661	-3576.4194	-3576.6513	-3577.5312
**[2a]**$^{+}$	-2943.6015	-2942.8108	-2942.8780	-2943.5313
**A···2a···BCF**	-5886.3933	-5882.2407	-5882.5847	-5886.0463
**TS-2**	-5886.3750	-5882.2203	-5882.5703	-5886.0221
**TS-3**	-4211.8522	-4209.9676	-4210.3547	-4211.4620
**[NTf2]**$^{-}$	-1827.5865	-1825.9268	-1825.9398	-1827.5706
**[2a-A]**$^{+}$**···[NTf2]**$^{-}$	-5405.3722	-5402.3621	-5402.6245	-5405.1068
**[2a-A]**$^{+}$**···[NTf2]**$^{-}$**-II**	-5405.3685	-5402.3568	-5402.6182	-5405.1041
**[2a-A]**$^{+}$**···[NTf2]**$^{-}$**-III**	-5405.3687	-5402.3603	-5402.6261	-5405.0999
N_s	-444.8703	-444.2001	-444.3950	-444.6724
**[2a-**N_s**]**$^{+}$	-3388.5558	-3387.0704	-3387.3648	-3388.2584
**[2a-**N_s**]**$^{+}$**-II**	-3388.5518	-3387.0661	-3387.3604	-3388.2546
**[2a-**N_s**]**$^{+}$**-III**	-3388.5475	-3387.0618	-3387.3570	-3388.2493
**TS-4**	-3833.3923	-3831.2174	-3831.7300	-3832.8767
**TS-5**	-5405.3330	-5402.3285	-5402.5918	-5405.0667

Cartesian coordinates of the reported structures

Cartesian coordinates of the optimized geometries are given below in standard XYZ format (units are in Å). The first line is the molecule name (as defined above in Table S2), the second line indicates total number of atoms.

BCF

34

B	3.933304	-0.774885	-0.459732
C	3.019494	0.234103	-1.245578
C	2.356071	1.283230	-0.602829
C	2.820960	0.128210	-2.625142
C	1.532961	2.175067	-1.280129
C	2.018075	1.011890	-3.336510
C	1.368735	2.037600	-2.655436
C	4.890010	-0.251141	0.671496
C	5.586952	0.955071	0.548588
C	5.094505	-0.964488	1.856932
C	6.447395	1.427008	1.532691
C	5.935247	-0.514689	2.867550
C	6.616293	0.687533	2.699557
C	3.884358	-2.306960	-0.805260
C	2.684919	-2.956544	-1.113955
C	5.036620	-3.099187	-0.819259
C	2.621470	-4.312550	-1.411715
C	5.011713	-4.453833	-1.127680
C	3.794267	-5.061918	-1.420541
F	1.537552	-2.286643	-1.107183
F	1.463852	-4.893812	-1.684201
F	3.752131	-6.348155	-1.706762
F	6.127064	-5.166483	-1.145824
F	6.222435	-2.562671	-0.552233
F	4.459736	-2.112837	2.066620
F	6.092600	-1.212947	3.980957
F	7.423991	1.124896	3.645389
F	7.102282	2.566204	1.372595
F	5.464316	1.692269	-0.549787
F	2.476925	1.447528	0.710333
F	0.907961	3.146938	-0.634293
F	0.597055	2.880214	-3.313820
F	1.865122	0.887646	-4.645458
F	3.426276	-0.831488	-3.316948

A

19

S	-0.678959	-0.712202	-0.026081
C	0.073115	-0.215316	1.588670
C	-0.812941	-0.863144	2.654708
C	-2.248996	-0.790570	2.154938
C	-2.258546	-1.320091	0.719253
H	-0.018454	0.880241	1.662729
H	-0.514377	-1.917593	2.785671
H	-0.680012	-0.358781	3.624926
H	-2.942393	-1.370721	2.784219
H	-2.595062	0.257455	2.159590
H	-2.201297	-2.420237	0.741908
C	-3.471013	-0.901343	-0.096346
H	-3.431314	-1.303429	-1.120273
H	-4.391291	-1.282685	0.375411
H	-3.543037	0.195745	-0.160891
C	1.541386	-0.599031	1.675094
H	2.136641	-0.110464	0.888559
H	1.954724	-0.286306	2.647857
H	1.670635	-1.688355	1.577046

A···BCF

53

B	-0.344615	-1.313511	-0.224105
C	1.190952	-1.415856	-0.756795
C	2.048296	-0.313247	-0.768907
C	1.798773	-2.618248	-1.127892
C	3.387997	-0.369583	-1.130029
C	3.135352	-2.718922	-1.512366
C	3.938073	-1.587265	-1.513933
C	-1.266990	-2.617994	-0.629220
C	-1.521963	-2.775577	-1.995621
C	-1.877913	-3.572073	0.181352
C	-2.320798	-3.777691	-2.530883
C	-2.691757	-4.591941	-0.312771
C	-2.918691	-4.697206	-1.676775
C	-1.216311	-0.007875	-0.689875
C	-0.935164	0.793327	-1.798665
C	-2.420172	0.290889	-0.050978
C	-1.745733	1.854037	-2.197929
C	-3.255164	1.341342	-0.411414
C	-2.913396	2.133664	-1.499955
F	-2.809946	-0.433549	1.003951
F	-4.361076	1.592500	0.276978
F	-3.689936	3.139990	-1.868059
F	-1.417867	2.587275	-3.254074
F	0.135486	0.577651	-2.560052
F	1.606736	0.886499	-0.384154
F	4.140883	0.721508	-1.101523
F	5.210856	-1.666956	-1.863902
F	3.646972	-3.893499	-1.855029
F	1.131640	-3.772699	-1.096397
F	-0.956300	-1.943272	-2.874102
F	-2.512392	-3.865013	-3.840761
F	-3.686225	-5.661891	-2.159668
F	-3.244728	-5.465738	0.519522
F	-1.706594	-3.581626	1.505220
C	1.406769	-2.064693	2.287865
C	0.235938	0.375881	2.628558
C	2.064257	-1.103225	3.278745
C	1.745569	0.315729	2.830880
H	1.666468	-1.277489	4.292239
H	3.145637	-1.302009	3.307919
H	2.040173	1.061662	3.584291
H	2.267305	0.563705	1.894192
S	-0.208938	-1.263655	1.843862
H	1.999594	-2.072176	1.365434
H	-0.245498	0.244605	3.610008
C	1.208854	-3.484969	2.781528
H	0.795043	-4.133840	1.997680
H	2.187861	-3.894415	3.075125
H	0.543211	-3.520827	3.656807
C	-0.326840	1.631129	1.995294
H	-1.425051	1.628363	2.000502
H	0.000462	2.483710	2.610120
H	0.032148	1.790141	0.972762

2a···BCF

49

F	-1.916269	-9.062250	1.145136
C	-2.960958	-8.806701	5.932639
C	-1.717621	-9.302686	3.470249
C	-3.030113	-9.730881	3.711561
C	-1.130959	-9.593657	2.115256
C	-3.652468	-9.483055	4.927383
C	-1.039940	-8.623961	4.487703
C	-1.655260	-8.378929	5.717422
H	-1.111326	-7.855492	6.505212
F	-1.123830	-10.942810	1.891832
B	0.452330	-13.233811	2.905477

C	0.161639	-12.528201	4.276862
C	1.173565	-11.964305	5.067177
C	-1.135480	-12.443112	4.808565
C	0.926439	-11.344472	6.286875
C	-1.410340	-11.868693	6.042046
C	-0.375735	-11.293125	6.772014
C	1.736771	-12.935146	2.042102
C	2.247437	-11.648670	1.850461
C	2.409019	-13.962068	1.369410
C	3.334916	-11.379020	1.029951
C	3.512617	-13.732782	0.555031
C	3.972047	-12.431657	0.380073
C	-0.538971	-14.332638	2.355493
C	-0.966874	-14.332653	1.027482
C	-1.003667	-15.376046	3.156834
C	-1.828611	-15.299029	0.519687
C	-1.852146	-16.367055	2.680263
C	-2.267079	-16.323640	1.351976
F	-2.179128	-12.914963	4.140033
F	-2.643047	-11.852330	6.522440
F	-0.629308	-10.708933	7.925197
F	1.915235	-10.814631	6.989119
F	2.442977	-12.022854	4.687038
F	1.687590	-10.608596	2.457087
F	3.770338	-10.139528	0.867869
F	5.012721	-12.197004	-0.394861
F	4.125372	-14.737223	-0.051887
F	2.022379	-15.225912	1.506392
F	-0.565674	-13.378241	0.193631
F	-0.625116	-15.455932	4.430306
F	-2.264512	-17.347231	3.469807
F	-3.077537	-17.254346	0.883440
F	-2.226016	-15.256900	-0.742960
H	-4.673959	-9.827471	5.097265
H	-3.564107	-10.266637	2.923821
H	-3.435086	-8.616588	6.897536
H	-0.108165	-9.222939	1.977600
Br	0.736889	-7.986670	4.266002

TS-1

49

F	-2.475913	-9.768883	1.303773
C	-2.248169	-8.815770	6.141910
C	-1.718418	-9.360262	3.466401
C	-3.023741	-9.509506	3.977955
C	-1.470339	-9.650383	2.088537
C	-3.286731	-9.235956	5.309407
C	-0.676851	-8.932402	4.324104
C	-0.947360	-8.661439	5.657500
H	-0.150813	-8.334790	6.327299
F	-1.040131	-11.485773	2.005701
B	-0.113417	-12.548905	2.597098
C	-0.166163	-12.293380	4.209535
C	0.912052	-11.990966	5.037915
C	-1.407480	-12.324034	4.847752
C	0.777127	-11.739642	6.403933
C	-1.586911	-12.080439	6.202239
C	-0.480276	-11.780704	6.988425
C	1.360477	-12.424775	1.902431
C	1.765140	-11.485239	0.961611
C	2.307399	-13.405027	2.199586
C	3.027249	-11.492387	0.367751
C	3.574906	-13.453590	1.634698
C	3.937368	-12.484666	0.703838
C	-0.790706	-13.953450	2.095131
C	-1.111739	-14.105355	0.745463
C	-1.034578	-15.070109	2.891957

C	-1.668606	-15.264769	0.214433
C	-1.587143	-16.250727	2.400052
C	-1.907141	-16.348366	1.051795
F	-2.510768	-12.589575	4.143031
F	-2.798557	-12.097391	6.744512
F	-0.632840	-11.510177	8.275523
F	1.839459	-11.437272	7.140220
F	2.154401	-11.905246	4.563611
F	0.949676	-10.500902	0.558779
F	3.358011	-10.563136	-0.520819
F	5.139441	-12.508116	0.147823
F	4.435379	-14.406681	1.969033
F	2.010447	-14.351094	3.095335
F	-0.879784	-13.112778	-0.116664
F	-0.737769	-15.066795	4.192589
F	-1.804151	-17.282639	3.206990
F	-2.432105	-17.464047	0.567940
F	-1.961269	-15.348688	-1.077917
H	-4.295359	-9.358416	5.705064
H	-3.820796	-9.849674	3.315600
H	-2.444513	-8.610717	7.196352
H	-0.531815	-9.404539	1.588145
Br	1.088799	-8.704897	3.700673

$[2a]^{+}\cdots[BCFF]^{-}$

49

F	-2.530085	-9.597510	1.346947
C	-2.172674	-8.819502	6.203525
C	-1.733644	-9.201702	3.486943
C	-3.039514	-9.334735	4.028491
C	-1.532503	-9.411909	2.116303
C	-3.254520	-9.141405	5.377468
C	-0.643914	-8.879956	4.348354
C	-0.874745	-8.688076	5.699635
H	-0.052681	-8.444749	6.373851
F	-1.030605	-11.553940	1.950365
B	-0.149189	-12.570406	2.538864
C	-0.208359	-12.309521	4.162319
C	0.860463	-12.027413	5.008882
C	-1.459512	-12.312526	4.781217
C	0.710731	-11.775367	6.373637
C	-1.654735	-12.069851	6.134281
C	-0.555525	-11.794484	6.939389
C	1.347603	-12.444891	1.874213
C	1.754164	-11.472703	0.967145
C	2.303251	-13.420029	2.156423
C	3.022128	-11.450809	0.385463
C	3.577218	-13.440210	1.603471
C	3.938871	-12.443309	0.702560
C	-0.777719	-14.021883	2.077243
C	-1.088212	-14.214877	0.730924
C	-1.007318	-15.122536	2.898915
C	-1.621050	-15.397131	0.225237
C	-1.537564	-16.324939	2.435165
C	-1.847310	-16.463071	1.088158
F	-2.559164	-12.559900	4.062732
F	-2.875940	-12.060559	6.657992
F	-0.724108	-11.519821	8.224964
F	1.767344	-11.490289	7.126453
F	2.113485	-11.965392	4.555482
F	0.937297	-10.478843	0.587849
F	3.354245	-10.492278	-0.472558
F	5.147686	-12.439209	0.158293
F	4.446331	-14.392191	1.922182
F	2.013490	-14.391237	3.028337
F	-0.868325	-13.241013	-0.156936
F	-0.718806	-15.082829	4.202408

F	-1.743200	-17.340074	3.267569
F	-2.351182	-17.600339	0.629748
F	-1.903442	-15.519605	-1.067288
H	-4.254718	-9.249193	5.797204
H	-3.865171	-9.598529	3.366275
H	-2.333931	-8.677804	7.274425
H	-0.576102	-9.293609	1.600966
Br	1.110137	-8.695320	3.699568

$[2a\text{-}A]^{+}\cdots[BCFF]^{-}$

68

F	0.514268	-11.259082	2.080172
C	2.570459	-10.670334	6.531541
C	2.092260	-10.797559	3.772780
C	1.032595	-10.853315	4.685295
C	1.793482	-10.880284	2.297870
C	1.267360	-10.791438	6.054780
C	3.398121	-10.683371	4.269939
C	3.639245	-10.617897	5.640596
H	4.662228	-10.540862	6.011501
F	2.295645	-13.293005	0.618278
B	3.230368	-14.277787	1.113844
C	3.094264	-14.221381	2.757179
C	4.127012	-14.066279	3.677630
C	1.814201	-14.257566	3.313195
C	3.915019	-13.925058	5.048757
C	1.558459	-14.117651	4.672111
C	2.622023	-13.948475	5.549601
C	4.733425	-13.887794	0.559768
C	5.063362	-12.648202	0.019575
C	5.772154	-14.816301	0.571302
C	6.327541	-12.343405	-0.484345
C	7.047221	-14.558601	0.080748
C	7.326235	-13.307125	-0.457933
C	2.771233	-15.722950	0.465512
C	2.518961	-15.786666	-0.904187
C	2.600022	-16.920216	1.153857
C	2.099960	-16.942792	-1.556838
C	2.182024	-18.100720	0.542217
C	1.928496	-18.111229	-0.823683
F	0.746129	-14.444403	2.532343
F	0.312949	-14.120410	5.137210
F	2.401695	-13.796692	6.848643
F	4.938741	-13.736999	5.875978
F	5.405309	-14.037519	3.293252
F	4.174896	-11.645508	-0.037450
F	6.582652	-11.138332	-0.986174
F	8.534760	-13.035198	-0.932284
F	7.997012	-15.486729	0.124396
F	5.578041	-16.026033	1.106848
F	2.688905	-14.705308	-1.671495
F	2.845499	-17.001815	2.465018
F	2.029279	-19.215308	1.250621
F	1.531336	-19.225937	-1.423113
F	1.870441	-16.942983	-2.866432
H	0.428518	-10.850984	6.750188
H	0.014765	-10.971178	4.311718
H	2.765464	-10.630383	7.604755
H	2.470596	-11.547103	1.741319
S	2.065425	-9.200049	1.555841
C	0.537034	-8.261642	2.057840
C	-0.272962	-8.148335	0.761698
C	0.704544	-8.102799	-0.410374
C	1.665709	-9.289370	-0.266242
H	0.032326	-8.888652	2.805242
H	-0.941457	-9.015700	0.666509
H	-0.898943	-7.245715	0.802172

H	0.190575	-8.178344	-1.379186
H	1.265422	-7.155116	-0.407429
H	2.638283	-9.091364	-0.737645
C	1.110471	-10.617308	-0.749431
H	1.035191	-10.545575	-1.844736
H	1.768608	-11.462209	-0.515675
H	0.110624	-10.824085	-0.347510
C	0.964038	-6.941288	2.678104
H	0.061059	-6.406814	3.007947
H	1.606725	-7.098132	3.556261
H	1.497415	-6.301959	1.958572
Br	4.885544	-10.626990	3.098033

[BCFF]$^-$

35

B	4.526815	-0.669474	-1.044904
C	3.254680	0.285851	-1.490567
C	2.517324	1.110496	-0.646422
C	2.850217	0.300922	-2.825157
C	1.471313	1.921485	-1.085661
C	1.813618	1.093096	-3.308193
C	1.119155	1.915568	-2.428226
C	5.155708	-0.199971	0.409288
C	5.913793	0.967808	0.480798
C	4.994821	-0.868957	1.618919
C	6.505310	1.431757	1.652527
C	5.568328	-0.442603	2.815375
C	6.333346	0.716266	2.831460
C	4.140536	-2.276219	-1.052539
C	2.866830	-2.824053	-1.164924
C	5.174298	-3.207626	-0.958566
C	2.623506	-4.196767	-1.210058
C	4.979366	-4.584178	-0.998442
C	3.688275	-5.083607	-1.129065
F	1.778865	-2.047196	-1.221139
F	1.382709	-4.662377	-1.324508
F	3.477005	-6.393374	-1.169126
F	6.006099	-5.424116	-0.902006
F	6.435442	-2.797091	-0.790879
F	4.241379	-1.972016	1.702602
F	5.384513	-1.129050	3.939971
F	6.886817	1.140605	3.960942
F	7.218978	2.554237	1.659034
F	6.084544	1.732454	-0.601963
F	2.764509	1.156923	0.667845
F	0.806883	2.694472	-0.230866
F	0.126253	2.679773	-2.866314
F	1.472177	1.063411	-4.593403
F	3.439343	-0.500688	-3.717324
F	5.557250	-0.493679	-2.029876

[2a^{R}-A]$^+$ (= [2a-A]$^+$)

33

F	0.785915	-8.454108	0.908308
C	1.409210	-4.622493	3.993270
C	0.823346	-7.116763	2.863647
C	1.579789	-6.175357	2.157686
C	0.549568	-8.458959	2.239888
C	1.871929	-4.935143	2.717425
C	0.358038	-6.783292	4.144484
C	0.648588	-5.544655	4.709944
H	0.279331	-5.297759	5.706664
H	2.460975	-4.211446	2.151468
H	1.933058	-6.419554	1.154816
H	1.633959	-3.652083	4.440188
H	-0.472011	-8.824485	2.426943
S	1.657849	-9.712176	3.051733

C	3.321065	-9.300201	2.318781
C	3.743238	-10.605300	1.642076
C	2.508243	-11.225153	1.002558
C	1.418923	-11.279106	2.071160
H	3.106044	-8.542559	1.551700
H	4.525389	-10.379021	0.903558
H	4.176399	-11.290164	2.389033
H	2.165472	-10.620911	0.148809
H	2.700562	-12.245118	0.639852
H	1.676282	-12.034022	2.830506
C	-0.008056	-11.483162	1.603616
H	-0.066335	-12.481000	1.144649
H	-0.723793	-11.458704	2.438096
H	-0.302164	-10.745398	0.844413
C	4.251122	-8.746617	3.378536
H	5.220410	-8.531466	2.904432
H	3.867146	-7.808640	3.804614
H	4.418065	-9.469292	4.190756
Br	-0.688258	-8.007796	5.148294

[2a^{R}-A]$^{+}$-II

33

F	2.392324	-11.649100	-1.307396
C	0.626082	-15.061000	1.786625
C	1.585418	-12.678963	0.664049
C	2.315506	-13.347603	1.659448
C	2.084435	-11.430214	0.002670
C	1.836770	-14.528369	2.223275
C	0.377978	-13.243120	0.230443
C	-0.103226	-14.422894	0.785417
H	-1.047093	-14.843372	0.434774
H	2.409888	-15.036375	3.000442
H	0.257070	-15.985967	2.234107
H	2.952630	-10.980802	0.504684
S	0.759156	-10.125984	-0.079171
C	1.712285	-8.746849	-0.909460
C	1.740720	-7.626437	0.132247
C	1.839763	-8.259109	1.512158
C	0.748237	-9.321309	1.600983
H	2.719713	-9.166981	-1.044865
H	2.592115	-6.966575	-0.086580
H	0.821649	-7.024047	0.054192
H	2.828428	-8.721584	1.666995
H	1.686983	-7.525216	2.316575
H	-0.243673	-8.842911	1.576252
C	0.819988	-10.290629	2.762448
H	0.631444	-9.714739	3.680330
H	0.060289	-11.081958	2.698197
H	1.815495	-10.745655	2.857906
C	1.095505	-8.406142	-2.250285
H	1.685318	-7.594947	-2.702513
H	1.118388	-9.265638	-2.934420
H	0.058192	-8.056501	-2.142845
H	-0.194188	-12.750177	-0.559481
Br	3.985310	-12.691633	2.283200

[2a^{R}-A]$^{+}$-III

33

F	3.356996	-10.827364	1.445876
C	0.975297	-15.227075	1.693907
C	1.687432	-12.525358	1.444851
C	2.236384	-13.494304	0.586313
C	2.022367	-11.064174	1.386413
C	1.881419	-14.835342	0.709697
C	0.790504	-12.946611	2.434130
C	0.429943	-14.284043	2.560558
H	-0.270851	-14.587278	3.340057

H	2.315806	-15.578431	0.039061
H	0.703773	-16.280995	1.781406
H	1.523164	-10.502520	2.189203
S	1.448841	-10.314222	-0.239925
C	2.349025	-8.673574	-0.215707
C	1.232217	-7.629824	-0.225407
C	0.084349	-8.161902	0.617110
C	-0.224730	-9.568134	0.116301
H	2.863518	-8.668612	0.755111
H	1.634861	-6.684025	0.164030
H	0.898077	-7.450816	-1.259857
H	0.359669	-8.194292	1.684144
H	-0.821192	-7.544805	0.525066
H	-0.624282	-9.518350	-0.908989
C	-1.137150	-10.418097	0.974932
H	-0.805214	-10.454172	2.021520
H	-2.131183	-9.947558	0.958276
H	-1.243873	-11.441553	0.588713
C	3.349804	-8.598994	-1.350205
H	3.837131	-7.613271	-1.312731
H	4.131124	-9.365174	-1.253169
H	2.862156	-8.703526	-2.330566
H	0.372759	-12.205134	3.117905
Br	3.492959	-13.044669	-0.761721

[2a^{S}-A]$^{+}$
33

C	4.232768	-4.257114	6.740151
C	3.612934	-6.985057	6.529028
C	3.490465	-6.308898	7.750290
C	3.232861	-8.436967	6.502981
C	3.790924	-4.955240	7.860456
C	4.064318	-6.263699	5.409514
C	4.375180	-4.910037	5.516955
H	4.729160	-4.361715	4.642465
H	3.684758	-4.450656	8.822105
H	3.154510	-6.863109	8.630372
H	4.475601	-3.194961	6.810697
S	1.704491	-8.680885	5.452896
C	0.469044	-7.652519	6.401749
C	-0.644507	-8.643494	6.734341
C	0.017407	-9.958657	7.117120
C	0.994826	-10.319560	6.002069
H	0.997005	-7.377681	7.327246
H	-1.251684	-8.226717	7.550486
H	-1.303848	-8.778917	5.861792
H	0.557006	-9.859527	8.073108
H	-0.710956	-10.774664	7.228993
H	0.438481	-10.603987	5.095171
C	2.036596	-11.373431	6.317900
H	1.499521	-12.313480	6.512704
H	2.723615	-11.544779	5.478518
H	2.615162	-11.128484	7.219690
C	0.082608	-6.414688	5.620151
H	-0.654592	-5.851683	6.211655
H	0.947502	-5.757977	5.445716
H	-0.375220	-6.671545	4.653551
Br	4.277950	-7.074320	3.708724
F	4.181873	-9.240130	5.968002
H	2.976039	-8.796523	7.510304

[2a^{S}-A]$^{+}$-II
33

C	6.104692	-5.764463	6.486477
C	3.415490	-6.547359	6.603586
C	4.283613	-6.813845	7.667239
C	1.968540	-6.953090	6.701177

C	5.619044	-6.427614	7.610815
C	3.915763	-5.857203	5.488623
C	5.252687	-5.473024	5.423428
H	5.626794	-4.939099	4.548437
H	6.279681	-6.644594	8.451982
H	3.901989	-7.323004	8.552876
H	7.151499	-5.459190	6.433090
S	1.555116	-8.312142	5.491814
C	-0.304219	-8.390553	5.676457
C	-0.576330	-9.830187	6.113454
C	0.546767	-10.265078	7.042376
C	1.861519	-9.949736	6.336596
H	-0.520828	-7.701949	6.506643
H	-1.560350	-9.866968	6.601943
H	-0.617023	-10.485307	5.228576
H	0.496741	-9.728035	8.001744
H	0.511252	-11.343454	7.254773
H	1.962393	-10.588174	5.443787
C	3.127866	-10.027310	7.161780
H	3.044982	-9.448790	8.089981
H	3.272183	-11.083587	7.432913
H	4.015211	-9.702385	6.601012
C	-0.989806	-7.935306	4.404512
H	-2.076769	-8.015877	4.555209
H	-0.761402	-6.887037	4.166236
H	-0.714863	-8.567324	3.547128
Br	2.783765	-5.389995	4.036480
F	1.629123	-7.348078	7.947845
H	1.292680	-6.140843	6.384913

[2a^{S}-A]$^{+}$-III

33

C	5.970100	-5.869672	6.492472
C	3.218648	-6.384970	6.411586
C	3.888858	-6.261107	7.636520
C	1.742179	-6.645549	6.430703
C	5.253451	-6.003432	7.680597
C	3.955642	-6.243814	5.225882
C	5.325276	-5.989130	5.263978
H	5.888413	-5.880046	4.335920
H	5.757394	-5.906098	8.643517
H	3.326419	-6.360313	8.567687
H	7.042891	-5.668194	6.514994
S	1.442555	-8.363748	7.091210
C	2.224266	-9.478986	5.814846
C	1.040306	-9.981453	4.984924
C	-0.157812	-10.127092	5.917292
C	-0.329380	-8.804231	6.674602
H	2.902787	-8.841100	5.232658
H	0.815529	-9.267667	4.178286
H	1.310208	-10.937741	4.515132
H	-1.084983	-10.345107	5.368497
H	0.006709	-10.952078	6.627032
H	-0.785364	-8.951326	7.662989
C	-1.074618	-7.726237	5.907934
H	-2.119950	-8.057581	5.824316
H	-1.073321	-6.763109	6.434609
H	-0.685431	-7.587020	4.889021
C	3.017667	-10.551617	6.542488
H	3.503892	-11.186944	5.788021
H	3.801318	-10.111180	7.175199
H	2.372892	-11.189839	7.165213
Br	3.133432	-6.387474	3.518962
F	1.097124	-5.834508	7.307117
H	1.270600	-6.569540	5.443095

[2h]$^{+}$

14
C -2.891206 5.270919 -0.680510
F -2.887945 4.469104 -1.673334
C -4.029608 5.977637 -0.339381
C -4.045687 6.879361 0.781620
C -5.218690 5.806257 -1.117849
C -5.207076 7.560243 1.088104
C -6.363766 6.494690 -0.795650
H -5.199903 5.120533 -1.966300
C -6.351453 7.364531 0.303560
H -5.242919 8.248616 1.933392
H -7.272032 6.365297 -1.384390
H -7.259678 7.912513 0.565161
H -1.930634 5.325151 -0.155176
Br -2.529577 7.160586 1.848950

A···2a···BCF
68
F 0.861348 -11.599959 0.353369
C 1.212594 -10.752241 5.180922
C 1.757157 -11.450720 2.519481
C 0.495775 -11.684290 3.079584
C 1.976844 -11.841715 1.081219
C 0.219218 -11.336983 4.396986
C 2.746857 -10.875391 3.324485
C 2.478019 -10.523391 4.647860
H 3.260101 -10.075877 5.263312
F 2.183495 -13.193641 0.992669
B 4.101378 -15.185591 1.714294
C 3.794319 -14.573124 3.116421
C 4.783896 -13.939609 3.884816
C 2.511949 -14.611539 3.691400
C 4.531805 -13.398715 5.140664
C 2.232295 -14.092705 4.946651
C 3.248227 -13.473205 5.668154
C 5.458230 -14.872432 0.951160
C 5.736360 -13.714892 0.235401
C 6.458055 -15.838218 0.993500
C 6.952302 -13.517767 -0.411949
C 7.688493 -15.675438 0.368065
C 7.932547 -14.502751 -0.340676
C 3.175291 -16.264282 1.035438
C 2.845713 -16.190935 -0.318886
C 2.711875 -17.383594 1.729167
C 2.072687 -17.158117 -0.952810
C 1.954535 -18.378191 1.123860
C 1.631078 -18.258541 -0.225179
F 1.489656 -15.142504 3.038548
F 1.010482 -14.155646 5.450438
F 2.989093 -12.947045 6.846798
F 5.491268 -12.793393 5.822459
F 6.029057 -13.839712 3.445531
F 4.824021 -12.753001 0.135034
F 7.182024 -12.405455 -1.093550
F 9.093683 -14.325605 -0.947430
F 8.619905 -16.614337 0.440751
F 6.244765 -16.962255 1.681645
F 3.252711 -15.160652 -1.052056
F 3.008476 -17.537709 3.016191
F 1.540958 -19.431543 1.811553
F 0.904893 -19.190254 -0.813522
F 1.761970 -17.044770 -2.234924
H -0.771265 -11.527198 4.813688
H -0.280118 -12.136877 2.459252
H 1.009857 -10.477326 6.217727
H 2.824527 -11.332144 0.606721
S 2.055681 -8.166421 0.788041

C	1.007473	-7.909380	2.289077
C	-0.394188	-7.600374	1.750113
C	-0.229495	-6.802046	0.458974
C	0.792411	-7.545698	-0.408243
H	0.994917	-8.859020	2.839781
H	-0.923926	-8.543352	1.541286
H	-0.985147	-7.053129	2.501324
H	-1.183796	-6.685771	-0.078711
H	0.143274	-5.791288	0.689191
H	1.311059	-6.842523	-1.077082
C	0.186332	-8.671196	-1.237456
H	-0.504259	-8.255934	-1.990032
H	0.964416	-9.238704	-1.769373
H	-0.371986	-9.379275	-0.607681
C	1.581639	-6.824831	3.190957
H	0.983232	-6.743175	4.113439
H	2.617195	-7.059070	3.481553
H	1.584973	-5.843080	2.691752
Br	4.491249	-10.518469	2.668849

TS-2

68

F	1.135963	-11.814472	0.295923
C	1.256550	-10.963046	5.161299
C	1.861378	-11.453911	2.487031
C	0.556435	-11.651954	2.966413
C	2.143304	-11.692928	1.087860
C	0.254498	-11.405126	4.297911
C	2.862806	-11.001051	3.369501
C	2.558857	-10.759076	4.703507
H	3.332382	-10.410889	5.389578
F	2.622256	-13.415635	0.973438
B	3.598089	-14.496620	1.536343
C	3.511341	-14.307676	3.151054
C	4.566771	-13.989393	4.002694
C	2.263853	-14.409918	3.770324
C	4.403313	-13.783143	5.372857
C	2.056808	-14.210505	5.127817
C	3.140672	-13.889746	5.937331
C	5.065926	-14.290815	0.857085
C	5.446426	-13.313294	-0.054972
C	6.040638	-15.249542	1.134710
C	6.713829	-13.263706	-0.635583
C	7.314155	-15.241023	0.582074
C	7.652875	-14.234856	-0.317607
C	2.954478	-15.879885	0.951744
C	2.650827	-15.970180	-0.407637
C	2.732222	-17.041522	1.688861
C	2.129589	-17.114909	-1.002578
C	2.216337	-18.209173	1.131024
C	1.911756	-18.245576	-0.223863
F	1.182094	-14.696725	3.041849
F	0.840018	-14.290233	5.651859
F	2.960503	-13.659739	7.228447
F	5.442710	-13.460973	6.132811
F	5.810202	-13.841744	3.548580
F	4.601853	-12.349069	-0.441391
F	7.021674	-12.301127	-1.495882
F	8.859916	-14.203627	-0.861763
F	8.201541	-16.175296	0.897709
F	5.763106	-16.232345	1.996492
F	2.864467	-14.929018	-1.214605
F	3.015511	-17.095994	2.990854
F	2.019266	-19.286207	1.881626
F	1.420511	-19.347738	-0.769119
F	1.851418	-17.140102	-2.300306
H	-0.759431	-11.565178	4.666304

H	-0.216311	-12.004502	2.282293
H	1.030059	-10.777431	6.213109
H	3.059813	-11.345456	0.612041
S	2.151428	-8.488711	0.695536
C	1.267770	-7.992466	2.241951
C	-0.154295	-7.639230	1.790257
C	-0.055696	-6.997520	0.408574
C	0.826803	-7.905567	-0.455190
H	1.244793	-8.872711	2.899372
H	-0.763004	-8.555600	1.734667
H	-0.635190	-6.971572	2.521889
H	-1.044932	-6.865796	-0.056852
H	0.404660	-6.000378	0.491139
H	1.333338	-7.320092	-1.236514
C	0.071697	-9.058781	-1.102431
H	-0.654796	-8.665218	-1.831884
H	0.753712	-9.734551	-1.638973
H	-0.478861	-9.651574	-0.356340
C	1.994641	-6.860906	2.956411
H	1.489240	-6.631589	3.908686
H	3.034863	-7.138247	3.185043
H	2.014755	-5.944647	2.346123
Br	4.625080	-10.678385	2.776884

TS-3

52

C	4.162329	1.415217	1.345839
C	3.676924	2.482304	0.373815
C	3.403766	1.794195	-0.965628
C	3.163712	0.259746	1.271206
H	4.229577	1.789452	2.378750
H	5.164243	1.058743	1.053486
H	2.743763	2.935043	0.748554
H	4.409356	3.292546	0.239499
H	4.358757	1.598814	-1.477660
H	2.237137	0.565683	1.784232
S	2.752139	0.118983	-0.520795
C	3.636184	-1.065295	1.841164
H	2.862278	-1.842823	1.748337
H	3.862411	-0.944152	2.912194
H	4.546302	-1.417619	1.332008
C	2.477229	2.548763	-1.904086
H	2.285804	1.985607	-2.829778
H	2.947051	3.503508	-2.187621
H	1.513911	2.777454	-1.423705
C	0.068723	0.452382	-0.159928
F	0.202640	1.533636	0.542398
C	-0.040253	-0.800813	0.537068
C	-0.066430	-2.027801	-0.159919
C	-0.106344	-0.808166	1.942979
C	-0.137697	-3.226650	0.535194
C	-0.187337	-2.007995	2.634203
H	-0.103907	0.139235	2.484274
C	-0.197936	-3.212472	1.930142
H	-0.153915	-4.174784	-0.004116
H	-0.242469	-2.006261	3.723690
H	-0.257795	-4.160661	2.468101
H	0.138640	0.576500	-1.236858
Br	-0.033078	-2.068454	-2.045152
C	-4.136164	0.708215	1.305875
C	-4.034321	2.197842	1.012977
C	-2.677340	2.432002	0.349063
C	-3.785527	-0.037485	0.018650
H	-5.140680	0.414794	1.646310
H	-3.420744	0.427833	2.097483
H	-4.841367	2.508389	0.328565
H	-4.115123	2.810733	1.923432

S	-2.451785	0.983601	-0.776723
H	-4.624966	0.042130	-0.689260
H	-1.890160	2.354207	1.114714
C	-3.427278	-1.500558	0.203469
H	-3.110662	-1.971961	-0.738357
H	-4.320487	-2.038357	0.557317
H	-2.637285	-1.633996	0.955375
C	-2.530449	3.745653	-0.397627
H	-1.532050	3.847288	-0.848688
H	-2.665733	4.581670	0.306176
H	-3.284302	3.838319	-1.194162

[NTf2]$^{-}$

15

S	1.621208	0.612205	0.158624
S	1.621208	-0.044956	-2.598748
O	0.247025	0.147320	0.277893
O	0.247025	0.419929	-2.718017
F	0.781934	2.856330	-0.952738
F	0.781934	-2.289081	-1.487387
N	2.360376	0.283625	-1.220062
F	2.684687	3.021227	0.041678
F	2.684687	-2.453978	-2.481803
F	0.872280	2.893517	1.200605
F	0.872280	-2.326268	-3.640730
C	1.482767	2.464705	0.101760
C	1.482767	-1.897456	-2.541885
O	2.546869	0.366249	1.251628
O	2.546868	0.201000	-3.691752

[2a-A]$^{+}$···[NTf$_2$]$^{-}$

48

F	-0.000685	-10.687954	1.192016
C	3.372542	-11.745899	4.669791
C	2.041033	-10.830845	2.383812
C	1.319916	-11.303635	3.484590
C	1.300266	-10.313764	1.177349
C	1.980498	-11.759144	4.622252
C	3.443221	-10.827440	2.444256
C	4.107805	-11.282488	3.580268
H	5.198445	-11.275852	3.614139
H	1.403261	-12.126045	5.472833
H	0.229851	-11.319825	3.440923
H	3.898392	-12.100181	5.558684
H	1.761577	-10.625220	0.227360
S	1.442140	-8.468006	1.195293
C	0.067669	-7.964370	2.353912
C	-0.930769	-7.224509	1.449783
C	-0.178405	-6.674931	0.239110
C	0.652446	-7.817145	-0.349641
H	-0.354224	-8.900691	2.742209
H	-1.719426	-7.918233	1.125070
H	-1.410003	-6.419081	2.023655
H	-0.858263	-6.284981	-0.531978
H	0.494609	-5.856961	0.541891
H	1.513558	-7.445037	-0.913403
C	-0.115198	-8.835798	-1.170972
H	-0.487787	-8.310568	-2.063202
H	0.532586	-9.650819	-1.522609
H	-0.973553	-9.264904	-0.638929
C	0.658528	-7.137986	3.483307
H	-0.159163	-6.853551	4.162305
H	1.393832	-7.715377	4.061451
H	1.139617	-6.223287	3.108528
Br	4.465450	-10.196117	0.978416
S	4.178118	-6.440120	-1.020245
S	3.600923	-4.945249	1.323050

O	5.588648	-6.110284	-0.909594
O	4.505337	-3.890798	0.895768
F	3.688992	-4.043549	-2.010796
F	5.674090	-6.381445	2.082775
N	3.261482	-6.063079	0.234556
F	2.224754	-5.531576	-2.537413
F	3.842265	-6.897608	3.086206
F	4.164014	-5.548825	-3.479255
F	4.872907	-5.077306	3.601418
C	3.521040	-5.311941	-2.345032
C	4.561052	-5.893250	2.603633
O	3.792311	-7.768035	-1.479436
O	2.363020	-4.579170	1.999536

[2a-A]$^{+}$···[NTf$_2$]$^{-}$-II

48

F	-0.061382	-10.367556	1.442597
C	3.562425	-11.436579	4.648010
C	2.080142	-10.612906	2.419060
C	1.449656	-10.851707	3.644352
C	1.262009	-10.166932	1.235126
C	2.184809	-11.261222	4.753225
C	3.467763	-10.799841	2.328133
C	4.208262	-11.207506	3.434454
H	5.287009	-11.348072	3.349020
H	1.676224	-11.445213	5.701257
H	0.368993	-10.722743	3.721090
H	4.146547	-11.756737	5.513083
H	1.563758	-10.672600	0.305700
S	1.599779	-8.363123	0.943622
C	0.354327	-7.564966	2.080499
C	-0.816416	-7.219621	1.156130
C	-0.248086	-6.829083	-0.204133
C	0.730986	-7.920688	-0.652164
H	0.085341	-8.342910	2.806851
H	-1.480129	-8.090790	1.057105
H	-1.400176	-6.401891	1.602656
H	-1.036190	-6.720945	-0.962962
H	0.289463	-5.872710	-0.137637
H	1.546997	-7.501326	-1.255400
C	0.106548	-9.102942	-1.371290
H	-0.320092	-8.717814	-2.309058
H	0.854013	-9.860184	-1.645805
H	-0.699408	-9.577266	-0.795680
C	0.997770	-6.388167	2.794636
H	0.256201	-5.972119	3.492641
H	1.874318	-6.705337	3.377397
H	1.311188	-5.602198	2.095810
Br	4.380371	-10.500975	0.691663
S	3.577122	-5.482011	-0.922542
S	6.331038	-5.543296	-1.562159
O	3.275116	-6.324681	-2.075474
O	6.005078	-5.430573	-2.974801
F	3.744203	-3.385626	-2.506098
F	5.653632	-8.094311	-1.592114
N	5.108769	-5.308226	-0.548928
F	3.256386	-2.911802	-0.462068
F	7.059547	-7.571605	-0.046822
F	1.766579	-3.792343	-1.747520
F	7.711319	-7.697799	-2.097267
C	3.060009	-3.776663	-1.444251
C	6.708835	-7.345484	-1.303411
O	2.817713	-5.706639	0.304174
O	7.497029	-4.855808	-1.039408

[2a-A]$^{+}$···[NTf$_2$]$^{-}$-III

48

F	0.373726	-10.205682	0.793185
C	3.898209	-11.128727	4.378888
C	2.242988	-10.167252	2.316361
C	3.602978	-9.828537	2.382334
C	1.485206	-9.536535	1.170141
C	4.425487	-10.289555	3.400431
C	1.729989	-11.018766	3.308040
C	2.558134	-11.497766	4.326336
H	2.147013	-12.166388	5.084276
H	5.476112	-9.996067	3.421020
H	4.020065	-9.184764	1.607835
H	4.528793	-11.508290	5.185304
H	2.156217	-9.415748	0.304604
S	1.055093	-7.788601	1.636337
C	-0.543658	-7.968918	2.555236
C	-1.618249	-7.710564	1.497530
C	-1.100351	-6.610571	0.575564
C	0.313932	-6.988134	0.118216
H	-0.573420	-9.002249	2.924568
H	-1.802659	-8.632556	0.926733
H	-2.559377	-7.425394	1.989502
H	-1.740480	-6.483321	-0.309034
H	-1.076608	-5.646762	1.105605
H	0.960091	-6.109585	0.001165
C	0.373743	-7.851881	-1.128491
H	0.029877	-7.231508	-1.968995
H	1.396756	-8.174675	-1.356476
H	-0.278259	-8.732268	-1.059157
C	-0.529973	-6.993127	3.723682
H	-1.470438	-7.105441	4.282859
H	0.303805	-7.203110	4.408873
H	-0.451489	-5.947474	3.390178
Br	-0.071582	-11.628080	3.359459
S	4.437505	-7.750182	-0.863738
S	3.890773	-5.607506	0.923048
O	5.861019	-7.720868	-0.579661
O	4.935376	-4.833538	0.273925
F	4.972902	-5.728282	-2.480413
F	5.650342	-7.014165	2.304496
N	3.498942	-6.973960	0.176400
F	2.975939	-6.505481	-2.676742
F	3.749190	-6.775620	3.288848
F	4.649716	-7.618582	-3.457349
F	5.114533	-5.114937	3.170850
C	4.245843	-6.828036	-2.474954
C	4.651356	-6.175451	2.526662
O	3.794844	-9.036515	-1.123458
O	2.658753	-4.951796	1.336340

N*s*

26

N	-1.920897	-5.563156	-0.474418
C	-0.730913	-4.999795	-1.080609
H	0.013933	-4.768174	-0.304254
H	-0.272715	-5.712603	-1.780861
H	-0.938868	-4.061518	-1.642590
C	-2.461374	-4.659851	0.520781
H	-1.658501	-4.359224	1.210347
C	-2.886327	-5.992068	-1.485462
H	-3.109150	-5.146768	-2.177008
C	-4.218283	-6.444612	-0.880378
H	-4.770656	-5.612581	-0.420966
H	-4.854985	-6.866040	-1.671517
H	-4.057495	-7.224860	-0.119324
C	-2.318809	-7.126793	-2.327014
C	-2.485540	-7.139677	-3.714698

C	-1.672019	-8.209197	-1.717728
C	-2.021413	-8.210875	-4.480756
H	-2.983678	-6.297384	-4.203932
C	-1.204988	-9.278979	-2.479019
H	-1.523704	-8.194558	-0.635194
C	-1.379636	-9.284676	-3.864799
H	-2.158604	-8.202741	-5.564990
H	-0.699140	-10.114602	-1.988522
H	-1.012542	-10.122798	-4.462201
H	-2.889143	-3.730369	0.081614
H	-3.242697	-5.142334	1.123442

[2a-N*s*]$^{+}$

40

F	-0.331739	-6.663609	0.013890
C	0.164979	-4.047388	4.133447
C	-0.880471	-5.423729	1.920327
C	0.303811	-4.682348	1.814734
C	-1.384304	-6.178574	0.713361
C	0.821076	-3.993124	2.905900
C	-1.507065	-5.492517	3.174181
C	-0.995318	-4.804219	4.272689
H	-1.496117	-4.867185	5.239935
H	1.744134	-3.421207	2.797465
H	0.839593	-4.662268	0.864275
H	0.562308	-3.512291	4.998200
H	-2.019309	-7.019133	1.017553
Br	-3.062238	-6.549130	3.464050
N	-2.222179	-5.345281	-0.268679
C	-1.333589	-4.537506	-1.162969
H	-0.751483	-3.842439	-0.549661
H	-0.669755	-5.195101	-1.727955
H	-1.975724	-3.968742	-1.844059
C	-3.089810	-4.379833	0.477648
H	-3.644340	-4.893915	1.267304
H	-2.459032	-3.598054	0.912185
H	-3.791964	-3.934564	-0.236529
C	-3.140837	-6.250727	-1.152315
H	-3.614137	-5.506952	-1.806374
C	-4.240836	-6.938996	-0.356281
H	-4.910502	-6.227758	0.142718
H	-4.847569	-7.503408	-1.077526
H	-3.870096	-7.663050	0.382336
C	-2.358737	-7.203407	-2.035760
C	-2.086497	-6.835665	-3.358581
C	-1.951977	-8.467068	-1.589846
C	-1.402761	-7.699260	-4.213079
H	-2.417556	-5.861074	-3.726862
C	-1.266417	-9.330313	-2.440761
H	-2.162912	-8.787094	-0.566747
C	-0.987782	-8.948008	-3.753582
H	-1.199779	-7.396111	-5.242533
H	-0.950762	-10.310414	-2.076636
H	-0.452992	-9.628011	-4.420569

[2a-N*s*]$^{+}$-II

40

F	-0.506076	-7.557388	-0.091850
C	2.543269	-4.196027	2.128996
C	0.427395	-5.628876	0.944645
C	0.229547	-4.833923	2.084279
C	-0.801183	-6.347523	0.427815
C	1.263036	-4.113203	2.669007
C	1.735360	-5.726760	0.435340
C	2.778354	-5.008533	1.024353
H	3.787197	-5.094151	0.617626
H	1.068297	-3.500415	3.550679

H -0.769835 -4.783976 2.522228
H 3.372046 -3.642507 2.574713
H -1.496765 -6.484502 1.263489
Br 2.235295 -6.843970 -1.018250
N -1.605030 -5.598090 -0.632390
C -0.772774 -5.280445 -1.827902
H 0.035982 -4.607464 -1.520990
H -0.373004 -6.201437 -2.258737
H -1.407521 -4.769116 -2.559729
C -2.072214 -4.297157 -0.053687
H -2.659353 -4.477181 0.853046
H -1.202713 -3.673320 0.176776
H -2.686501 -3.794237 -0.807886
C -2.886331 -6.411812 -1.059648
H -3.532446 -5.602796 -1.419308
C -3.576256 -7.077361 0.121153
H -3.801625 -6.371170 0.931719
H -4.535281 -7.467057 -0.246747
H -3.011043 -7.925361 0.530163
C -2.671126 -7.344523 -2.239491
C -3.006288 -6.882282 -3.519200
C -2.229899 -8.666451 -2.100150
C -2.874796 -7.703942 -4.636997
H -3.380524 -5.862126 -3.641963
C -2.097066 -9.489594 -3.216487
H -1.983210 -9.067104 -1.117325
C -2.413053 -9.010658 -4.487958
H -3.140145 -7.322485 -5.625417
H -1.747122 -10.516456 -3.089435
H -2.308549 -9.659729 -5.360382

[2a-N$_s$]$^+$-III
40
F -1.783034 -7.756040 0.634097
C 2.785476 -7.419832 -1.227225
C 0.262587 -6.861625 -0.120676
C 0.427885 -7.883602 -1.061943
C -1.093910 -6.603736 0.490254
C 1.672132 -8.159765 -1.617191
C 1.404174 -6.149321 0.282067
C 2.655196 -6.417671 -0.269337
H 3.529240 -5.850084 0.053577
H 1.768069 -8.959755 -2.353356
H -0.434438 -8.477816 -1.359018
H 3.767726 -7.626206 -1.656930
H -0.970896 -6.160557 1.485908
Br 1.324991 -4.790390 1.613561
N -2.011864 -5.606601 -0.267842
C -1.192869 -4.702092 -1.133826
H -0.457570 -4.178931 -0.513617
H -0.681306 -5.280089 -1.906516
H -1.867074 -3.967378 -1.586589
C -2.657854 -4.743558 0.775191
H -3.141089 -5.374272 1.527913
H -1.881119 -4.131448 1.247442
H -3.391142 -4.090077 0.289611
C -3.149127 -6.247823 -1.149751
H -3.471162 -5.366735 -1.720955
C -4.345825 -6.722660 -0.334475
H -4.853968 -5.893690 0.171193
H -5.066128 -7.153825 -1.042829
H -4.094523 -7.489361 0.406970
C -2.647668 -7.274474 -2.152332
C -2.082594 -6.853346 -3.363140
C -2.810775 -8.650030 -1.937232
C -1.625695 -7.777863 -4.301158
H -2.006971 -5.787807 -3.590513

C	-2.369043	-9.575703	-2.879787
H	-3.276678	-9.015420	-1.022116
C	-1.759031	-9.143498	-4.057611
H	-1.177337	-7.426200	-5.232800
H	-2.500296	-10.643026	-2.689130
H	-1.404836	-9.870232	-4.792116

TS-4

66

F	0.033072	-6.950179	0.295285
C	-0.238017	-3.634462	3.983998
C	-0.655394	-5.733170	2.185237
C	0.459657	-4.884971	2.051687
C	-0.840958	-6.798754	1.236772
C	0.667326	-3.842875	2.943860
C	-1.559147	-5.509208	3.245638
C	-1.348552	-4.464649	4.137525
H	-2.048659	-4.294211	4.956623
H	1.535670	-3.192393	2.829688
H	1.163946	-5.057420	1.236542
H	-0.083400	-2.817253	4.691379
H	-1.659186	-7.514021	1.259681
Br	-3.069832	-6.614083	3.502168
N	-2.180531	-5.460027	-0.278007
C	-1.150220	-4.824584	-1.092905
H	-0.483875	-4.237922	-0.444886
H	-0.553073	-5.570120	-1.630141
H	-1.594722	-4.134054	-1.834296
C	-2.865328	-4.430476	0.499013
H	-3.564237	-4.870100	1.219116
H	-2.130525	-3.830559	1.052080
H	-3.430985	-3.747794	-0.162531
C	-3.152784	-6.221566	-1.113851
H	-3.714781	-5.466270	-1.697573
C	-4.163835	-6.997239	-0.273302
H	-4.800158	-6.334502	0.328157
H	-4.824461	-7.568289	-0.940813
H	-3.674236	-7.710529	0.404792
C	-2.465928	-7.119518	-2.130665
C	-2.454886	-6.765017	-3.484094
C	-1.848527	-8.316397	-1.754788
C	-1.831140	-7.576092	-4.432715
H	-2.938485	-5.836591	-3.800207
C	-1.218437	-9.129066	-2.694455
H	-1.860303	-8.622697	-0.709425
C	-1.206466	-8.759351	-4.039993
H	-1.833760	-7.280462	-5.484468
H	-0.739268	-10.057514	-2.374250
H	-0.716259	-9.395088	-4.780981
N	0.365620	-8.444787	2.571914
C	0.336324	-8.025632	3.968441
H	0.736909	-7.007150	4.057904
H	-0.685584	-8.033444	4.363346
H	0.957718	-8.690987	4.597319
C	1.724099	-8.282526	2.063787
H	1.773240	-8.449901	0.982619
H	2.066449	-7.258423	2.268715
H	2.424264	-8.980977	2.560485
C	-0.122278	-9.845848	2.406294
H	0.486643	-10.470234	3.090569
C	0.102074	-10.369519	0.989220
H	1.167047	-10.531017	0.778857
H	-0.405663	-11.336813	0.872745
H	-0.293522	-9.681178	0.229044
C	-1.570814	-10.003158	2.854211
C	-1.866281	-10.318679	4.186421
C	-2.638011	-9.865472	1.959828

C	-3.184696	-10.448287	4.620209
H	-1.049244	-10.465006	4.897839
C	-3.959299	-9.998159	2.384664
H	-2.443671	-9.659694	0.906593
C	-4.238108	-10.280826	3.721008
H	-3.389686	-10.687741	5.666276
H	-4.772933	-9.880759	1.664911
H	-5.271947	-10.383877	4.058716

TS-5

48

S	-1.725672	0.063105	-16.878345
S	-0.670288	2.714334	-16.978734
O	-1.685744	0.061880	-18.326918
O	-1.600743	3.003294	-18.050282
F	-3.917369	1.528163	-16.994395
F	0.990801	1.274650	-18.449503
N	-0.939282	1.314466	-16.200207
F	-3.621120	0.524270	-15.114239
F	1.934535	2.418799	-16.886890
F	-4.243805	-0.597485	-16.847216
F	1.202681	3.409814	-18.654890
C	-3.497441	0.411903	-16.429305
C	0.976835	2.424661	-17.802848
O	-0.384034	3.720487	-15.969786
C	-0.548165	-2.585477	-12.790016
C	-0.383684	0.064229	-13.634025
C	-1.517952	-0.392189	-12.936183
H	-2.333353	0.301506	-12.729374
C	-0.318560	1.434662	-14.043814
C	-1.598399	-1.709487	-12.514448
H	-2.479402	-2.057177	-11.973315
C	0.663531	-0.839535	-13.915562
C	0.581711	-2.156363	-13.488390
H	1.390542	-2.855994	-13.702763
H	-0.604082	-3.625559	-12.462148
O	-1.398114	-1.139369	-16.138671
S	1.338641	2.068110	-11.797604
C	1.447475	3.915109	-11.817026
C	-0.070597	2.001820	-10.607713
C	-0.143877	3.409125	-10.011385
C	0.163373	4.390327	-11.134892
H	2.311788	4.167018	-11.182546
H	-0.982076	1.821854	-11.201061
H	-1.135341	3.580521	-9.564577
H	0.605197	3.507191	-9.207385
H	-0.659815	4.392822	-11.868775
H	0.285519	5.421433	-10.769214
Br	2.185340	-0.290838	-14.876708
C	1.673907	4.460984	-13.216680
H	1.806145	5.553475	-13.166093
H	2.580563	4.039380	-13.677088
H	0.820926	4.254402	-13.879943
C	0.095904	0.876293	-9.601697
H	-0.765558	0.863352	-8.915122
H	0.142933	-0.105391	-10.097559
H	1.012497	1.007026	-9.005953
F	-1.270370	2.220588	-13.682313
H	0.548651	1.918628	-14.483693

2a-NTf_2

29

S	0.599050	2.443673	-4.400870
S	0.264890	4.549365	-2.381722
O	0.059837	3.374693	-5.354733
O	-1.075351	4.352745	-2.879380
F	-1.976099	1.955184	-4.084357

F	1.931771	6.578094	-2.535737
N	1.216958	3.315943	-3.055428
F	-0.781318	1.467565	-2.353956
F	-0.086556	6.985916	-3.163256
F	-0.710476	0.218461	-4.104259
F	1.236508	5.872587	-4.449234
C	-0.842786	1.451138	-3.667533
C	0.899993	6.116595	-3.205205
O	0.570843	4.658530	-0.979607
C	5.226873	5.872791	-1.271125
C	3.392612	3.879062	-1.990014
C	3.535635	4.269287	-0.655996
H	2.919406	3.788690	0.103529
C	2.400878	2.797090	-2.358889
C	4.443076	5.260171	-0.296044
H	4.535258	5.554277	0.751033
C	4.203783	4.490698	-2.955809
C	5.113009	5.486028	-2.604044
H	5.733954	5.954653	-3.369221
H	5.939333	6.653967	-0.998451
F	2.000386	2.106282	-1.256528
O	1.607321	1.467271	-4.736065
H	2.859982	2.085868	-3.059164
Br	4.109017	3.982236	-4.782071

References

1. A. G. Massey, A. J. Park, *J. Organomet. Chem.* **1964**, *2*, 245.
2. G. S. Hair, A. H. Cowley, R. A. Jones, B. G. McBurnett, A. Voigt, *J. Am. Chem. Soc.* **1999**, *121*, 4922.
3. K. Julienne, P. Metzner, V. Henryon, A. Greiner, *J. Org. Chem.* **1998**, *63*, 4532.
4. (a) L. Curless, E. R. Clark, J. J. Dunsford, M. J.; Ingleson, *Chem. Commun.* **2014**, *50*, 5270; (b) I. Jalsovszky, F. Ruff, M. Kajtar-Peredy, I. Kiivesdi, A. Kucsman, *Tetrahedron* **1986**, *42*, 5649.
5. (a) D. N. Kursanov, Z. N. Parnes, G. I. Bolestova, L. I. Belenkii, *Tetrahedron*, **1974**, *31*, 311; (b) A. Coste, J. Kim, T. C. Adams, M. Movassaghi, *Chem. Sci.* **2013**, *4*, 3191; (c) A. W. D. Avison, F. Bergel, A. Cohen, J. W. Haworth, *Nature* **1944**, *154*, 459.
6. R. Gupta, D. Mandal, A. K. Jaiswal, R. D. Young, *Org. Lett.* **2021**, *23*, 1915.
7. Gaussian 16, Revision A.03, M. J. Frisch, G. W. Trucks, H. B. Schlegel, G. E. Scuseria, M. A. Robb, J. R. Cheeseman, G. Scalmani, V. Barone, G. A. Petersson, H. Nakatsuji, X. Li, M. Caricato, A. V. Marenich, J. Bloino, B. G. Janesko, R. Gomperts, B. Mennucci, H. P. Hratchian, J. V. Ortiz, A. F. Izmaylov, J. L. Sonnenberg, D. Williams-Young, F. Ding, F. Lipparini, F. Egidi, J. Goings, B. Peng, A. Petrone, T. Henderson, D. Ranasinghe, V. G. Zakrzewski, J. Gao, N. Rega, G. Zheng, W. Liang, M. Hada, M. Ehara, K. Toyota, R. Fukuda, J. Hasegawa, M. Ishida, T. Nakajima, Y. Honda, O. Kitao, H. Nakai, T. Vreven, K. Throssell, J. A. Montgomery, Jr., J. E. Peralta, F. Ogliaro, M. J. Bearpark, J. J. Heyd, E. N. Brothers, K. N. Kudin, V. N. Staroverov, T. A. Keith, R. Kobayashi, J. Normand, K. Raghavachari, A. P. Rendell, J. C. Burant, S. S. Iyengar, J. Tomasi, M. Cossi, J. M. Millam, M. Klene, C. Adamo, R. Cammi, J. W. Ochterski, R. L. Martin, K. Morokuma, O. Farkas, J. B. Foresman, and D. J. Fox, Gaussian, Inc., Wallingford CT, 2016.
8. (a) J -D. Chai, M. Head-Gordon, *Phys. Chem. Chem. Phys.* **2008**, *10*, 6615; (b) J. -D. Chai, M. Head-Gordon, *J. Chem. Phys.* **2008**, *128*, 084106.
9. A. V. Marenich, C. J. Cramer, D. G. Truhlar, *J. Phys. Chem. B* **2009**, *113*, 6378.
10. F. Weigend, R. Ahlrichs, *Phys. Chem. Chem. Phys.*, **2005**, *7*, 3297.
